# Supplementary figures and images for: FOXP2 confers oncogenic effects in prostate cancer (part 1 of 2)
Source: eLife. 2023 Sep 5;12:e81258. doi: 10.7554/eLife.81258 (PMC10513481; doi:10.7554/eLife.81258)

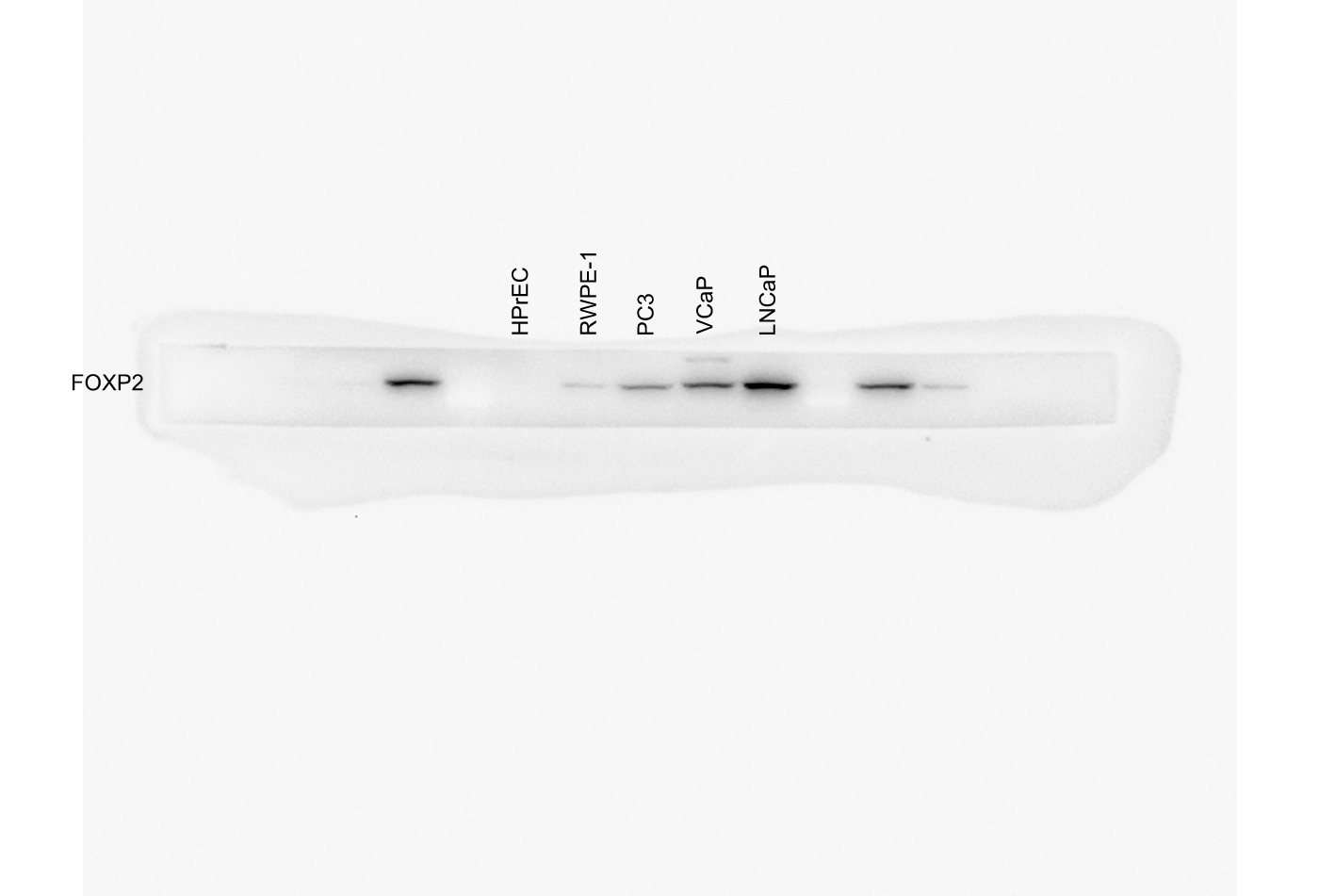

Supplement: Figure 1—source data 1. [file elife-81258-fig1-data1.zip › Figure 1-source data 1/Uncropped blot for Figure 1I/FOXP2.tif]

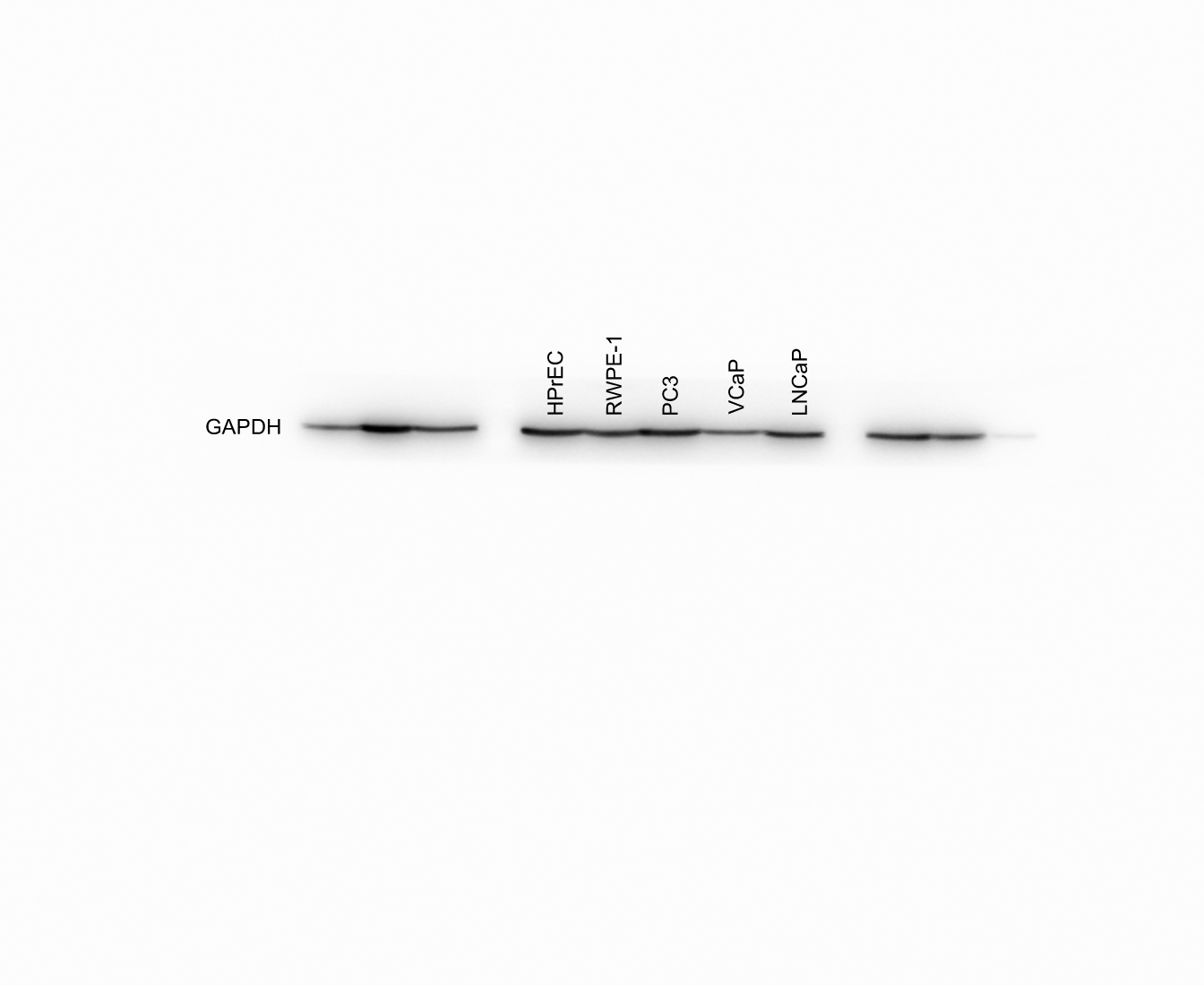

Supplement: Figure 1—source data 1. [file elife-81258-fig1-data1.zip › Figure 1-source data 1/Uncropped blot for Figure 1I/GAPDH.tif]

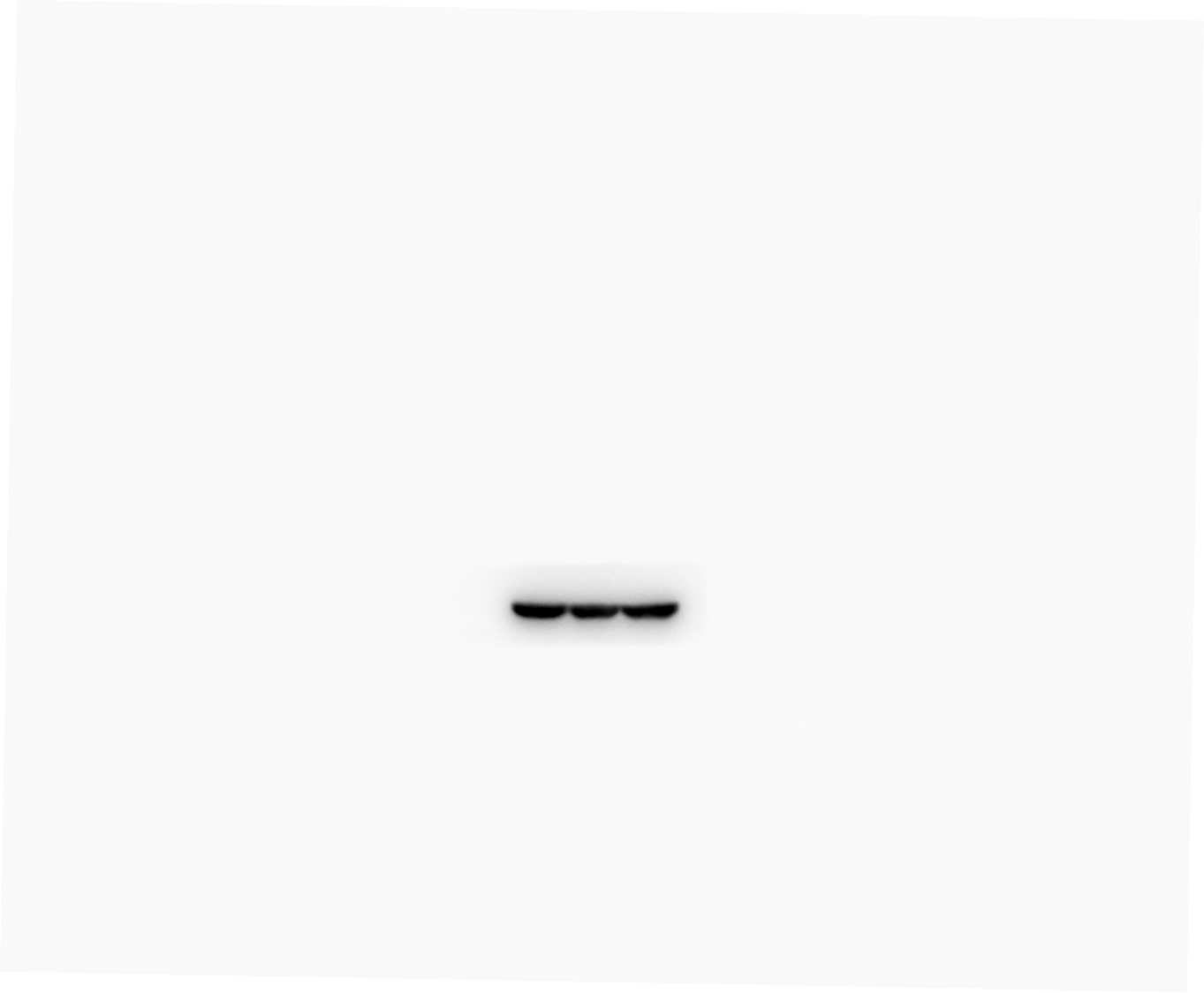

Supplement: Figure 1—source data 2. [file elife-81258-fig1-data2.zip › Figure 1-source data 2/Original files for Figure 1N/PC3-ACTIN.tif]

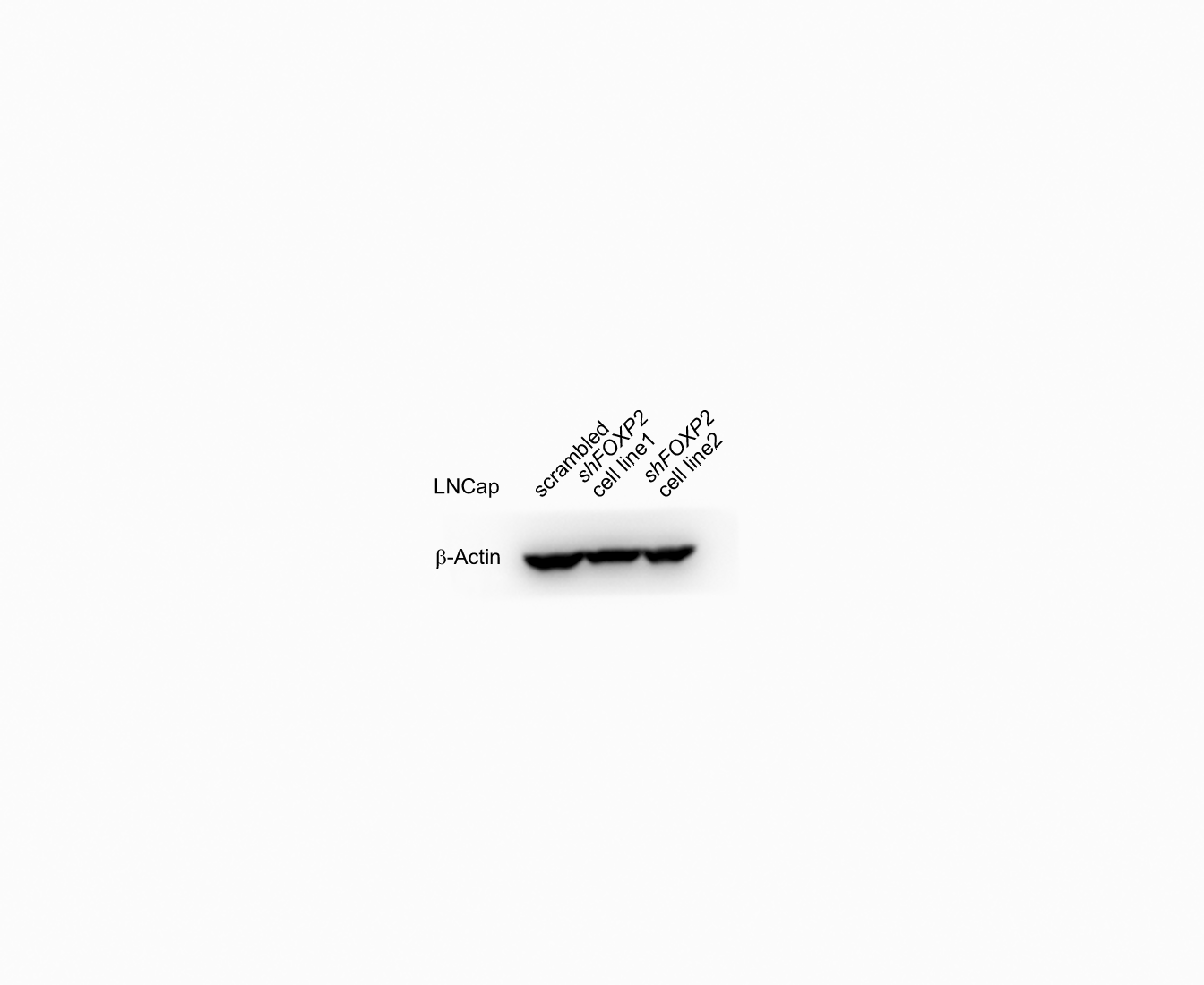

Supplement: Figure 1—source data 2. [file elife-81258-fig1-data2.zip › Figure 1-source data 2/Uncropped blot for Figure 1N/LNCaP_Actin.tif]

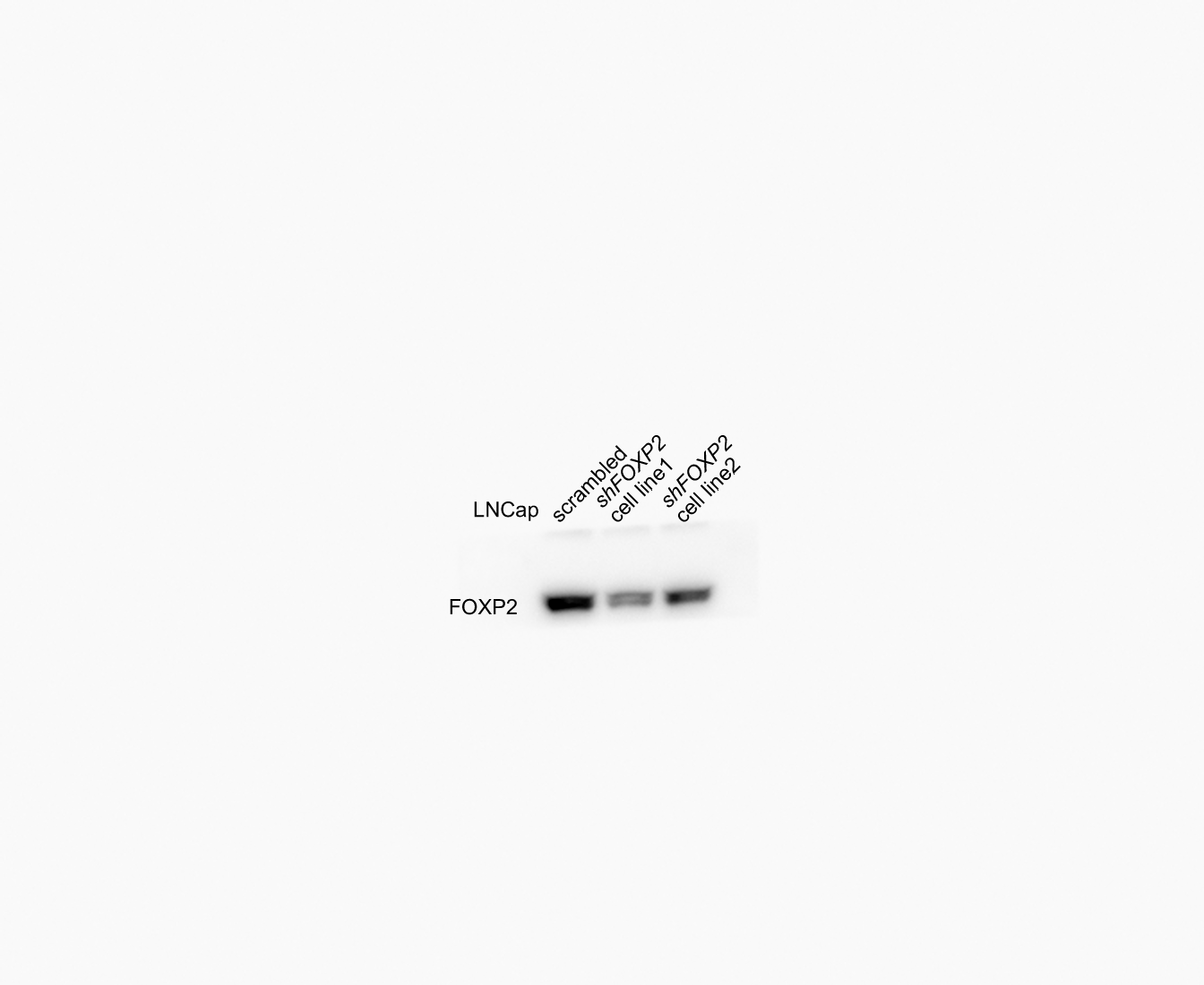

Supplement: Figure 1—source data 2. [file elife-81258-fig1-data2.zip › Figure 1-source data 2/Uncropped blot for Figure 1N/LNCaP_FOXP2.tif]

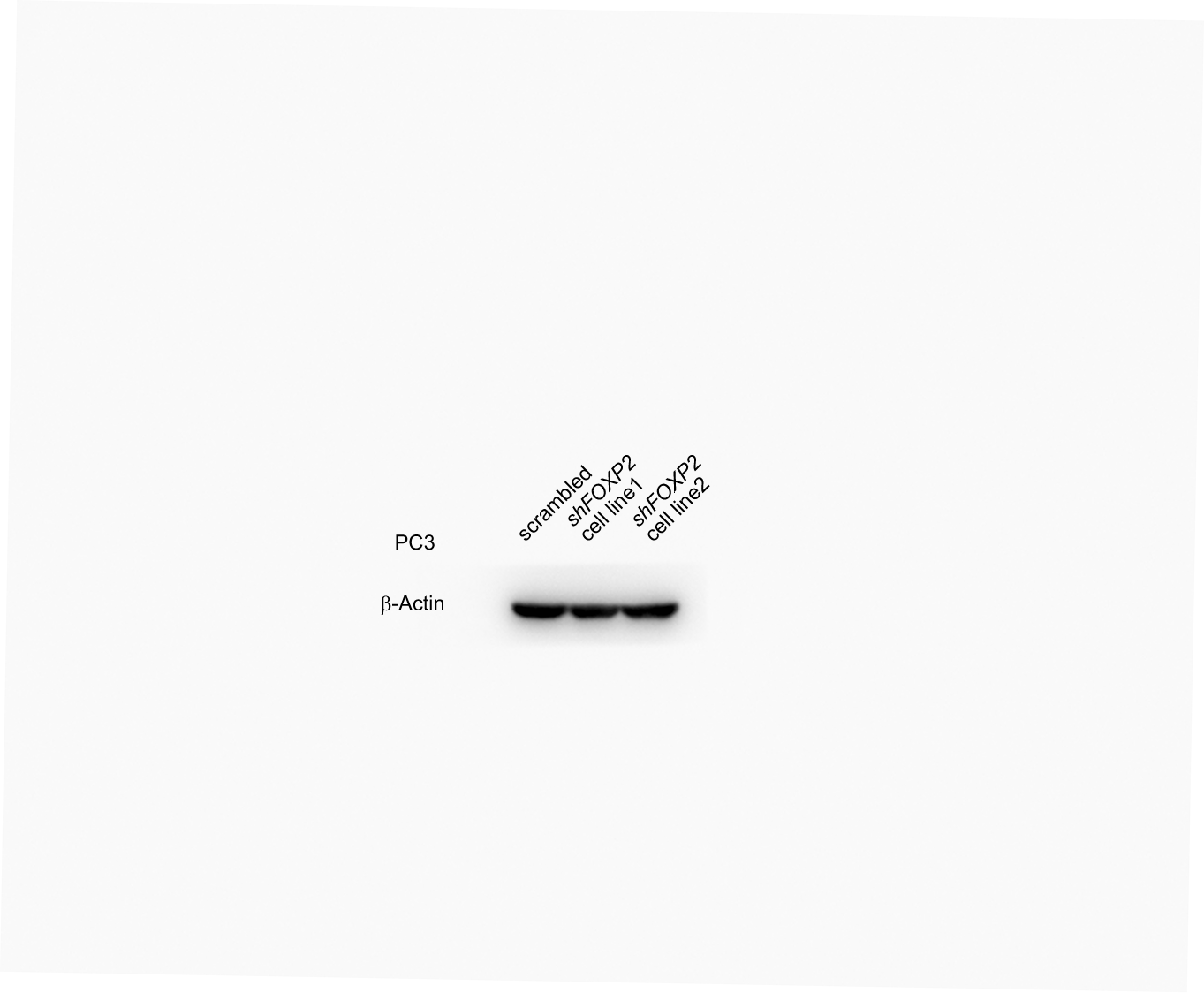

Supplement: Figure 1—source data 2. [file elife-81258-fig1-data2.zip › Figure 1-source data 2/Uncropped blot for Figure 1N/PC3_Actin.tif]

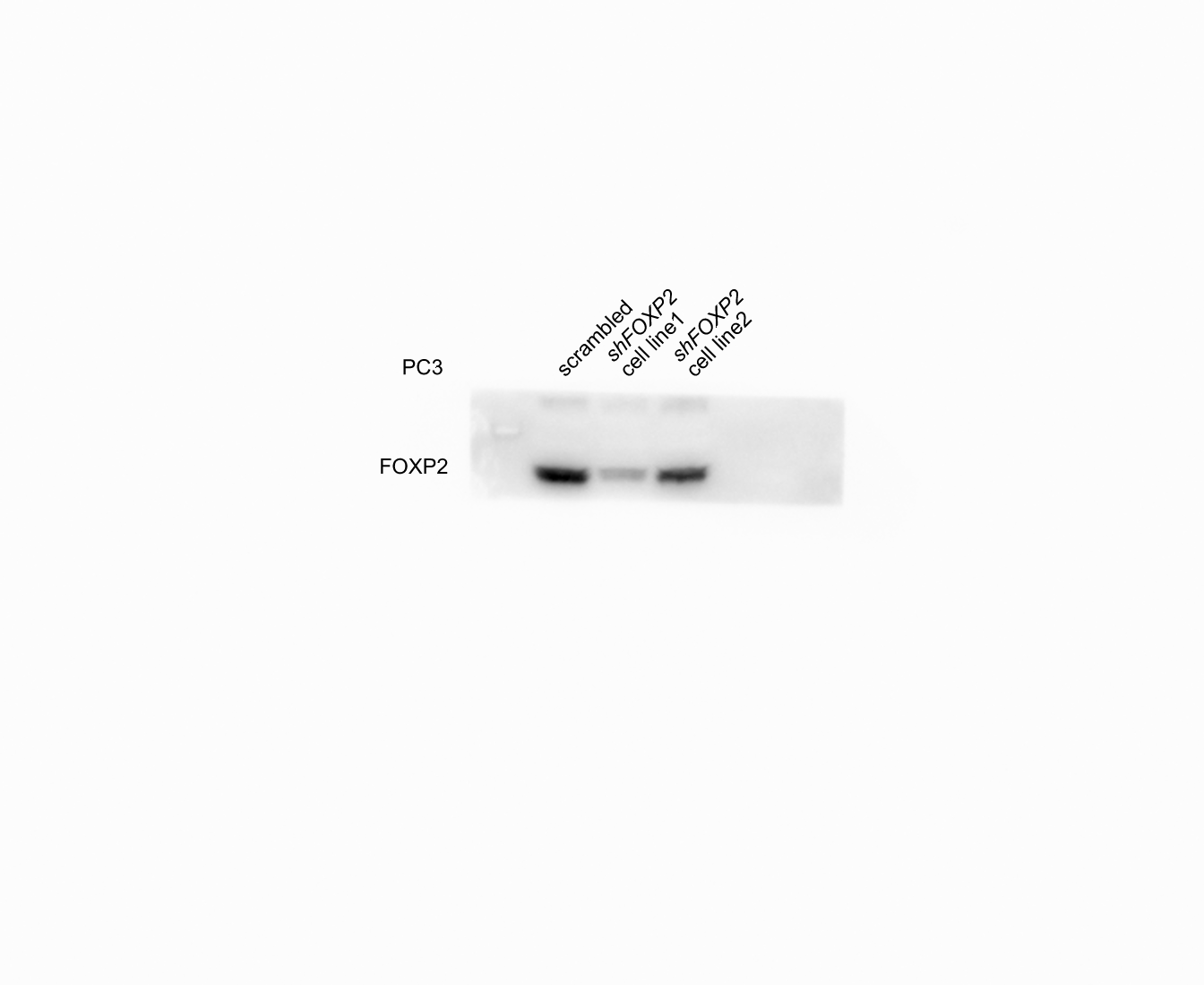

Supplement: Figure 1—source data 2. [file elife-81258-fig1-data2.zip › Figure 1-source data 2/Uncropped blot for Figure 1N/PC3_FOXP2.tif]

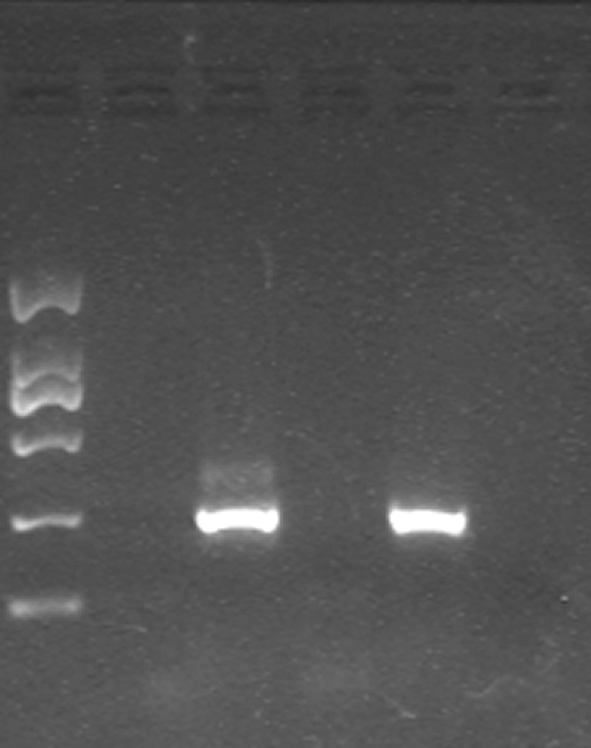

Supplement: Figure 1—figure supplement 1—source data 1. [file elife-81258-fig1-figsupp1-data1.zip › Figure 1-figure supplement 1-source data 1/raw gel for Figure1-figure supplement 1A.jpg]

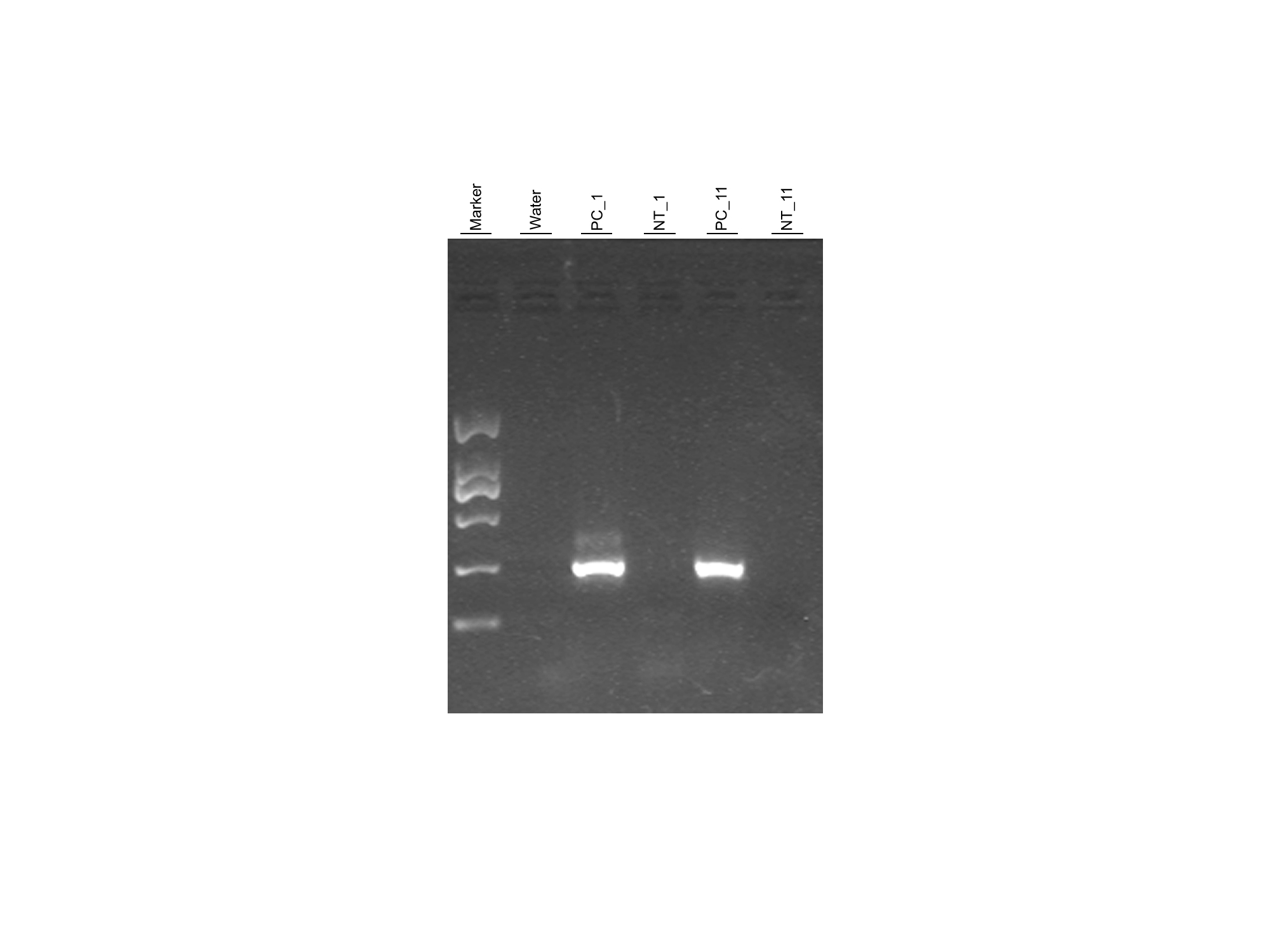

Supplement: Figure 1—figure supplement 1—source data 1. [file elife-81258-fig1-figsupp1-data1.zip › Figure 1-figure supplement 1-source data 1/Uncropped gel for Figure1-figure supplement 1A.jpg]

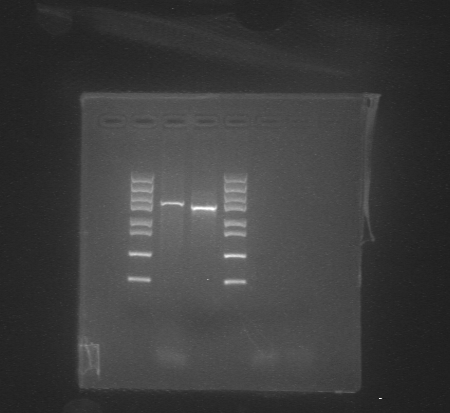

Supplement: Figure 1—figure supplement 1—source data 2. [file elife-81258-fig1-figsupp1-data2.zip › Figure 1-figure supplement 1-source data 2/raw gel for Figure1-figure supplement 1B.jpg]

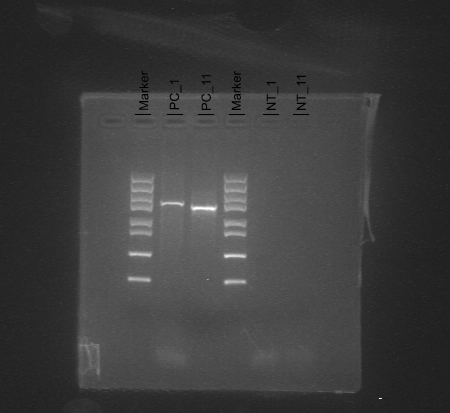

Supplement: Figure 1—figure supplement 1—source data 2. [file elife-81258-fig1-figsupp1-data2.zip › Figure 1-figure supplement 1-source data 2/uncropped gel for Figure1-figure supplement 1B.jpg]

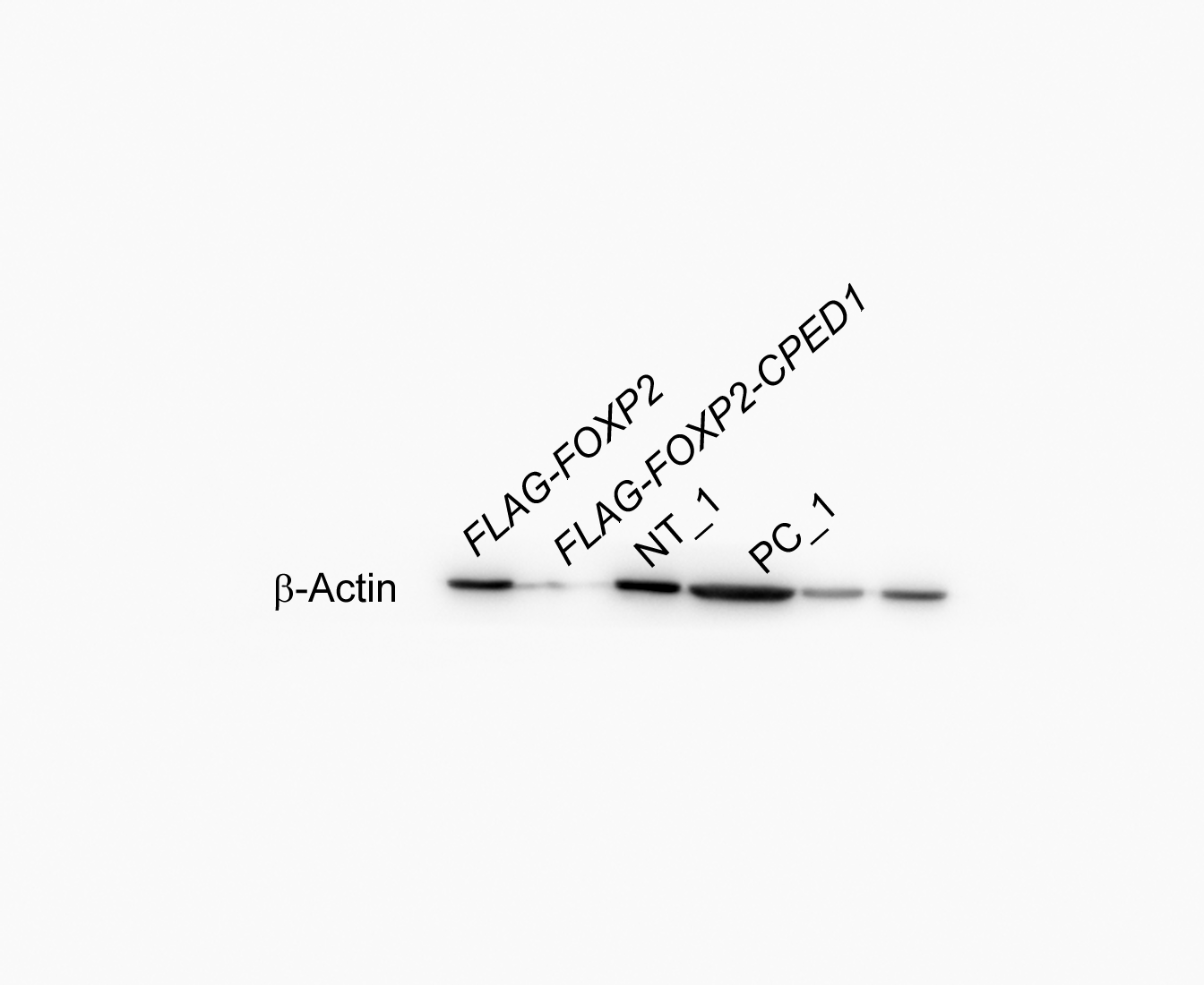

Supplement: Figure 1—figure supplement 1—source data 3. [file elife-81258-fig1-figsupp1-data3.zip › Figure 1-figure supplement 1-source data 3/Uncropped blot for Figure1-figure supplement 1G/Actin.tif]

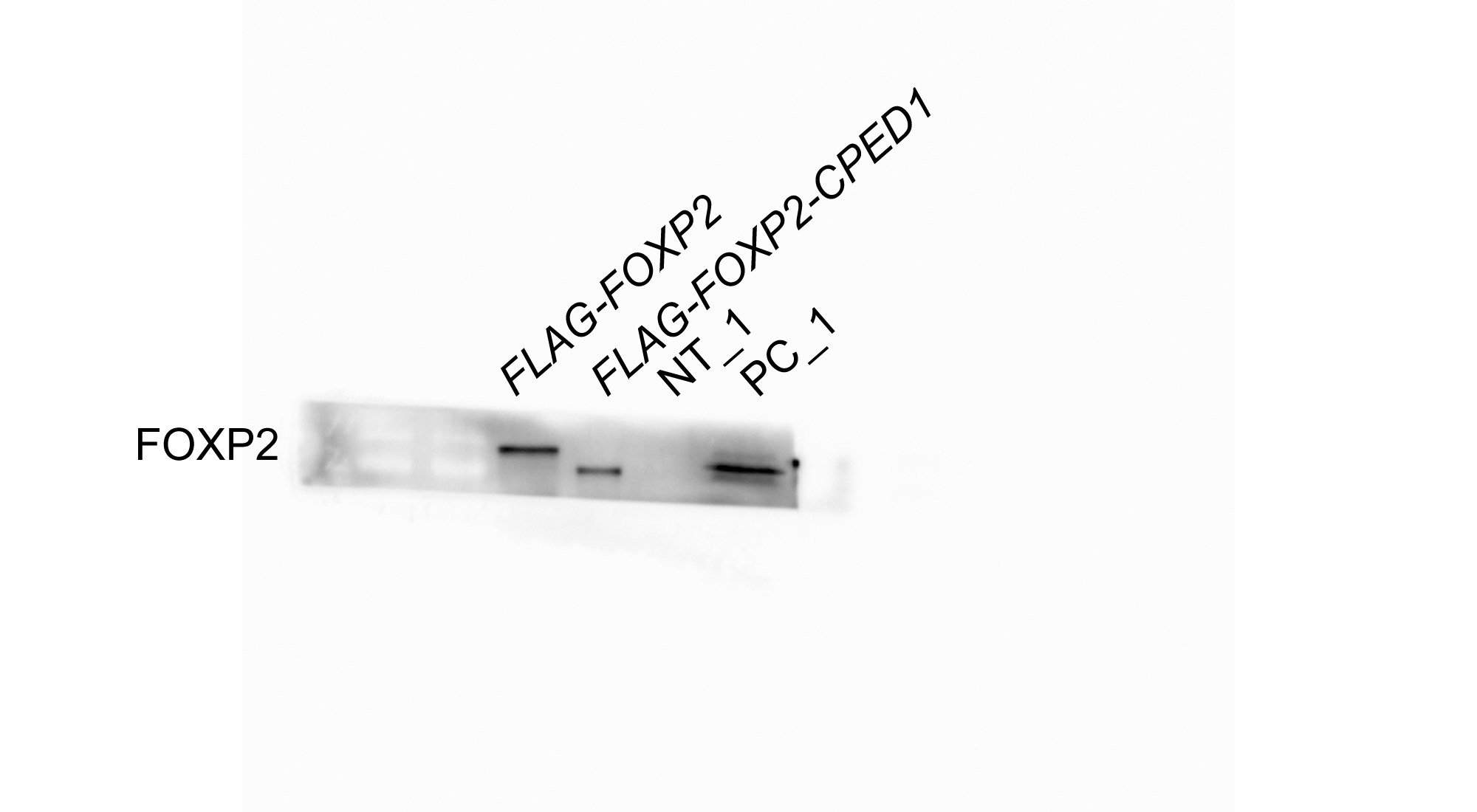

Supplement: Figure 1—figure supplement 1—source data 3. [file elife-81258-fig1-figsupp1-data3.zip › Figure 1-figure supplement 1-source data 3/Uncropped blot for Figure1-figure supplement 1G/FOXP2.tif]

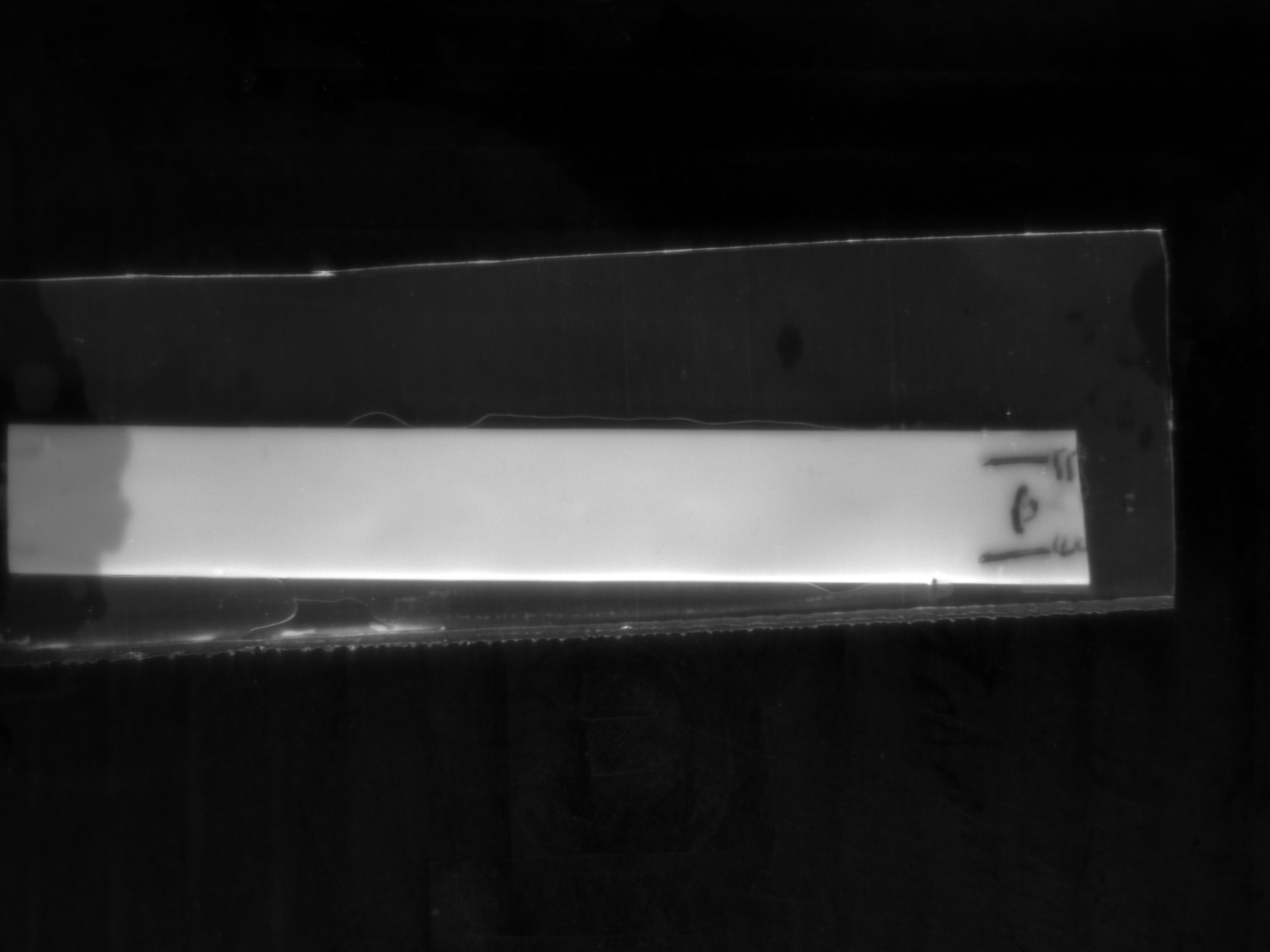

Supplement: Figure 1—figure supplement 1—source data 4. [file elife-81258-fig1-figsupp1-data4.zip › Figure 1-figure supplement 1-source data 4/Original files for Figure 1-figure supplement 1K/ACTIN-White-light image corresponding to WB image.BMP]

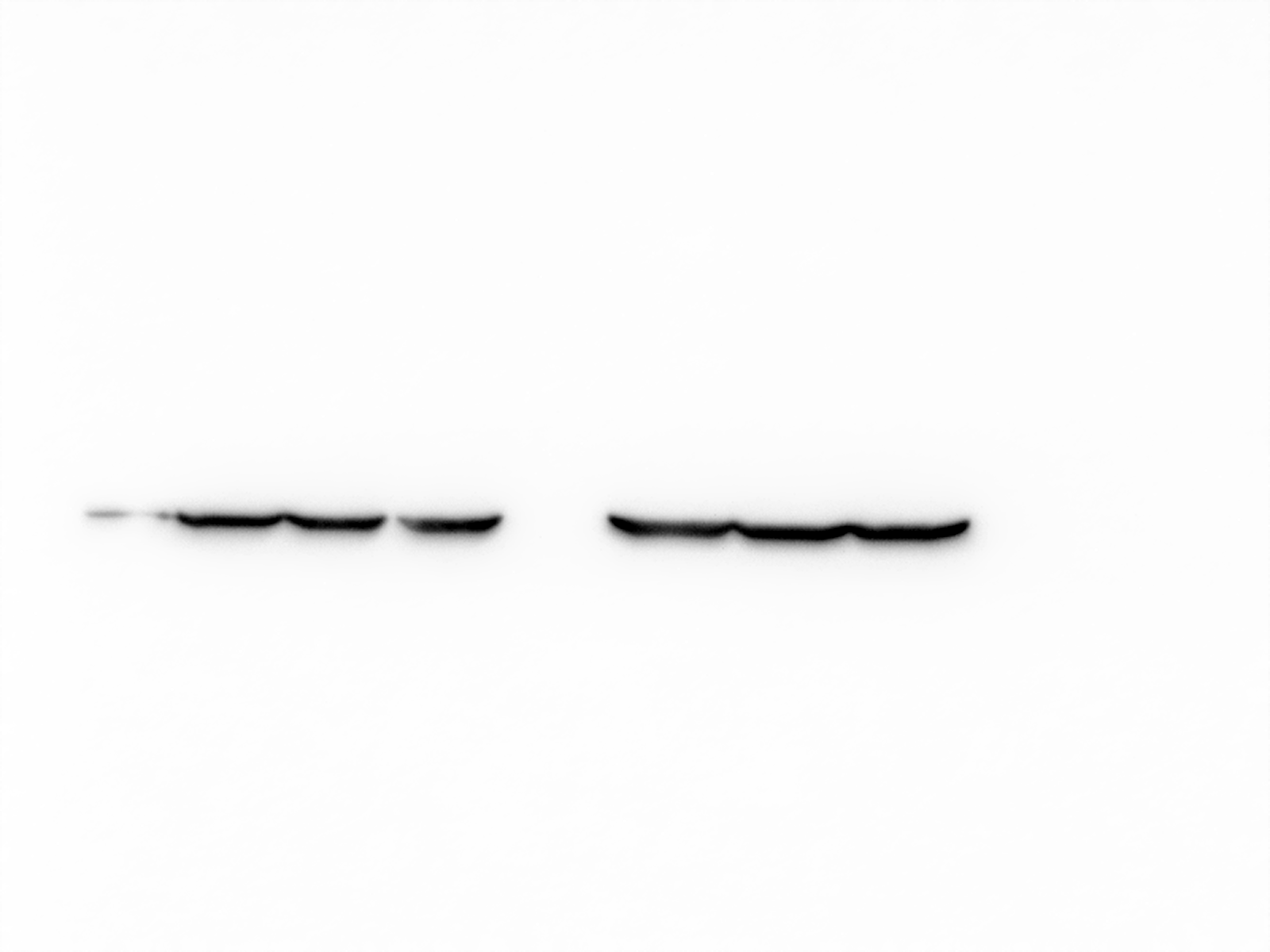

Supplement: Figure 1—figure supplement 1—source data 4. [file elife-81258-fig1-figsupp1-data4.zip › Figure 1-figure supplement 1-source data 4/Original files for Figure 1-figure supplement 1K/ACTIN.BMP]

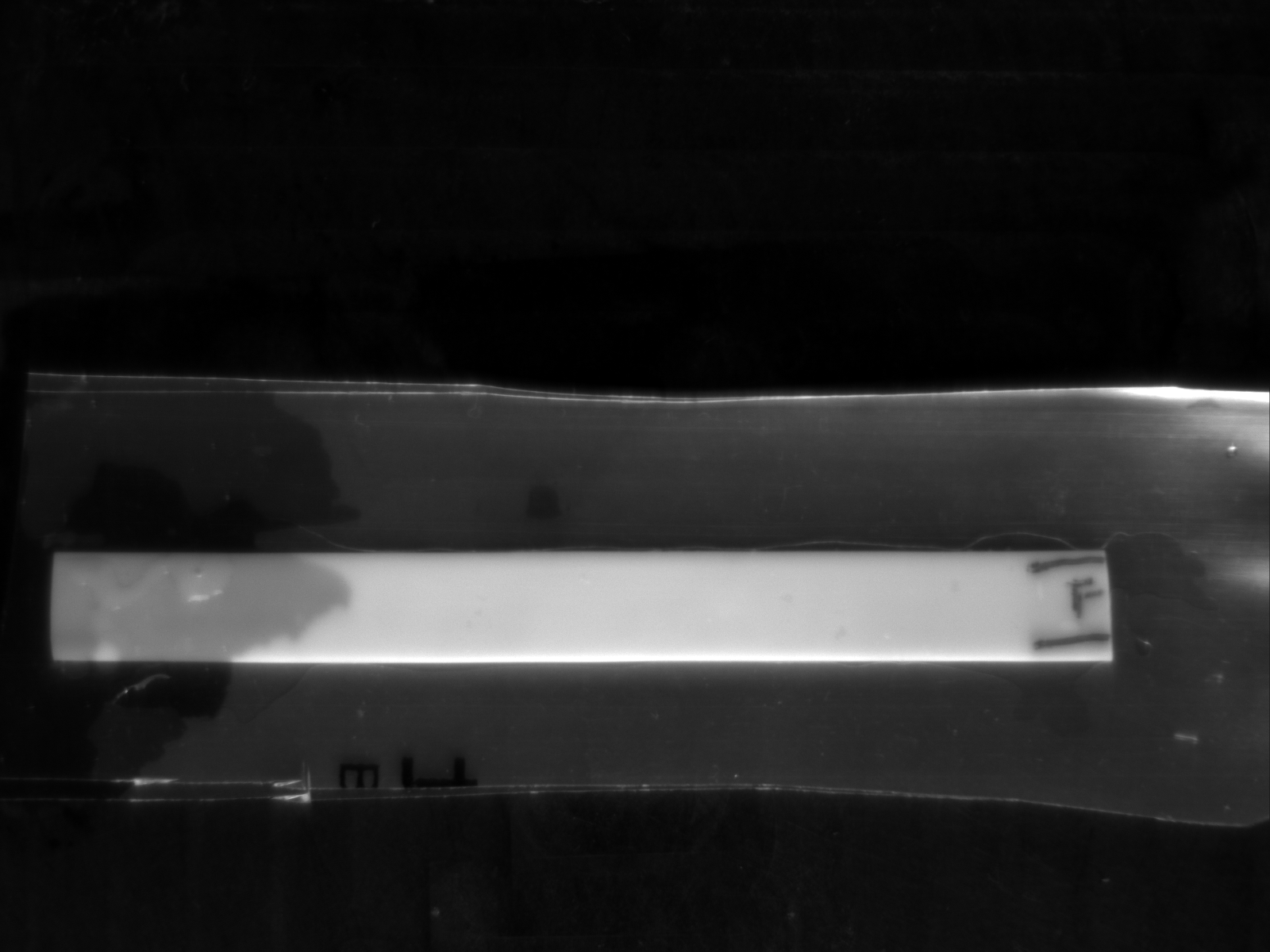

Supplement: Figure 1—figure supplement 1—source data 4. [file elife-81258-fig1-figsupp1-data4.zip › Figure 1-figure supplement 1-source data 4/Original files for Figure 1-figure supplement 1K/FOXP2-White-light image corresponding to WB image.BMP]

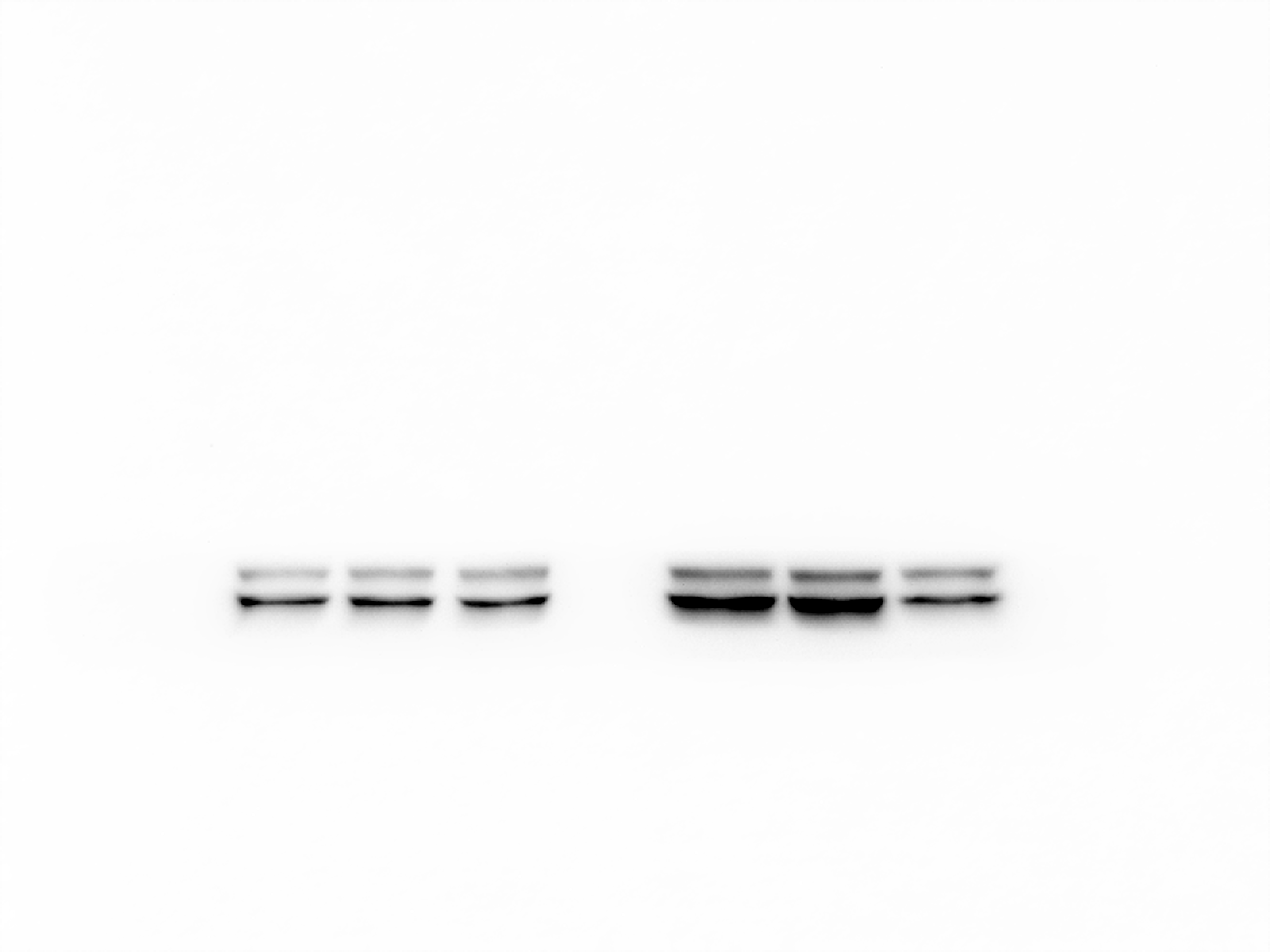

Supplement: Figure 1—figure supplement 1—source data 4. [file elife-81258-fig1-figsupp1-data4.zip › Figure 1-figure supplement 1-source data 4/Original files for Figure 1-figure supplement 1K/FOXP2.BMP]

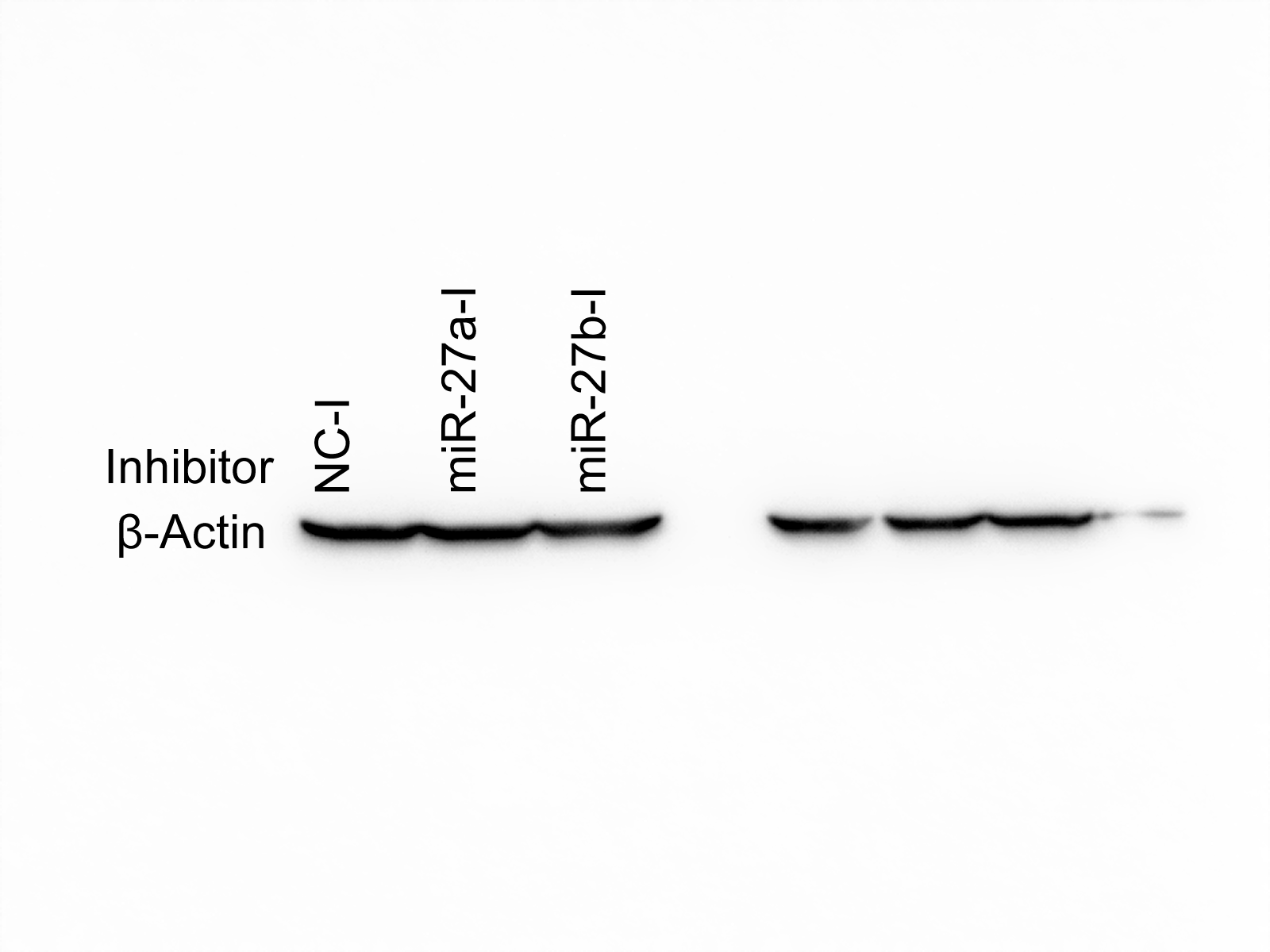

Supplement: Figure 1—figure supplement 1—source data 4. [file elife-81258-fig1-figsupp1-data4.zip › Figure 1-figure supplement 1-source data 4/Uncropped blots for Figure1-figure supplement 1K/Actin.tif]

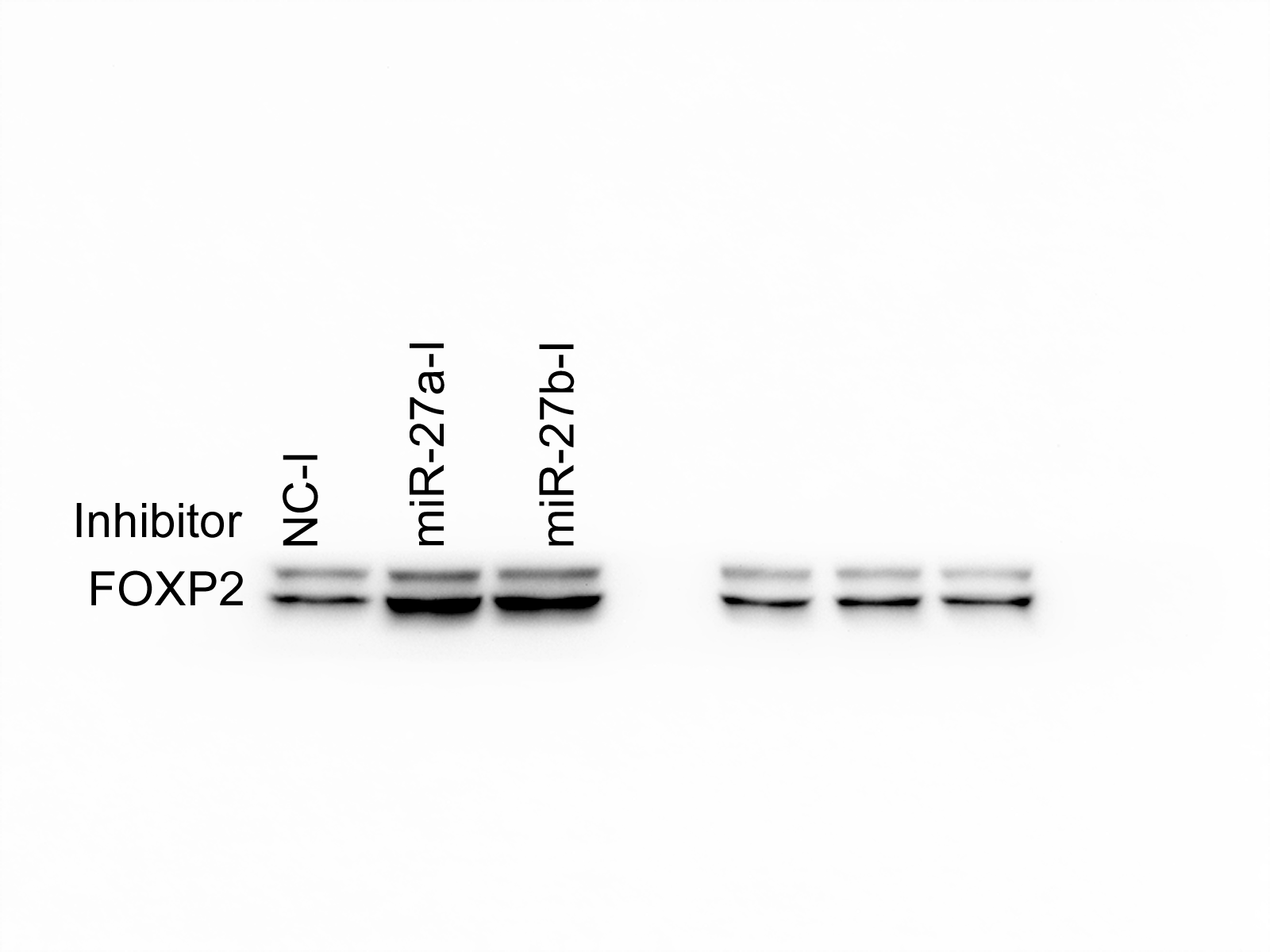

Supplement: Figure 1—figure supplement 1—source data 4. [file elife-81258-fig1-figsupp1-data4.zip › Figure 1-figure supplement 1-source data 4/Uncropped blots for Figure1-figure supplement 1K/FOXP2.tif]

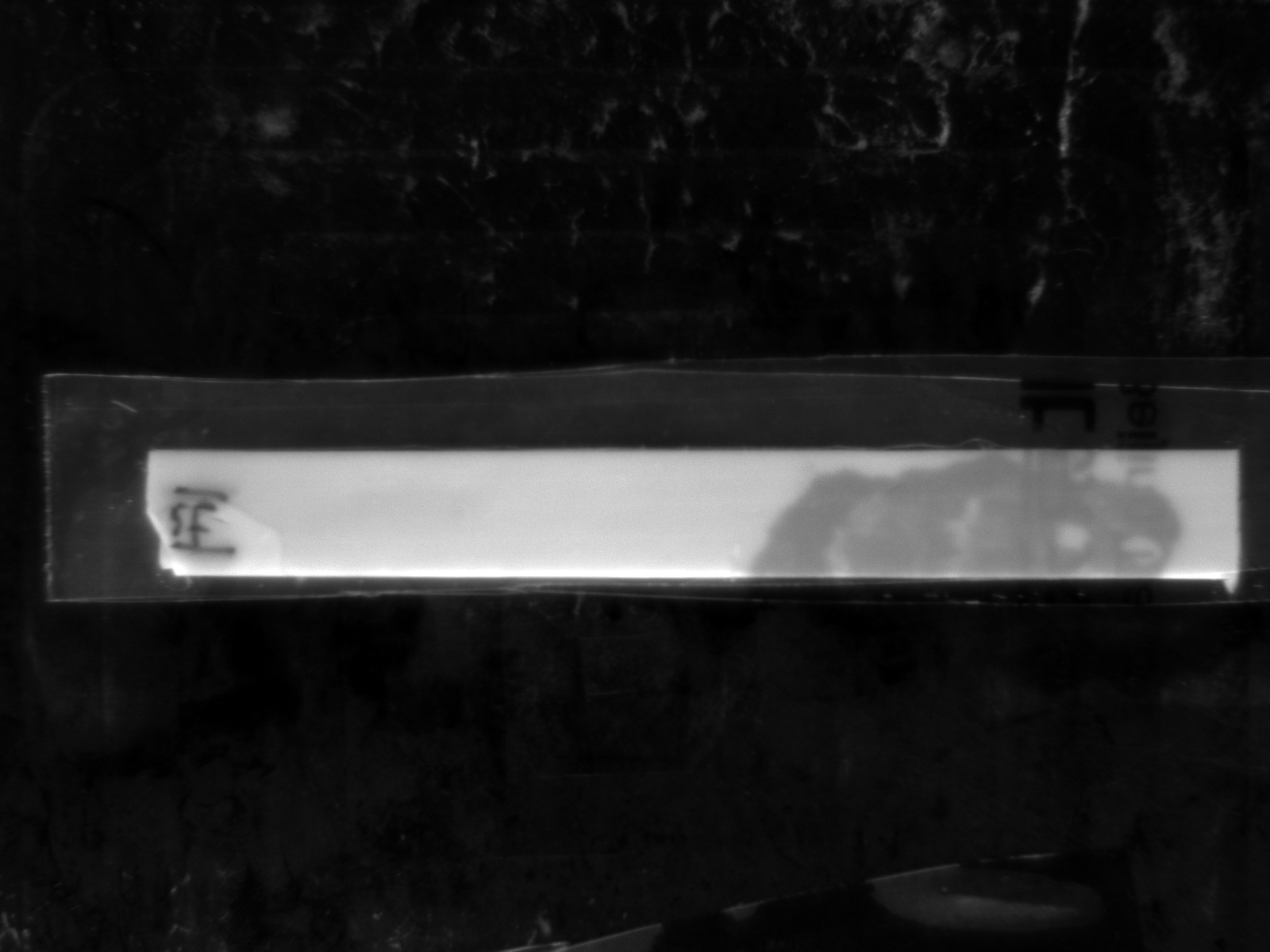

Supplement: Figure 1—figure supplement 1—source data 5. [file elife-81258-fig1-figsupp1-data5.zip › Figure 1-figure supplement 1-source data 5/Original files for Figure 1-figure supplement 1L/FOXP2-White-light image corresponding to WB image.BMP]

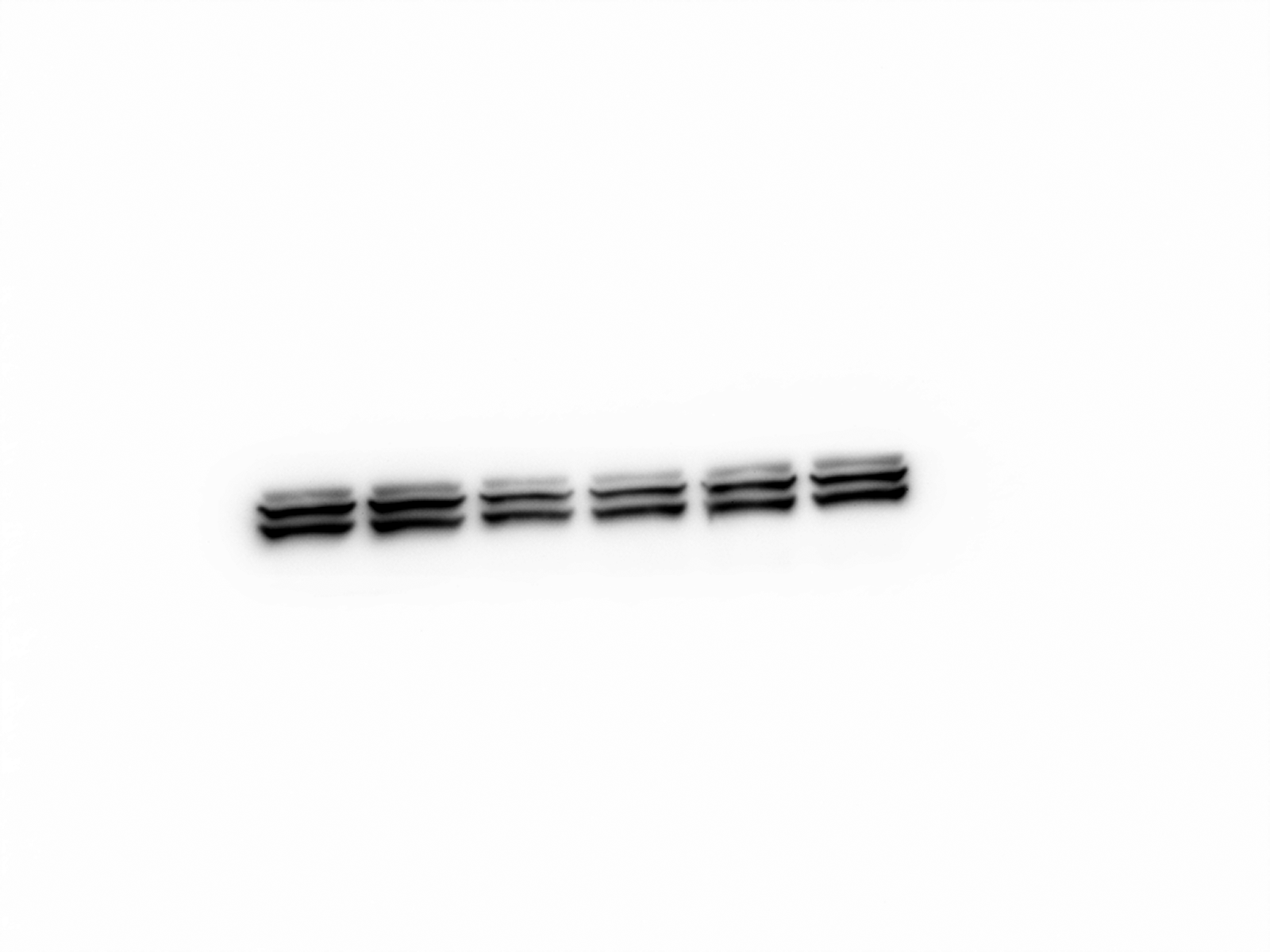

Supplement: Figure 1—figure supplement 1—source data 5. [file elife-81258-fig1-figsupp1-data5.zip › Figure 1-figure supplement 1-source data 5/Original files for Figure 1-figure supplement 1L/FOXP2.tif]

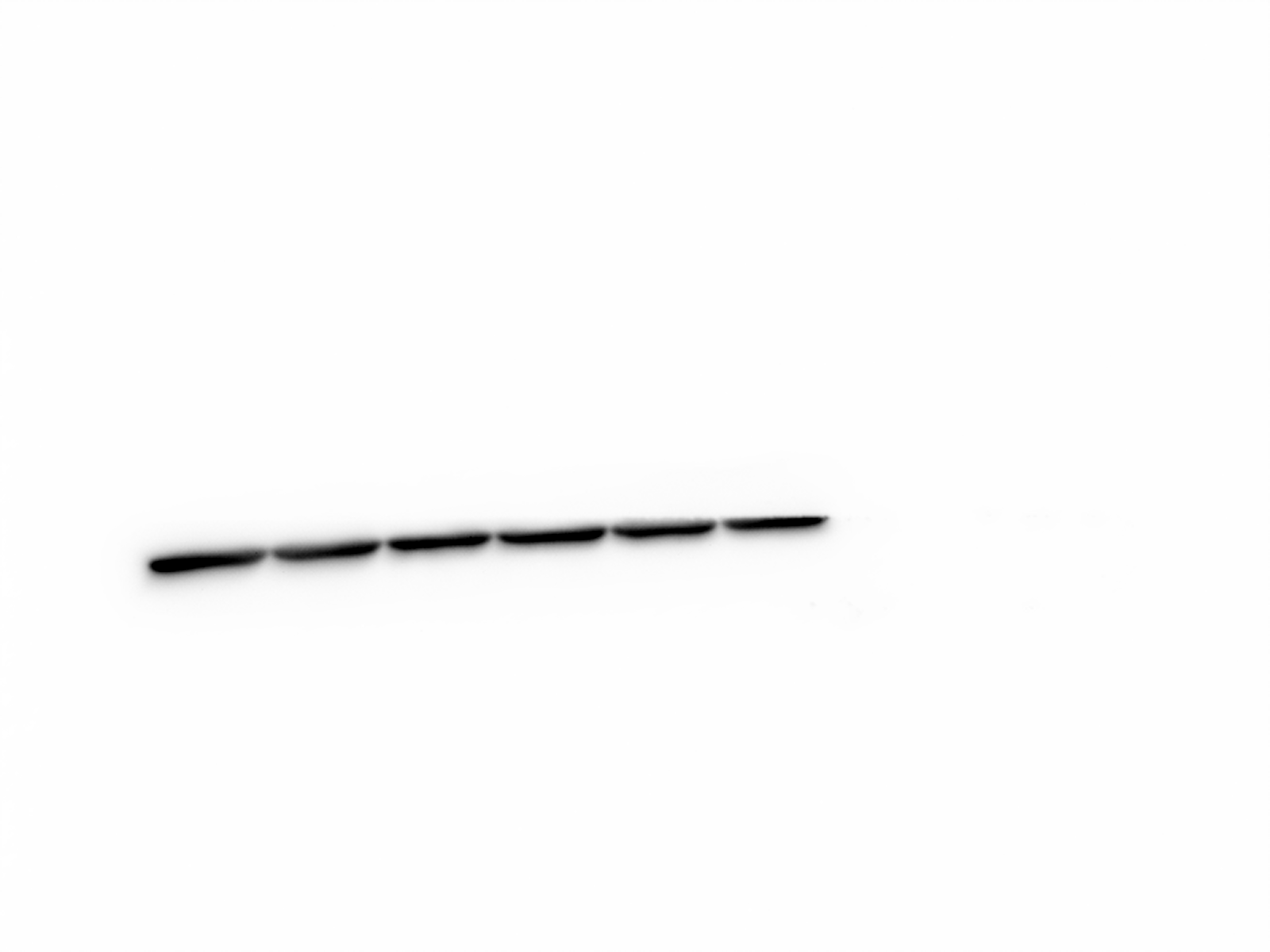

Supplement: Figure 1—figure supplement 1—source data 5. [file elife-81258-fig1-figsupp1-data5.zip › Figure 1-figure supplement 1-source data 5/Original files for Figure 1-figure supplement 1L/GAPDH-30S.BMP]

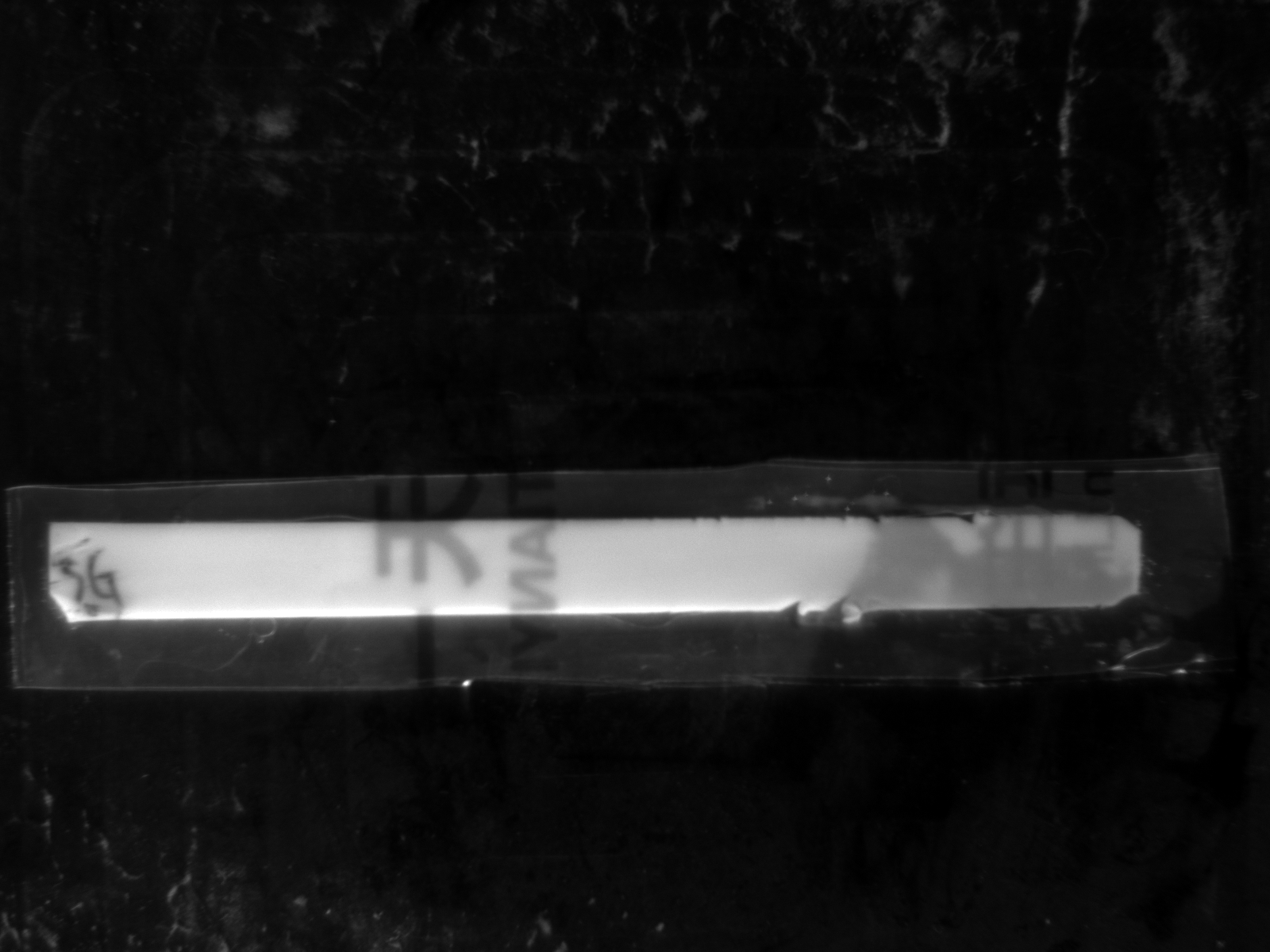

Supplement: Figure 1—figure supplement 1—source data 5. [file elife-81258-fig1-figsupp1-data5.zip › Figure 1-figure supplement 1-source data 5/Original files for Figure 1-figure supplement 1L/GAPDH-White-light image corresponding to WB image.BMP]

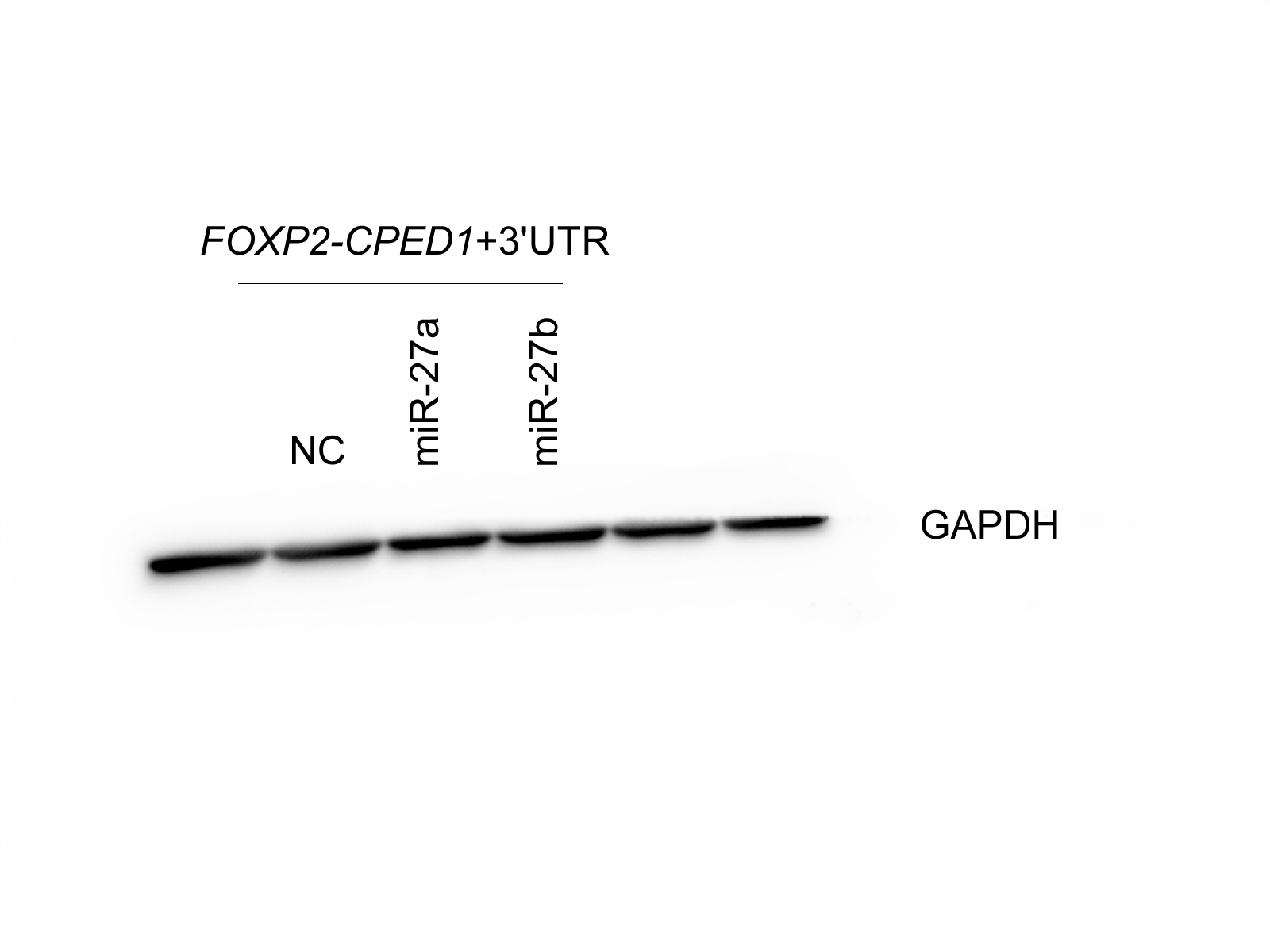

Supplement: Figure 1—figure supplement 1—source data 5. [file elife-81258-fig1-figsupp1-data5.zip › Figure 1-figure supplement 1-source data 5/Uncropped blots for Figure1-figure supplement 1L/2015-01-15-5GAPDH-30S.bmp]

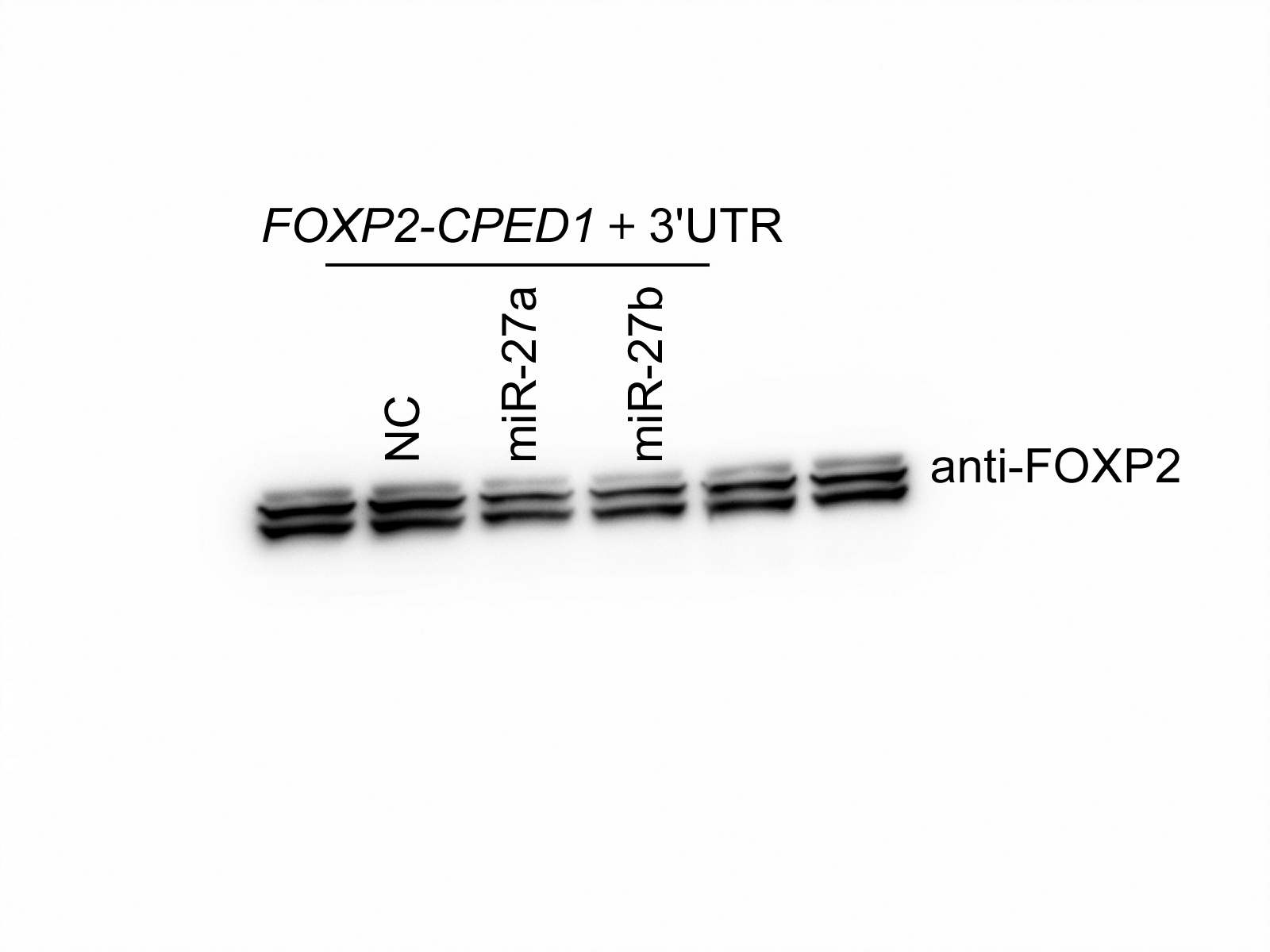

Supplement: Figure 1—figure supplement 1—source data 5. [file elife-81258-fig1-figsupp1-data5.zip › Figure 1-figure supplement 1-source data 5/Uncropped blots for Figure1-figure supplement 1L/FOXP2.tif]

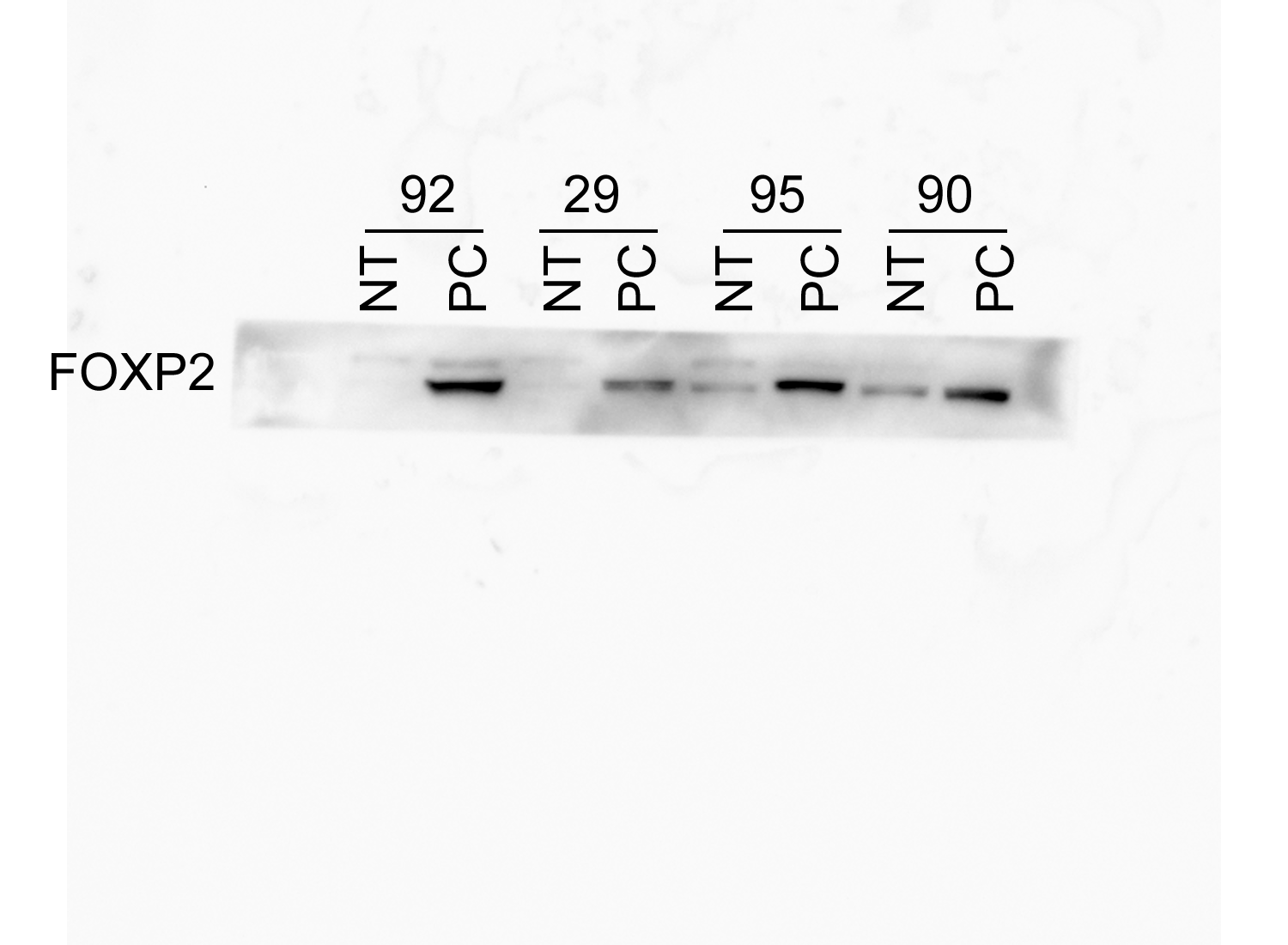

Supplement: Figure 1—figure supplement 2—source data 1. [file elife-81258-fig1-figsupp2-data1.zip › Figure 1-figure supplement 2-source data 1/Uncropped blots for Figure1-figure supplement 2A/Figure 1- figure supplement 2A source data 1.tif]

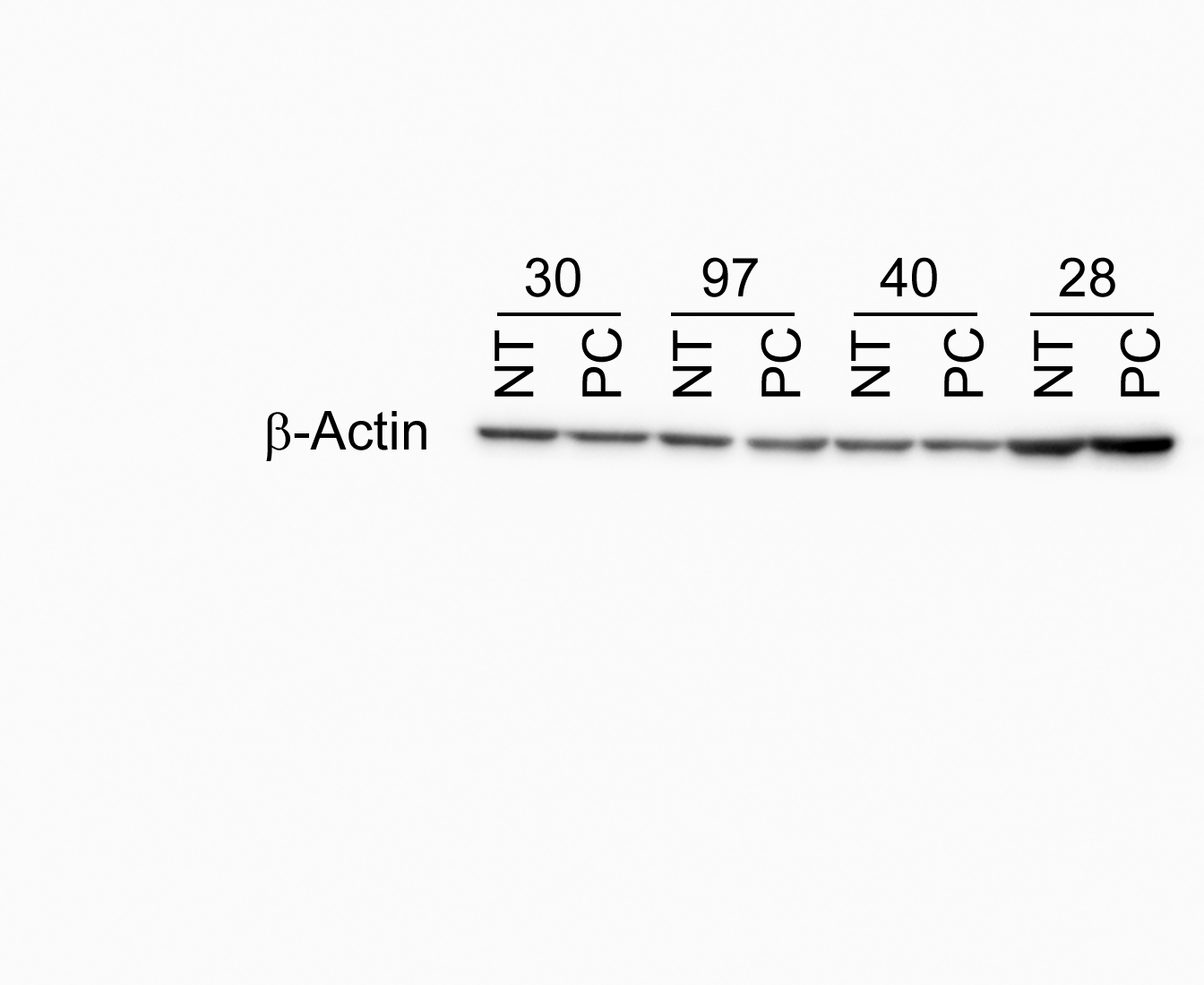

Supplement: Figure 1—figure supplement 2—source data 1. [file elife-81258-fig1-figsupp2-data1.zip › Figure 1-figure supplement 2-source data 1/Uncropped blots for Figure1-figure supplement 2A/Figure 1- figure supplement 2A source data 10.tif]

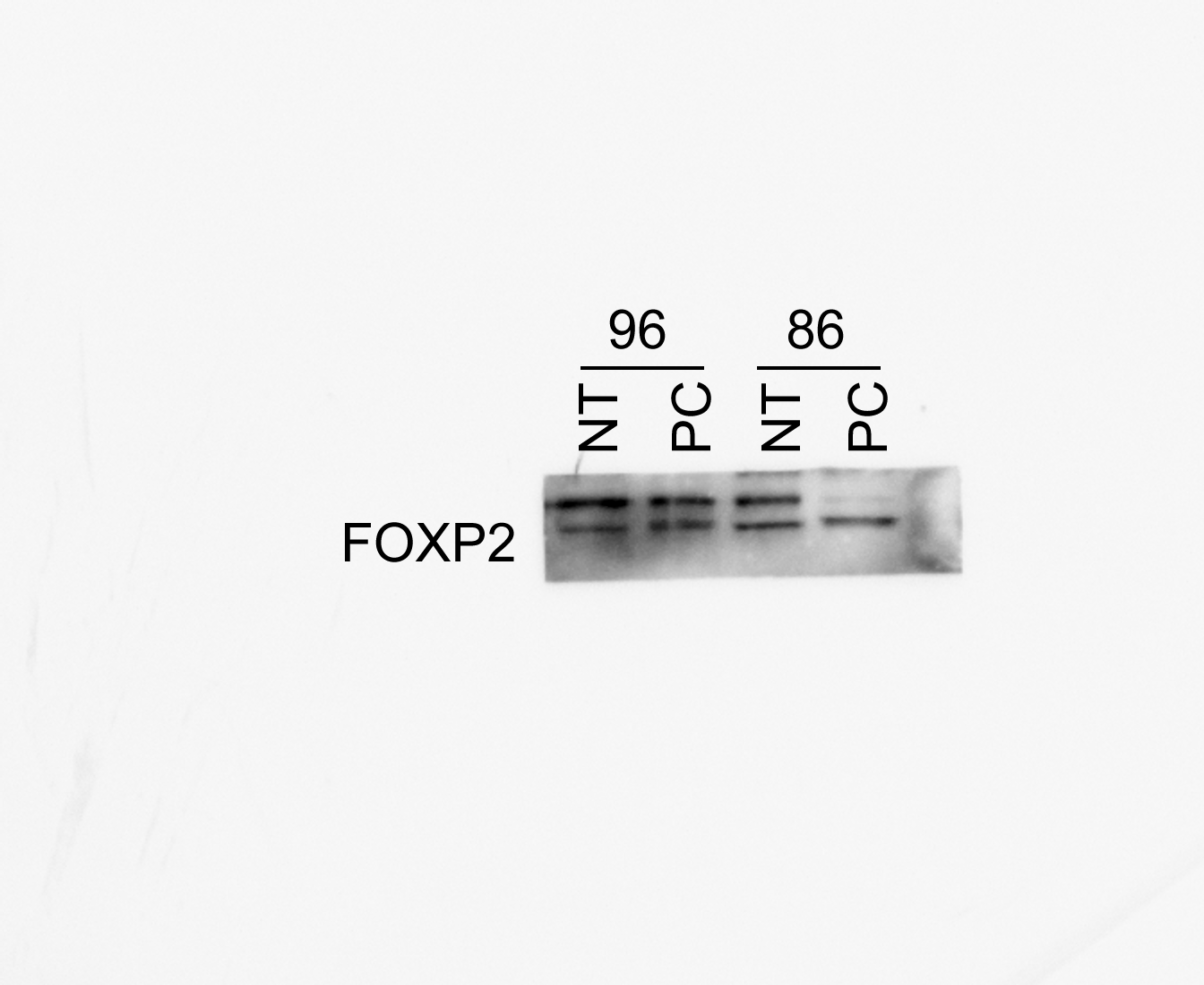

Supplement: Figure 1—figure supplement 2—source data 1. [file elife-81258-fig1-figsupp2-data1.zip › Figure 1-figure supplement 2-source data 1/Uncropped blots for Figure1-figure supplement 2A/Figure 1- figure supplement 2A source data 11.tif]

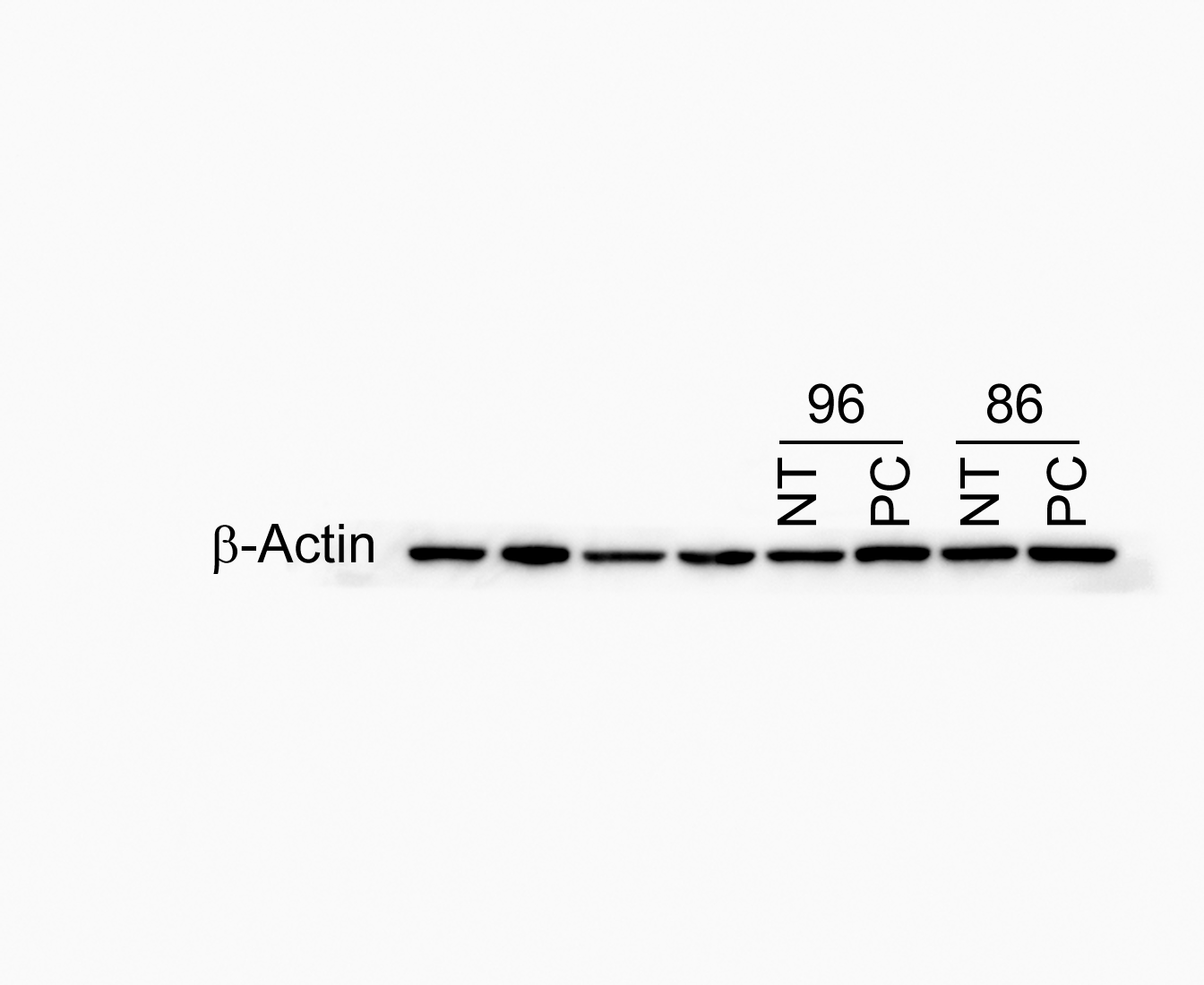

Supplement: Figure 1—figure supplement 2—source data 1. [file elife-81258-fig1-figsupp2-data1.zip › Figure 1-figure supplement 2-source data 1/Uncropped blots for Figure1-figure supplement 2A/Figure 1- figure supplement 2A source data 12.tif]

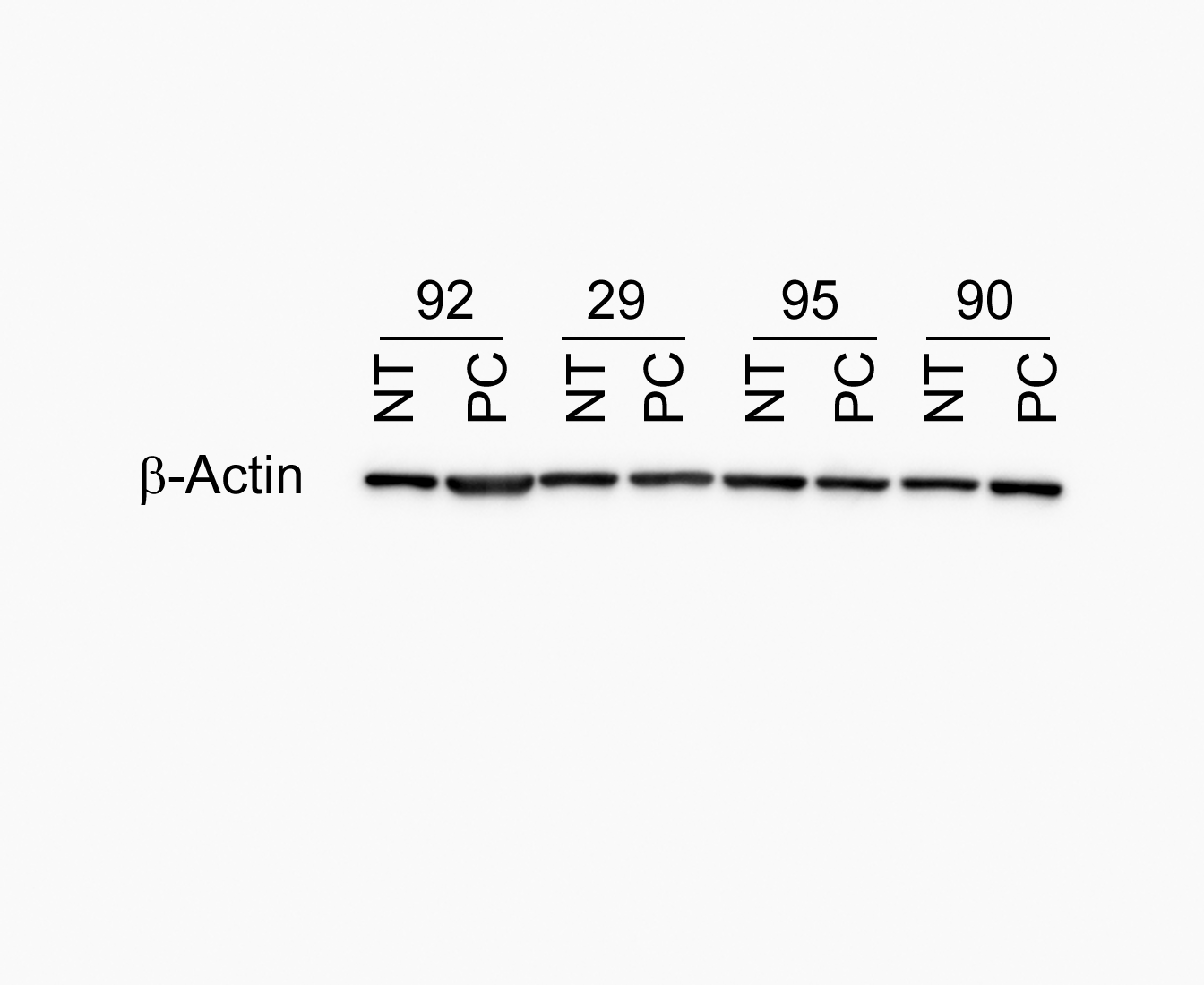

Supplement: Figure 1—figure supplement 2—source data 1. [file elife-81258-fig1-figsupp2-data1.zip › Figure 1-figure supplement 2-source data 1/Uncropped blots for Figure1-figure supplement 2A/Figure 1- figure supplement 2A source data 2.tif]

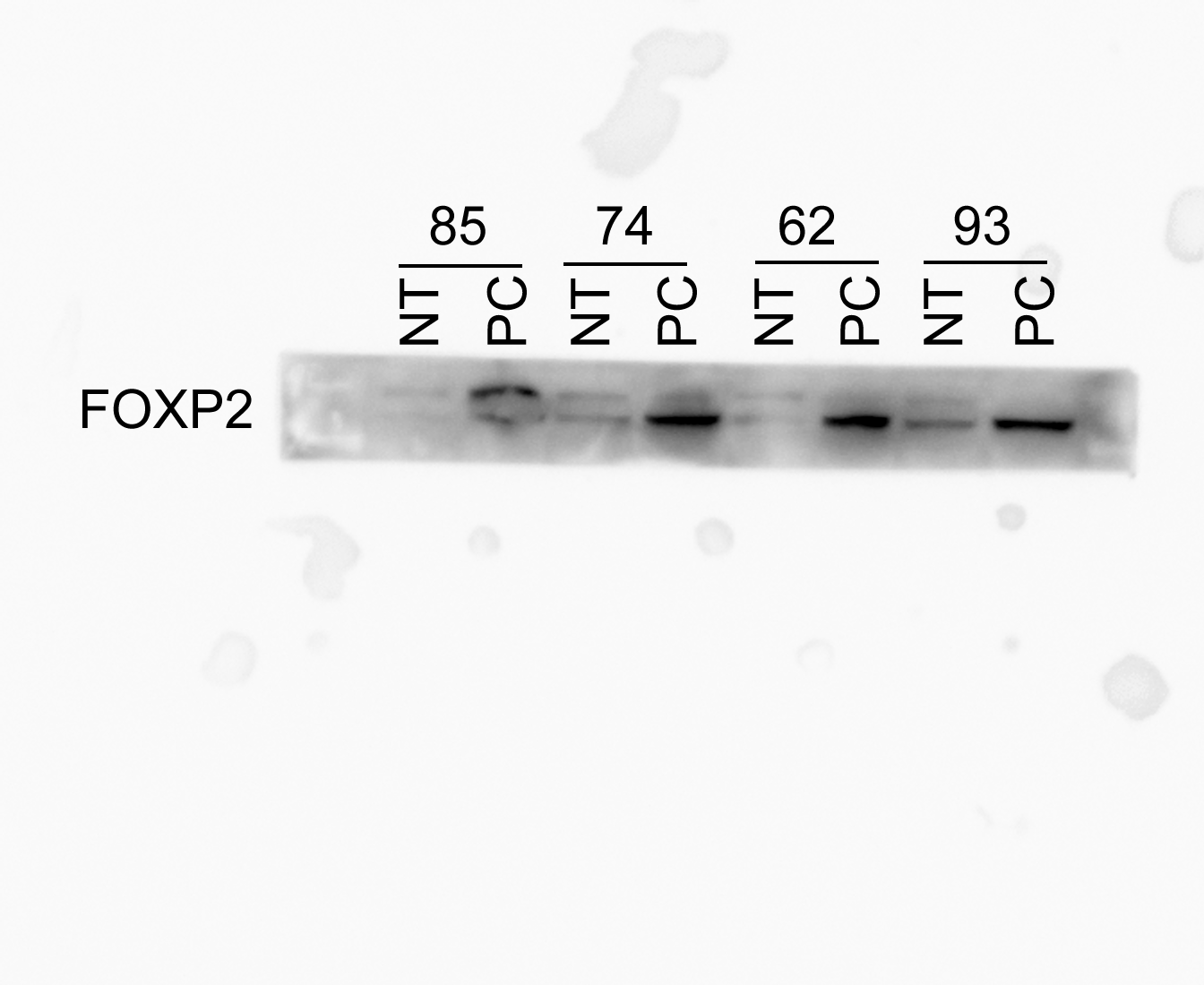

Supplement: Figure 1—figure supplement 2—source data 1. [file elife-81258-fig1-figsupp2-data1.zip › Figure 1-figure supplement 2-source data 1/Uncropped blots for Figure1-figure supplement 2A/Figure 1- figure supplement 2A source data 3.tif]

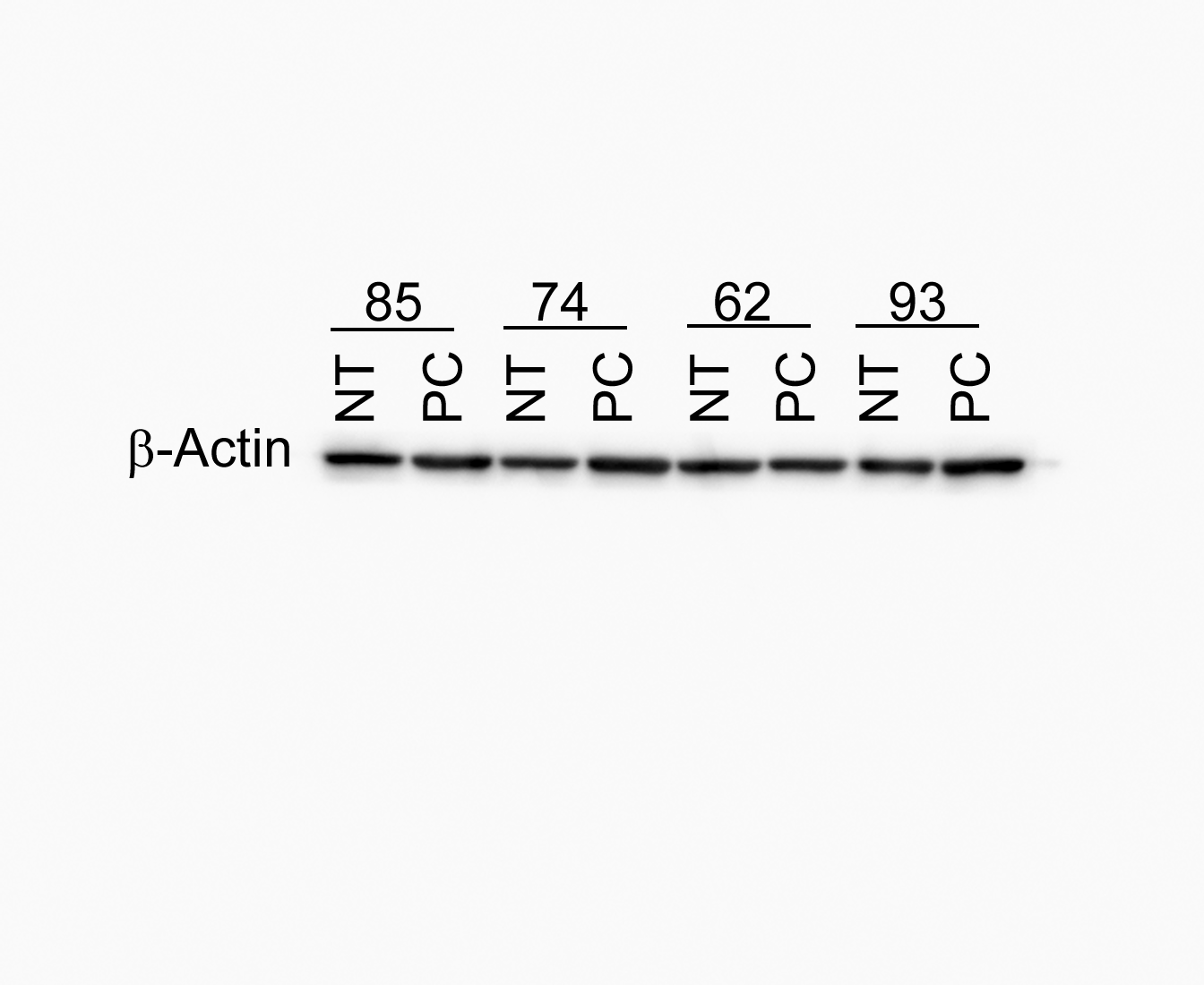

Supplement: Figure 1—figure supplement 2—source data 1. [file elife-81258-fig1-figsupp2-data1.zip › Figure 1-figure supplement 2-source data 1/Uncropped blots for Figure1-figure supplement 2A/Figure 1- figure supplement 2A source data 4.tif]

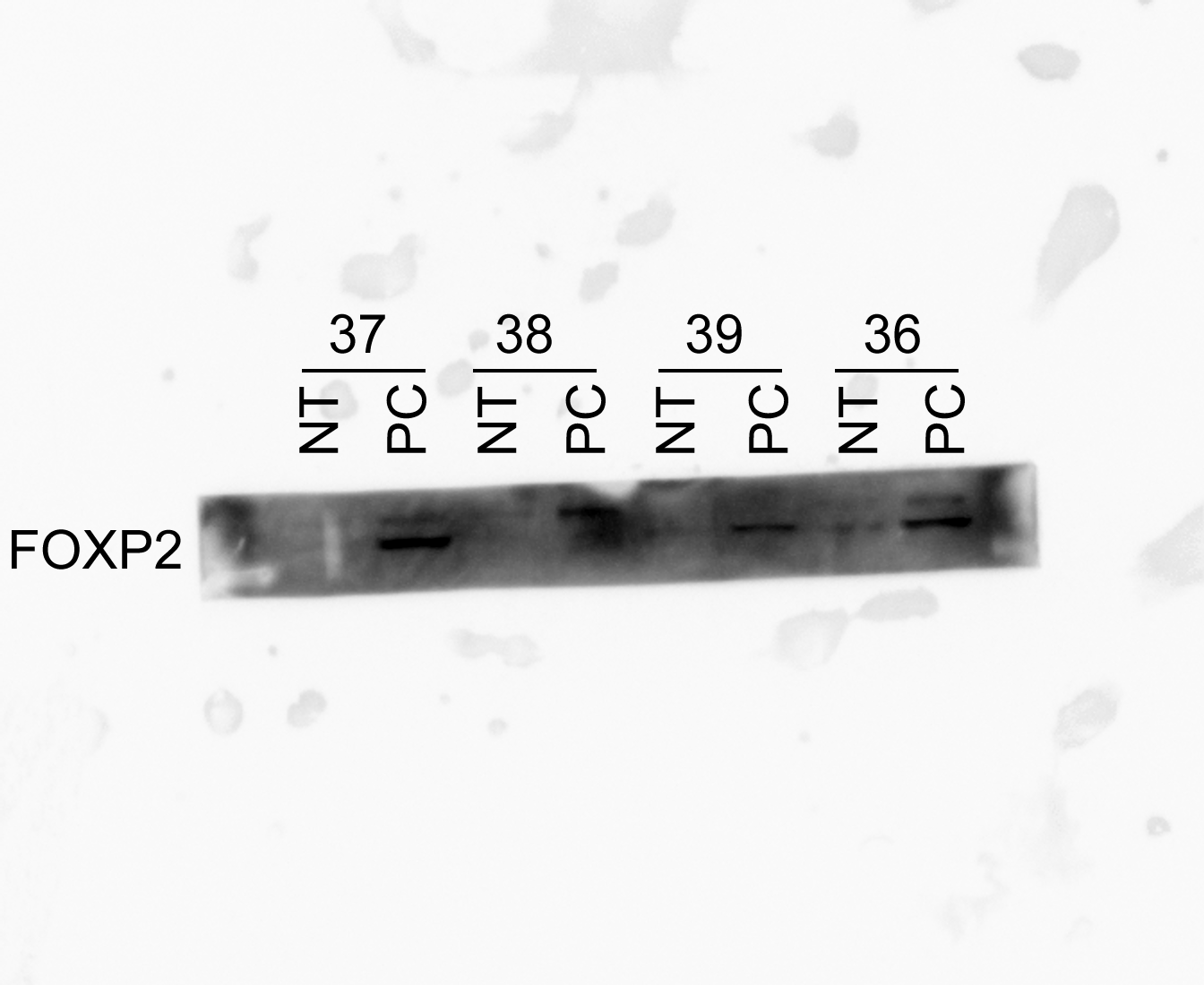

Supplement: Figure 1—figure supplement 2—source data 1. [file elife-81258-fig1-figsupp2-data1.zip › Figure 1-figure supplement 2-source data 1/Uncropped blots for Figure1-figure supplement 2A/Figure 1- figure supplement 2A source data 5.tif]

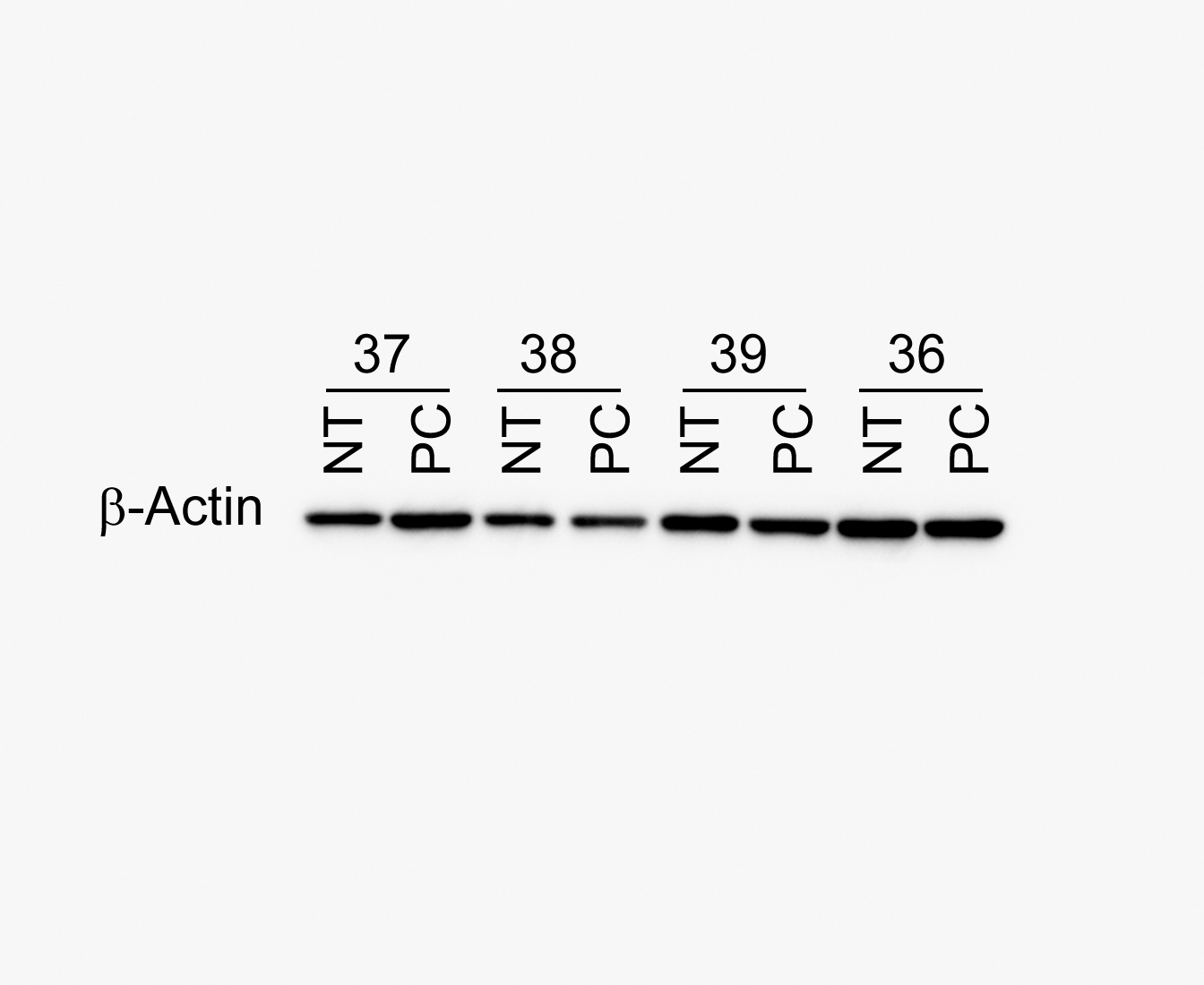

Supplement: Figure 1—figure supplement 2—source data 1. [file elife-81258-fig1-figsupp2-data1.zip › Figure 1-figure supplement 2-source data 1/Uncropped blots for Figure1-figure supplement 2A/Figure 1- figure supplement 2A source data 6.tif]

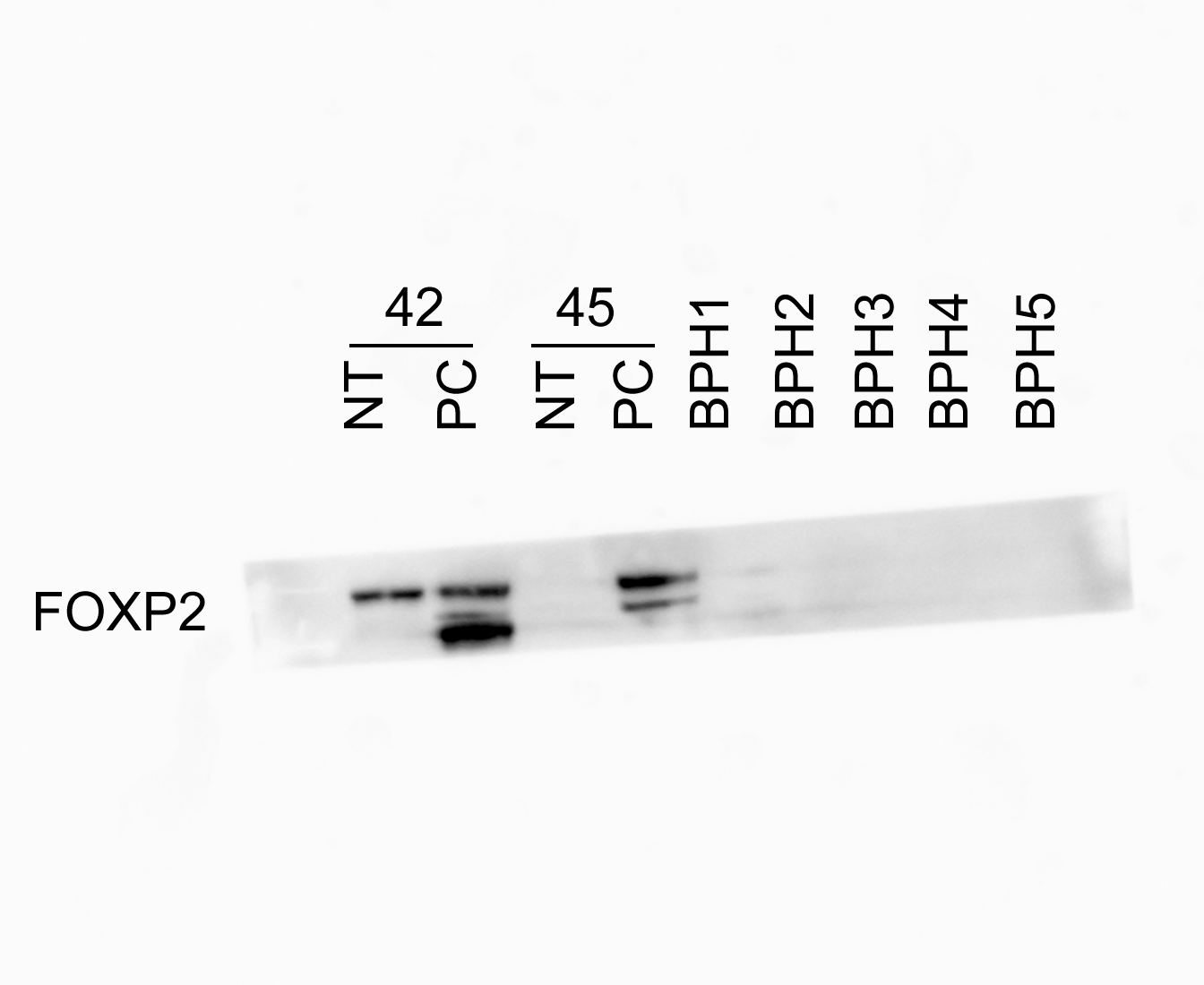

Supplement: Figure 1—figure supplement 2—source data 1. [file elife-81258-fig1-figsupp2-data1.zip › Figure 1-figure supplement 2-source data 1/Uncropped blots for Figure1-figure supplement 2A/Figure 1- figure supplement 2A source data 7.tif]

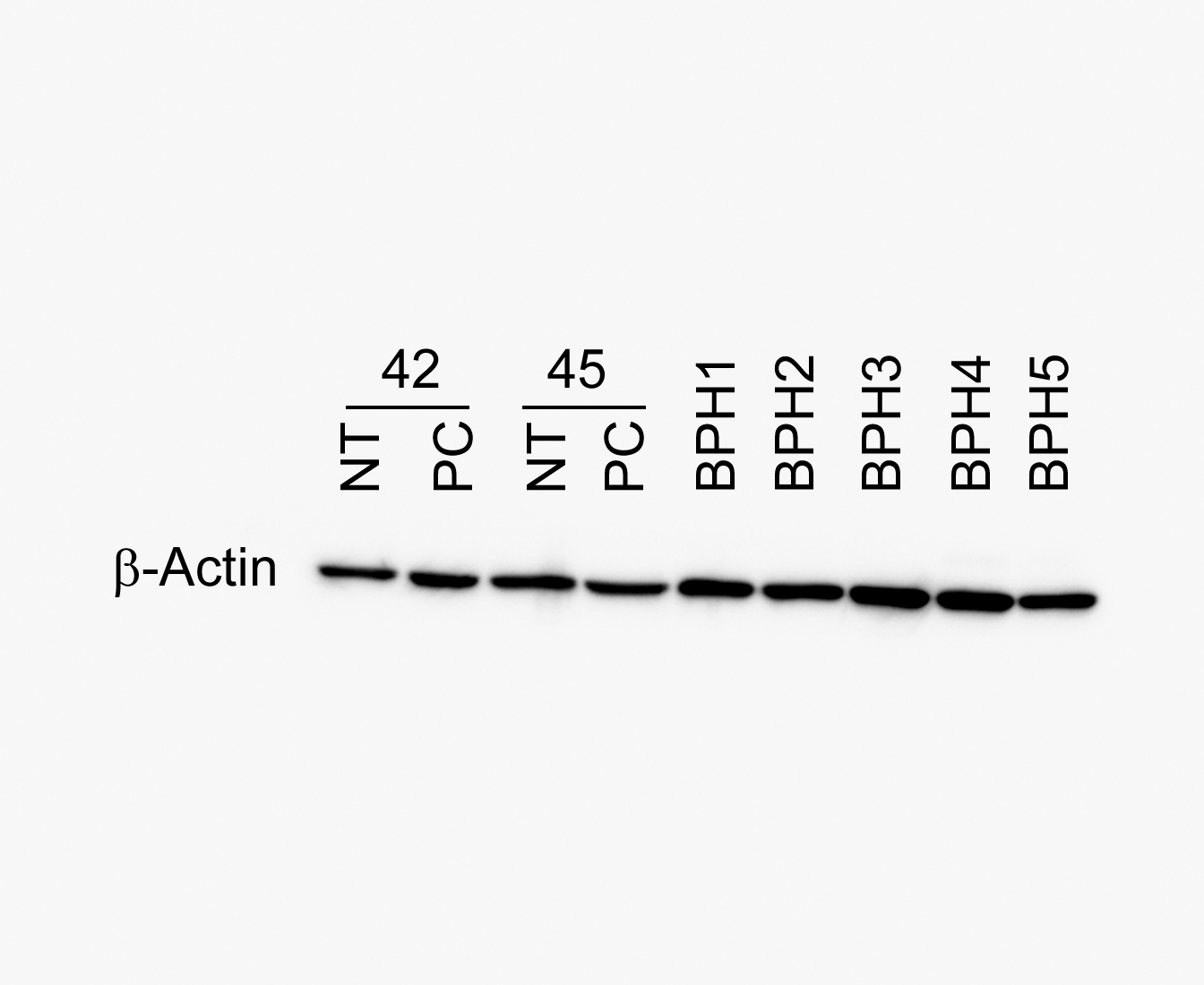

Supplement: Figure 1—figure supplement 2—source data 1. [file elife-81258-fig1-figsupp2-data1.zip › Figure 1-figure supplement 2-source data 1/Uncropped blots for Figure1-figure supplement 2A/Figure 1- figure supplement 2A source data 8.tif]

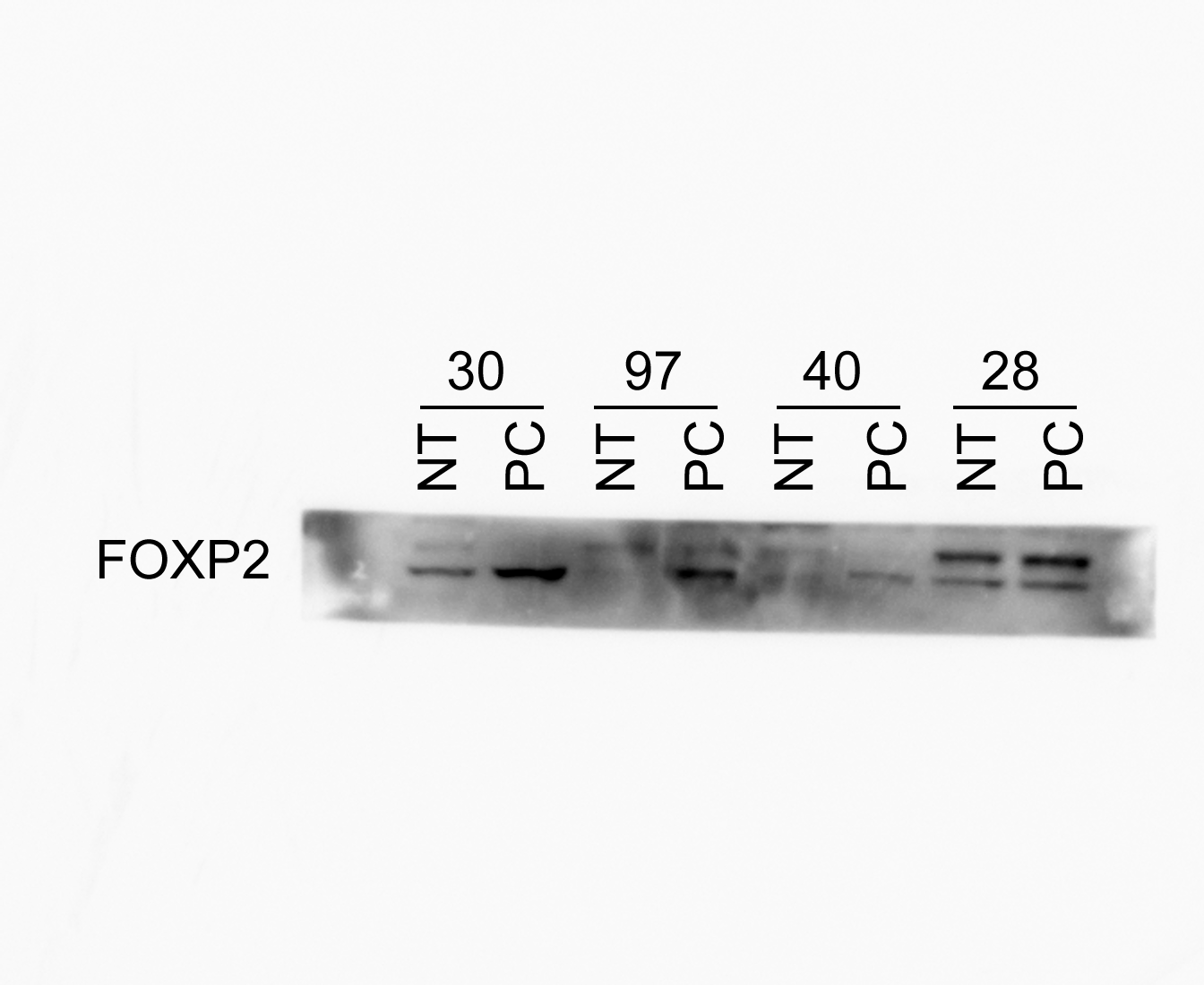

Supplement: Figure 1—figure supplement 2—source data 1. [file elife-81258-fig1-figsupp2-data1.zip › Figure 1-figure supplement 2-source data 1/Uncropped blots for Figure1-figure supplement 2A/Figure 1- figure supplement 2A source data 9.tif]

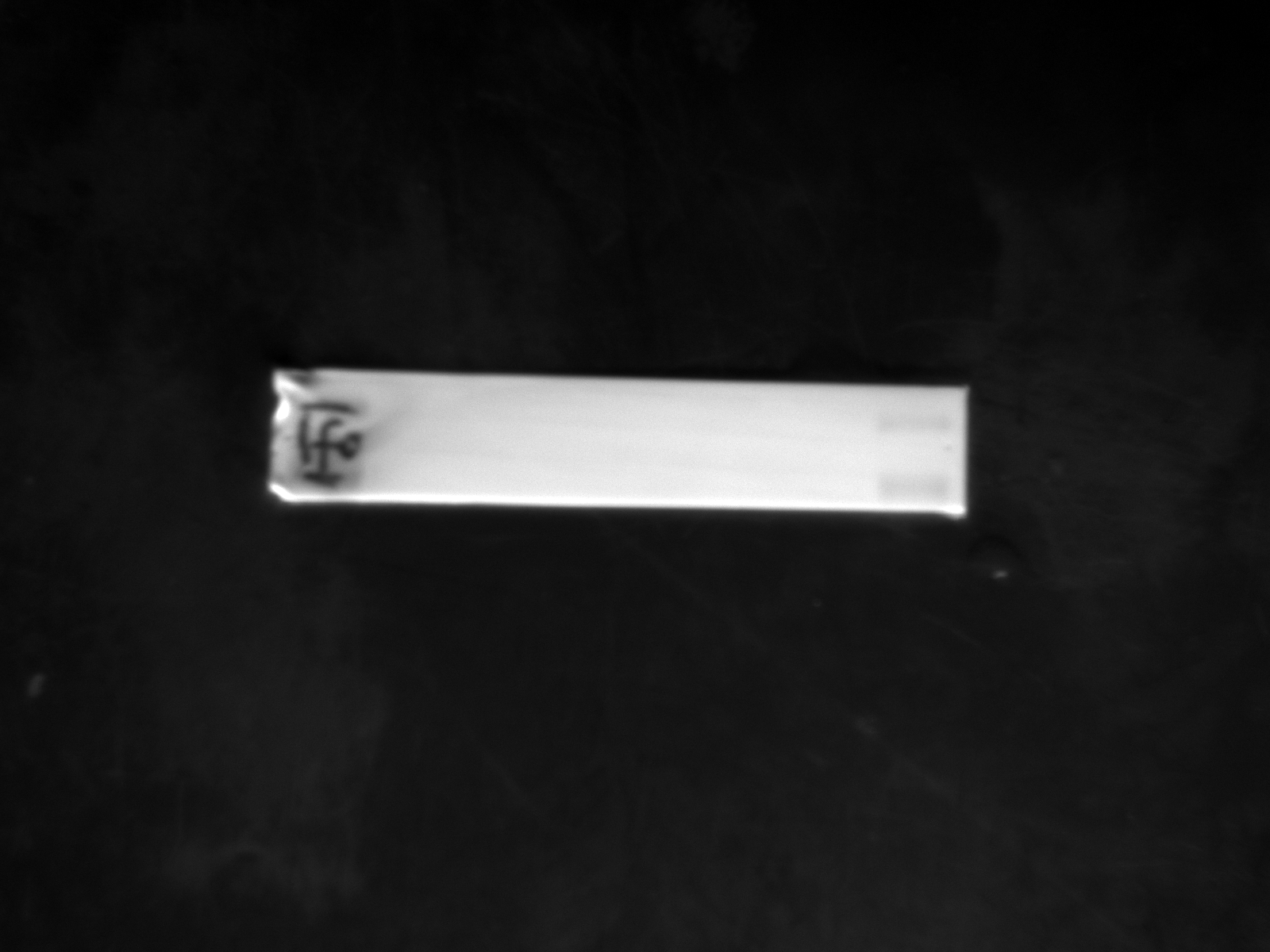

Supplement: Figure 1—figure supplement 3—source data 1. [file elife-81258-fig1-figsupp3-data1.zip › Figure 1-figure supplement 3-source data 1/Original files for Figure 1-figure supplement 3B/FOXP2-White-light image corresponding to WB image.BMP]

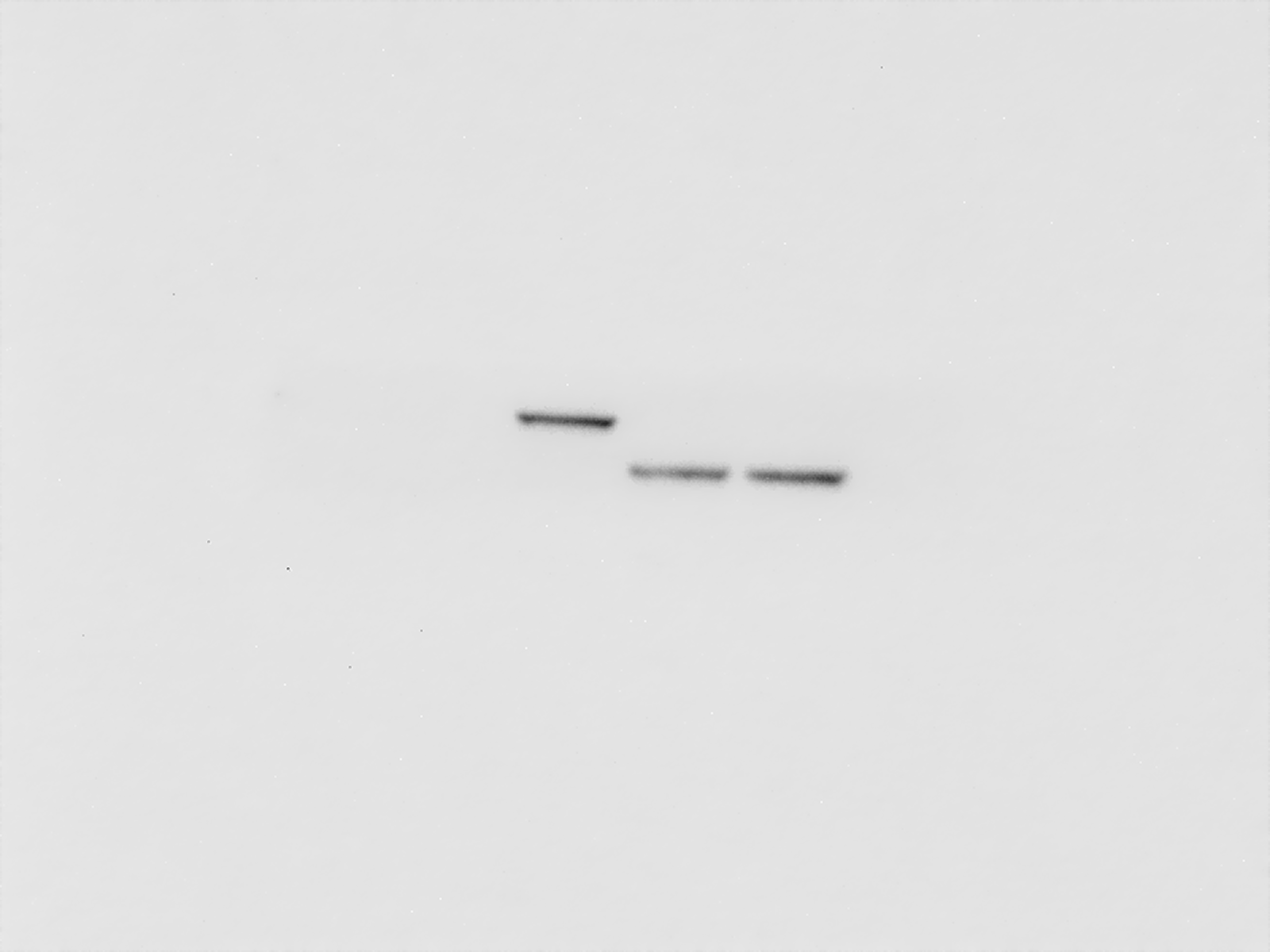

Supplement: Figure 1—figure supplement 3—source data 1. [file elife-81258-fig1-figsupp3-data1.zip › Figure 1-figure supplement 3-source data 1/Original files for Figure 1-figure supplement 3B/FOXP2.BMP]

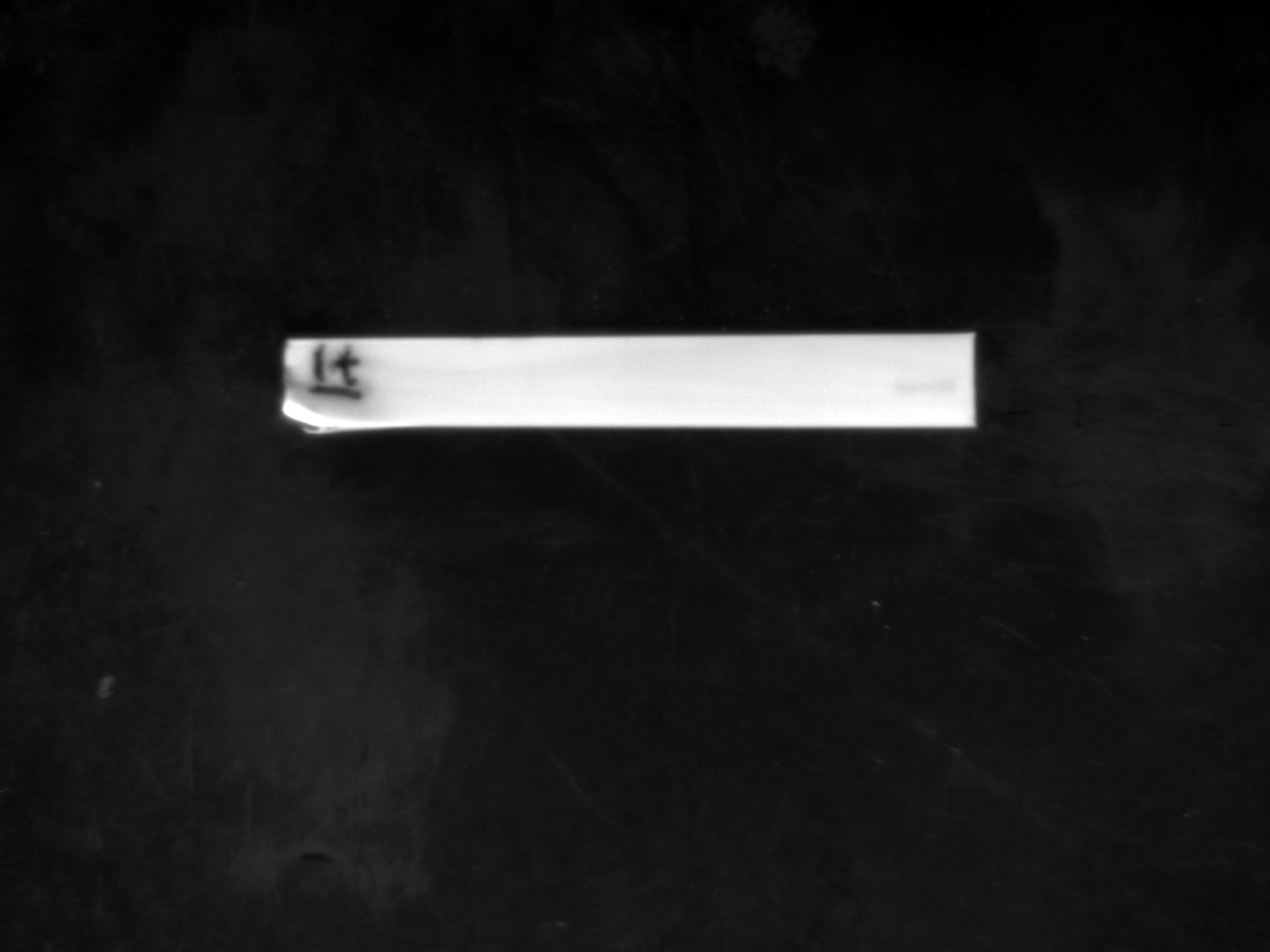

Supplement: Figure 1—figure supplement 3—source data 1. [file elife-81258-fig1-figsupp3-data1.zip › Figure 1-figure supplement 3-source data 1/Original files for Figure 1-figure supplement 3B/Tubulin-White-light image corresponding to WB image.BMP]

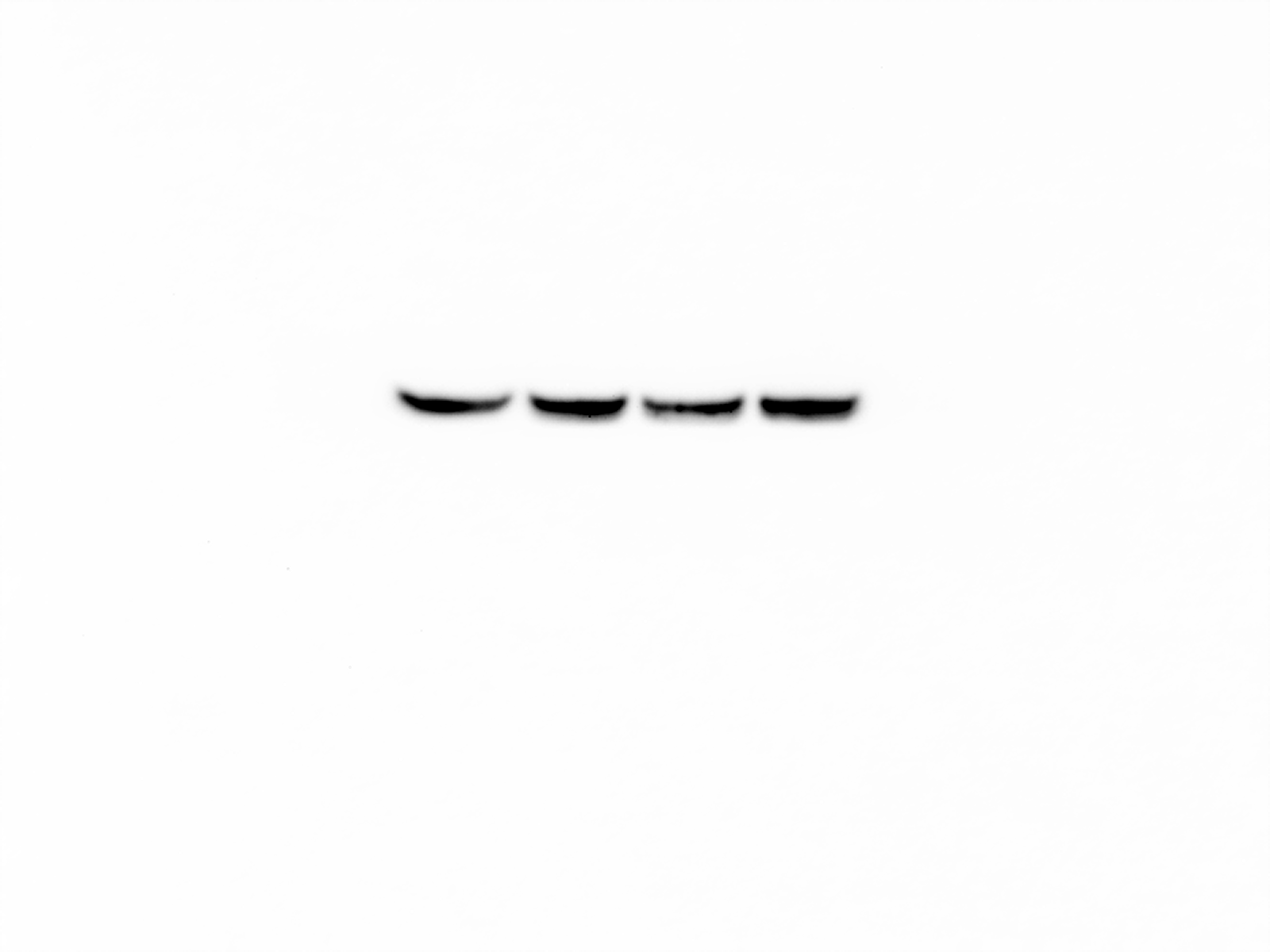

Supplement: Figure 1—figure supplement 3—source data 1. [file elife-81258-fig1-figsupp3-data1.zip › Figure 1-figure supplement 3-source data 1/Original files for Figure 1-figure supplement 3B/Tubulin.BMP]

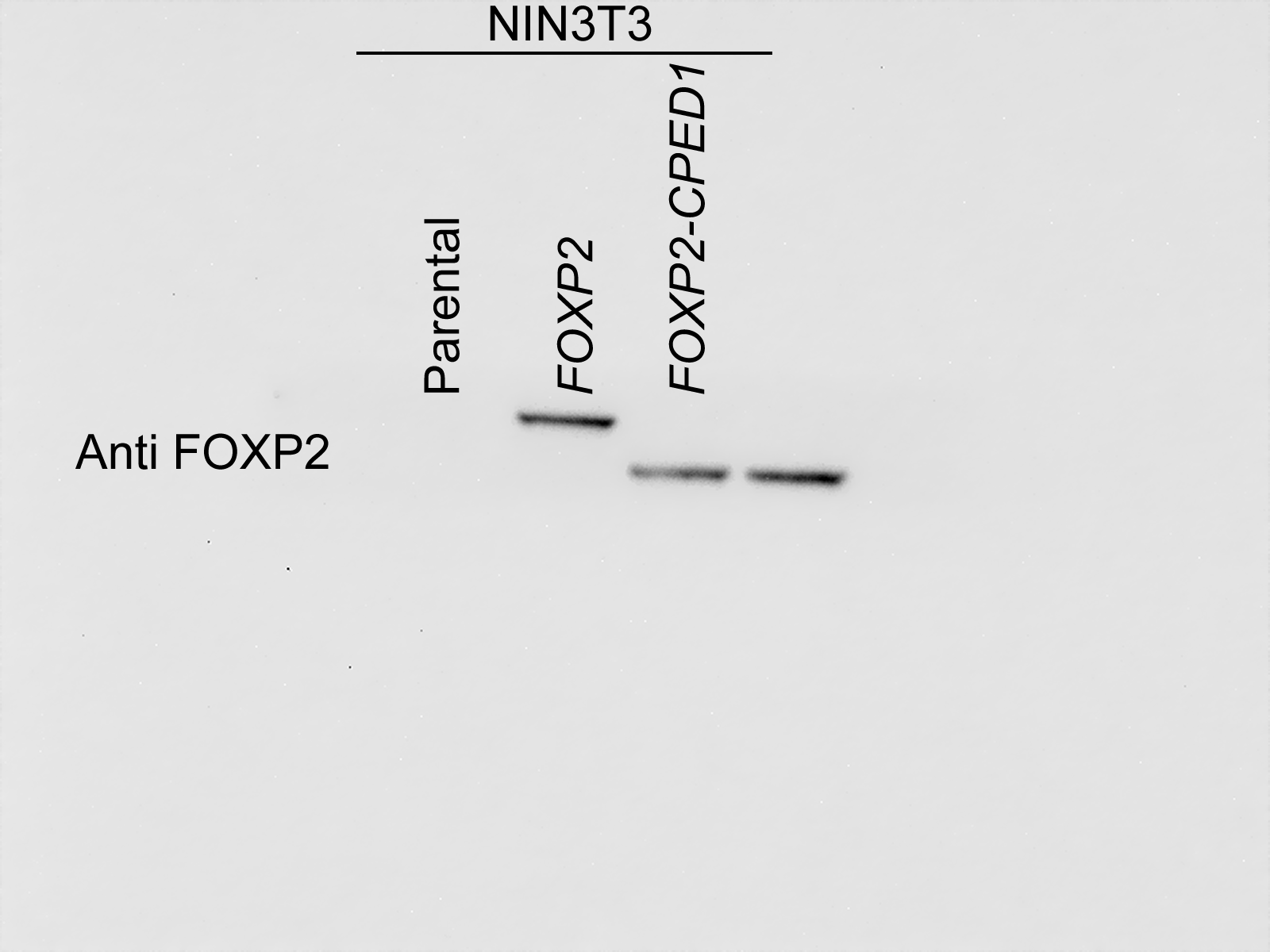

Supplement: Figure 1—figure supplement 3—source data 1. [file elife-81258-fig1-figsupp3-data1.zip › Figure 1-figure supplement 3-source data 1/Uncropped blots for Figure 1-figure supplement 3B/Figure 1- figure supplement 3B source data 1.tif]

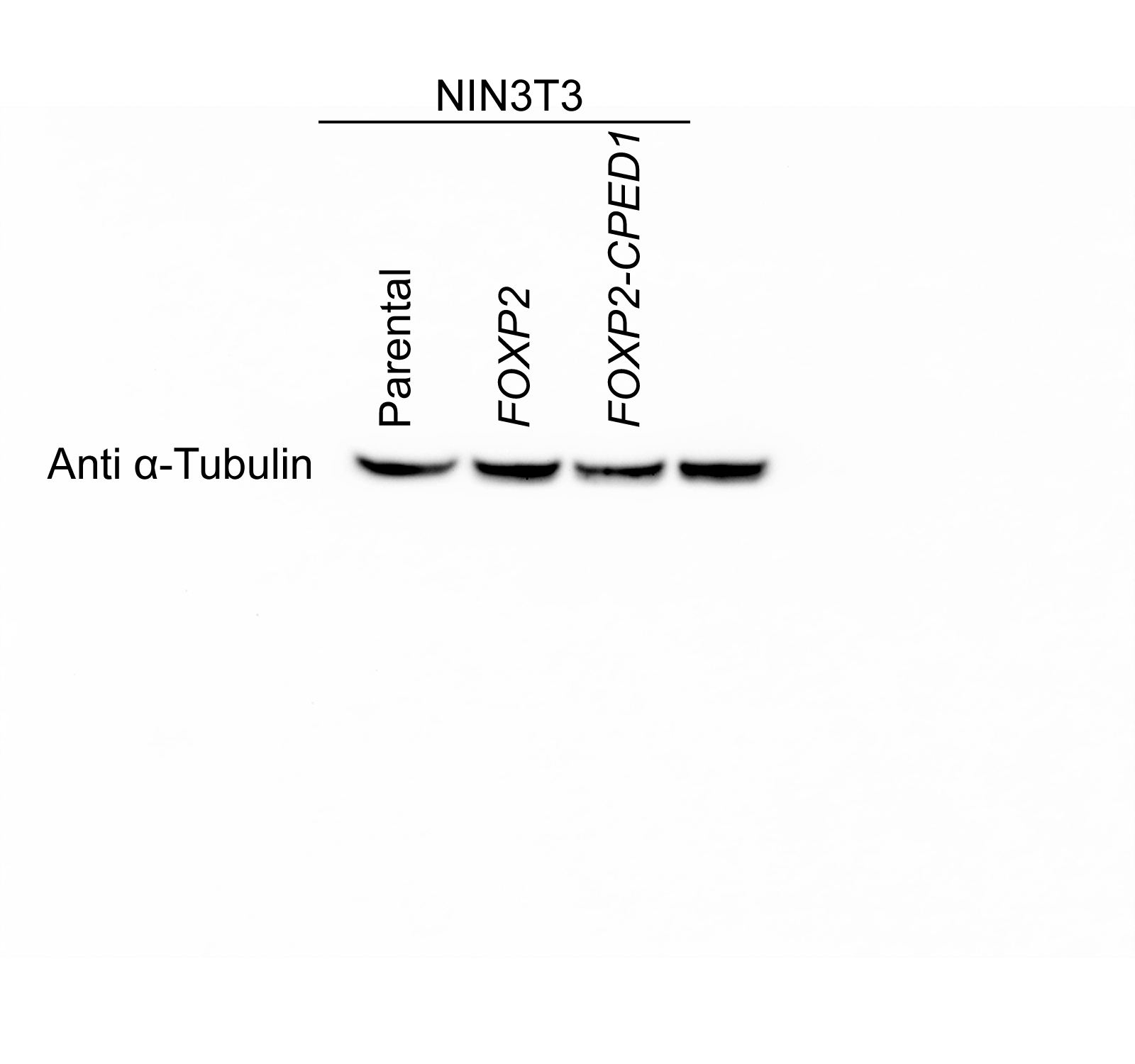

Supplement: Figure 1—figure supplement 3—source data 1. [file elife-81258-fig1-figsupp3-data1.zip › Figure 1-figure supplement 3-source data 1/Uncropped blots for Figure 1-figure supplement 3B/Figure 1- figure supplement 3B source data 2.tif]

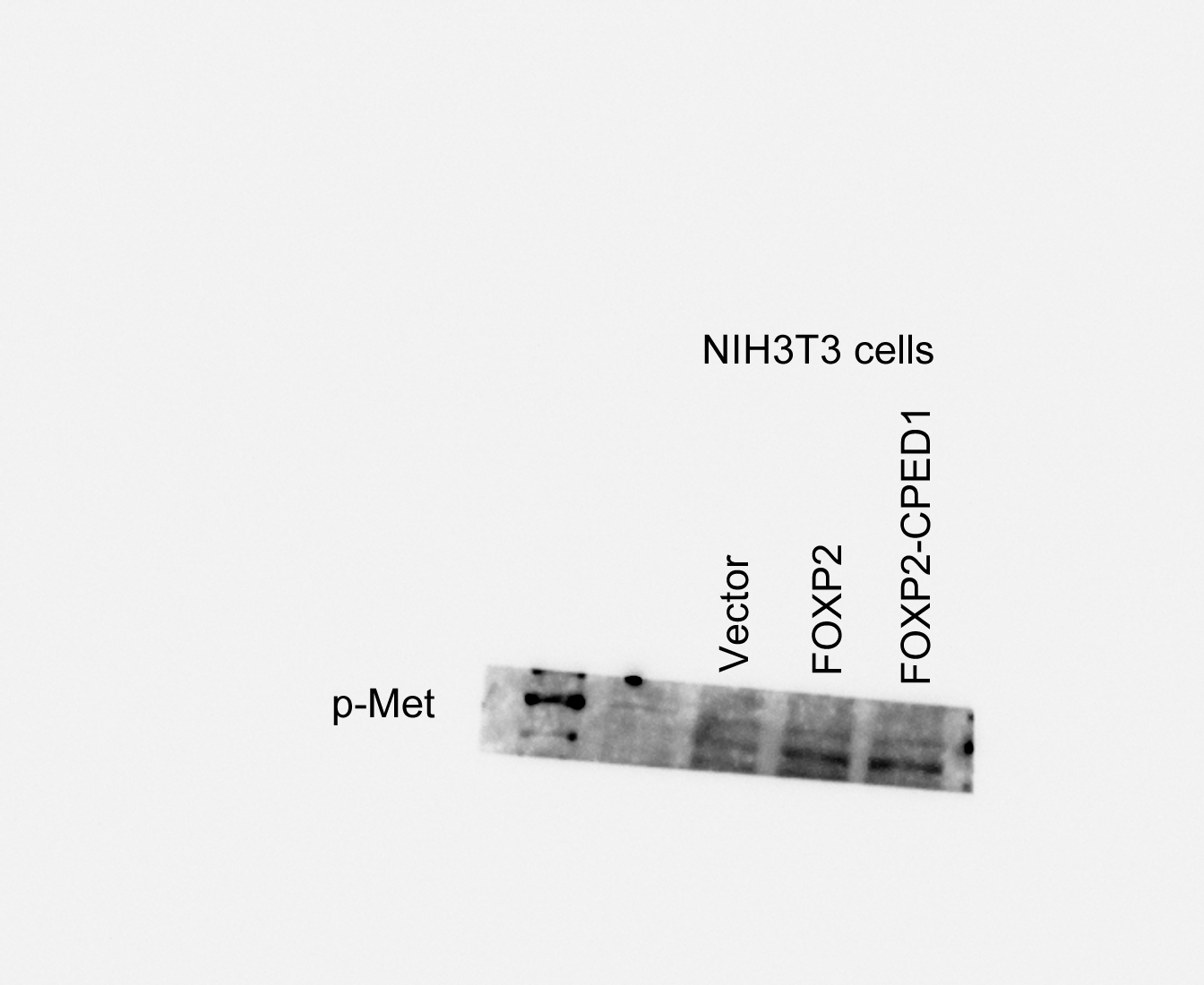

Supplement: Figure 3—source data 1. [file elife-81258-fig3-data1.zip › Figure 3-source data 1/Uncropped blots for Figure 3A in Main text/Figure 3A-source data 1.tif]

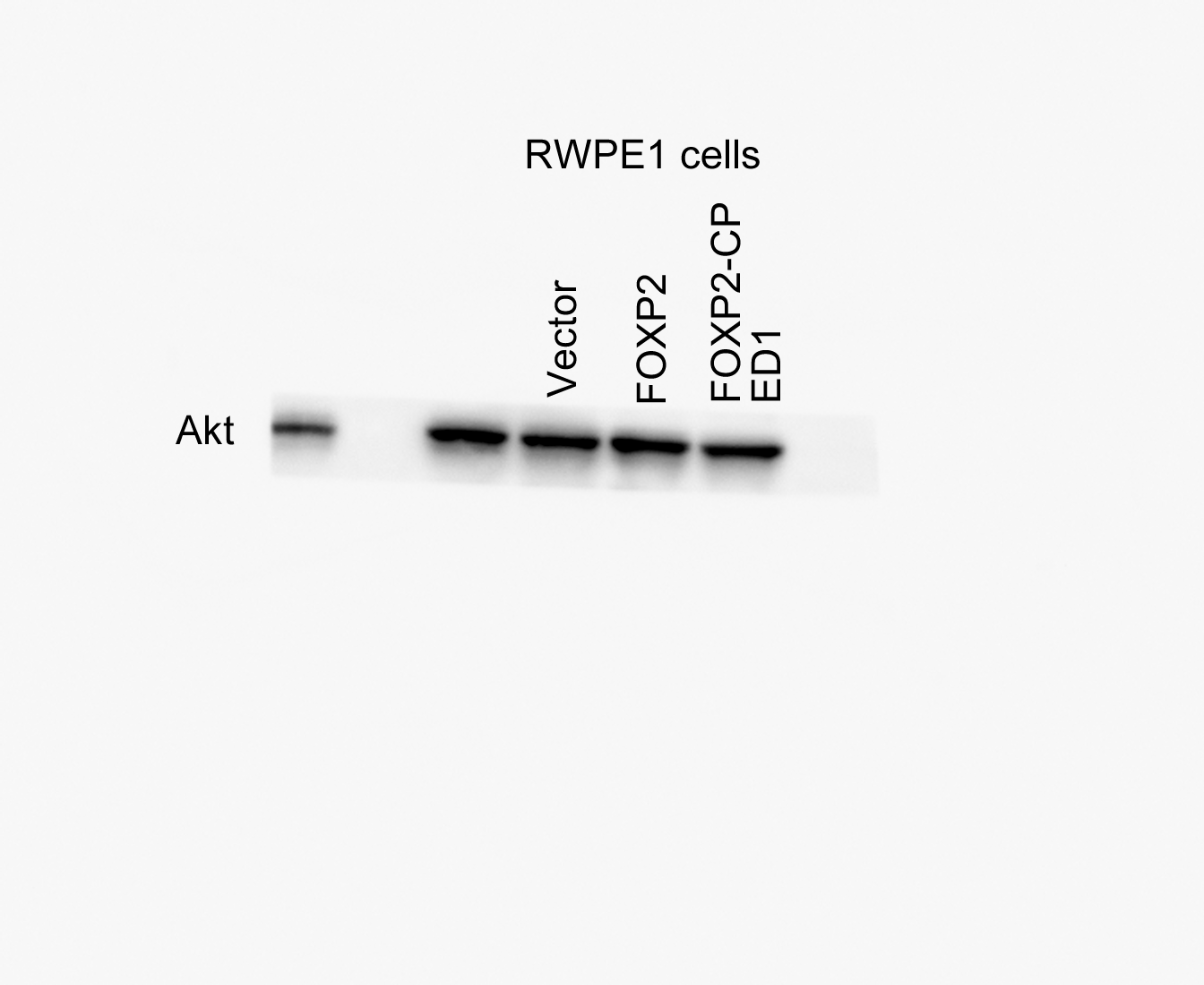

Supplement: Figure 3—source data 1. [file elife-81258-fig3-data1.zip › Figure 3-source data 1/Uncropped blots for Figure 3A in Main text/Figure 3A-source data 10.tif]

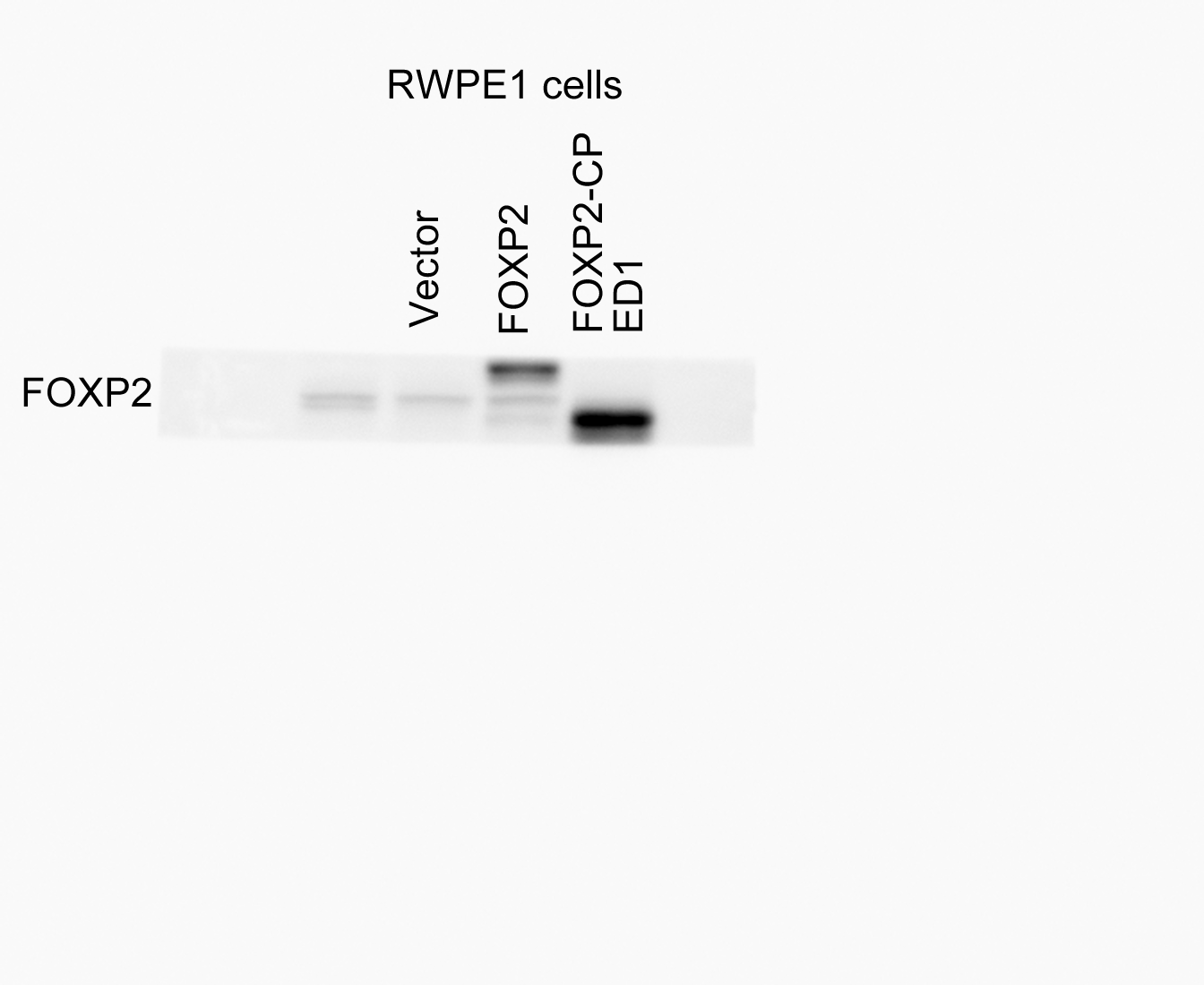

Supplement: Figure 3—source data 1. [file elife-81258-fig3-data1.zip › Figure 3-source data 1/Uncropped blots for Figure 3A in Main text/Figure 3A-source data 11.tif]

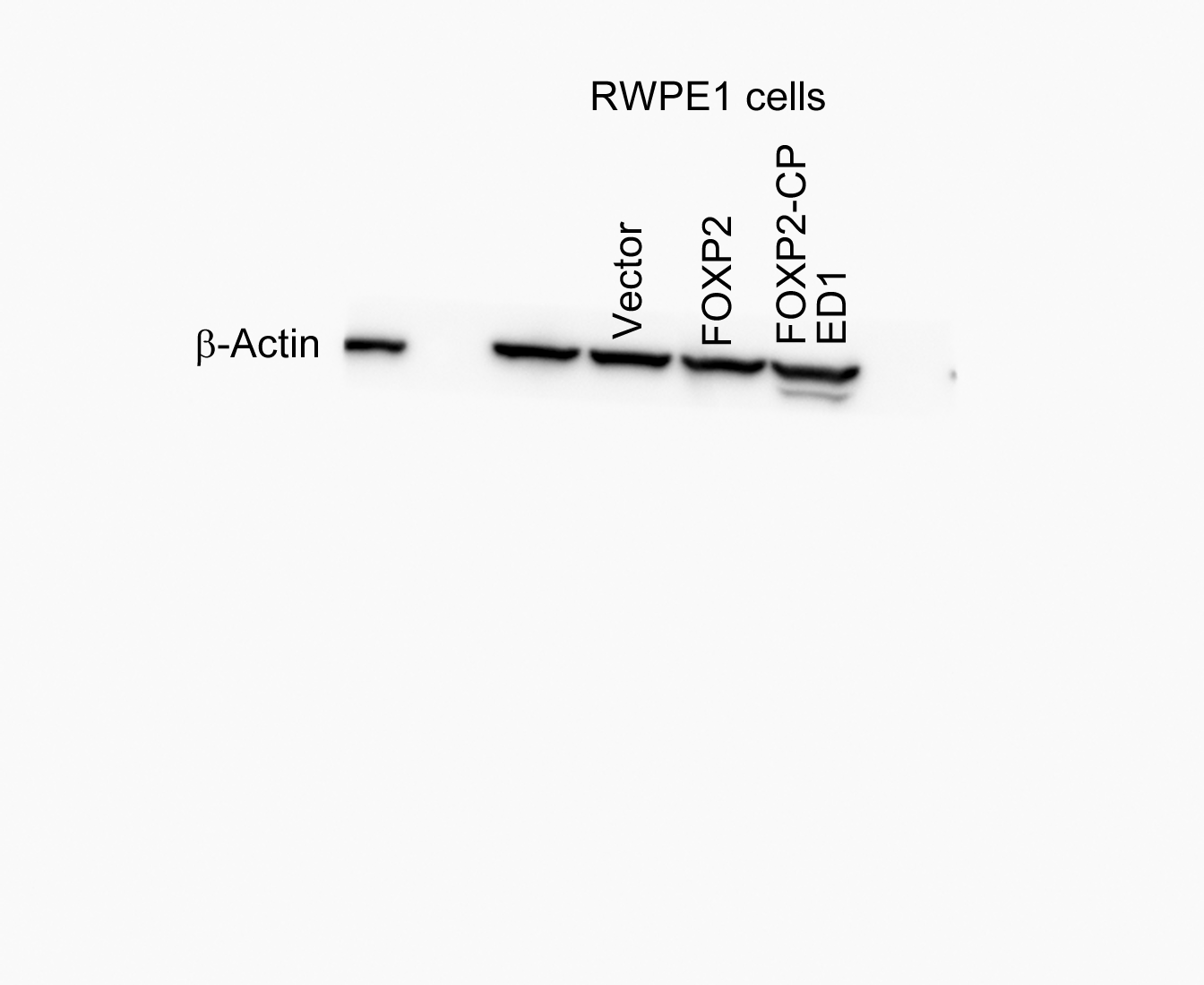

Supplement: Figure 3—source data 1. [file elife-81258-fig3-data1.zip › Figure 3-source data 1/Uncropped blots for Figure 3A in Main text/Figure 3A-source data 12.tif]

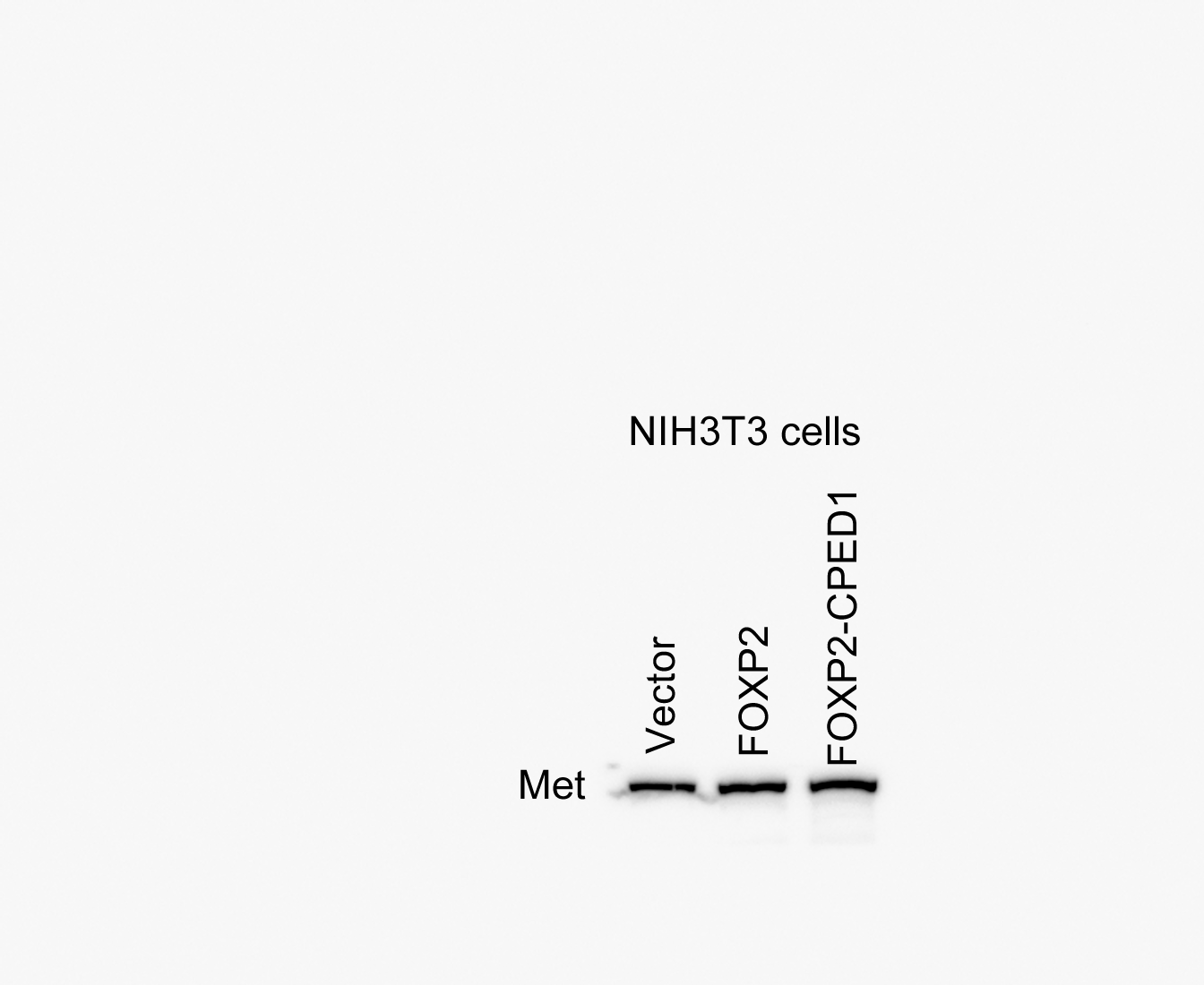

Supplement: Figure 3—source data 1. [file elife-81258-fig3-data1.zip › Figure 3-source data 1/Uncropped blots for Figure 3A in Main text/Figure 3A-source data 2.tif]

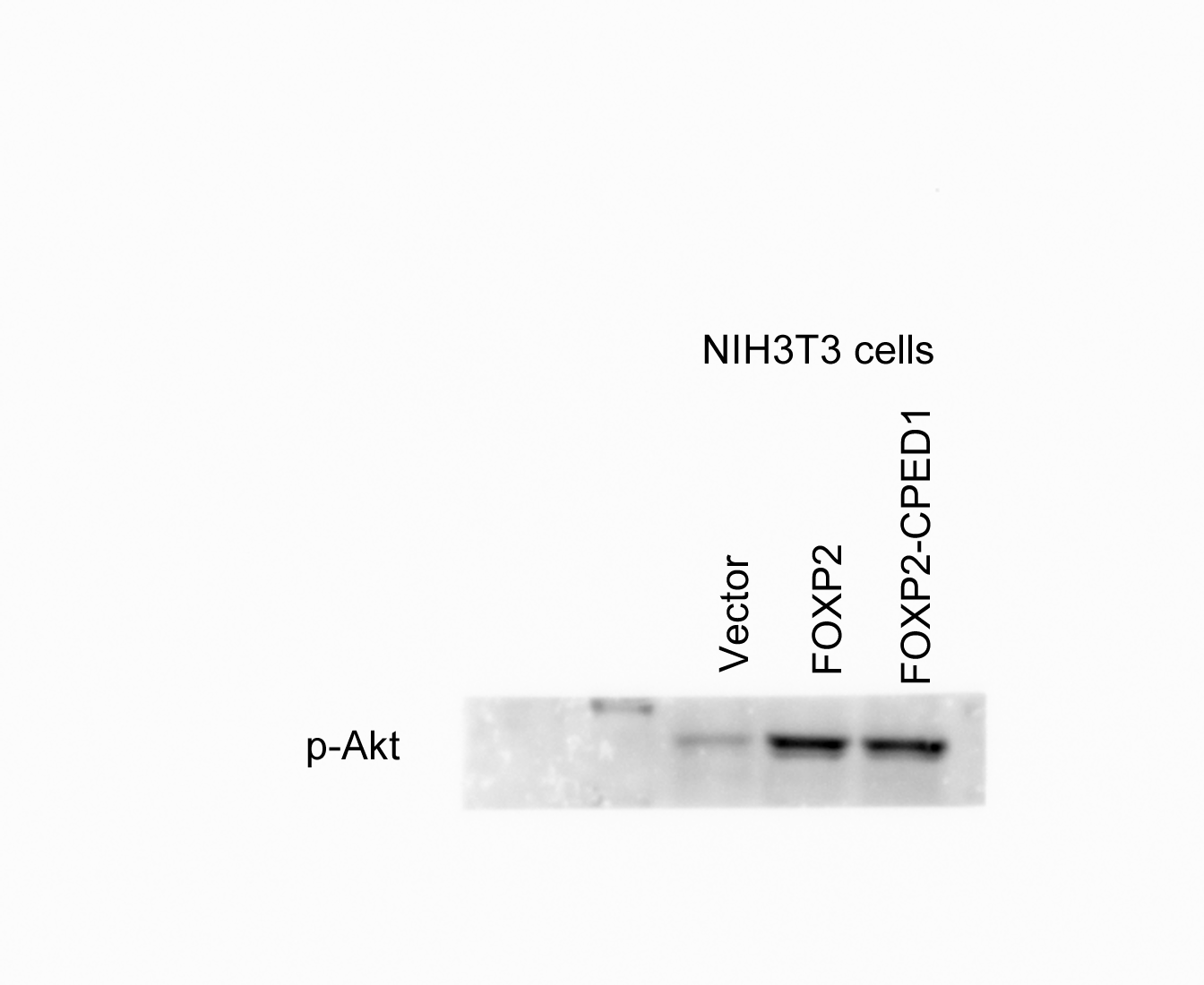

Supplement: Figure 3—source data 1. [file elife-81258-fig3-data1.zip › Figure 3-source data 1/Uncropped blots for Figure 3A in Main text/Figure 3A-source data 3.tif]

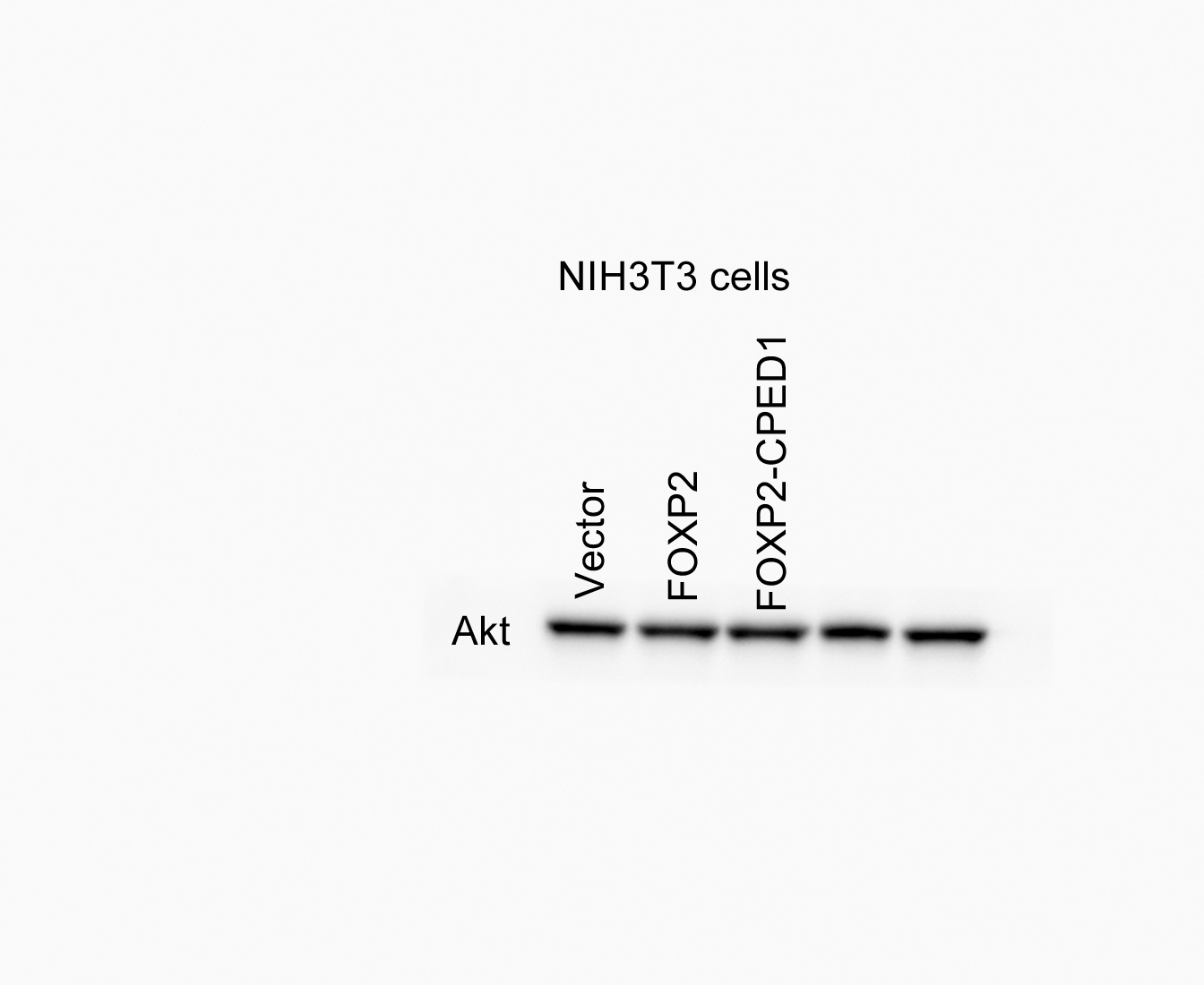

Supplement: Figure 3—source data 1. [file elife-81258-fig3-data1.zip › Figure 3-source data 1/Uncropped blots for Figure 3A in Main text/Figure 3A-source data 4.tif]

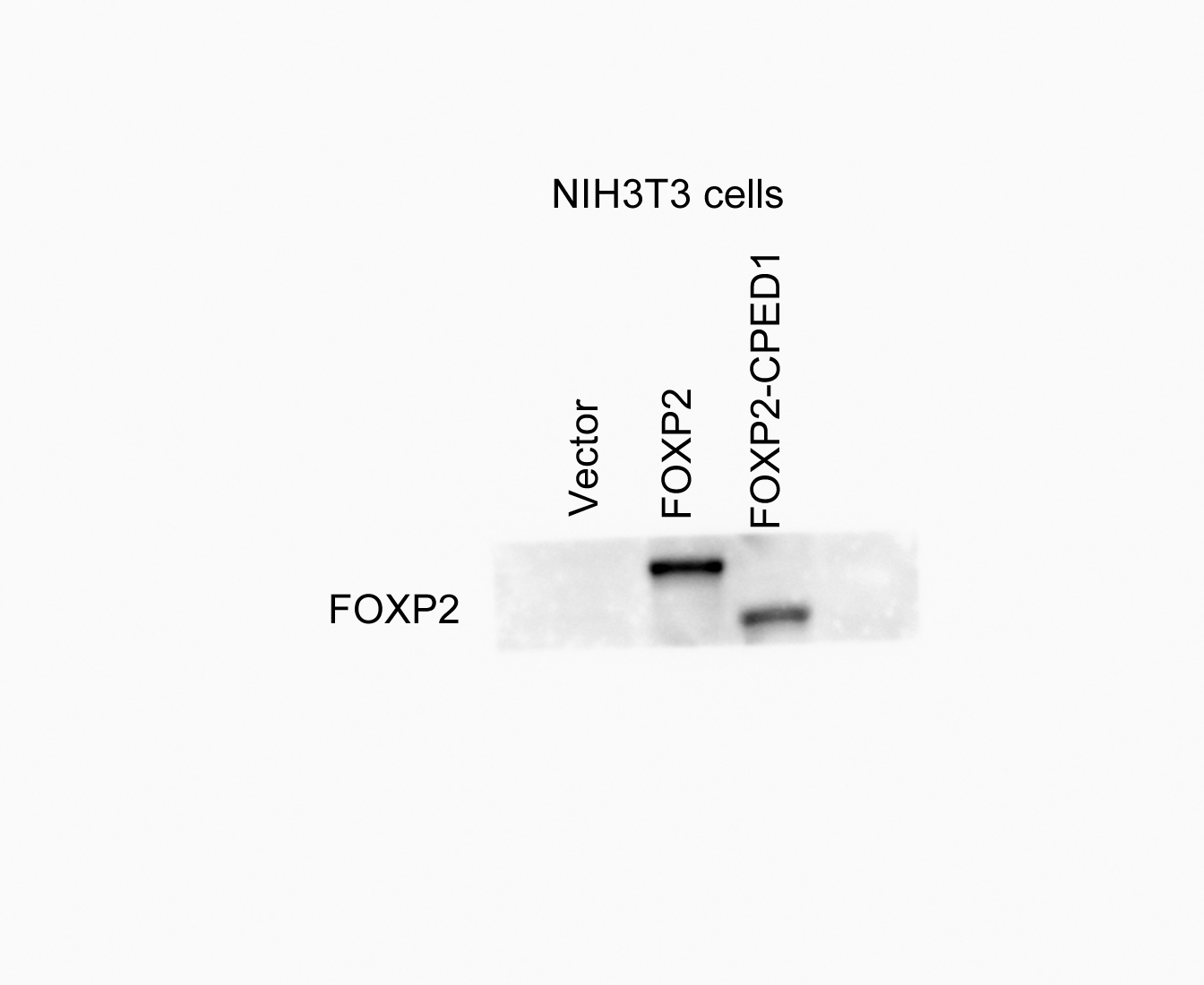

Supplement: Figure 3—source data 1. [file elife-81258-fig3-data1.zip › Figure 3-source data 1/Uncropped blots for Figure 3A in Main text/Figure 3A-source data 5.tif]

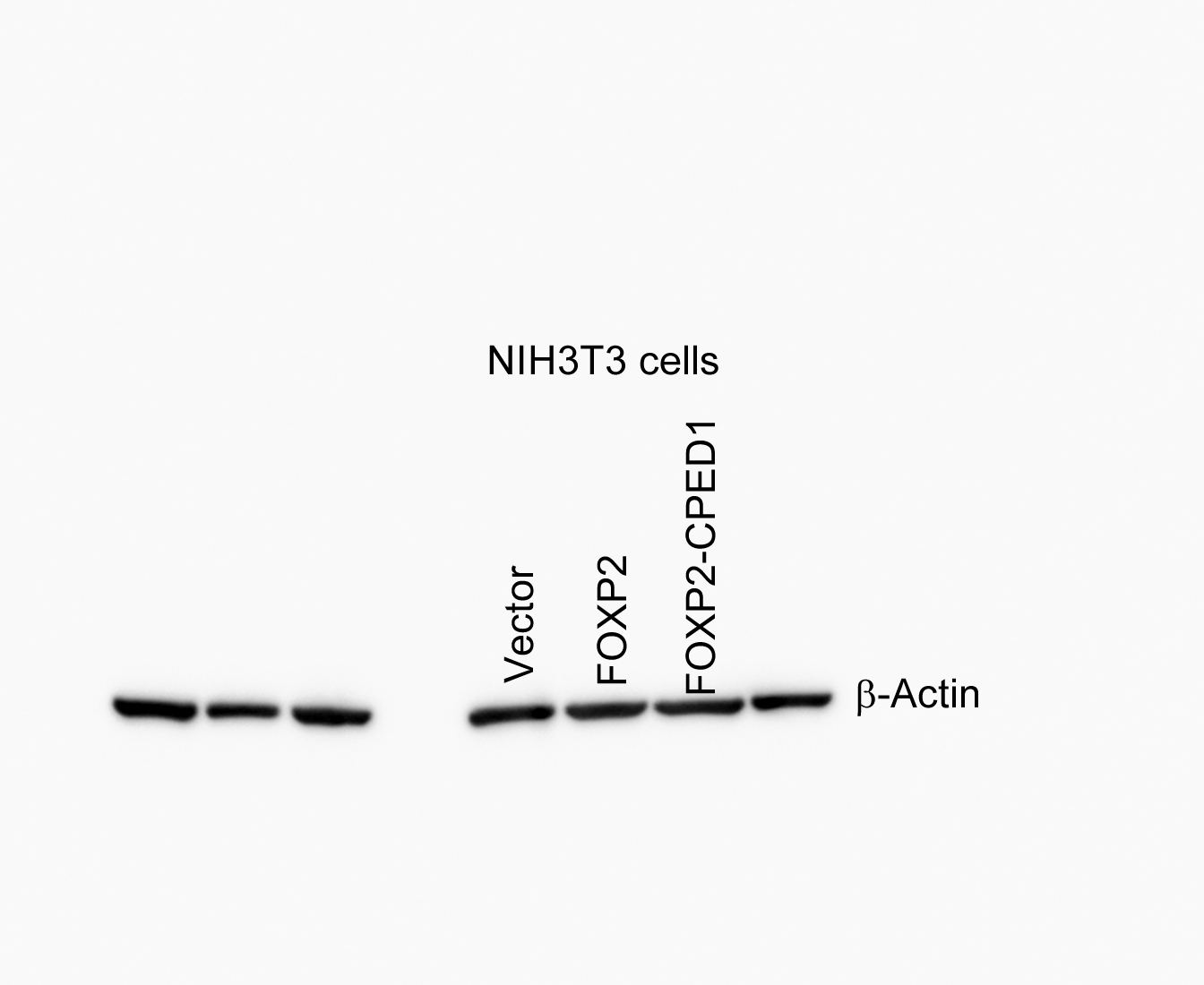

Supplement: Figure 3—source data 1. [file elife-81258-fig3-data1.zip › Figure 3-source data 1/Uncropped blots for Figure 3A in Main text/Figure 3A-source data 6.tif]

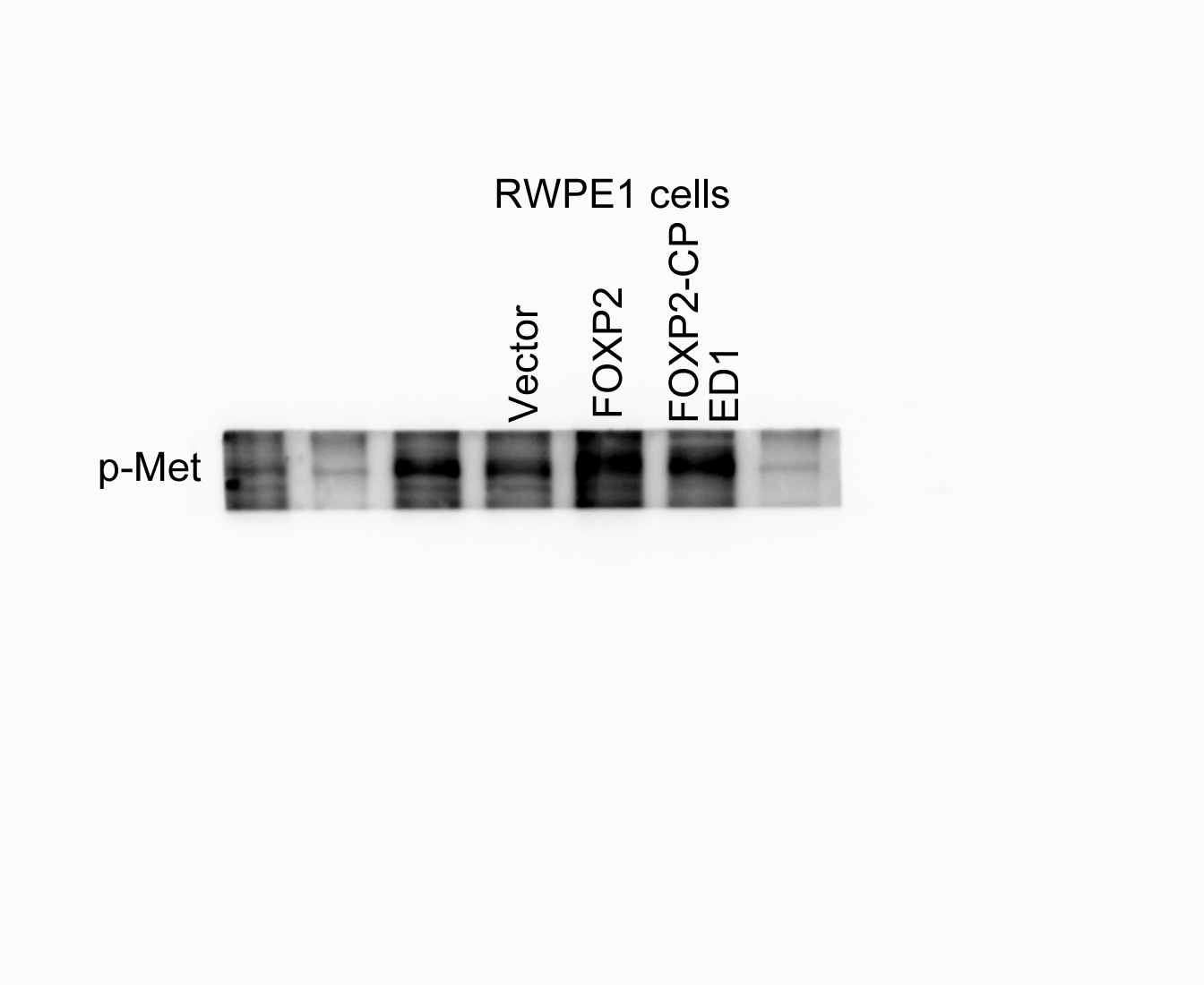

Supplement: Figure 3—source data 1. [file elife-81258-fig3-data1.zip › Figure 3-source data 1/Uncropped blots for Figure 3A in Main text/Figure 3A-source data 7.tif]

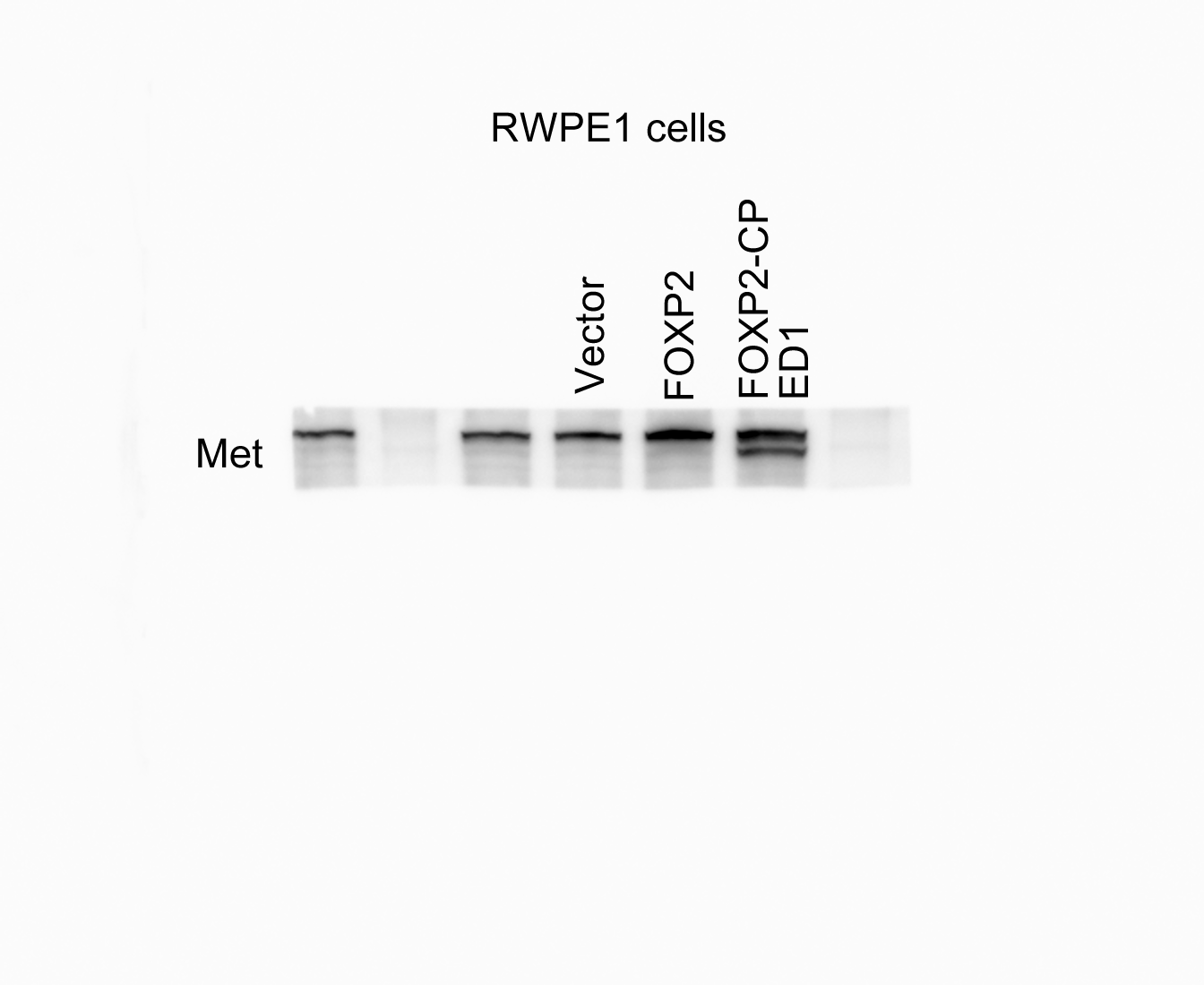

Supplement: Figure 3—source data 1. [file elife-81258-fig3-data1.zip › Figure 3-source data 1/Uncropped blots for Figure 3A in Main text/Figure 3A-source data 8.tif]

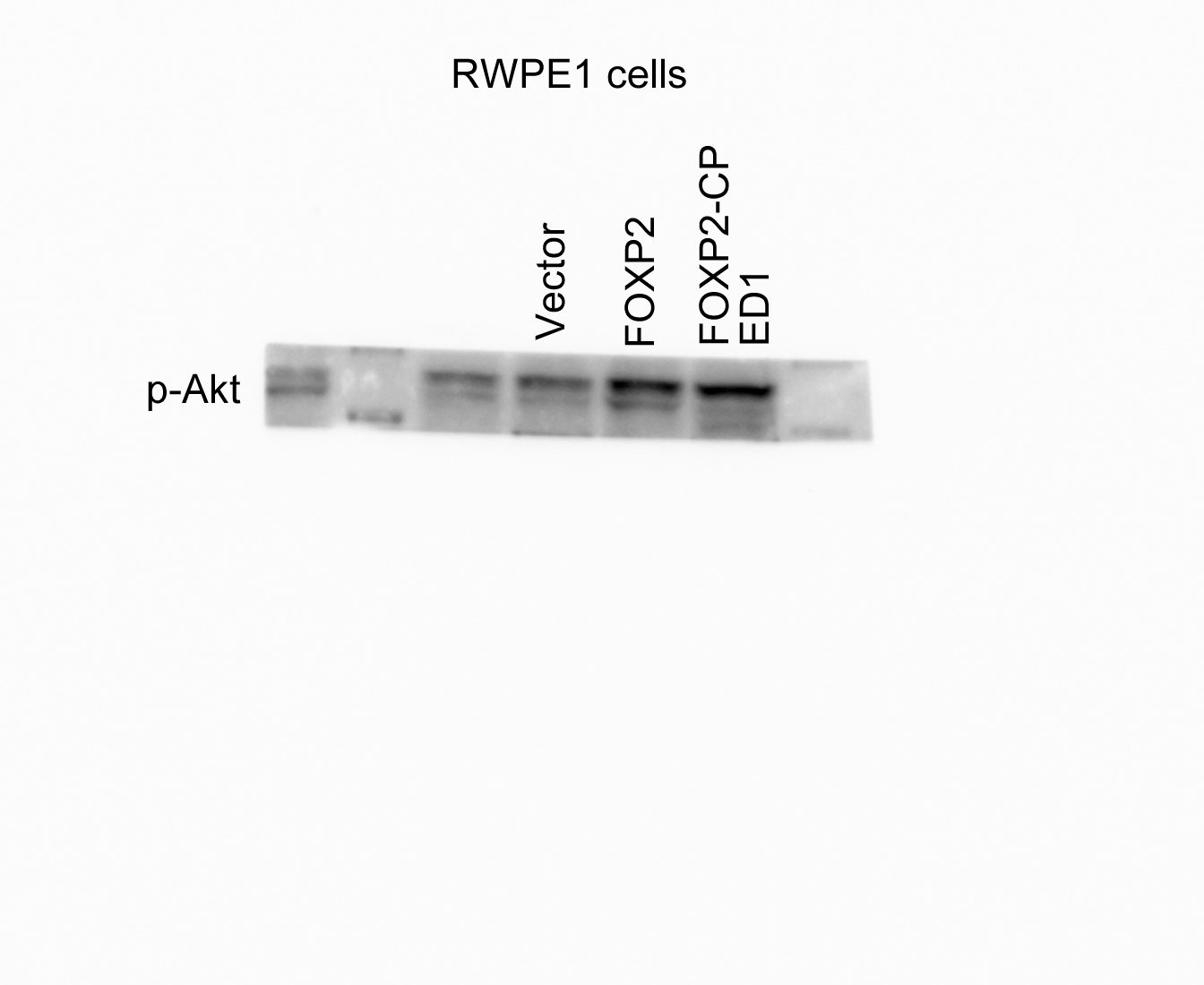

Supplement: Figure 3—source data 1. [file elife-81258-fig3-data1.zip › Figure 3-source data 1/Uncropped blots for Figure 3A in Main text/Figure 3A-source data 9.tif]

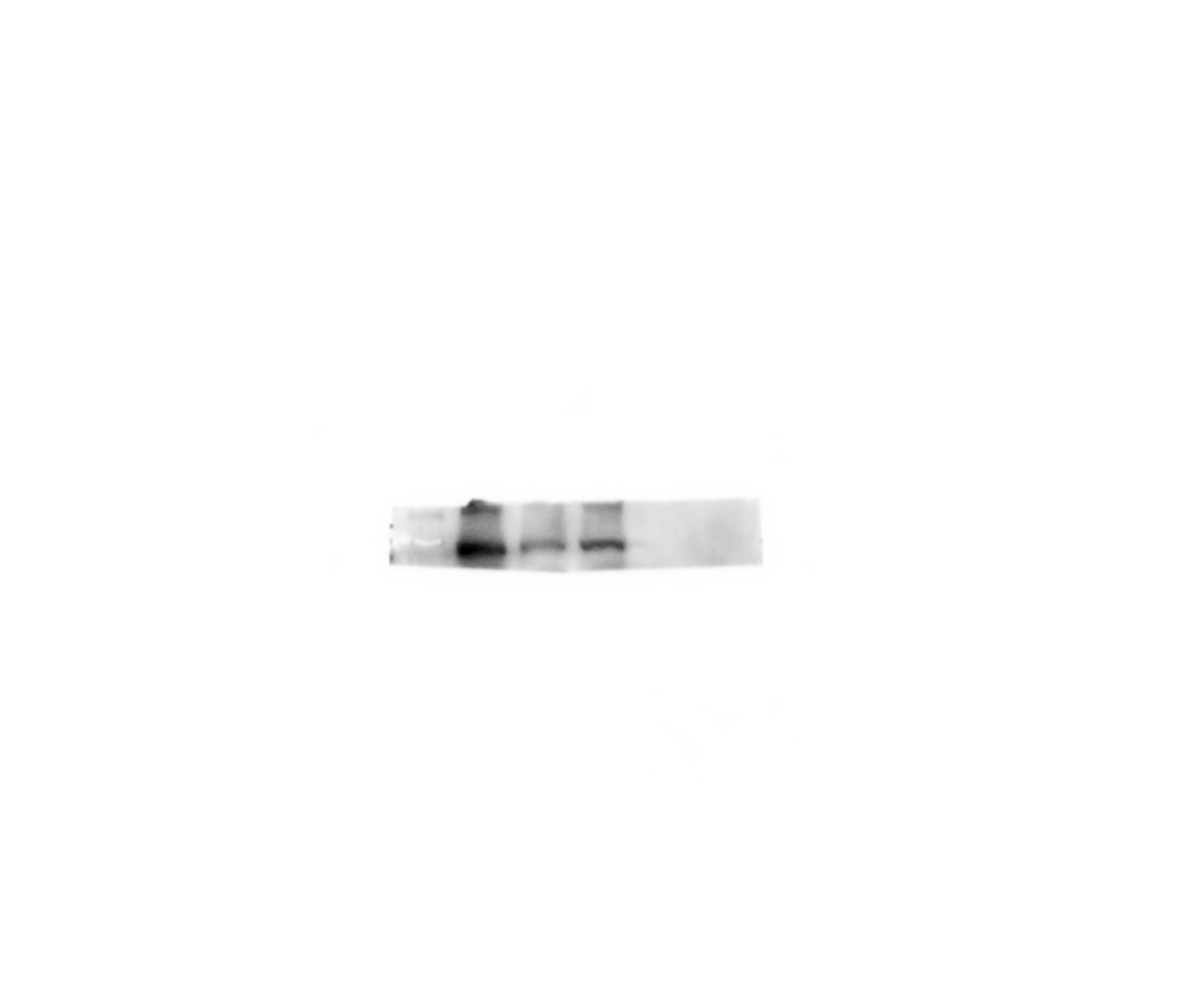

Supplement: Figure 3—source data 2. [file elife-81258-fig3-data2.zip › Figure 3-source data 2/Original files for Figure 3B PC3 cells/Original files for Figure 3B PC3 cells Repeat/8-P-MET.Tif]

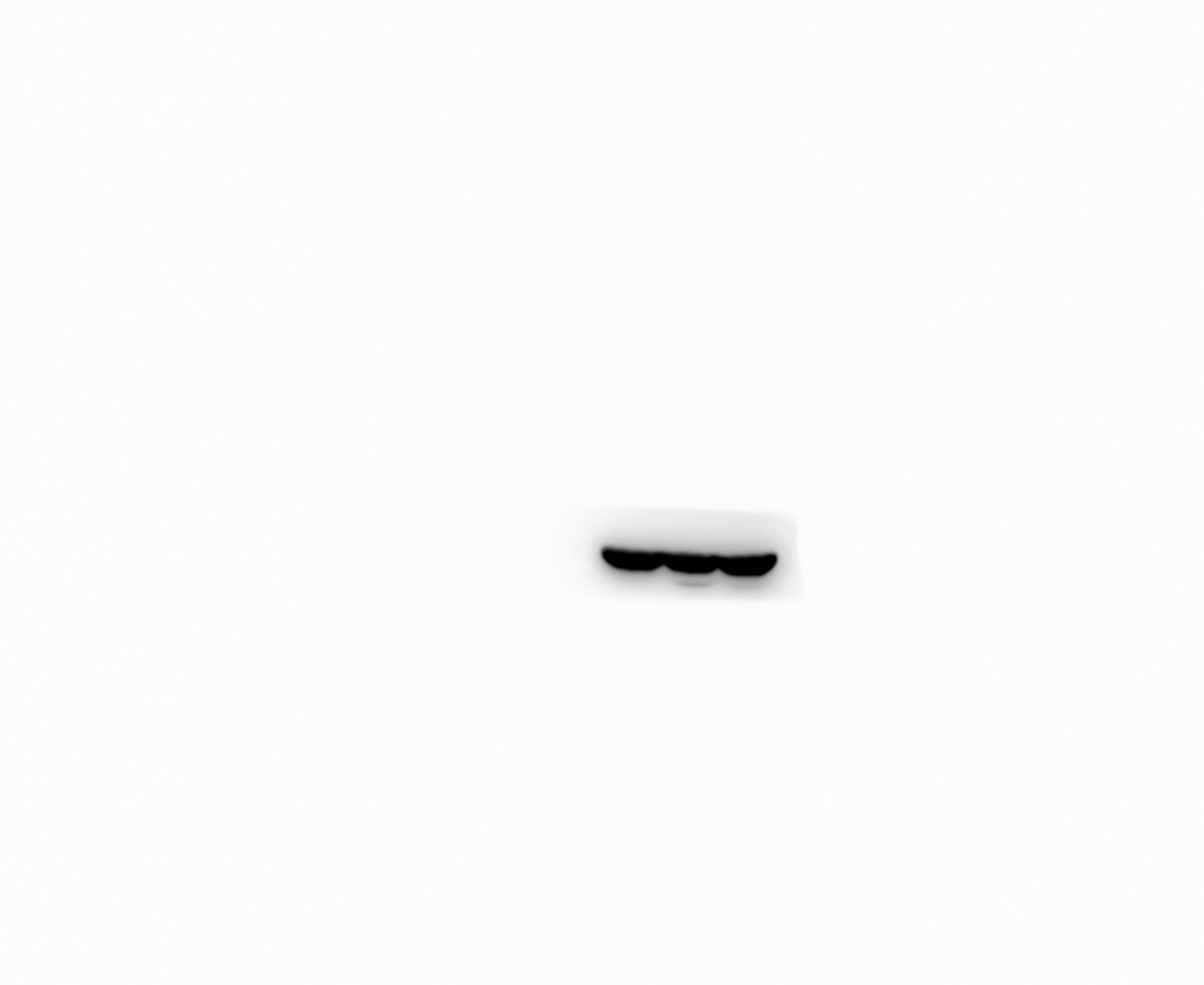

Supplement: Figure 3—source data 2. [file elife-81258-fig3-data2.zip › Figure 3-source data 2/Original files for Figure 3B PC3 cells/Original files for Figure 3B PC3 in Main text/ACTIN-2.tif]

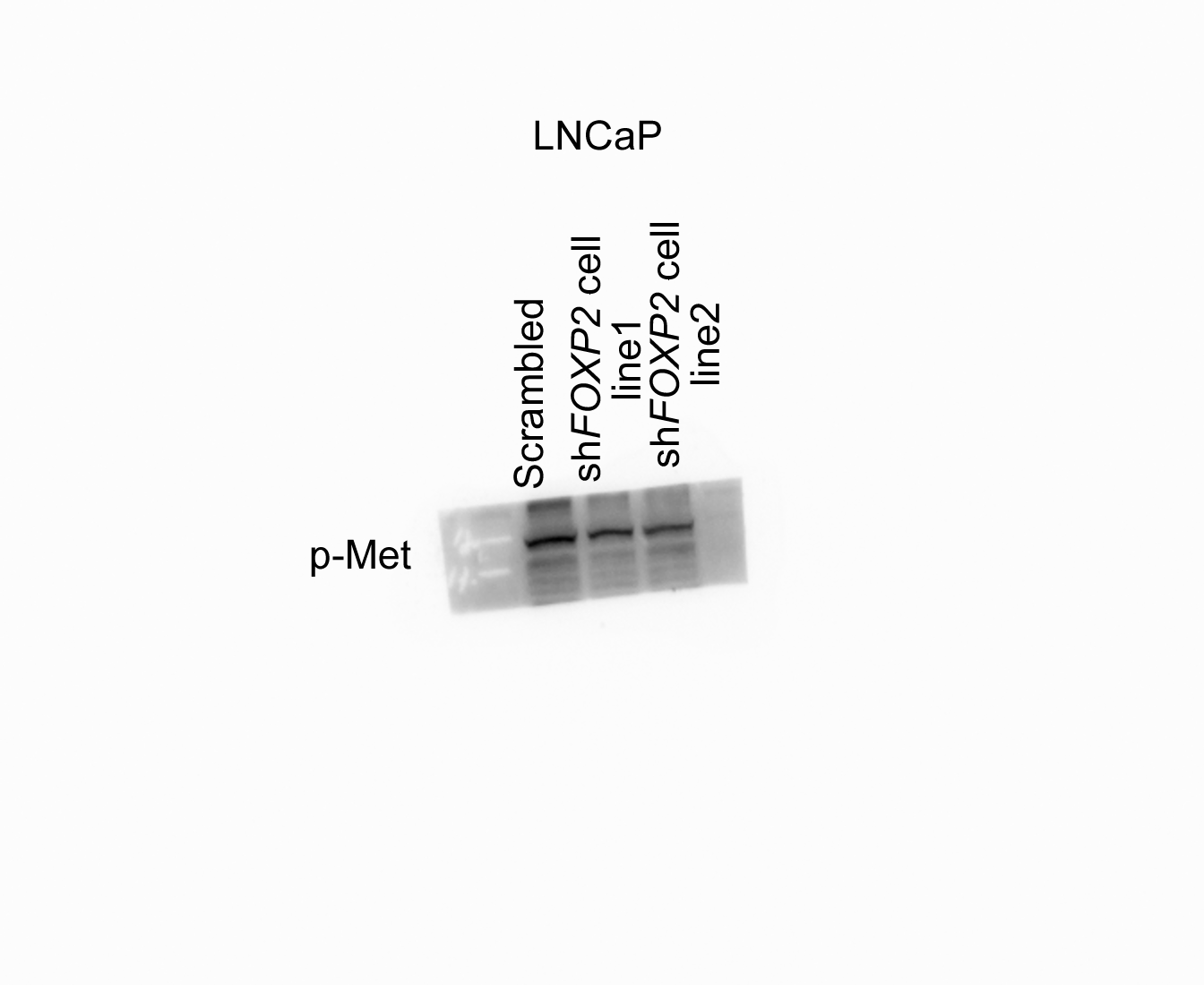

Supplement: Figure 3—source data 2. [file elife-81258-fig3-data2.zip › Figure 3-source data 2/Uncropped blots for Figure 3B in Main text/Figure 3B-source data 1.tif]

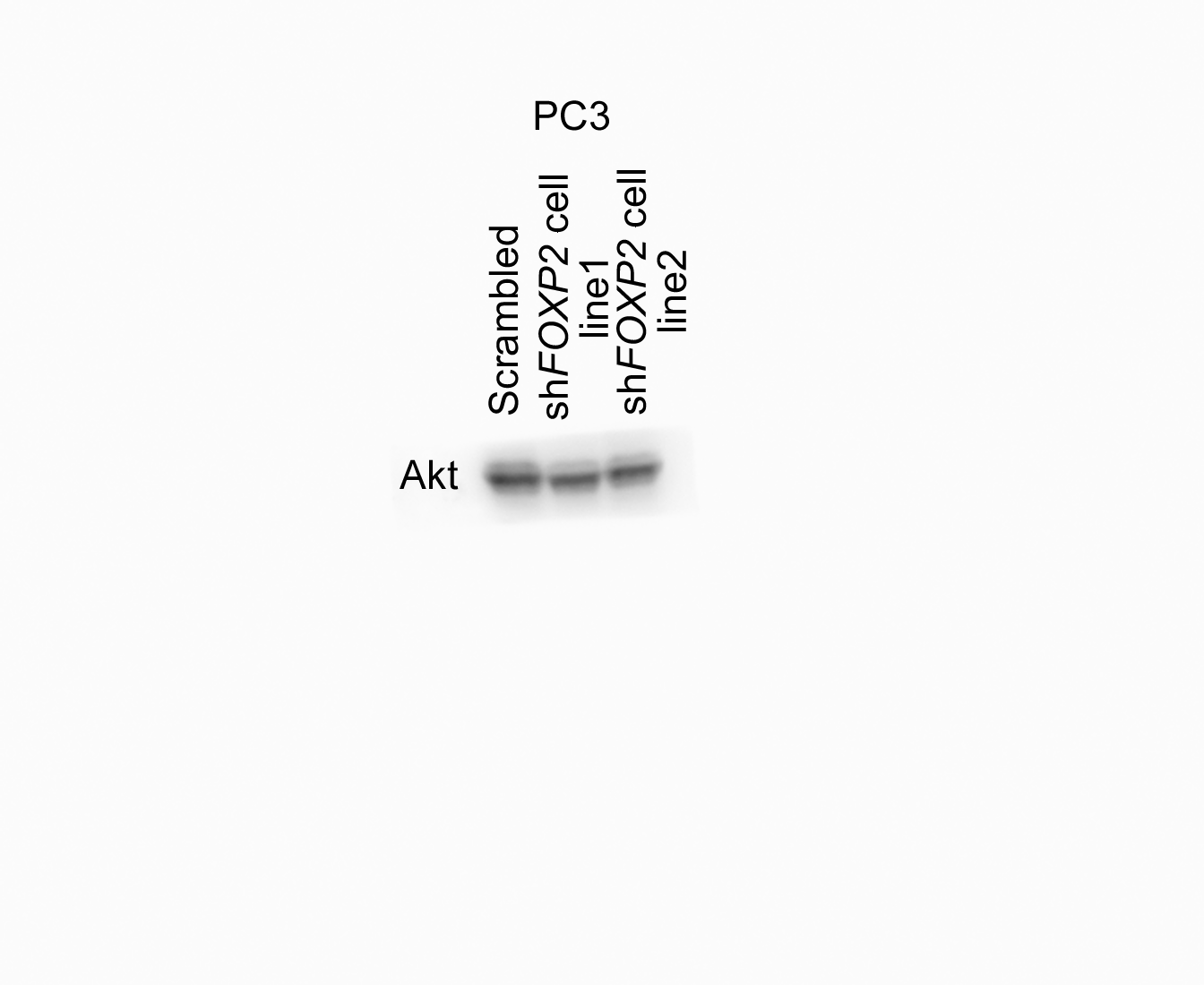

Supplement: Figure 3—source data 2. [file elife-81258-fig3-data2.zip › Figure 3-source data 2/Uncropped blots for Figure 3B in Main text/Figure 3B-source data 10.tif]

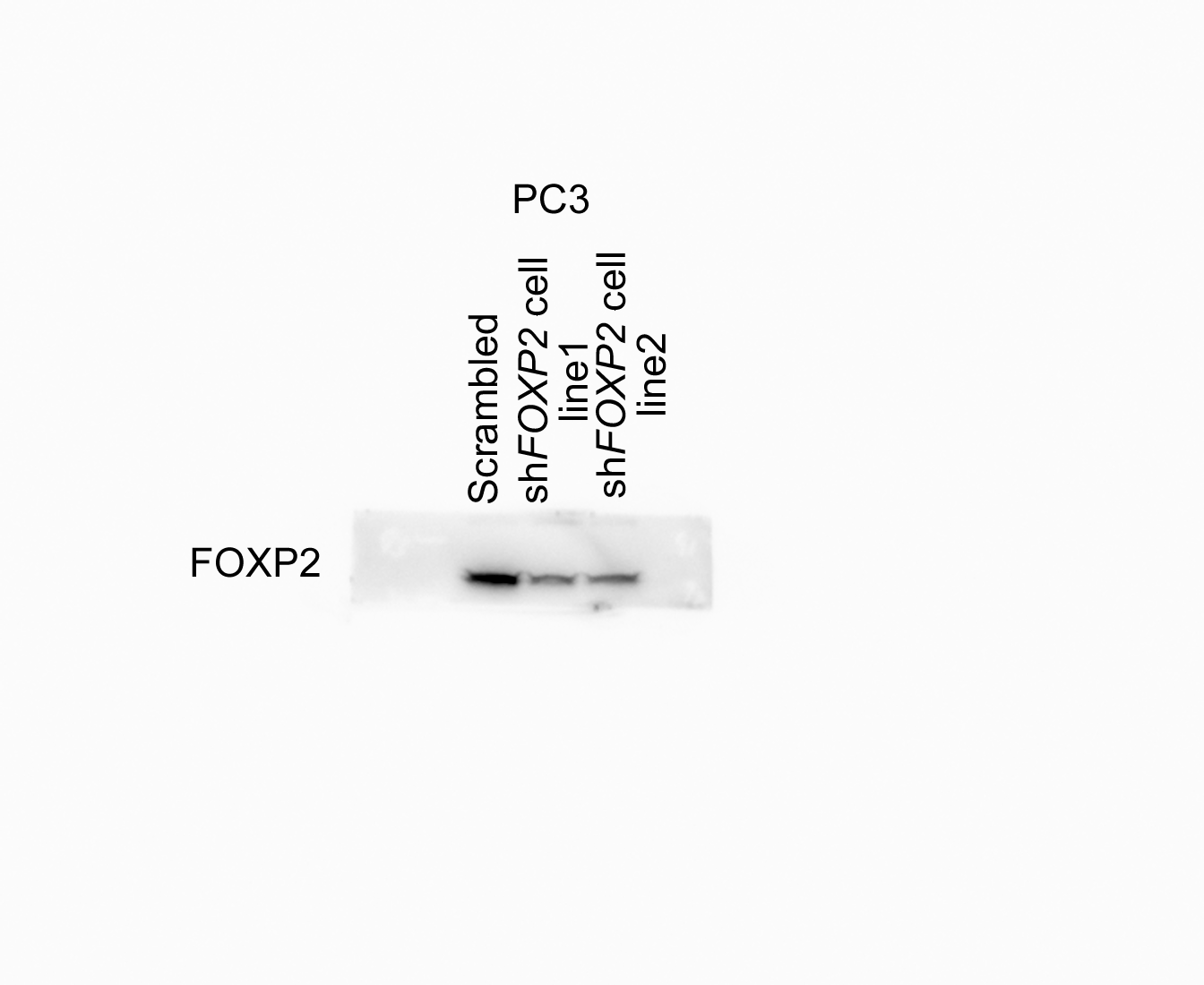

Supplement: Figure 3—source data 2. [file elife-81258-fig3-data2.zip › Figure 3-source data 2/Uncropped blots for Figure 3B in Main text/Figure 3B-source data 11.tif]

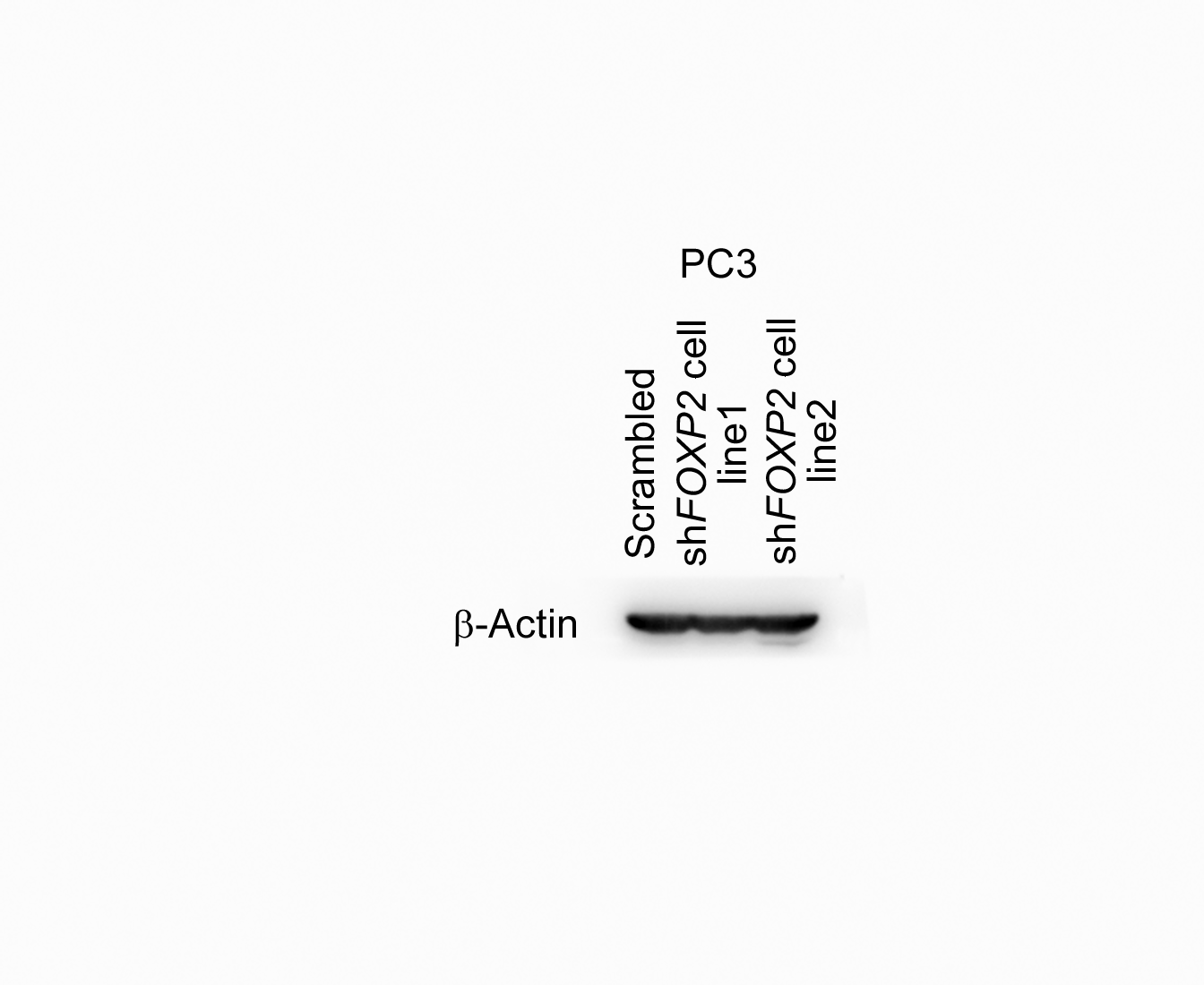

Supplement: Figure 3—source data 2. [file elife-81258-fig3-data2.zip › Figure 3-source data 2/Uncropped blots for Figure 3B in Main text/Figure 3B-source data 12.tif]

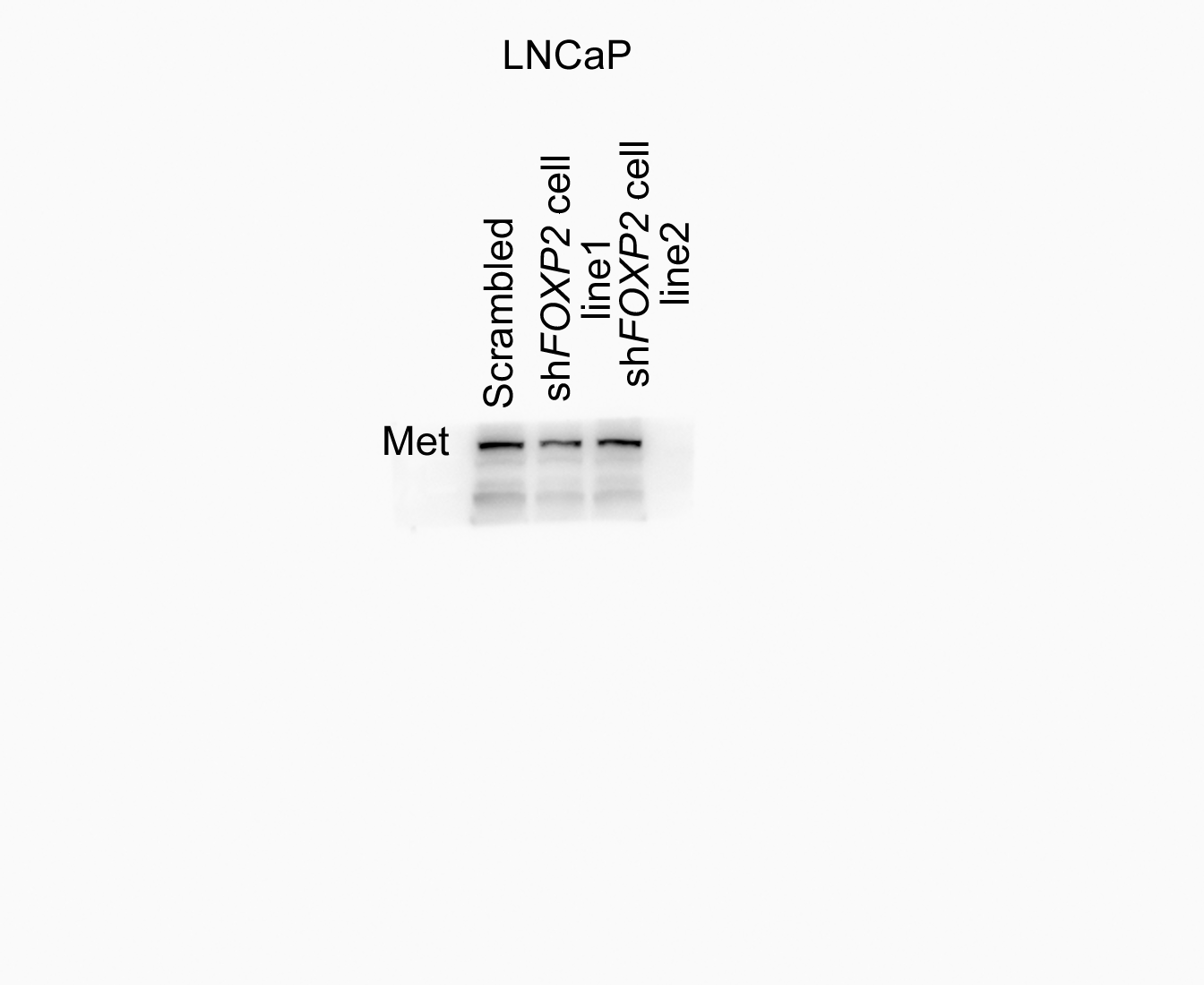

Supplement: Figure 3—source data 2. [file elife-81258-fig3-data2.zip › Figure 3-source data 2/Uncropped blots for Figure 3B in Main text/Figure 3B-source data 2.tif]

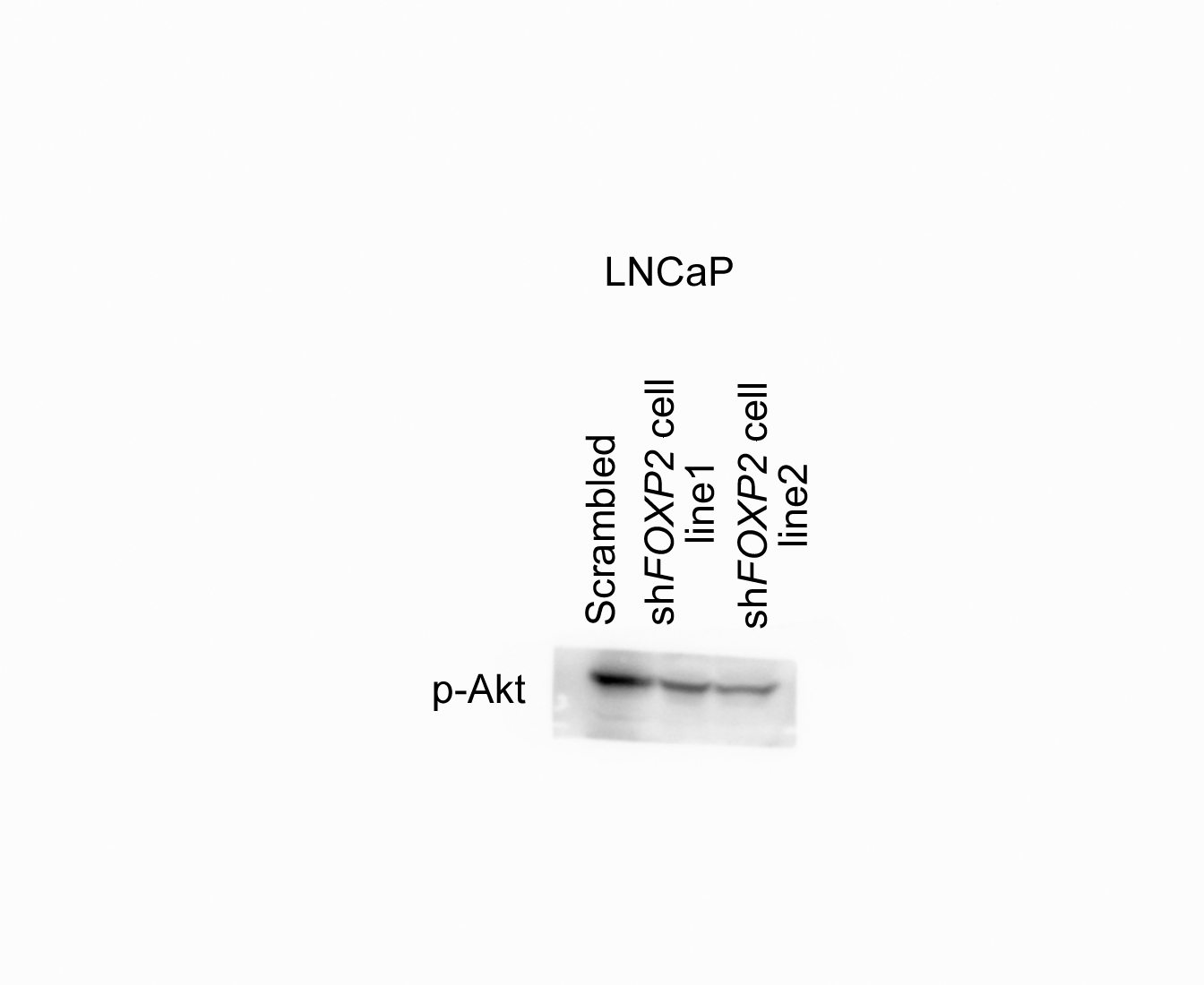

Supplement: Figure 3—source data 2. [file elife-81258-fig3-data2.zip › Figure 3-source data 2/Uncropped blots for Figure 3B in Main text/Figure 3B-source data 3.tif]

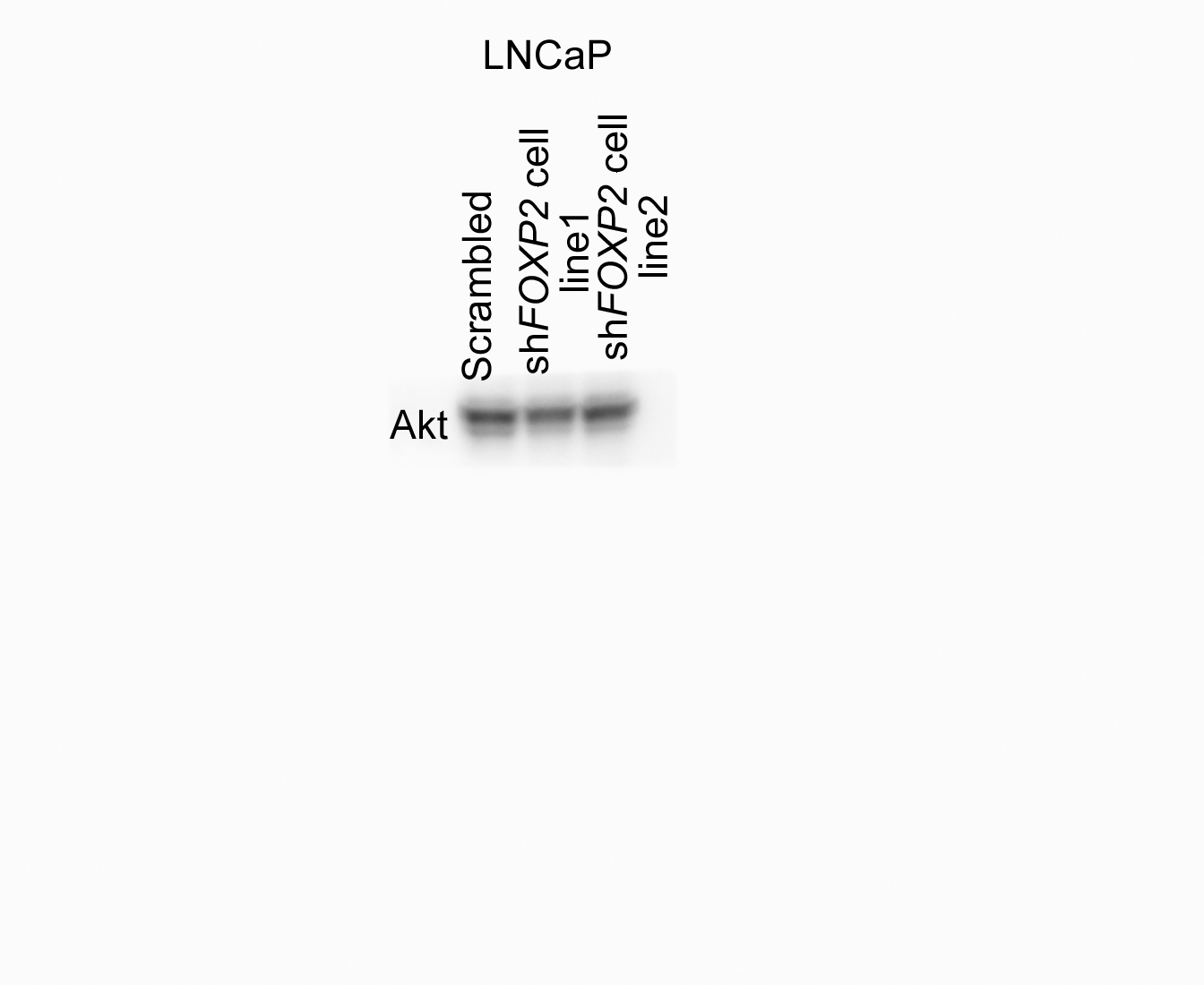

Supplement: Figure 3—source data 2. [file elife-81258-fig3-data2.zip › Figure 3-source data 2/Uncropped blots for Figure 3B in Main text/Figure 3B-source data 4.tif]

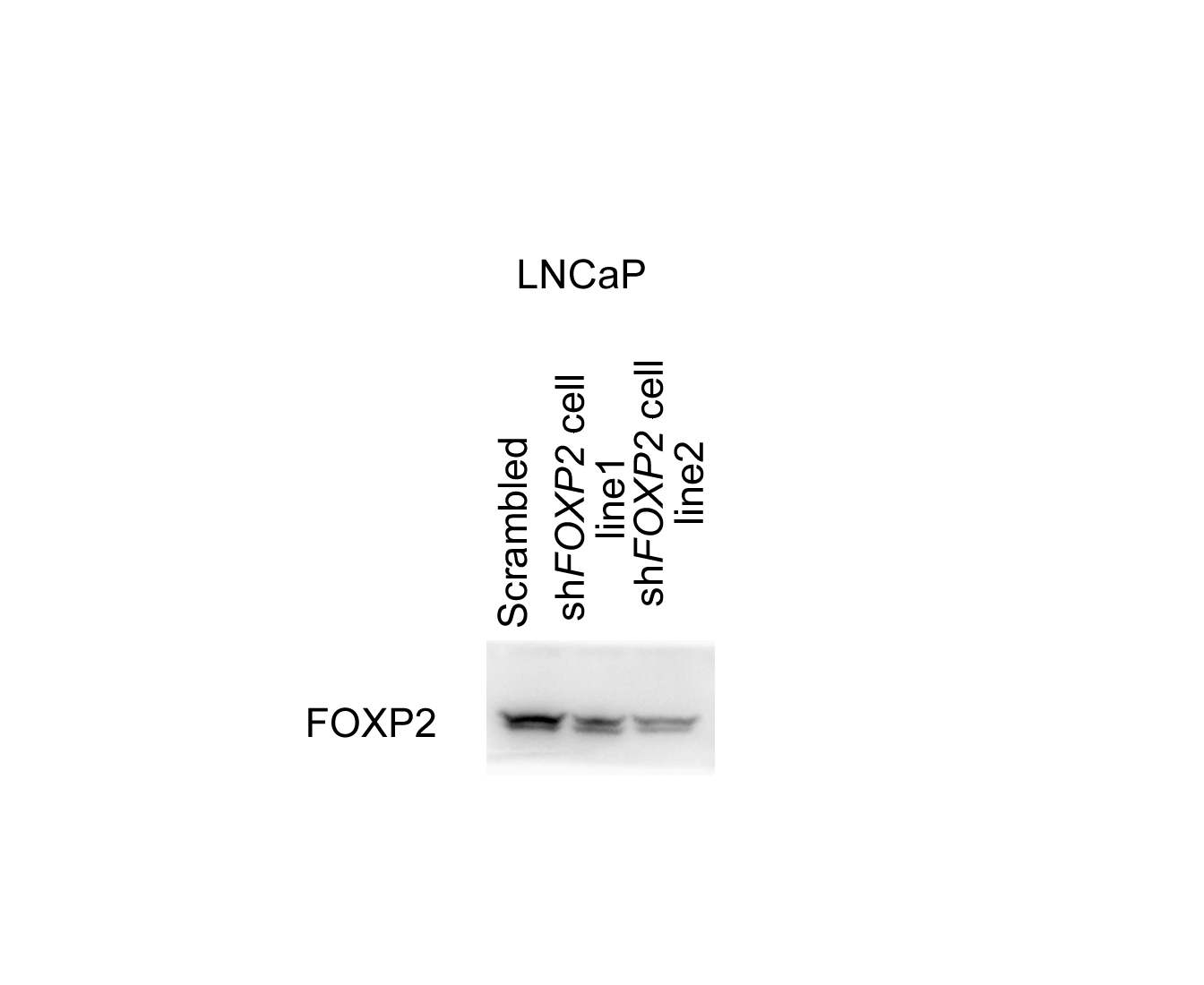

Supplement: Figure 3—source data 2. [file elife-81258-fig3-data2.zip › Figure 3-source data 2/Uncropped blots for Figure 3B in Main text/Figure 3B-source data 5.tif]

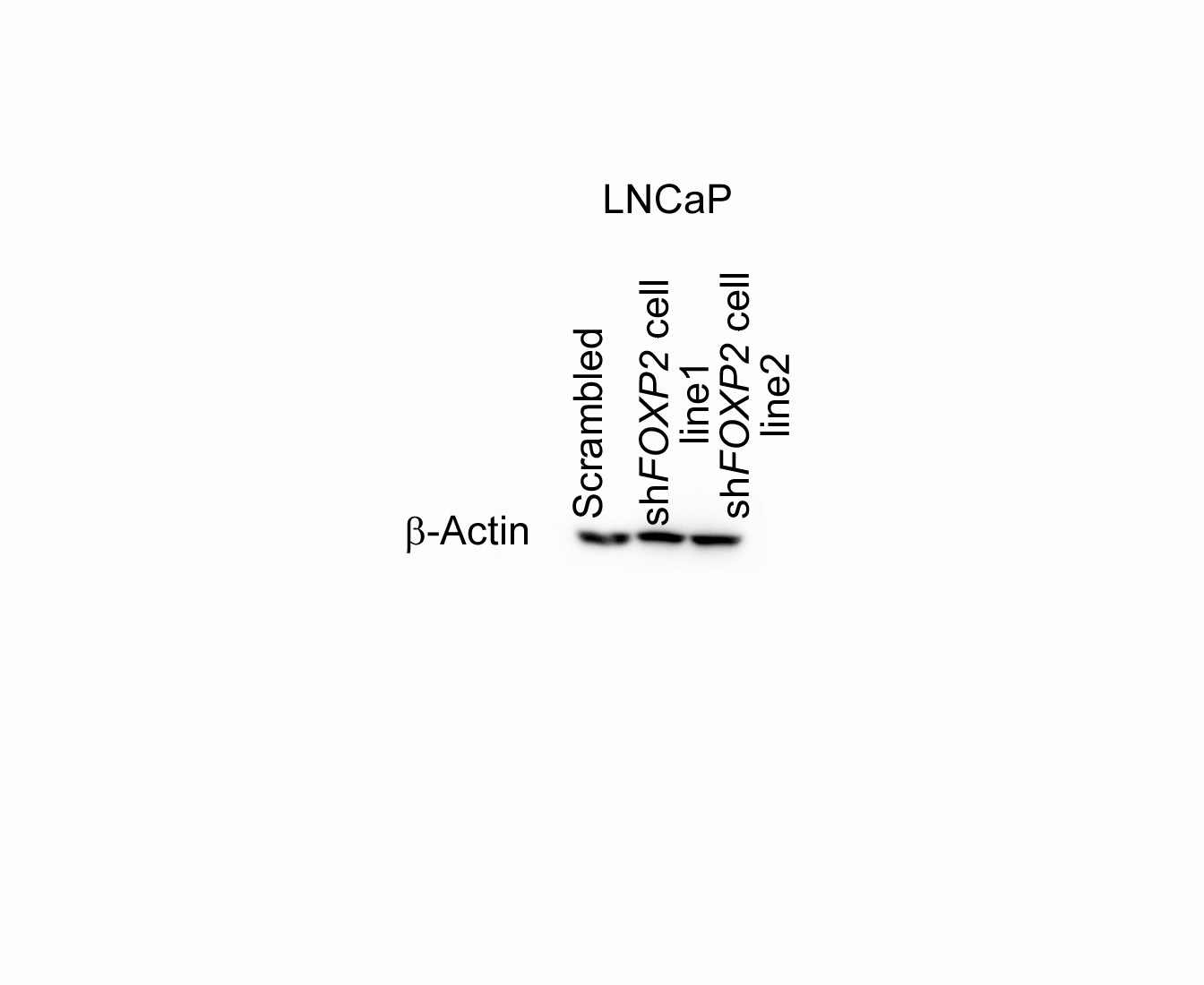

Supplement: Figure 3—source data 2. [file elife-81258-fig3-data2.zip › Figure 3-source data 2/Uncropped blots for Figure 3B in Main text/Figure 3B-source data 6.tif]

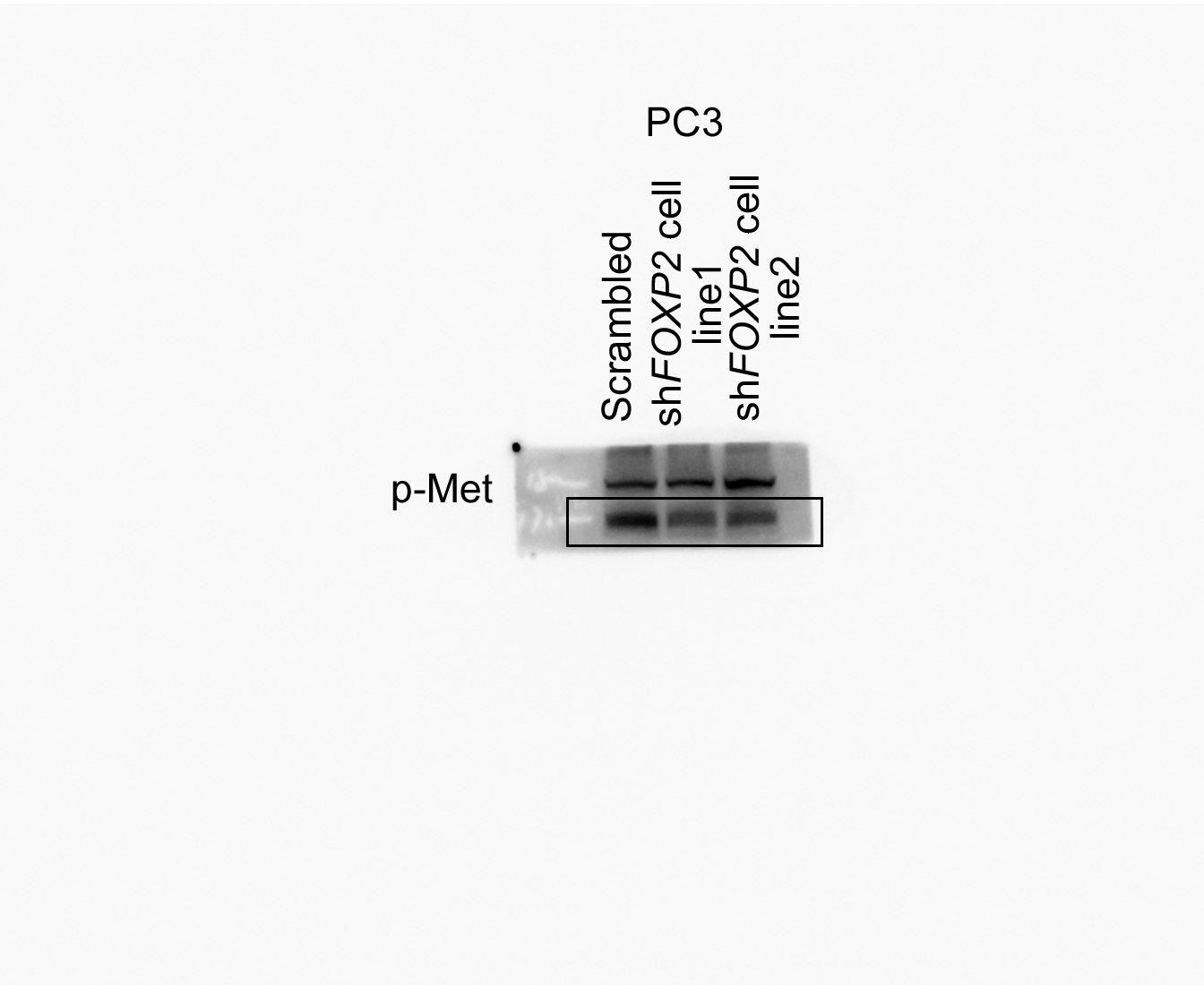

Supplement: Figure 3—source data 2. [file elife-81258-fig3-data2.zip › Figure 3-source data 2/Uncropped blots for Figure 3B in Main text/Figure 3B-source data 7.tif]

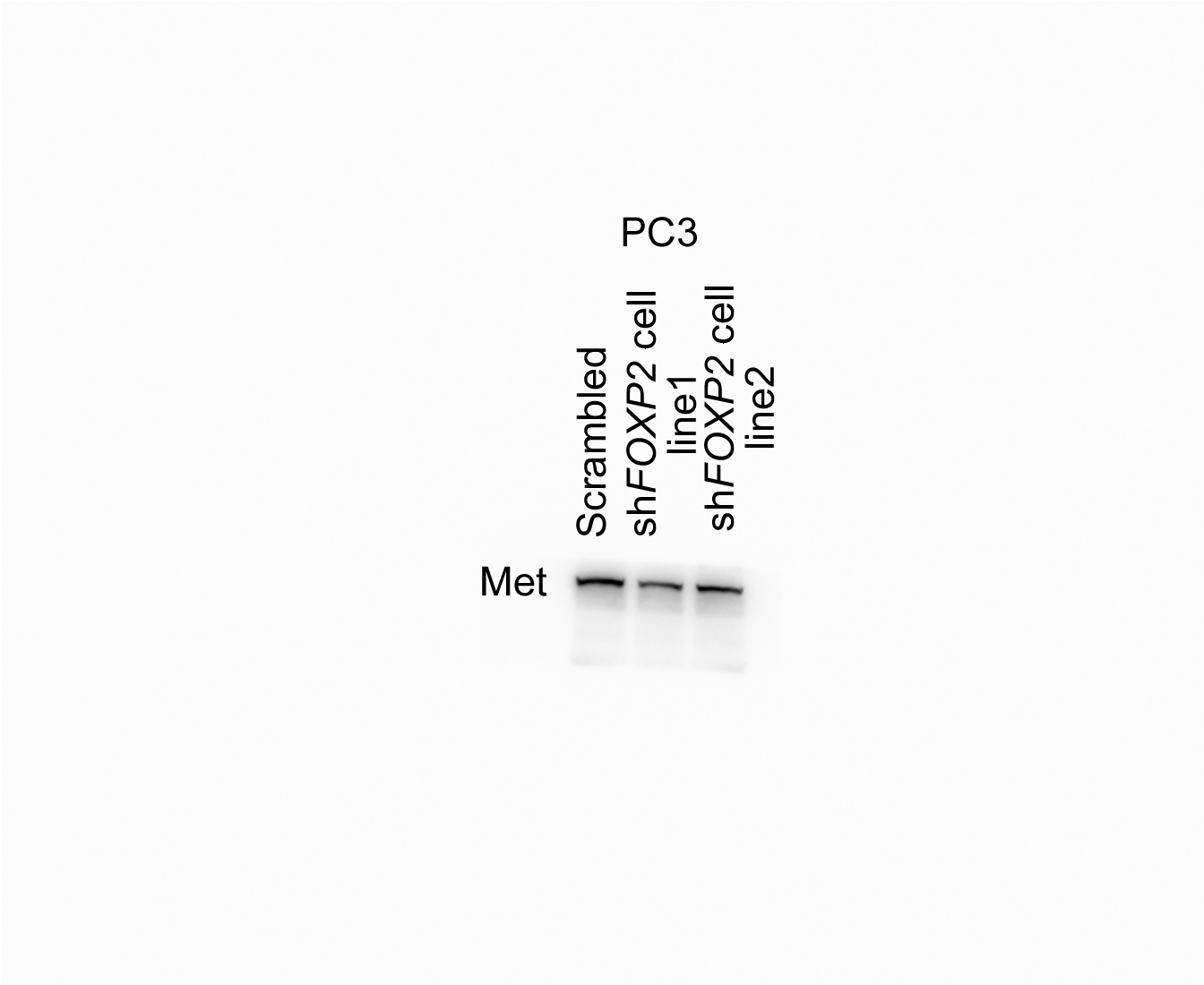

Supplement: Figure 3—source data 2. [file elife-81258-fig3-data2.zip › Figure 3-source data 2/Uncropped blots for Figure 3B in Main text/Figure 3B-source data 8.tif]

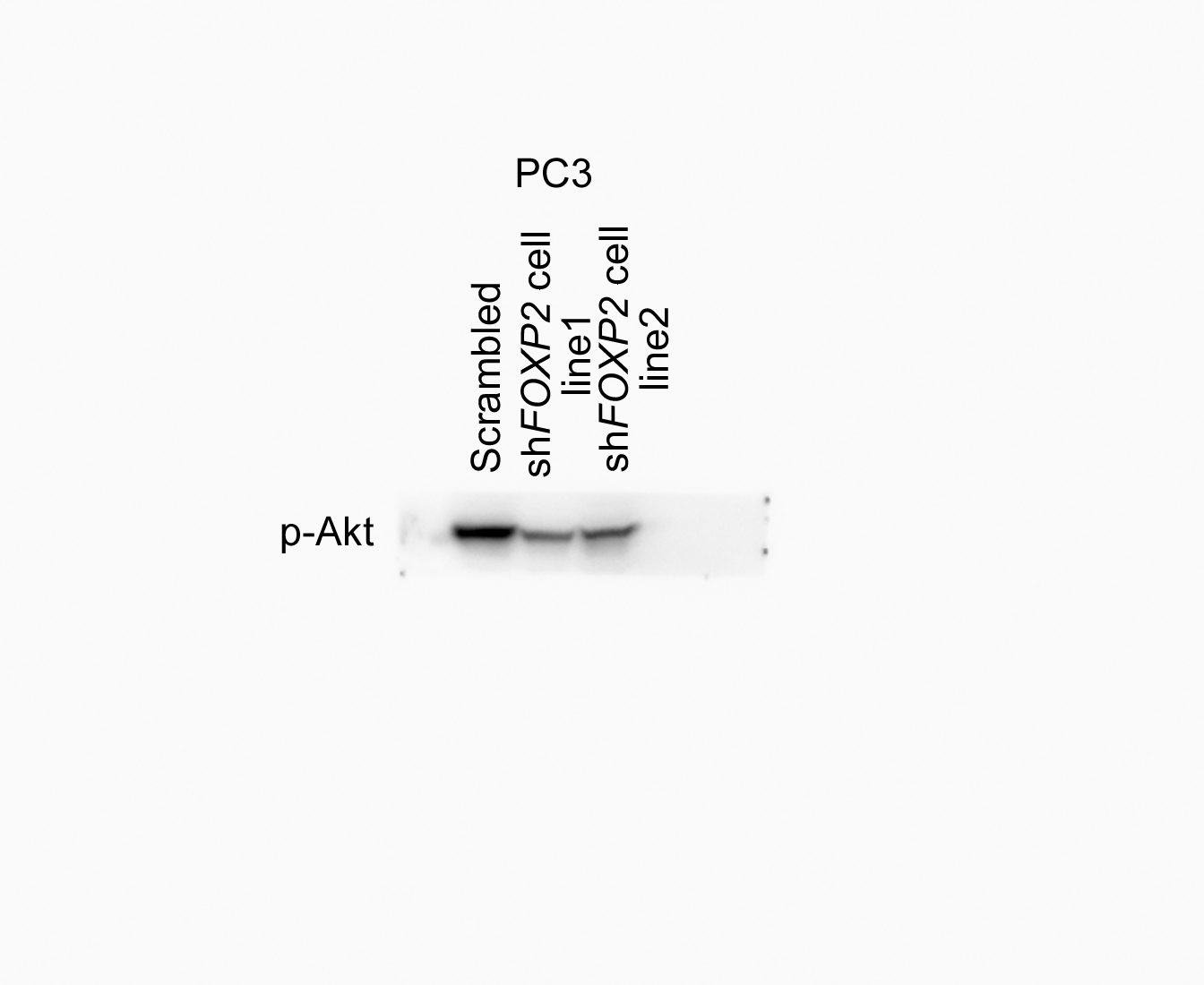

Supplement: Figure 3—source data 2. [file elife-81258-fig3-data2.zip › Figure 3-source data 2/Uncropped blots for Figure 3B in Main text/Figure 3B-source data 9.tif]

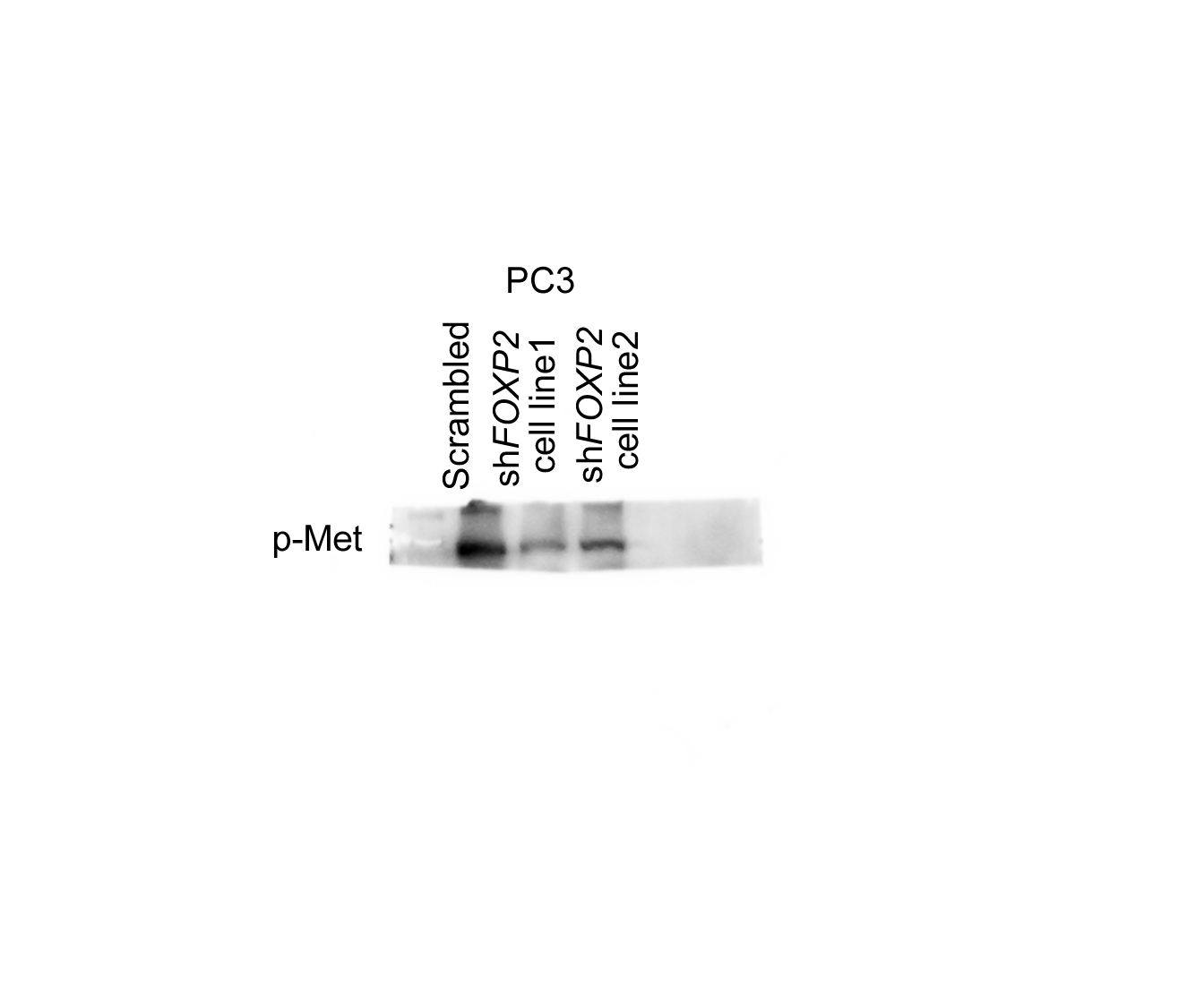

Supplement: Figure 3—source data 2. [file elife-81258-fig3-data2.zip › Figure 3-source data 2/Uncropped blots for Figure 3B PC3 cells repeat/repeat1/P-MET.tif]

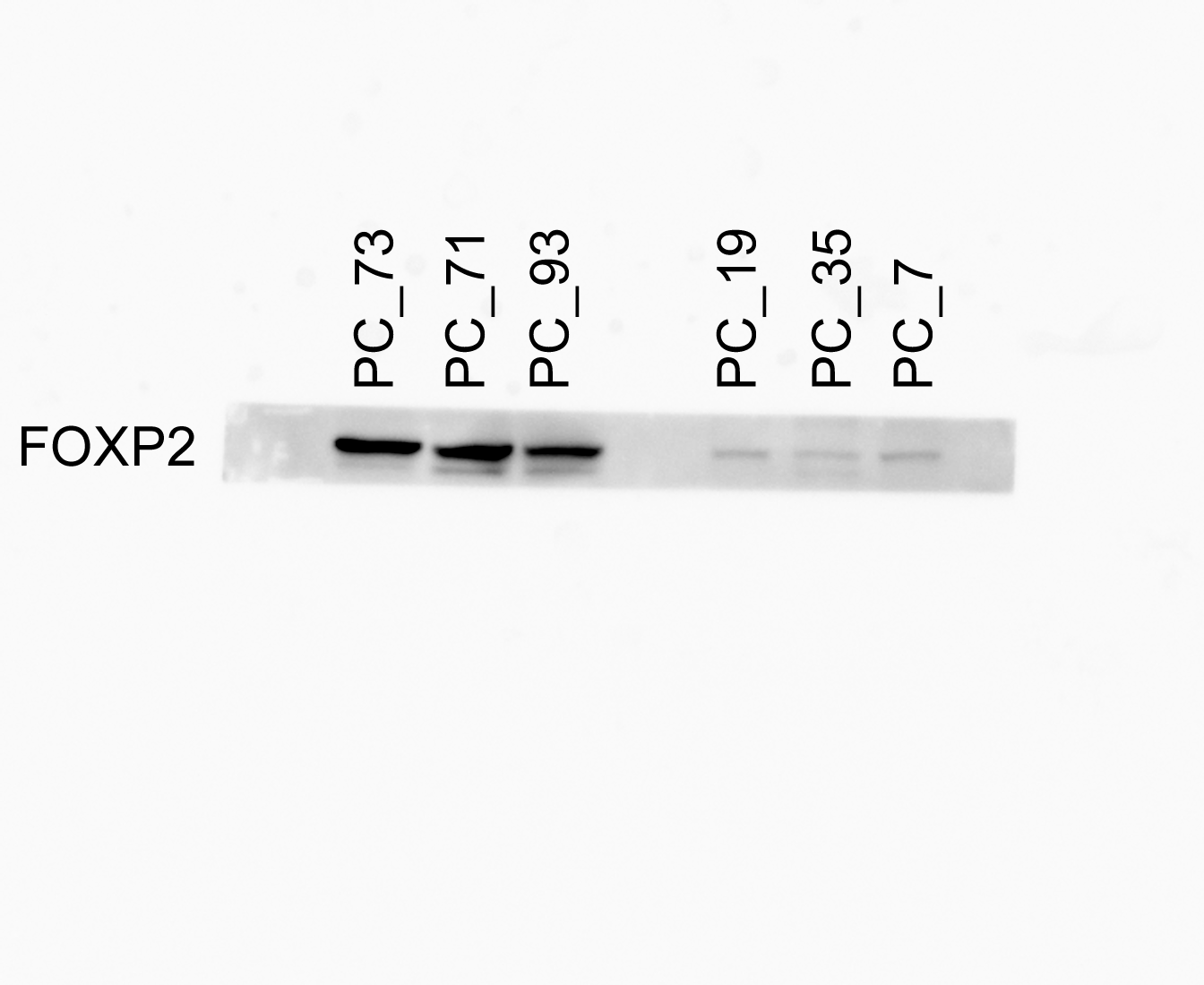

Supplement: Figure 3—source data 3. [file elife-81258-fig3-data3.zip › Figure 3-source data 3/Uncropped blots for Figure 3C/Figure 3C-source data 1.tif]

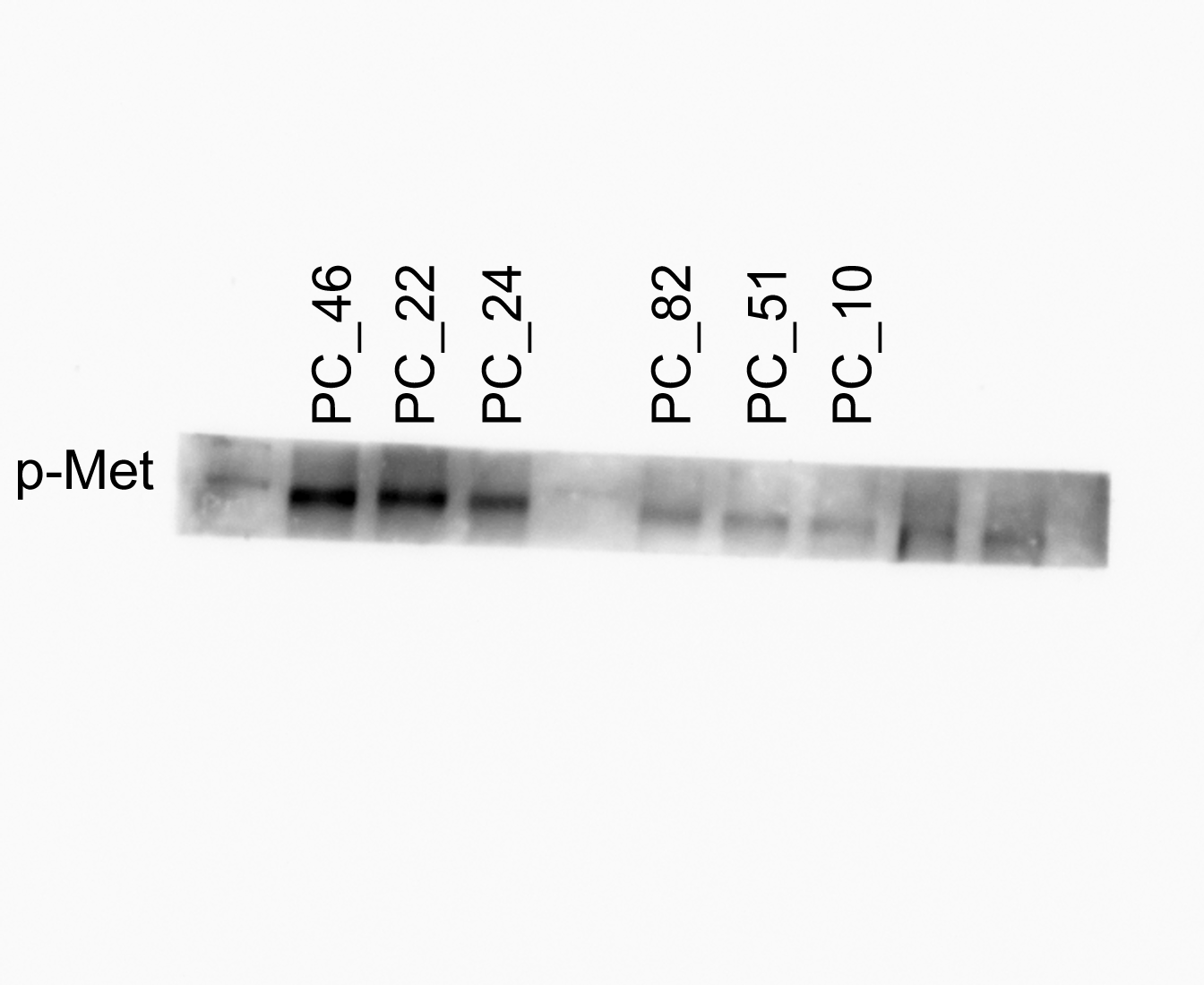

Supplement: Figure 3—source data 3. [file elife-81258-fig3-data3.zip › Figure 3-source data 3/Uncropped blots for Figure 3C/Figure 3C-source data 10.tif]

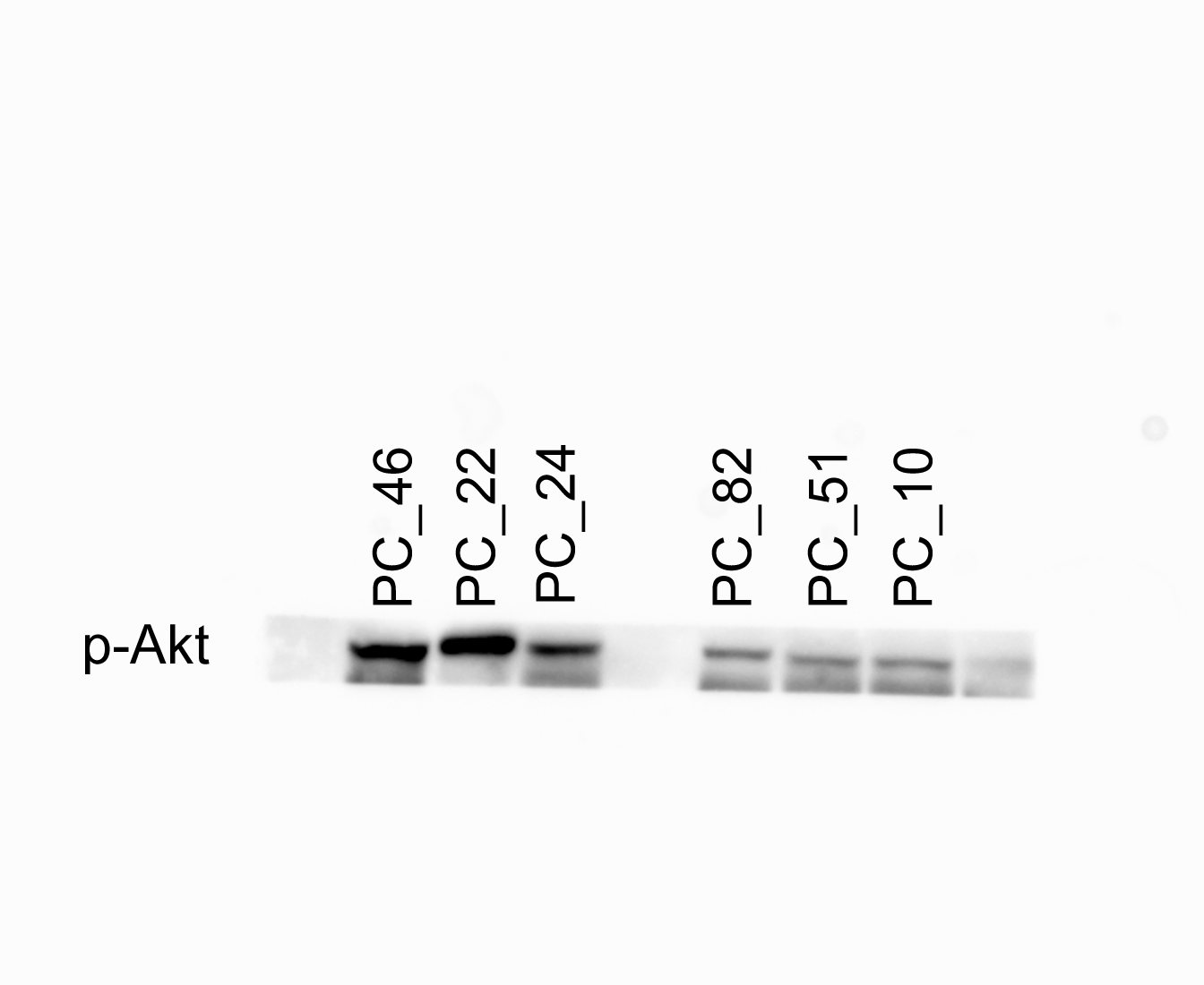

Supplement: Figure 3—source data 3. [file elife-81258-fig3-data3.zip › Figure 3-source data 3/Uncropped blots for Figure 3C/Figure 3C-source data 11.tif]

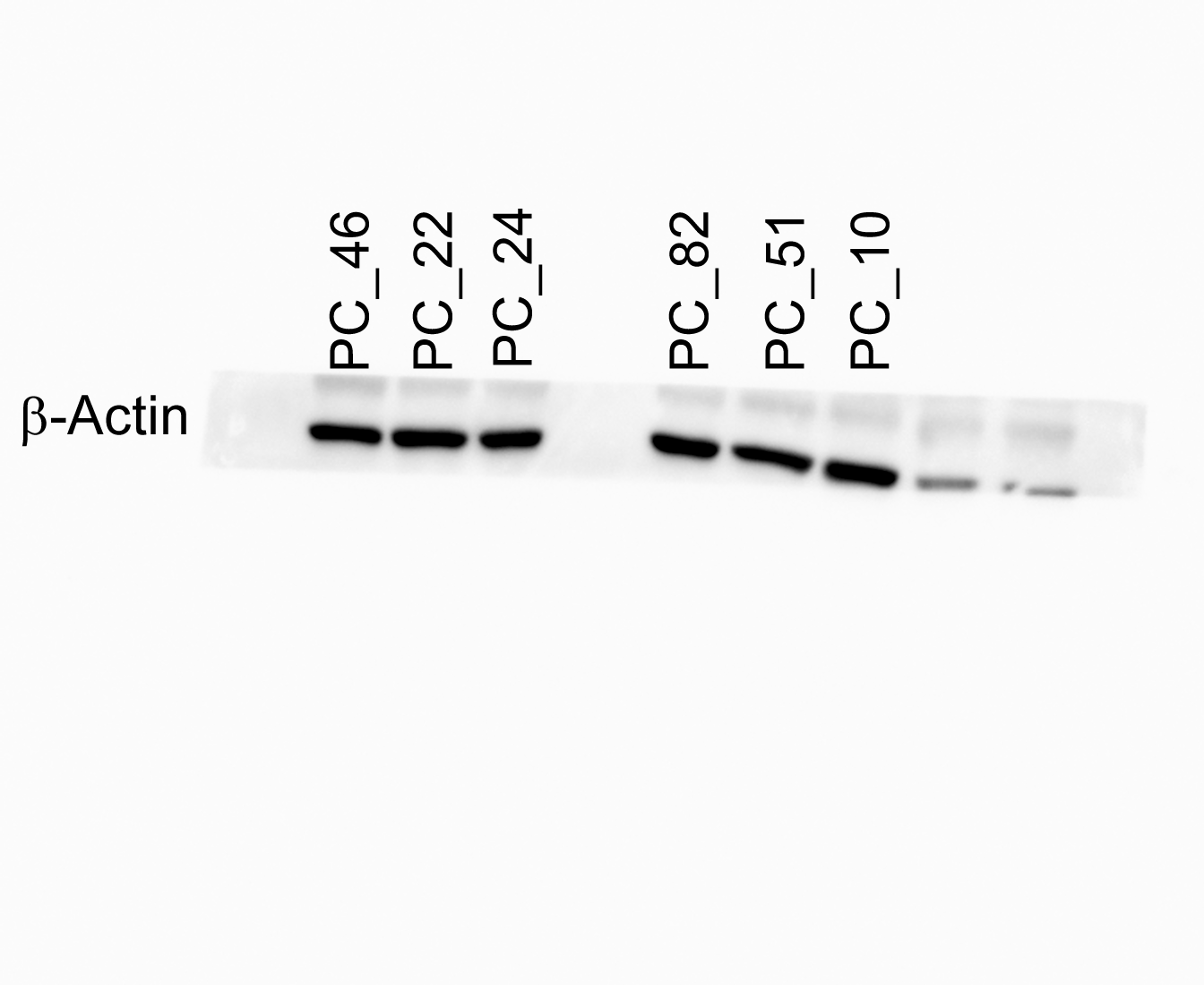

Supplement: Figure 3—source data 3. [file elife-81258-fig3-data3.zip › Figure 3-source data 3/Uncropped blots for Figure 3C/Figure 3C-source data 12.tif]

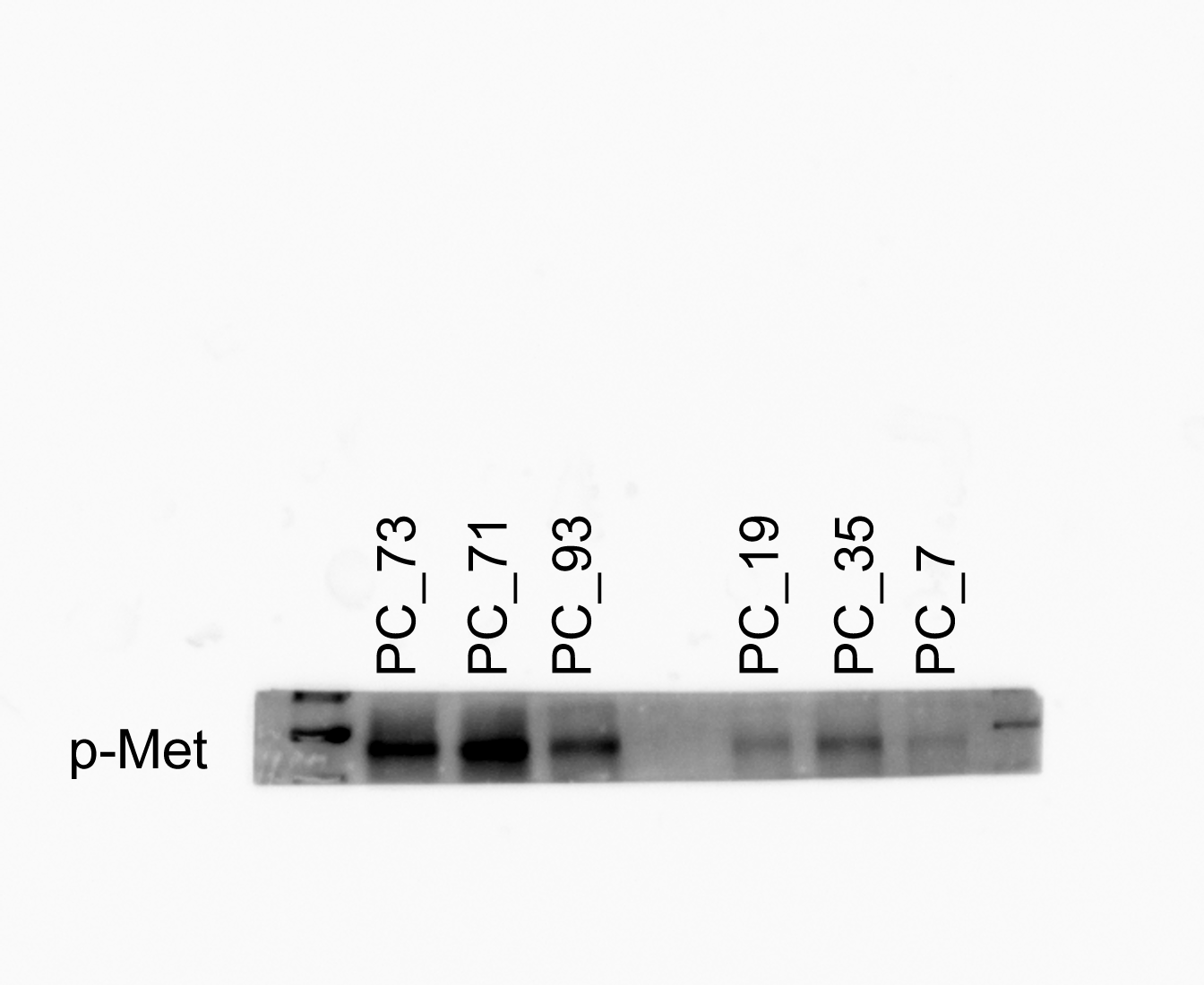

Supplement: Figure 3—source data 3. [file elife-81258-fig3-data3.zip › Figure 3-source data 3/Uncropped blots for Figure 3C/Figure 3C-source data 2.tif]

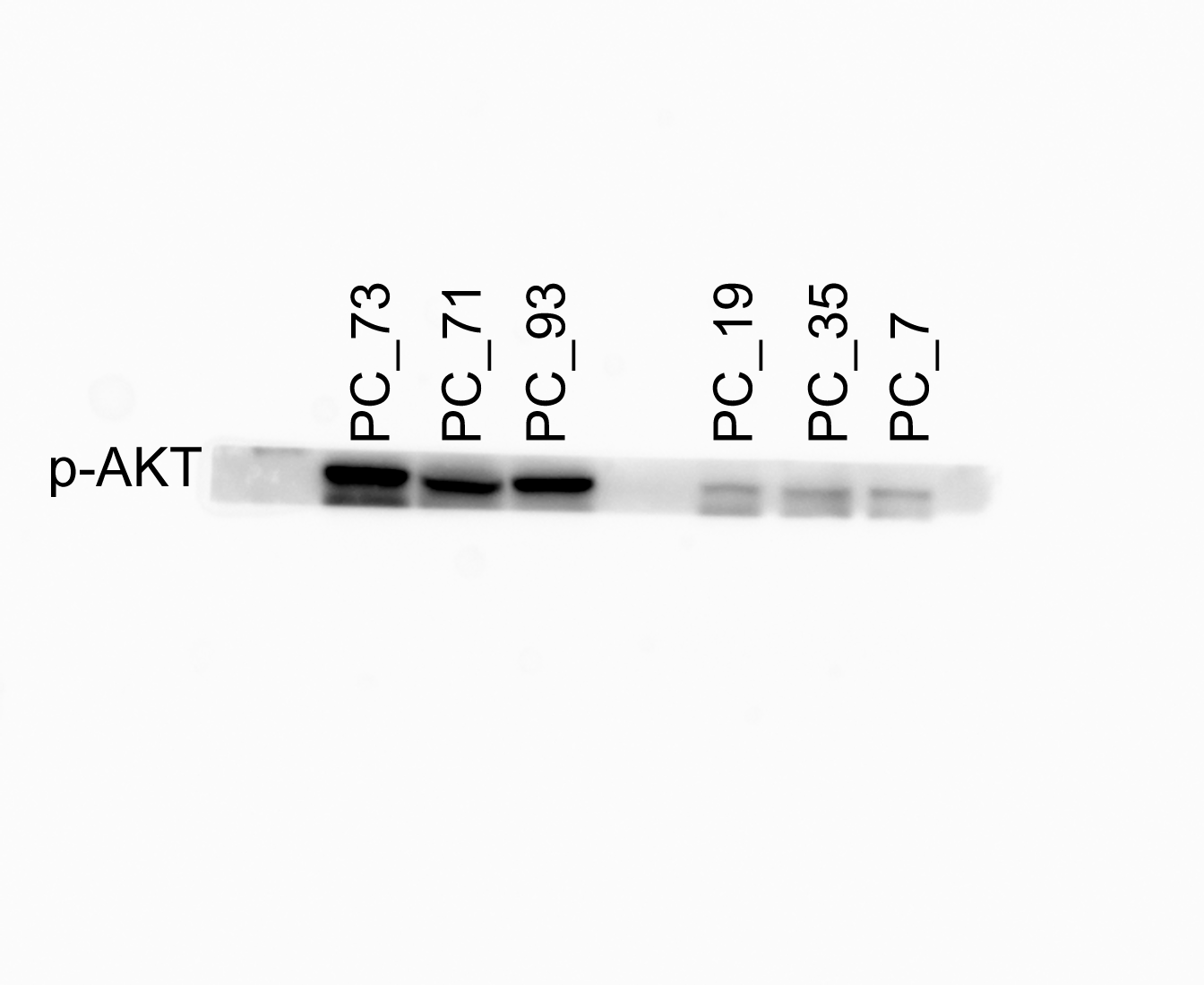

Supplement: Figure 3—source data 3. [file elife-81258-fig3-data3.zip › Figure 3-source data 3/Uncropped blots for Figure 3C/Figure 3C-source data 3.tif]

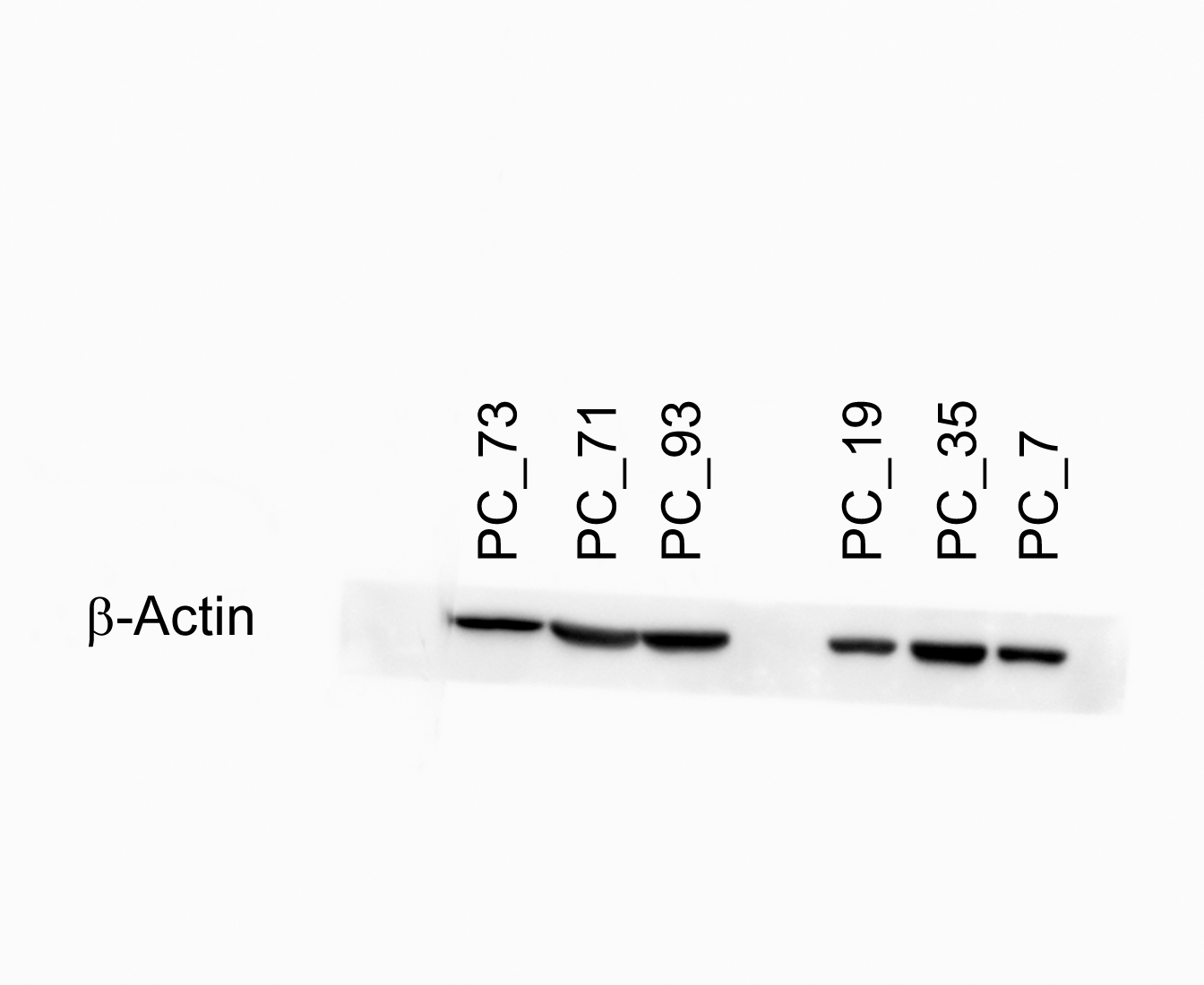

Supplement: Figure 3—source data 3. [file elife-81258-fig3-data3.zip › Figure 3-source data 3/Uncropped blots for Figure 3C/Figure 3C-source data 4.tif]

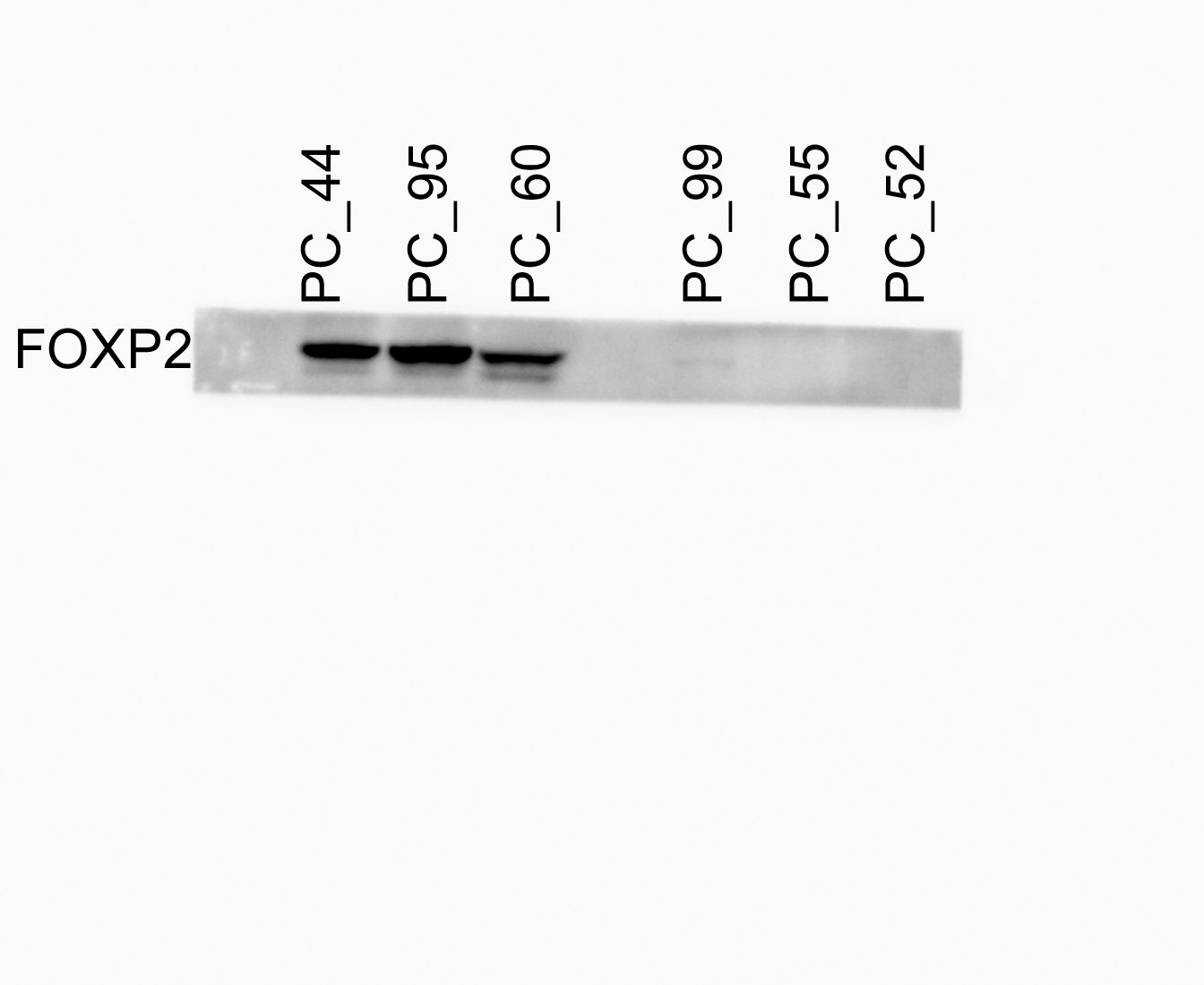

Supplement: Figure 3—source data 3. [file elife-81258-fig3-data3.zip › Figure 3-source data 3/Uncropped blots for Figure 3C/Figure 3C-source data 5.tif]

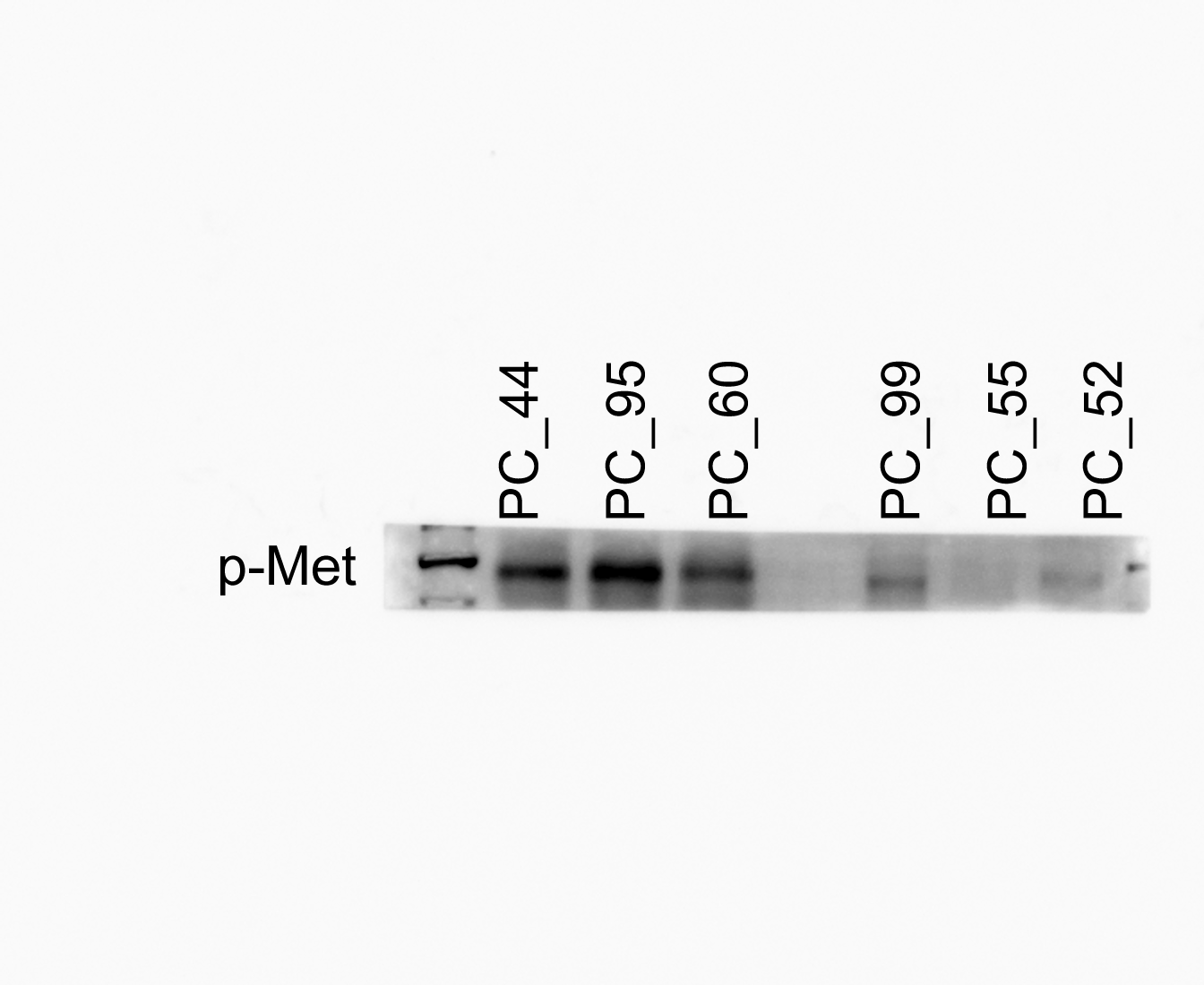

Supplement: Figure 3—source data 3. [file elife-81258-fig3-data3.zip › Figure 3-source data 3/Uncropped blots for Figure 3C/Figure 3C-source data 6.tif]

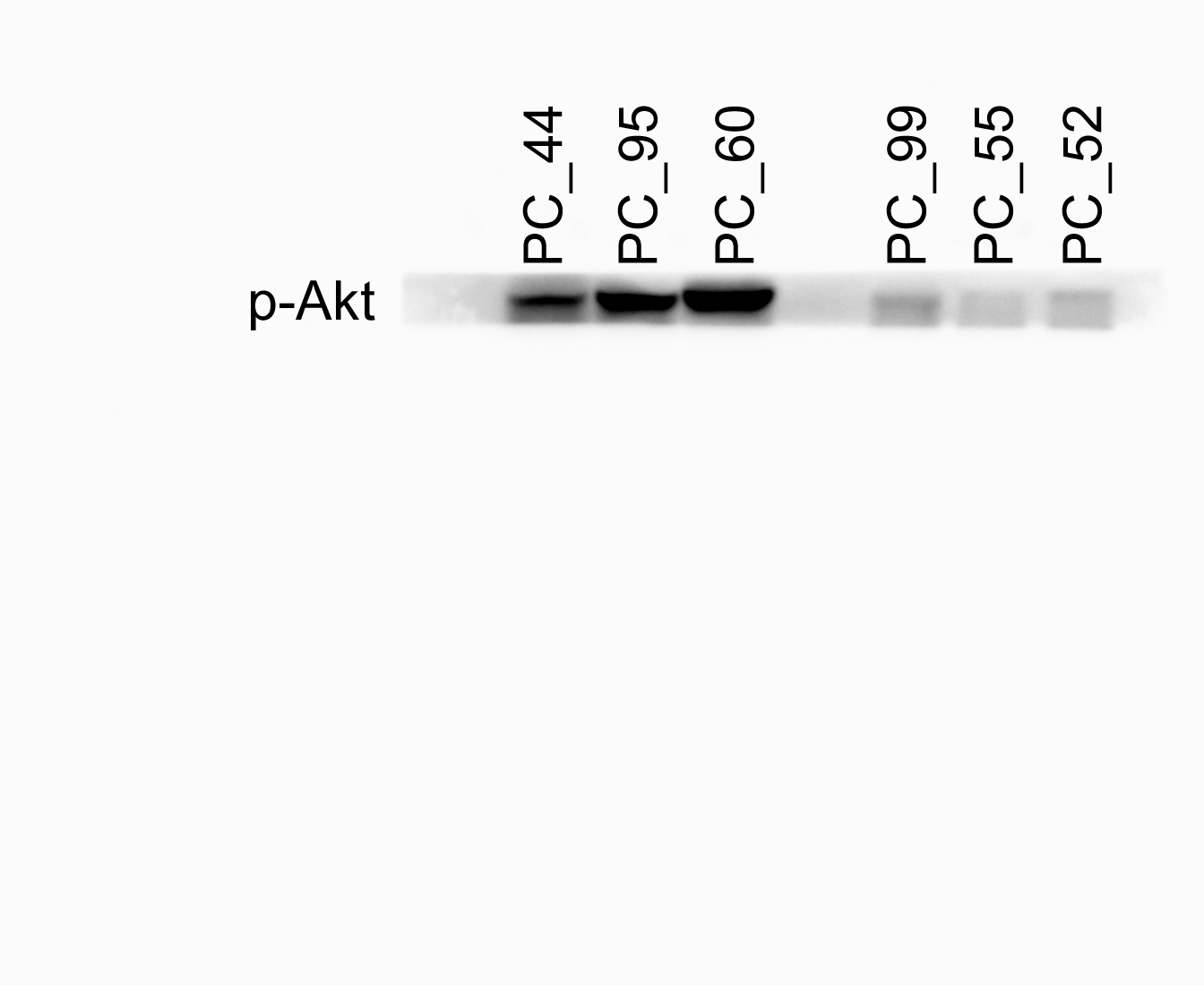

Supplement: Figure 3—source data 3. [file elife-81258-fig3-data3.zip › Figure 3-source data 3/Uncropped blots for Figure 3C/Figure 3C-source data 7.tif]

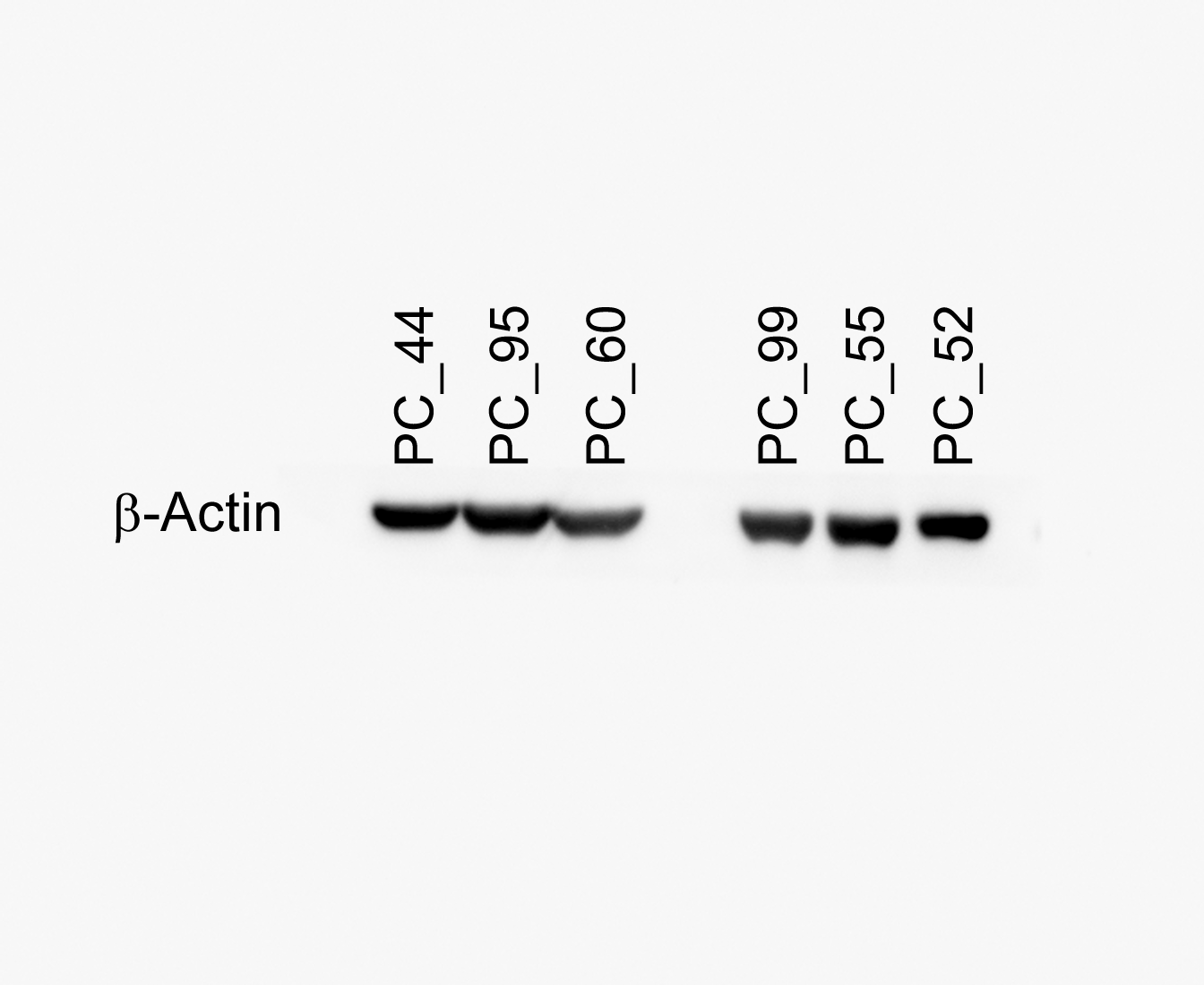

Supplement: Figure 3—source data 3. [file elife-81258-fig3-data3.zip › Figure 3-source data 3/Uncropped blots for Figure 3C/Figure 3C-source data 8.tif]

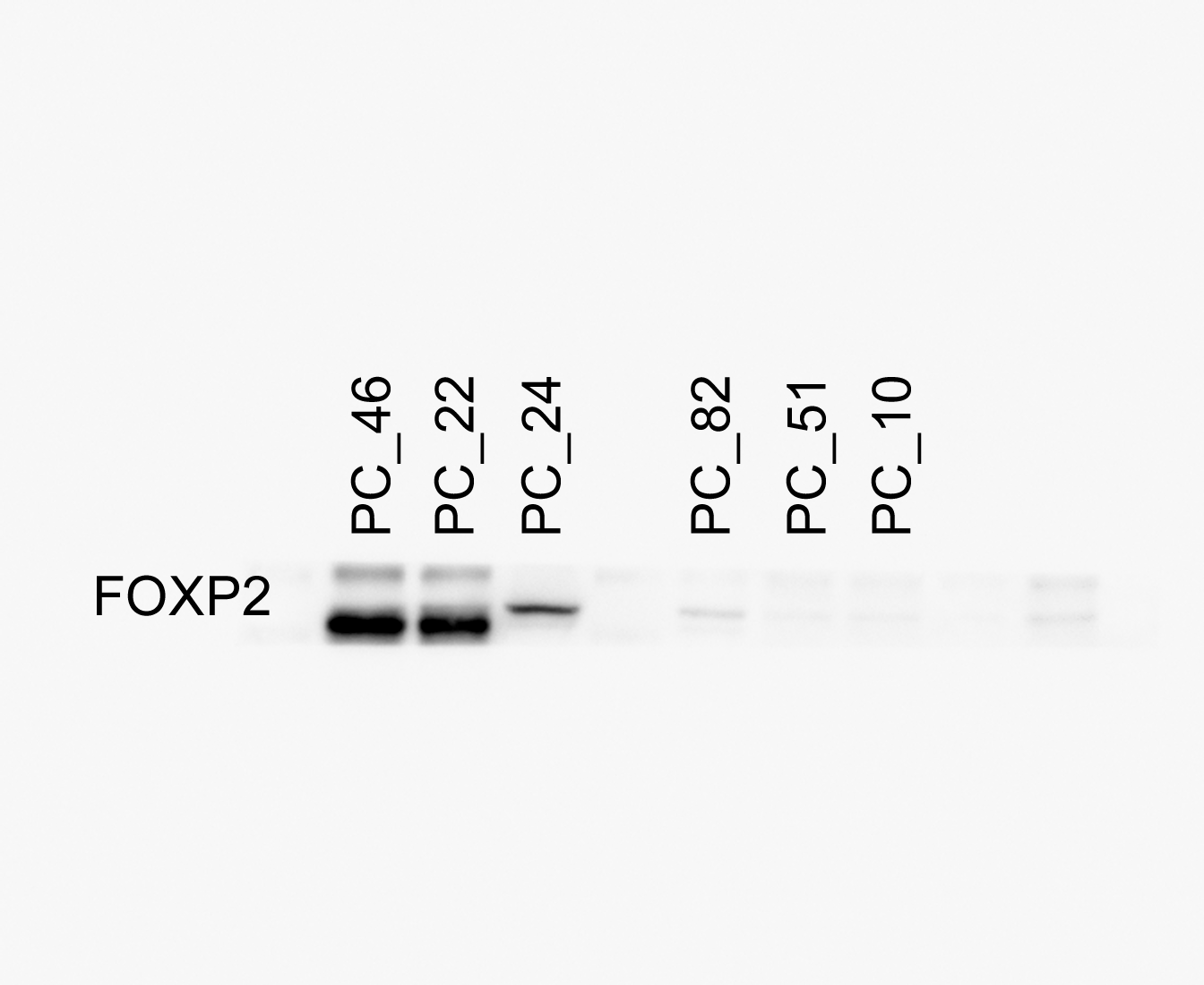

Supplement: Figure 3—source data 3. [file elife-81258-fig3-data3.zip › Figure 3-source data 3/Uncropped blots for Figure 3C/Figure 3C-source data 9.tif]

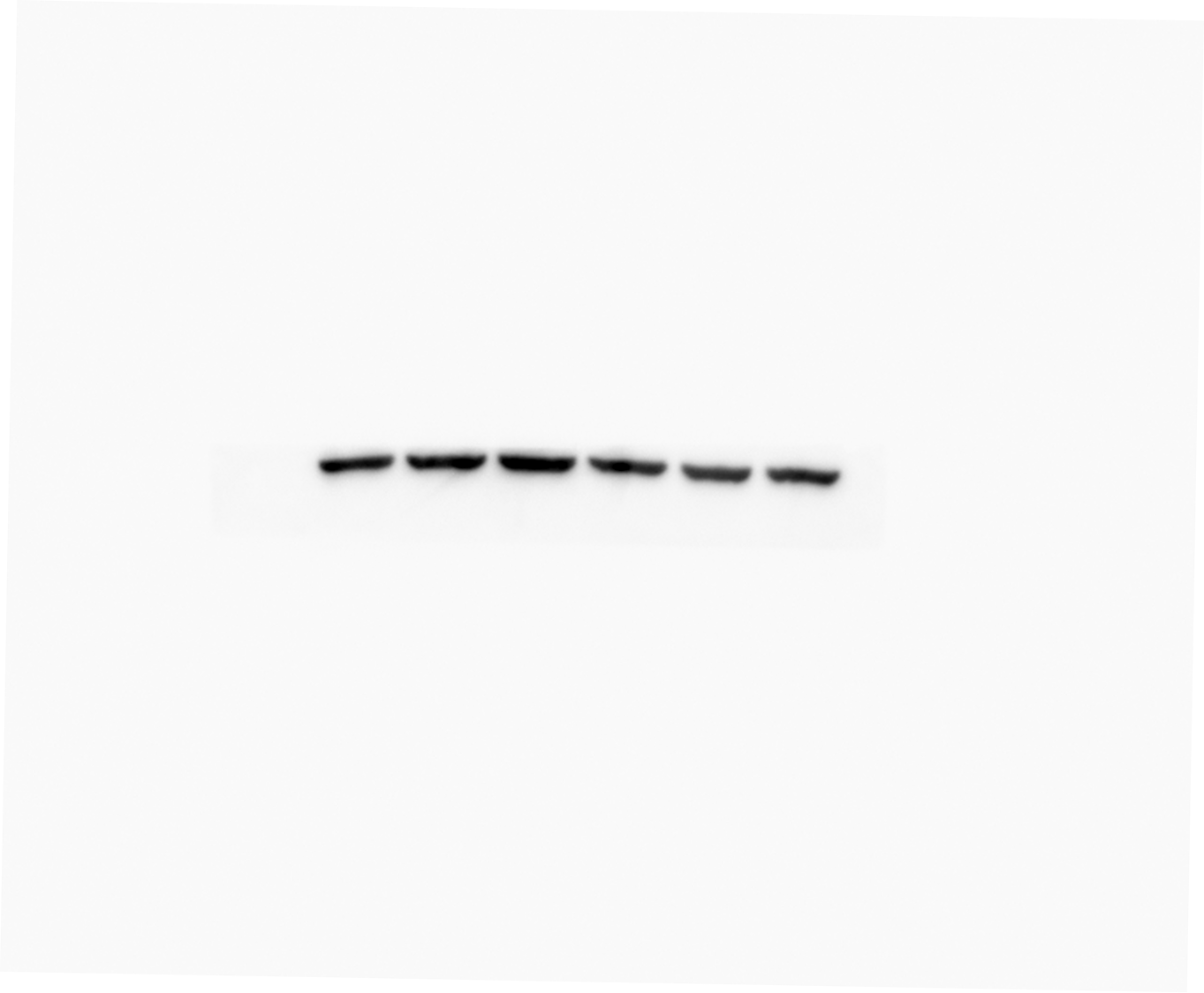

Supplement: Figure 3—source data 4. [file elife-81258-fig3-data4.zip › Figure 3-source data 4/Original files for Figure 3F NIH3T3 cells/Original files for Figure 3F NIH3T3 cells in Main text/2-ACTIN.tif]

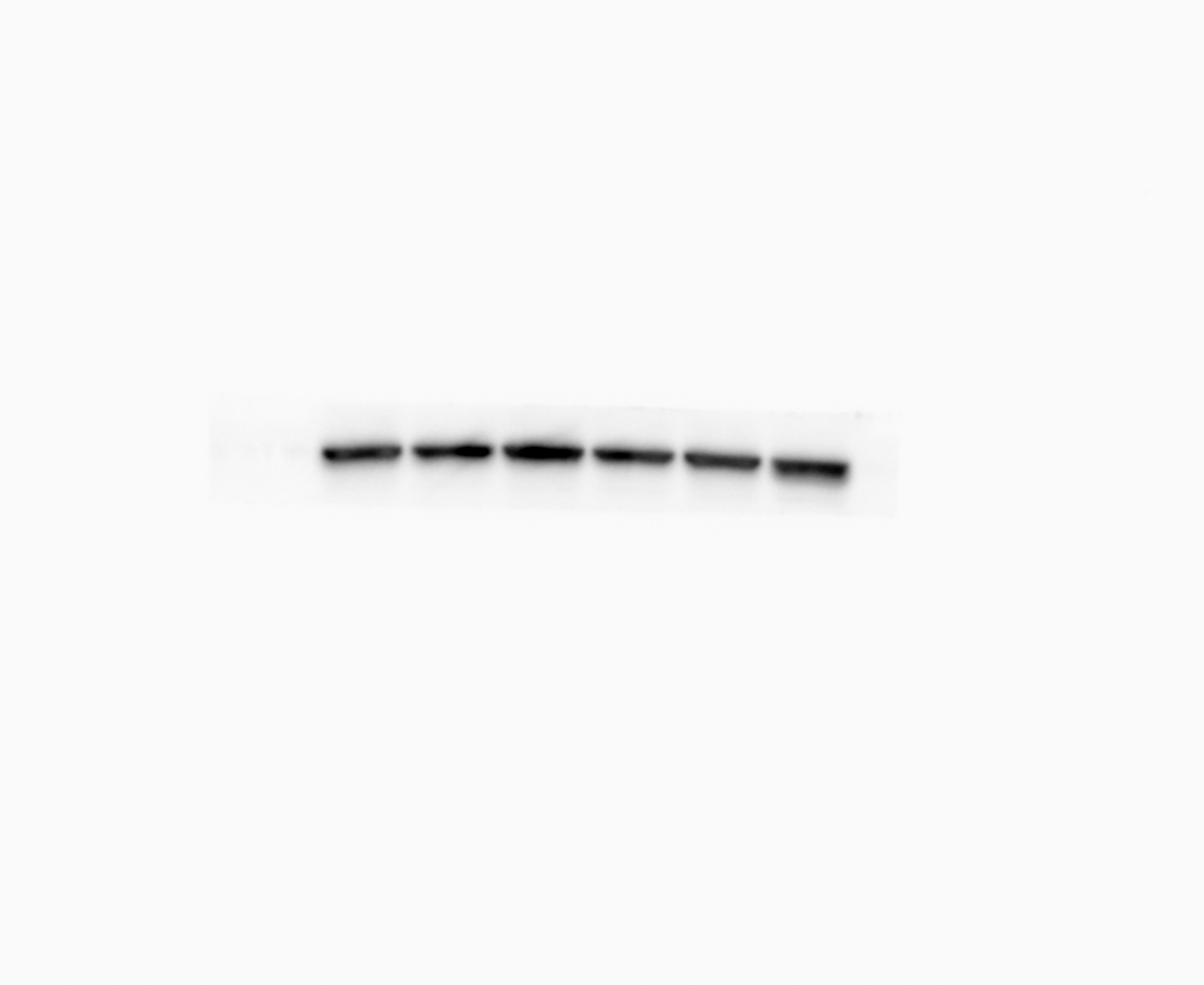

Supplement: Figure 3—source data 4. [file elife-81258-fig3-data4.zip › Figure 3-source data 4/Original files for Figure 3F NIH3T3 cells/Original files for Figure 3F NIH3T3 cells in Main text/3-T-AKT.tif]

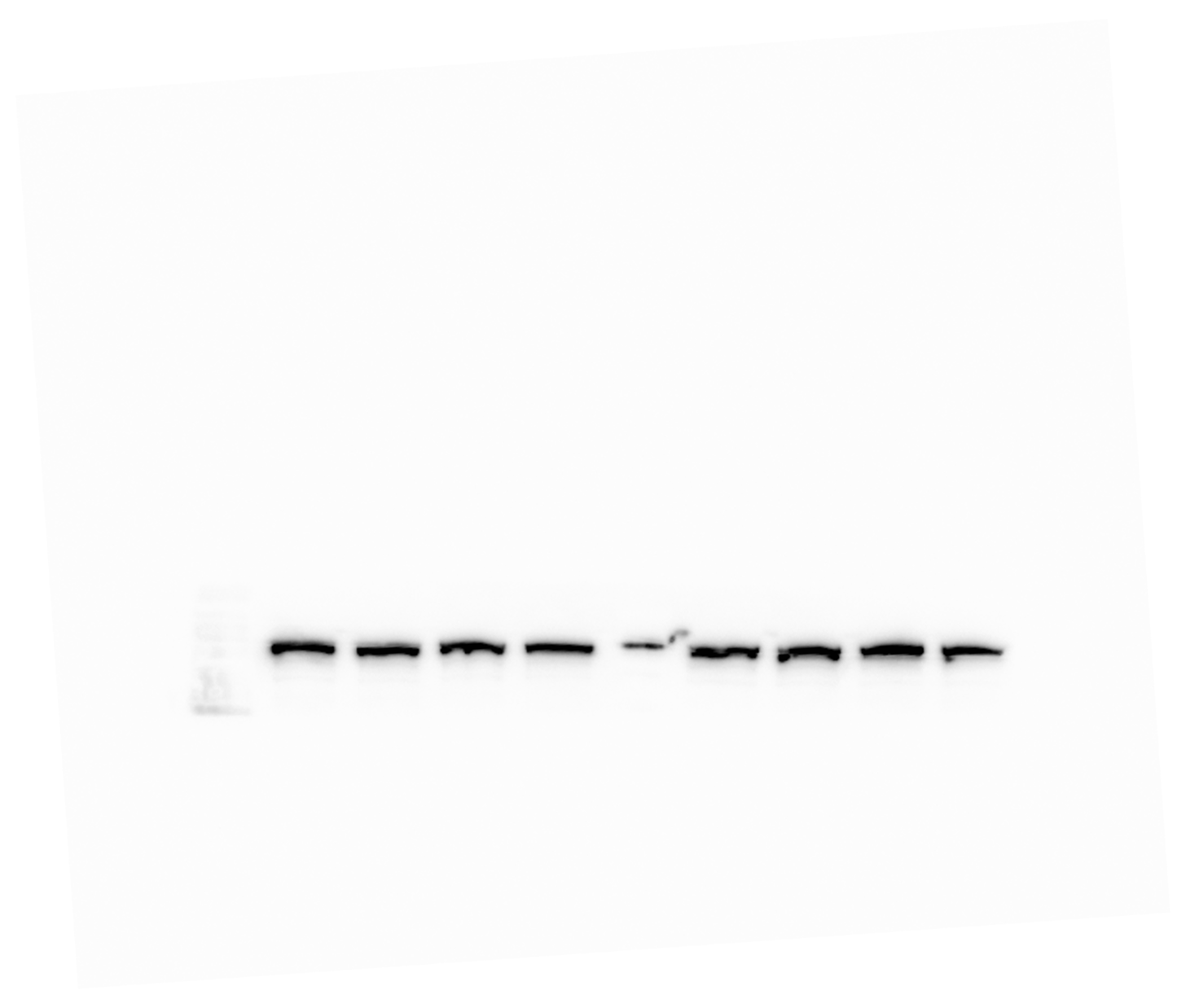

Supplement: Figure 3—source data 4. [file elife-81258-fig3-data4.zip › Figure 3-source data 4/Original files for Figure 3F NIH3T3 cells/Original files for Figure 3F NIH3T3 cells Repeat/1-T-MET.tif]

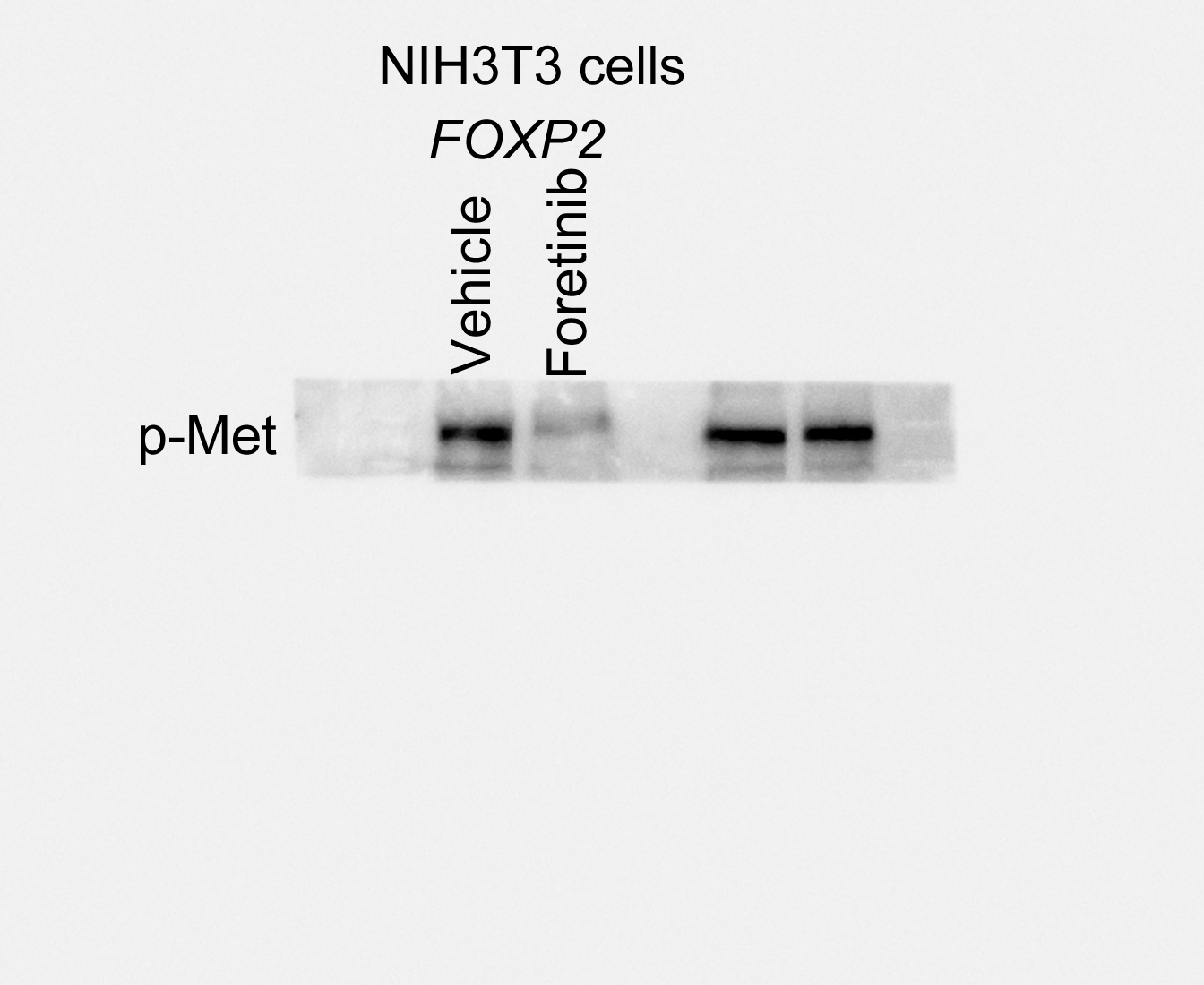

Supplement: Figure 3—source data 4. [file elife-81258-fig3-data4.zip › Figure 3-source data 4/Uncropped blots for Figure 3F in Main text/Figure 3F-source data 1.tif]

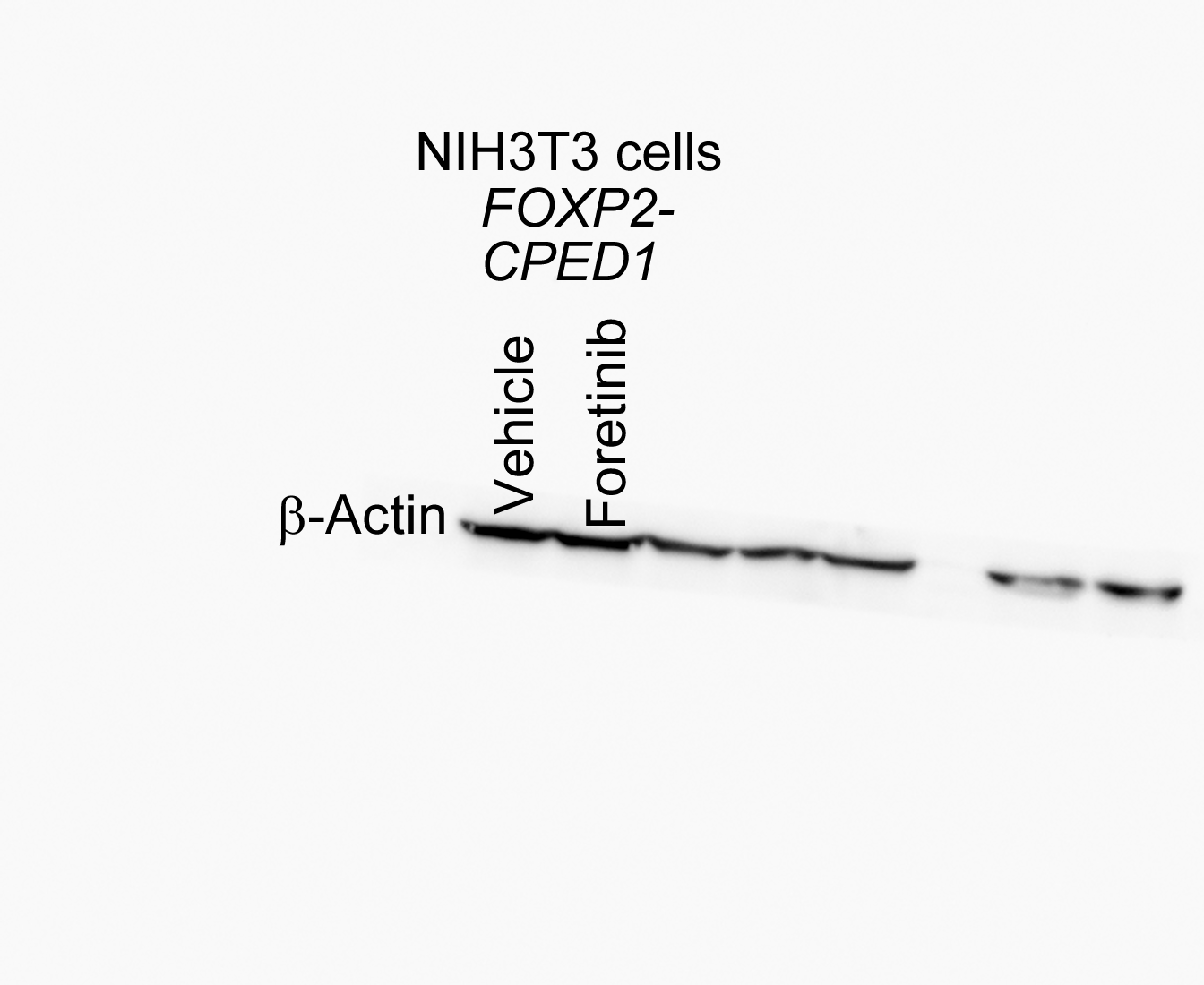

Supplement: Figure 3—source data 4. [file elife-81258-fig3-data4.zip › Figure 3-source data 4/Uncropped blots for Figure 3F in Main text/Figure 3F-source data 10.tif]

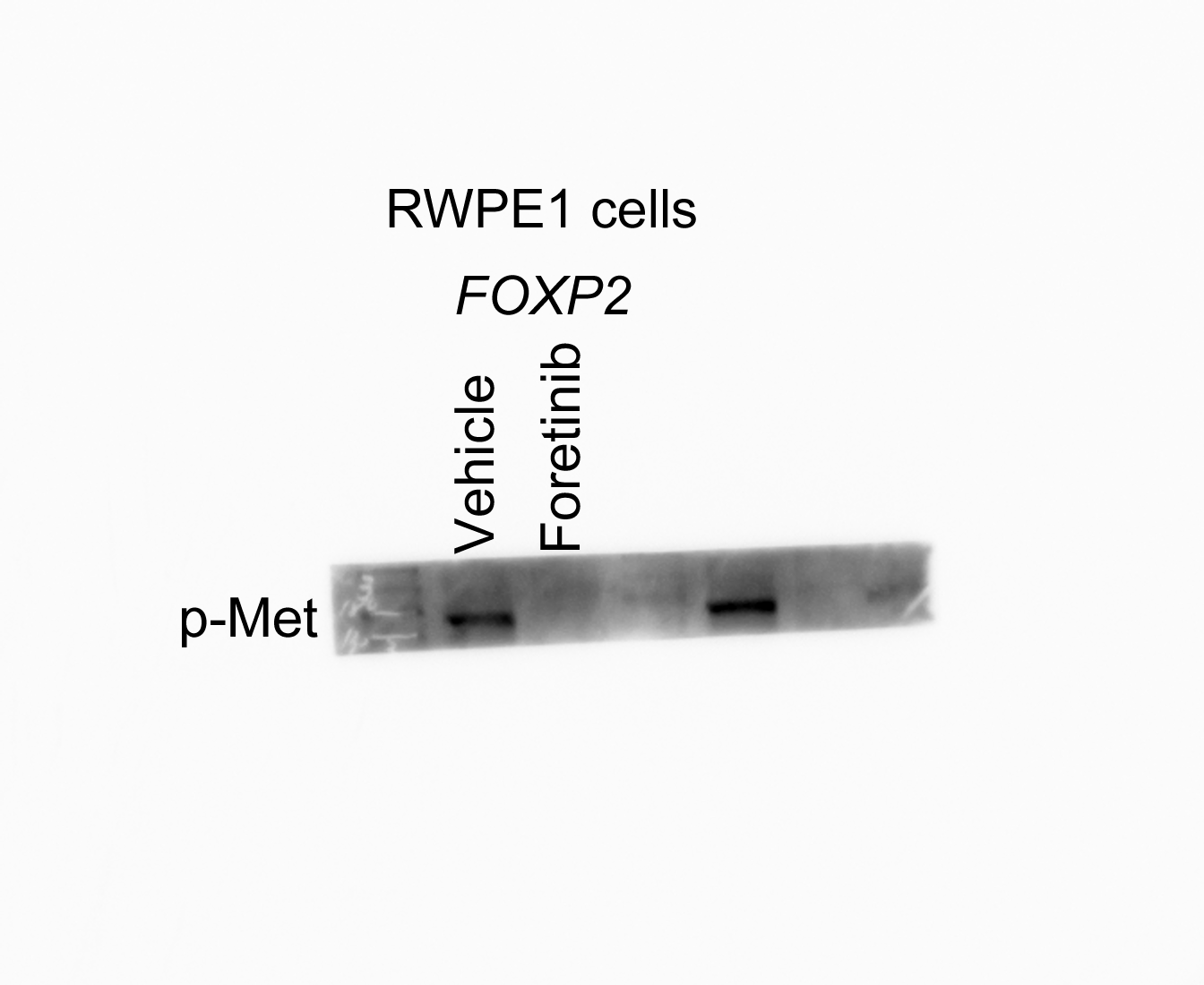

Supplement: Figure 3—source data 4. [file elife-81258-fig3-data4.zip › Figure 3-source data 4/Uncropped blots for Figure 3F in Main text/Figure 3F-source data 11.tif]

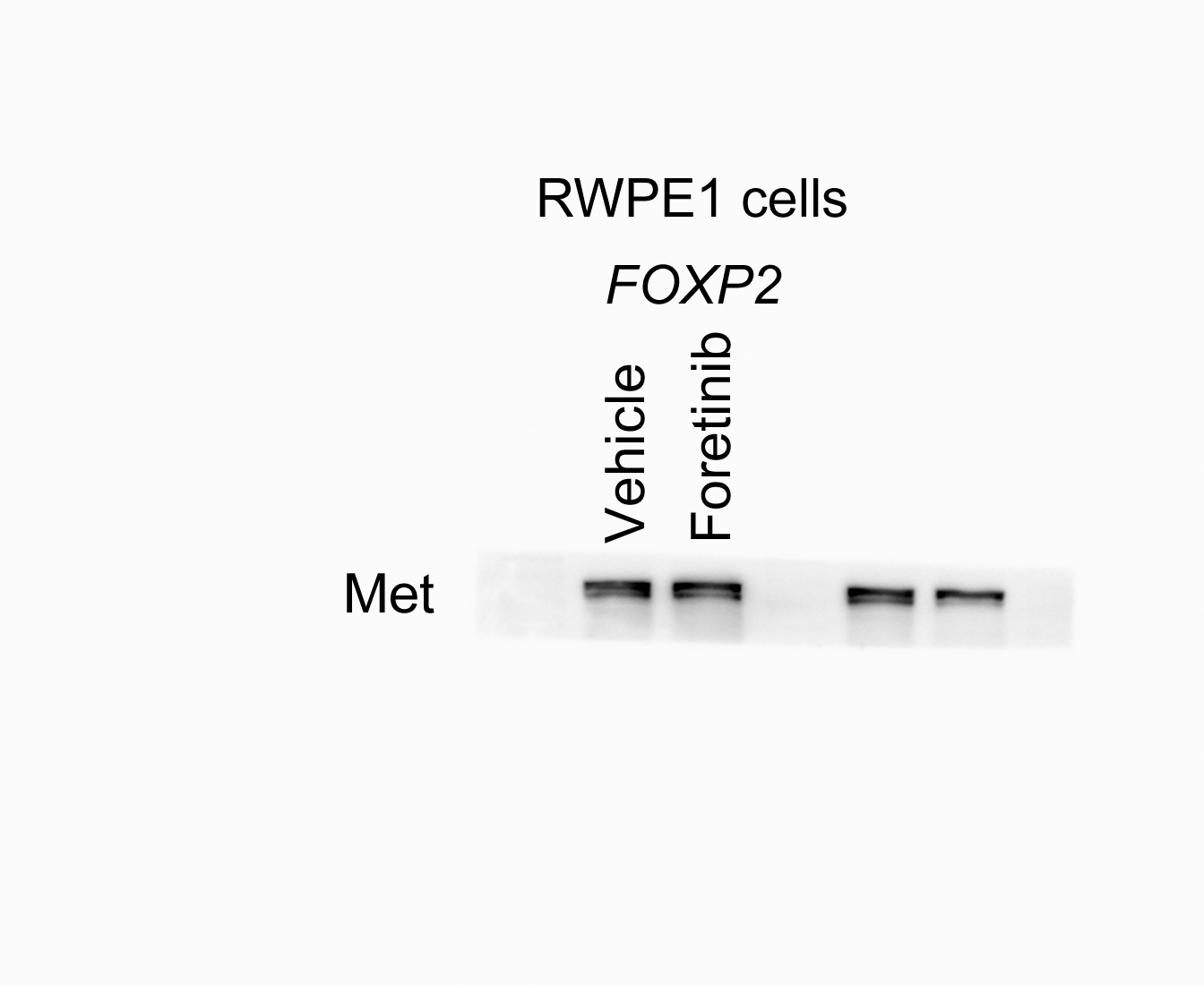

Supplement: Figure 3—source data 4. [file elife-81258-fig3-data4.zip › Figure 3-source data 4/Uncropped blots for Figure 3F in Main text/Figure 3F-source data 12.tif]

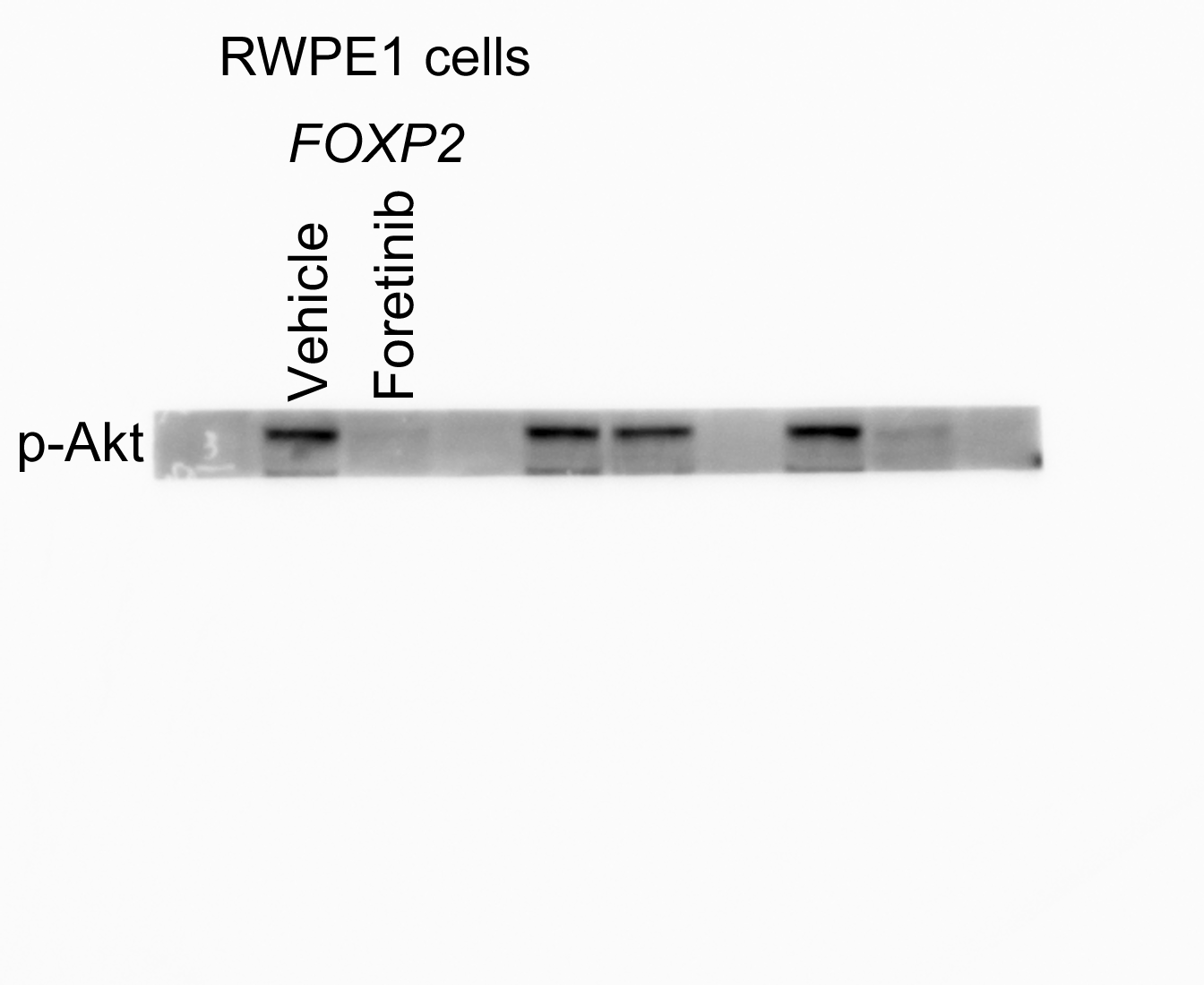

Supplement: Figure 3—source data 4. [file elife-81258-fig3-data4.zip › Figure 3-source data 4/Uncropped blots for Figure 3F in Main text/Figure 3F-source data 13.tif]

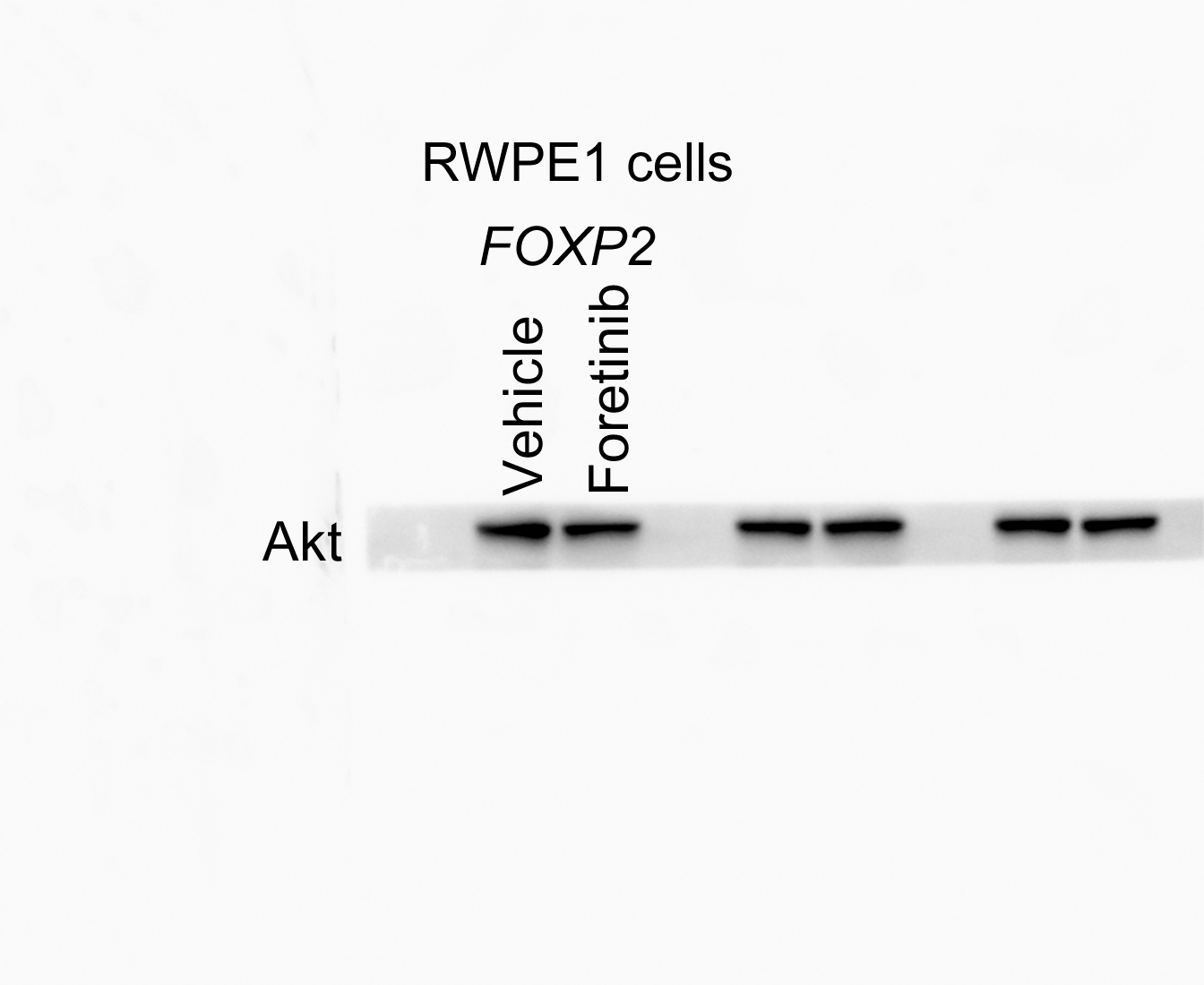

Supplement: Figure 3—source data 4. [file elife-81258-fig3-data4.zip › Figure 3-source data 4/Uncropped blots for Figure 3F in Main text/Figure 3F-source data 14.tif]

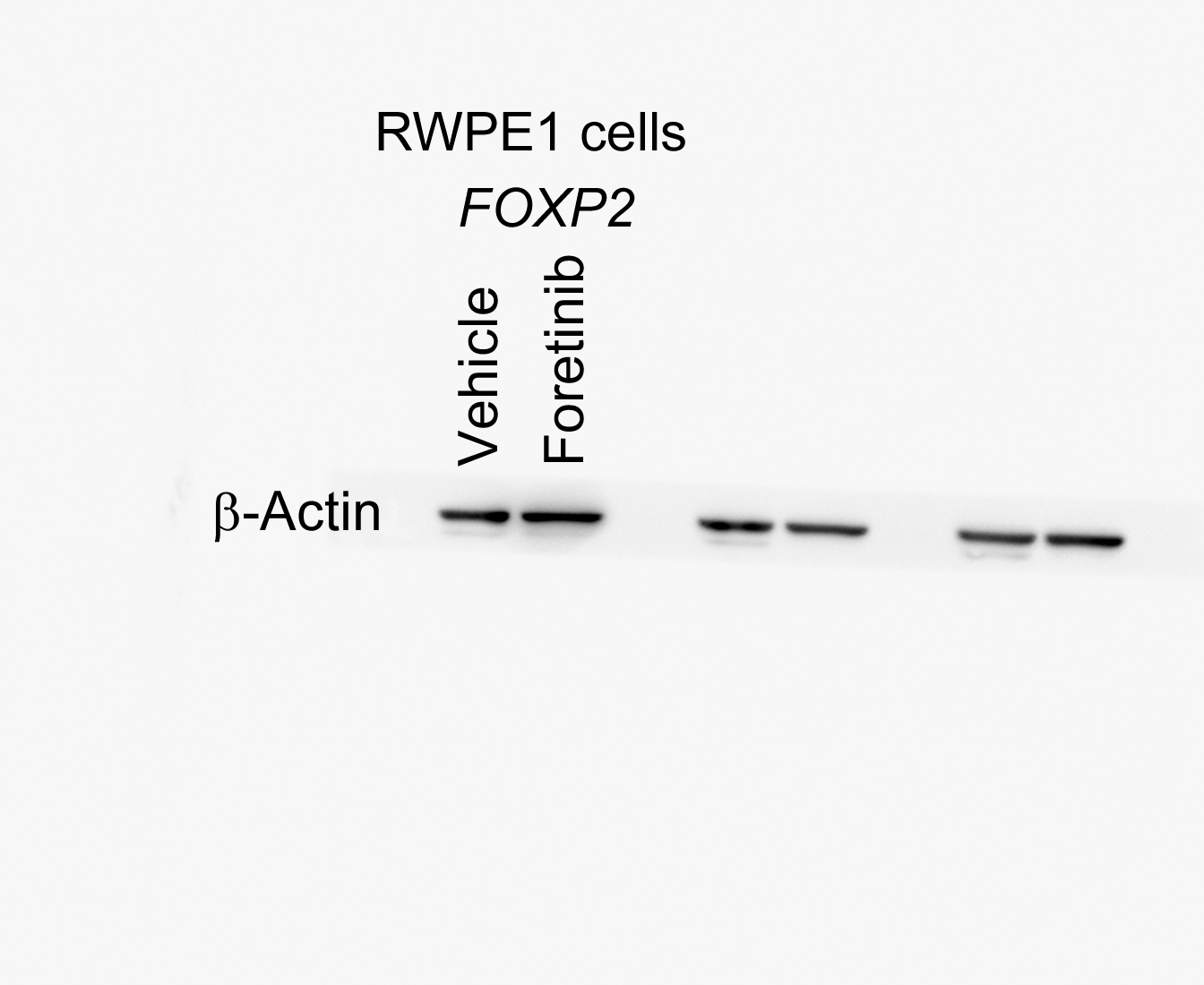

Supplement: Figure 3—source data 4. [file elife-81258-fig3-data4.zip › Figure 3-source data 4/Uncropped blots for Figure 3F in Main text/Figure 3F-source data 15.tif]

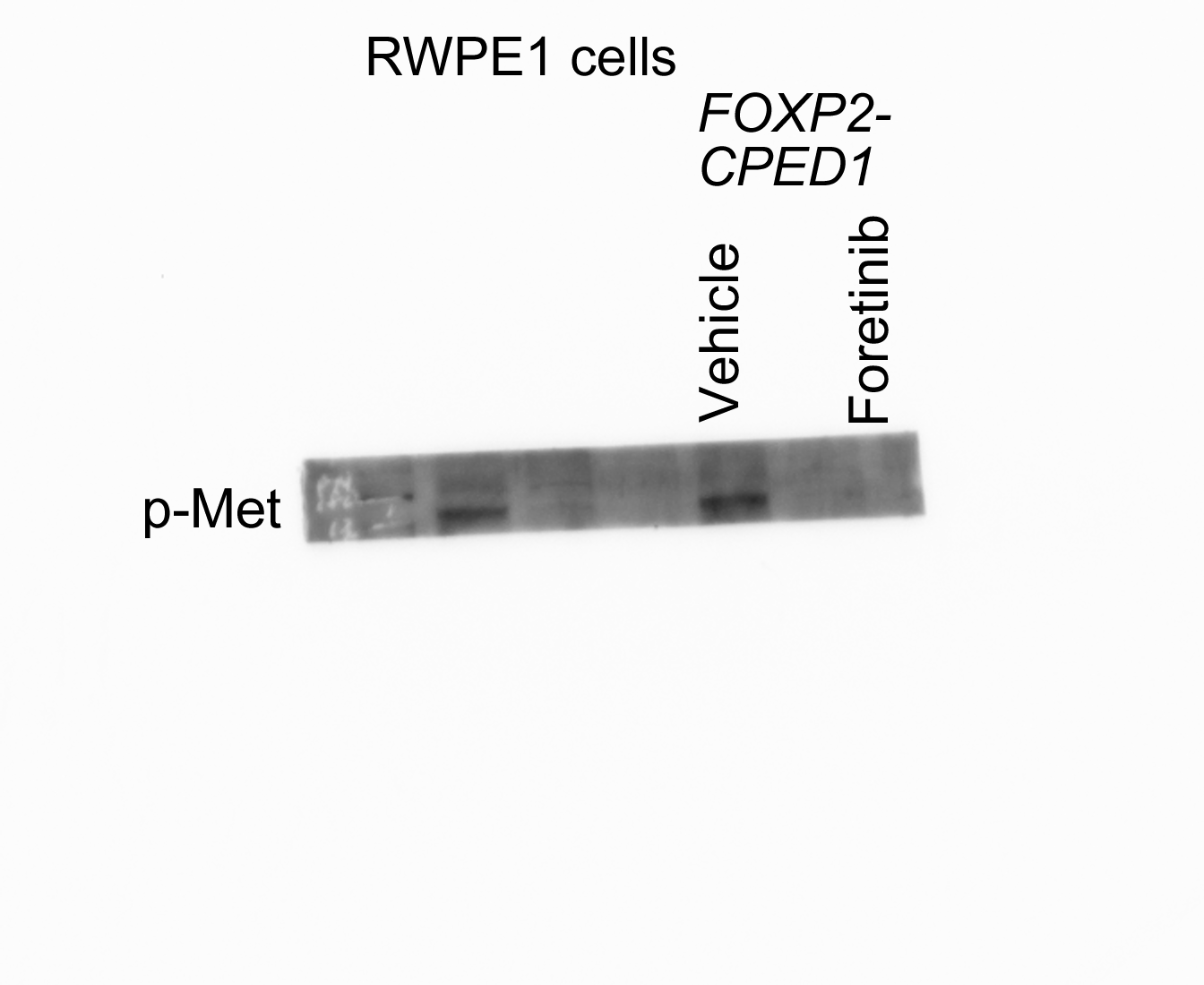

Supplement: Figure 3—source data 4. [file elife-81258-fig3-data4.zip › Figure 3-source data 4/Uncropped blots for Figure 3F in Main text/Figure 3F-source data 16.tif]

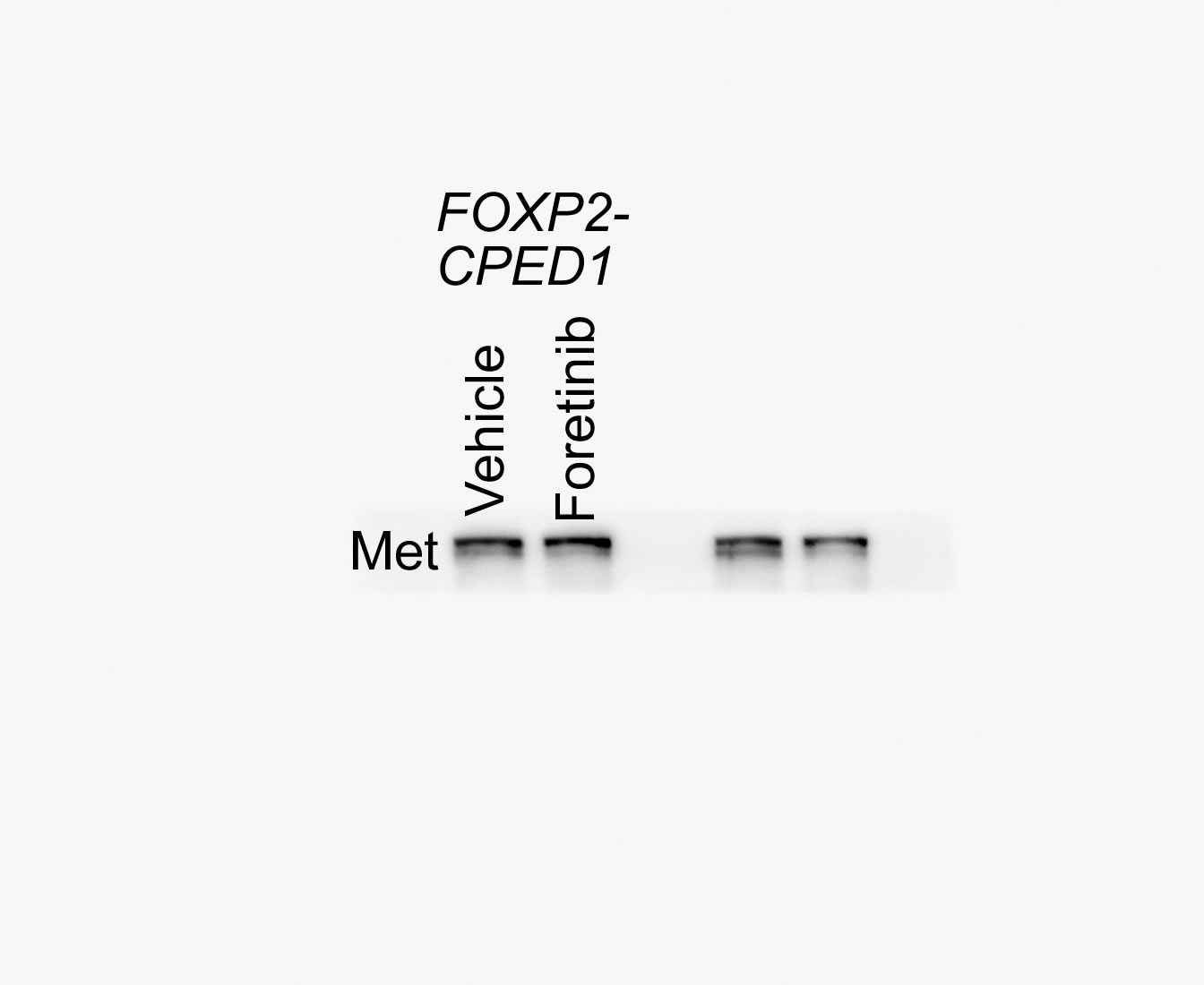

Supplement: Figure 3—source data 4. [file elife-81258-fig3-data4.zip › Figure 3-source data 4/Uncropped blots for Figure 3F in Main text/Figure 3F-source data 17.jpg]

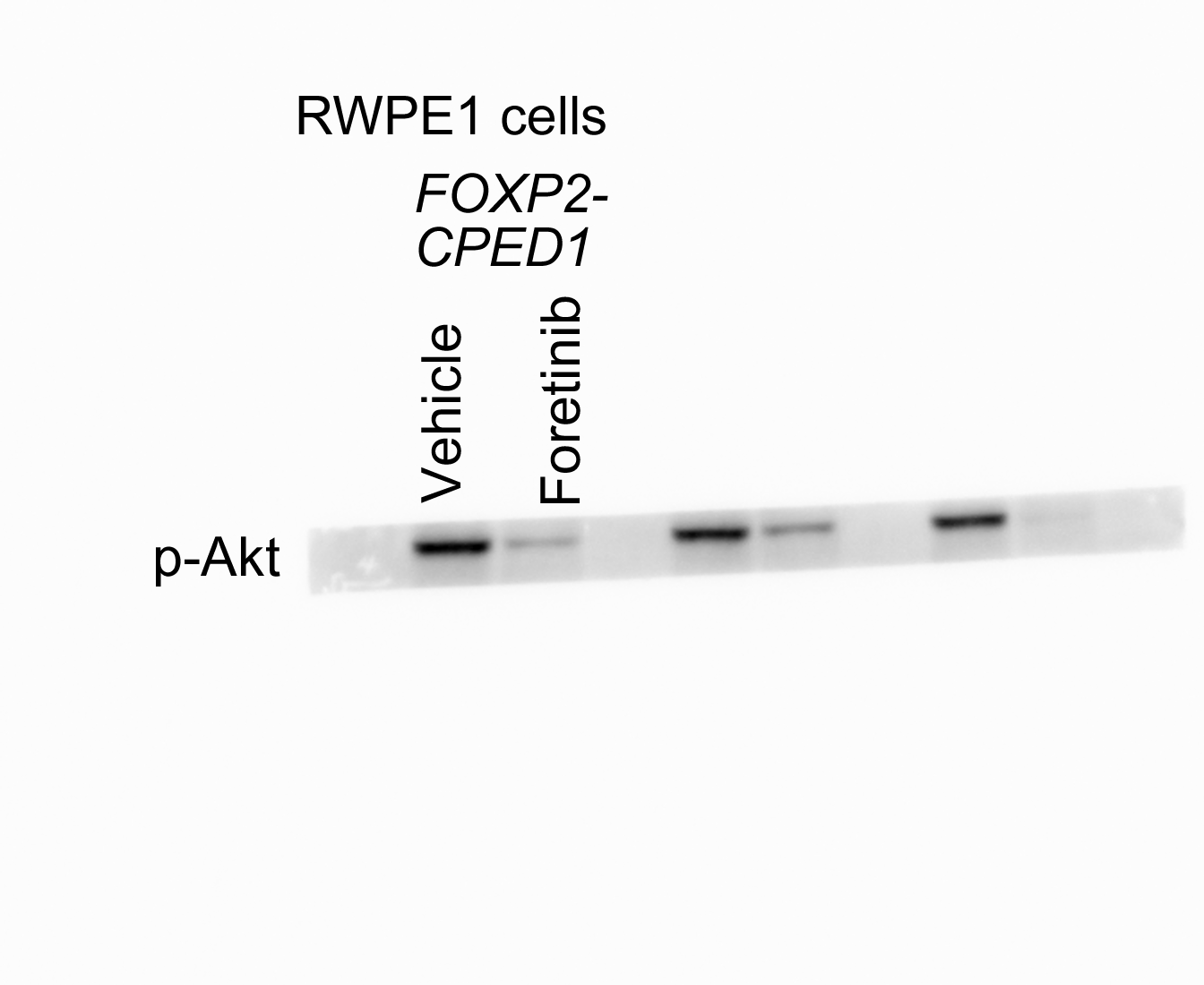

Supplement: Figure 3—source data 4. [file elife-81258-fig3-data4.zip › Figure 3-source data 4/Uncropped blots for Figure 3F in Main text/Figure 3F-source data 18.tif]

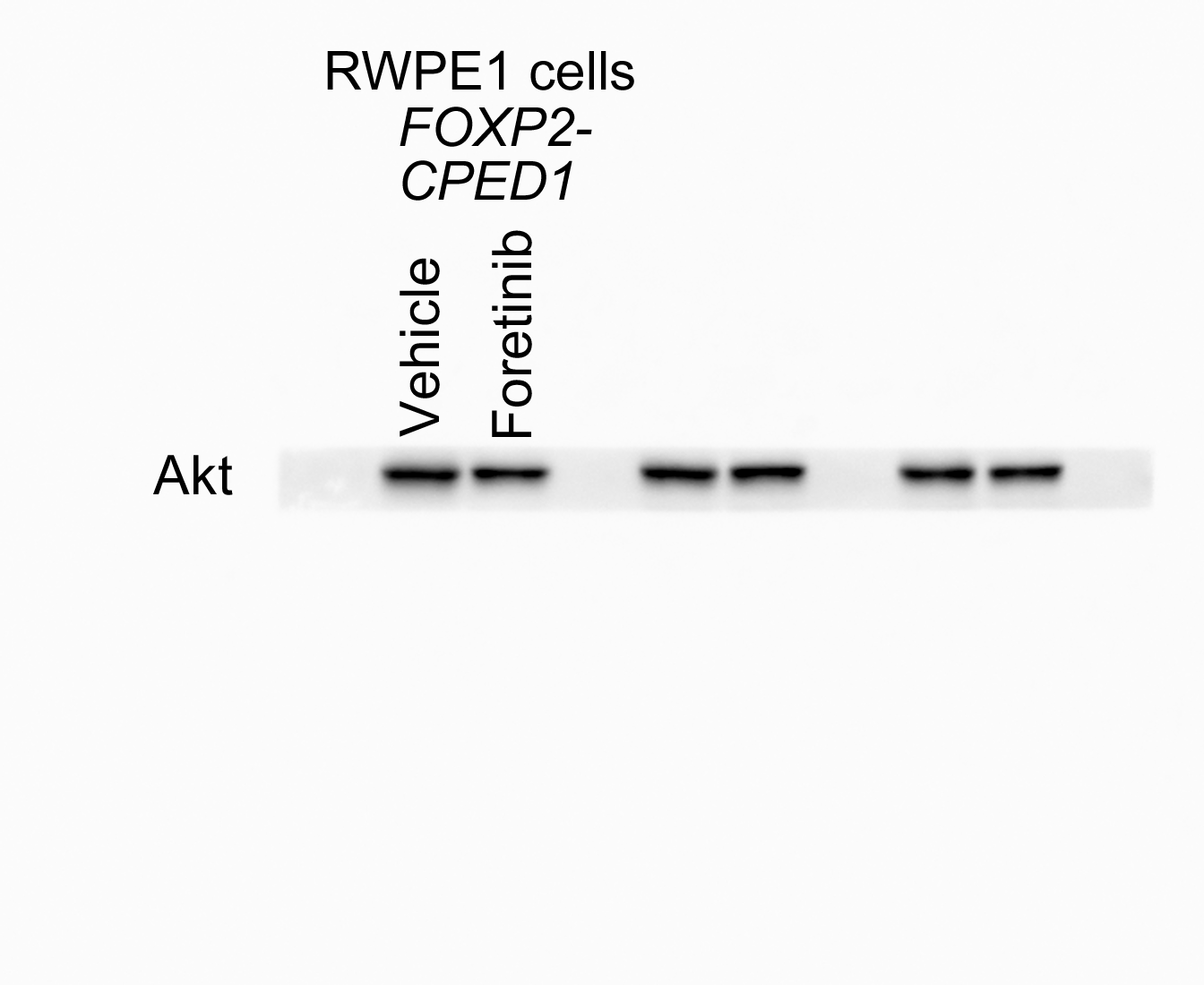

Supplement: Figure 3—source data 4. [file elife-81258-fig3-data4.zip › Figure 3-source data 4/Uncropped blots for Figure 3F in Main text/Figure 3F-source data 19.tif]

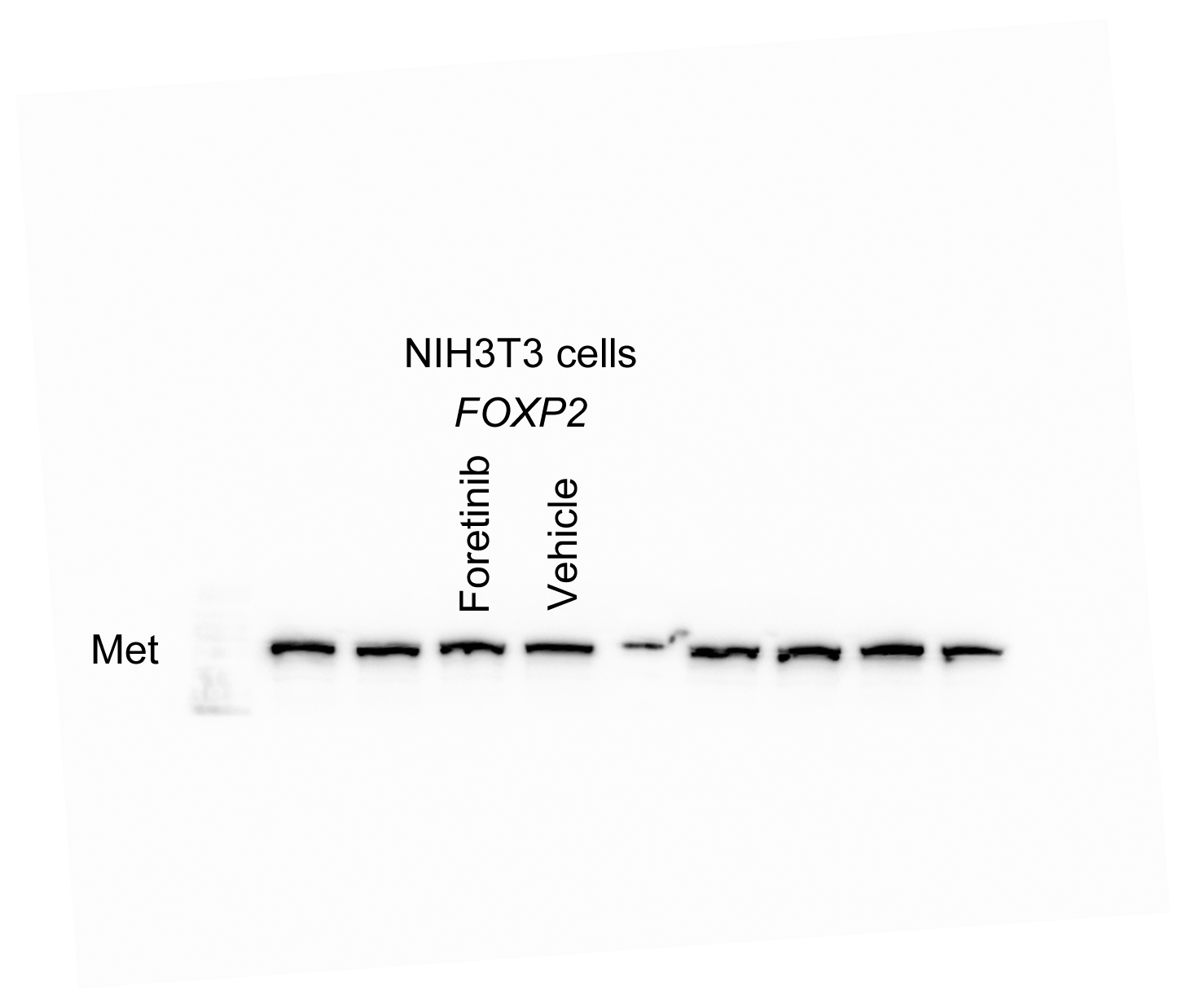

Supplement: Figure 3—source data 4. [file elife-81258-fig3-data4.zip › Figure 3-source data 4/Uncropped blots for Figure 3F in Main text/Figure 3F-source data 2.tif]

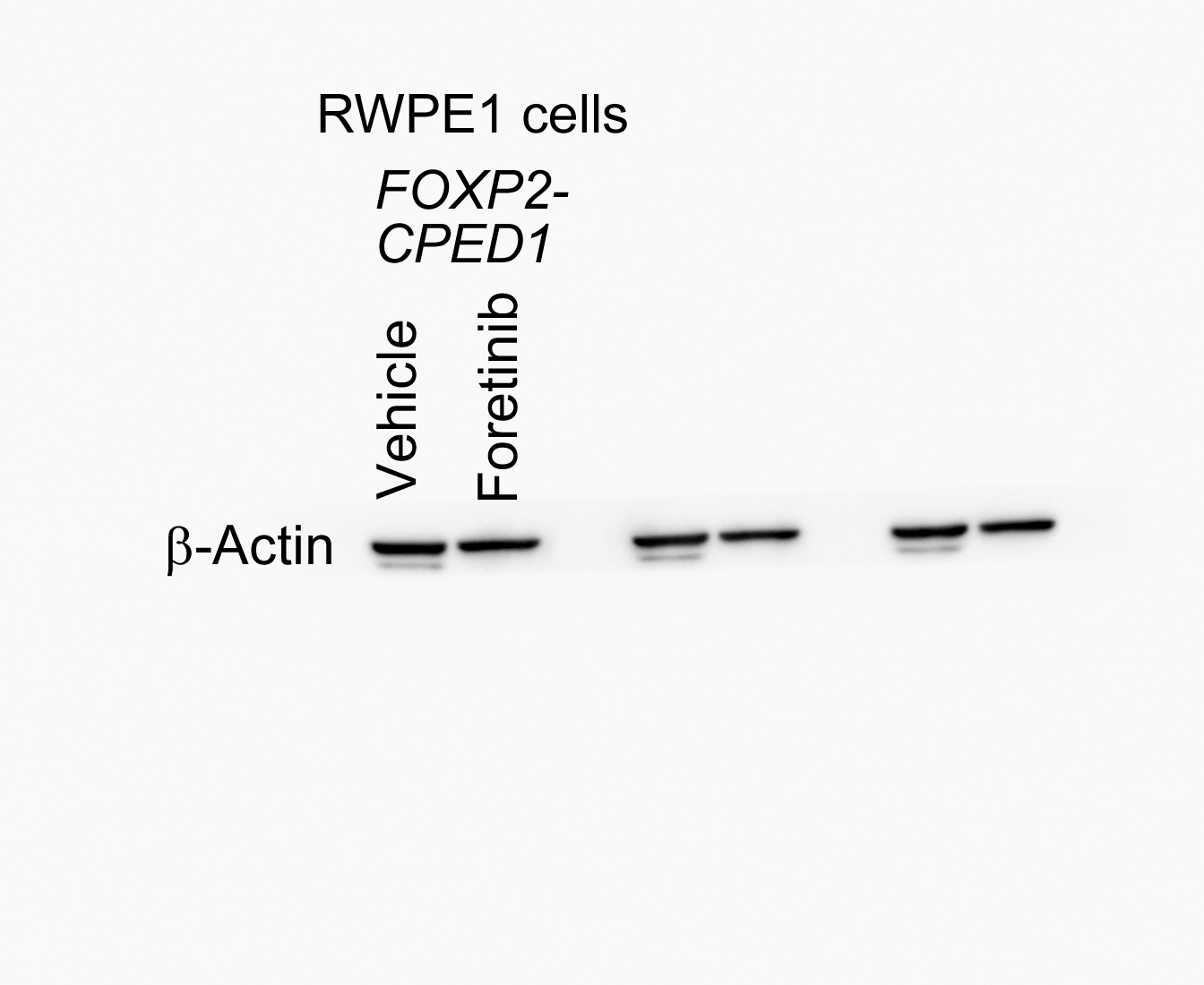

Supplement: Figure 3—source data 4. [file elife-81258-fig3-data4.zip › Figure 3-source data 4/Uncropped blots for Figure 3F in Main text/Figure 3F-source data 20.tif]

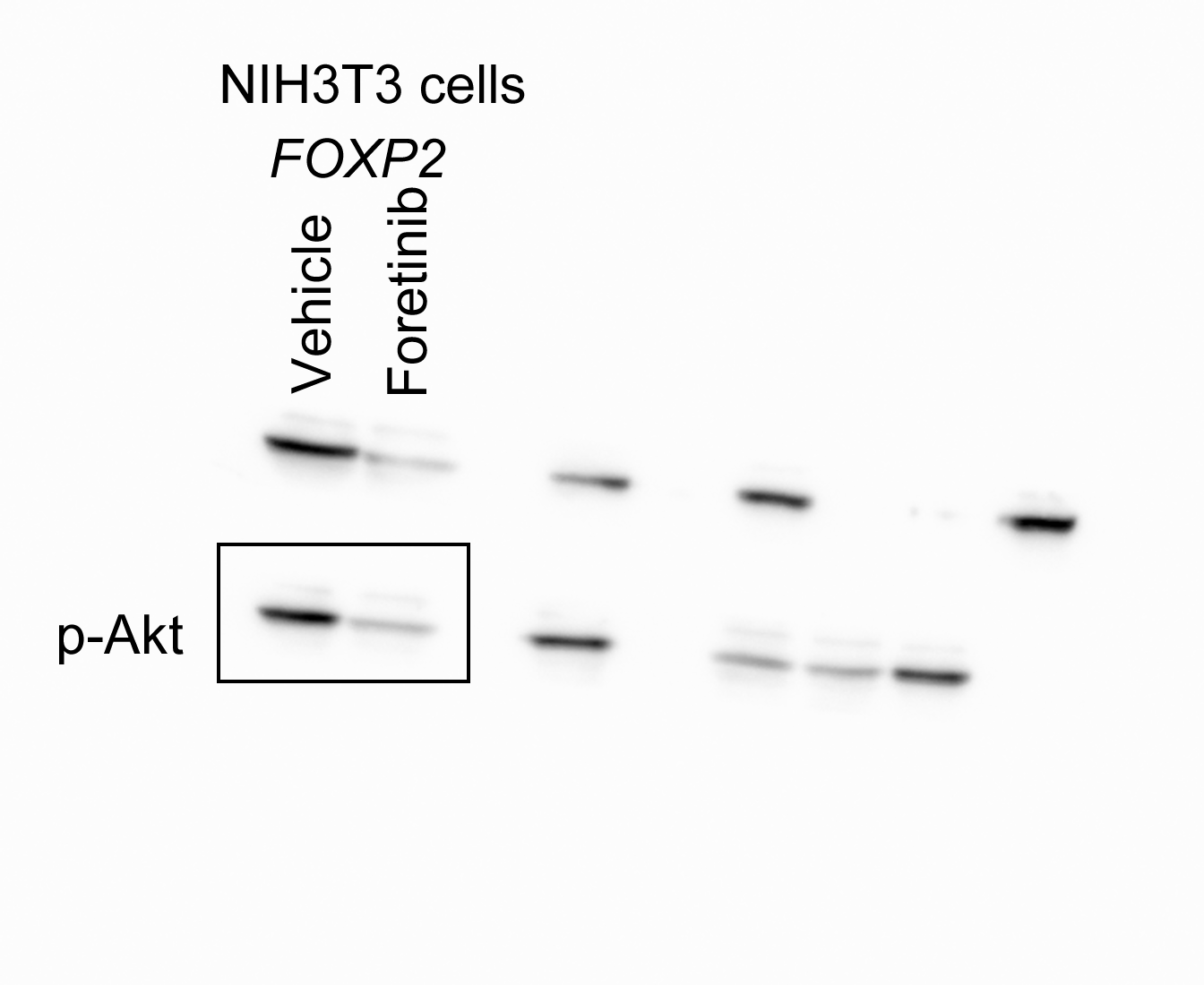

Supplement: Figure 3—source data 4. [file elife-81258-fig3-data4.zip › Figure 3-source data 4/Uncropped blots for Figure 3F in Main text/Figure 3F-source data 3.tif]

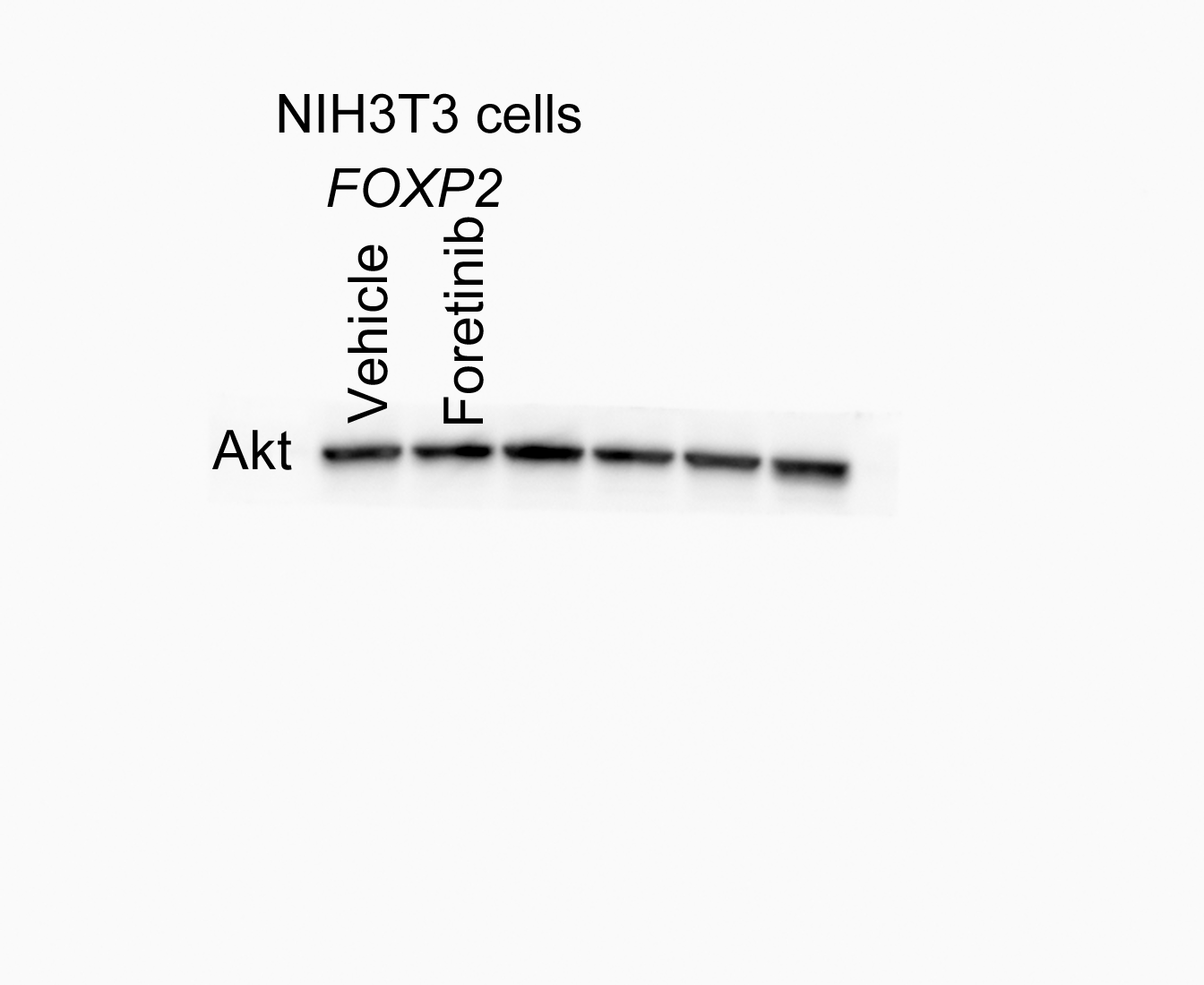

Supplement: Figure 3—source data 4. [file elife-81258-fig3-data4.zip › Figure 3-source data 4/Uncropped blots for Figure 3F in Main text/Figure 3F-source data 4.tif]
