# Supplementary figures and images for: FOXP2 confers oncogenic effects in prostate cancer (part 2 of 2)
Source: eLife. 2023 Sep 5;12:e81258. doi: 10.7554/eLife.81258 (PMC10513481; doi:10.7554/eLife.81258)

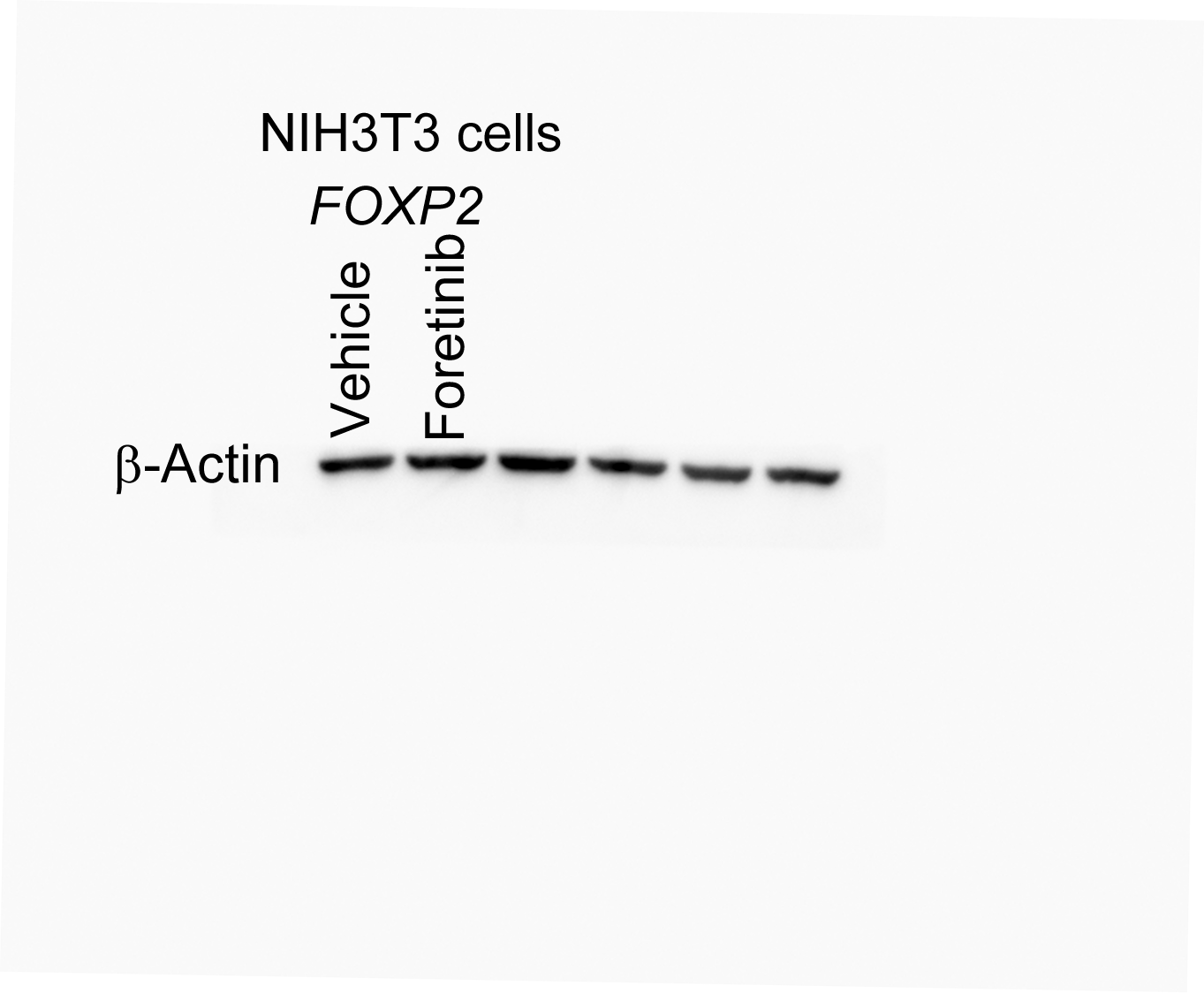

Supplement: Figure 3—source data 4. [file elife-81258-fig3-data4.zip › Figure 3-source data 4/Uncropped blots for Figure 3F in Main text/Figure 3F-source data 5.tif]

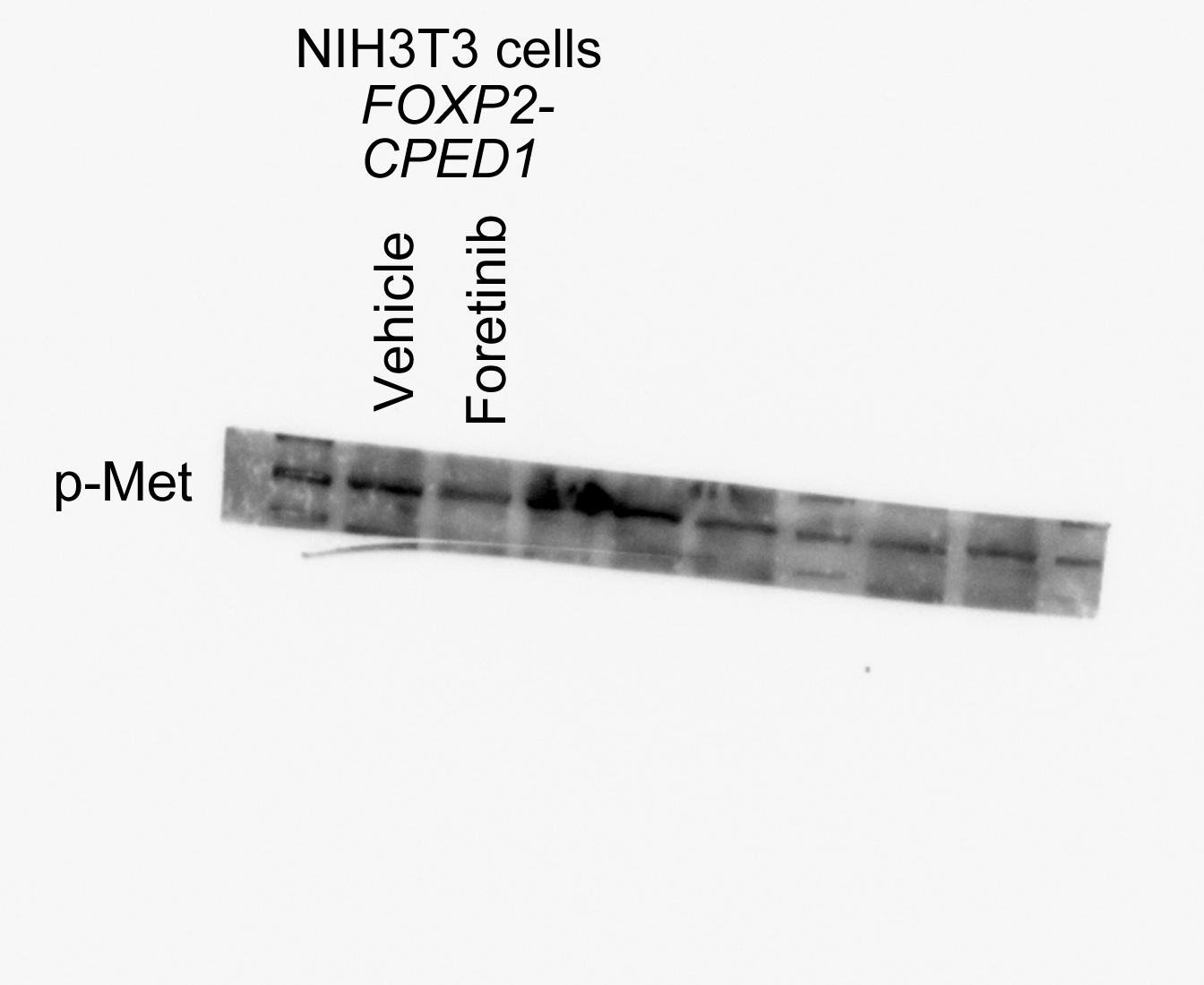

Supplement: Figure 3—source data 4. [file elife-81258-fig3-data4.zip › Figure 3-source data 4/Uncropped blots for Figure 3F in Main text/Figure 3F-source data 6.tif]

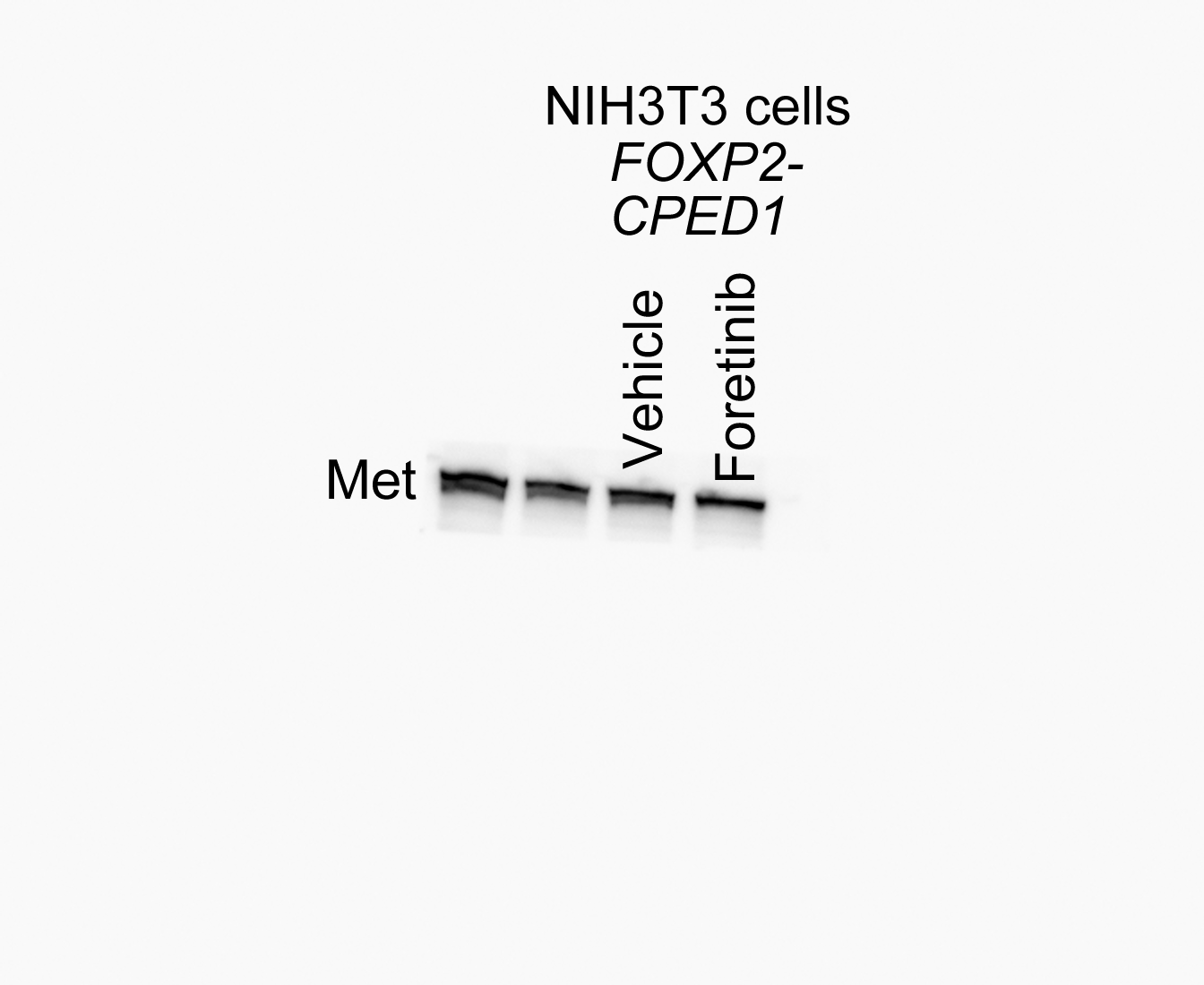

Supplement: Figure 3—source data 4. [file elife-81258-fig3-data4.zip › Figure 3-source data 4/Uncropped blots for Figure 3F in Main text/Figure 3F-source data 7.tif]

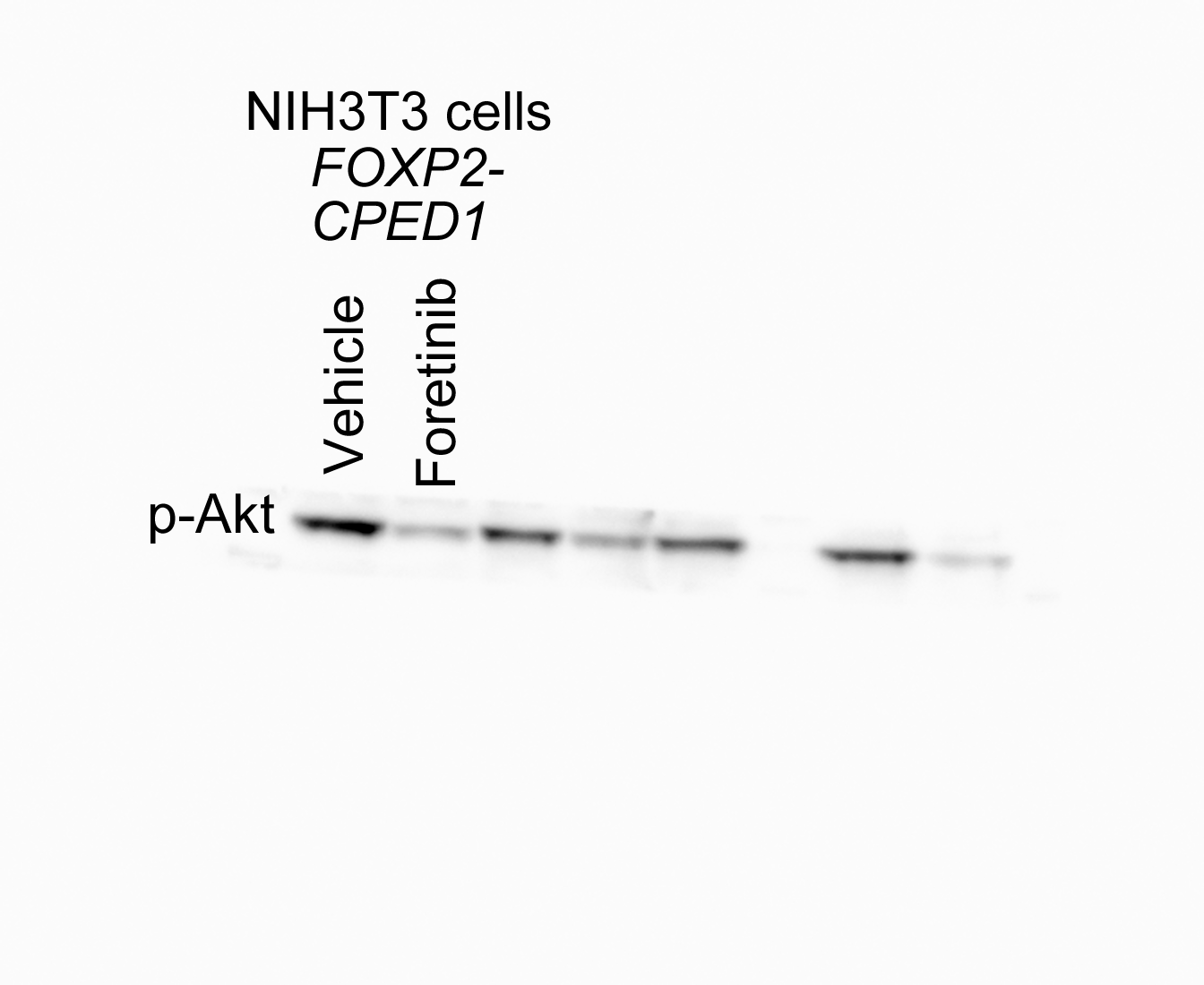

Supplement: Figure 3—source data 4. [file elife-81258-fig3-data4.zip › Figure 3-source data 4/Uncropped blots for Figure 3F in Main text/Figure 3F-source data 8.tif]

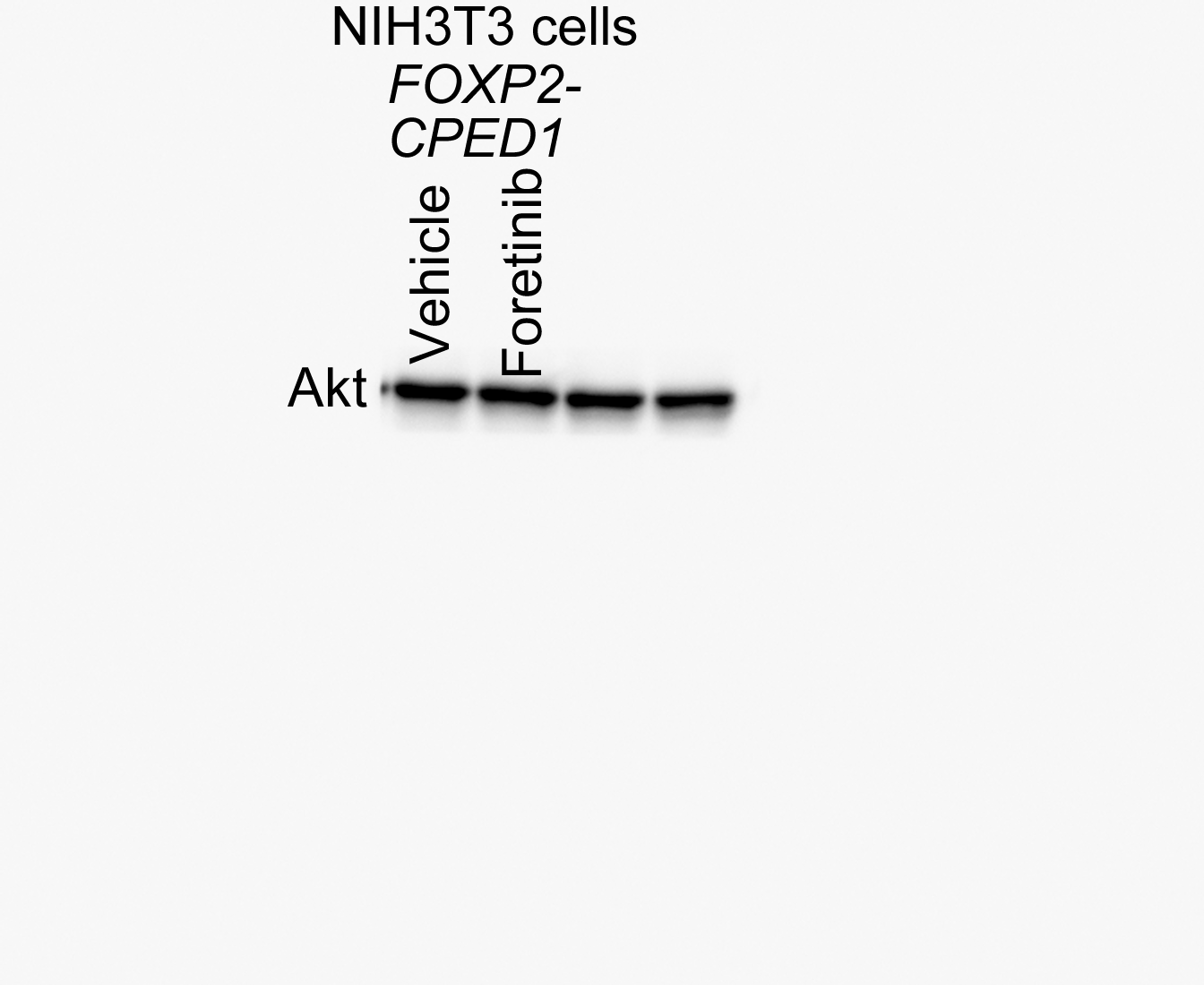

Supplement: Figure 3—source data 4. [file elife-81258-fig3-data4.zip › Figure 3-source data 4/Uncropped blots for Figure 3F in Main text/Figure 3F-source data 9.tif]

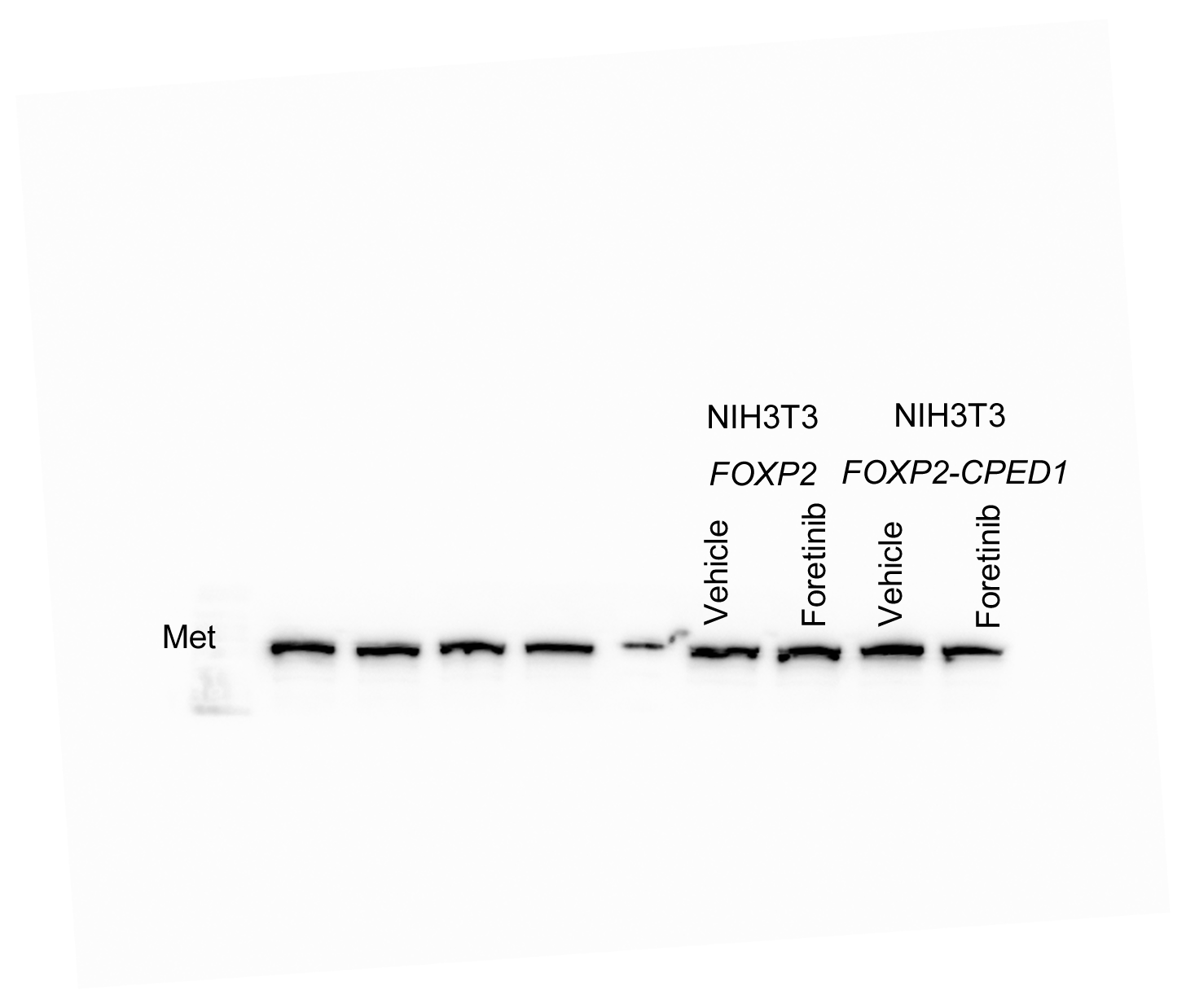

Supplement: Figure 3—source data 4. [file elife-81258-fig3-data4.zip › Figure 3-source data 4/Uncropped blots for Figure 3F NIH3T3 cells repeat/repeat/Total-MET FOXP2 FOXP2_CPED1.tif]

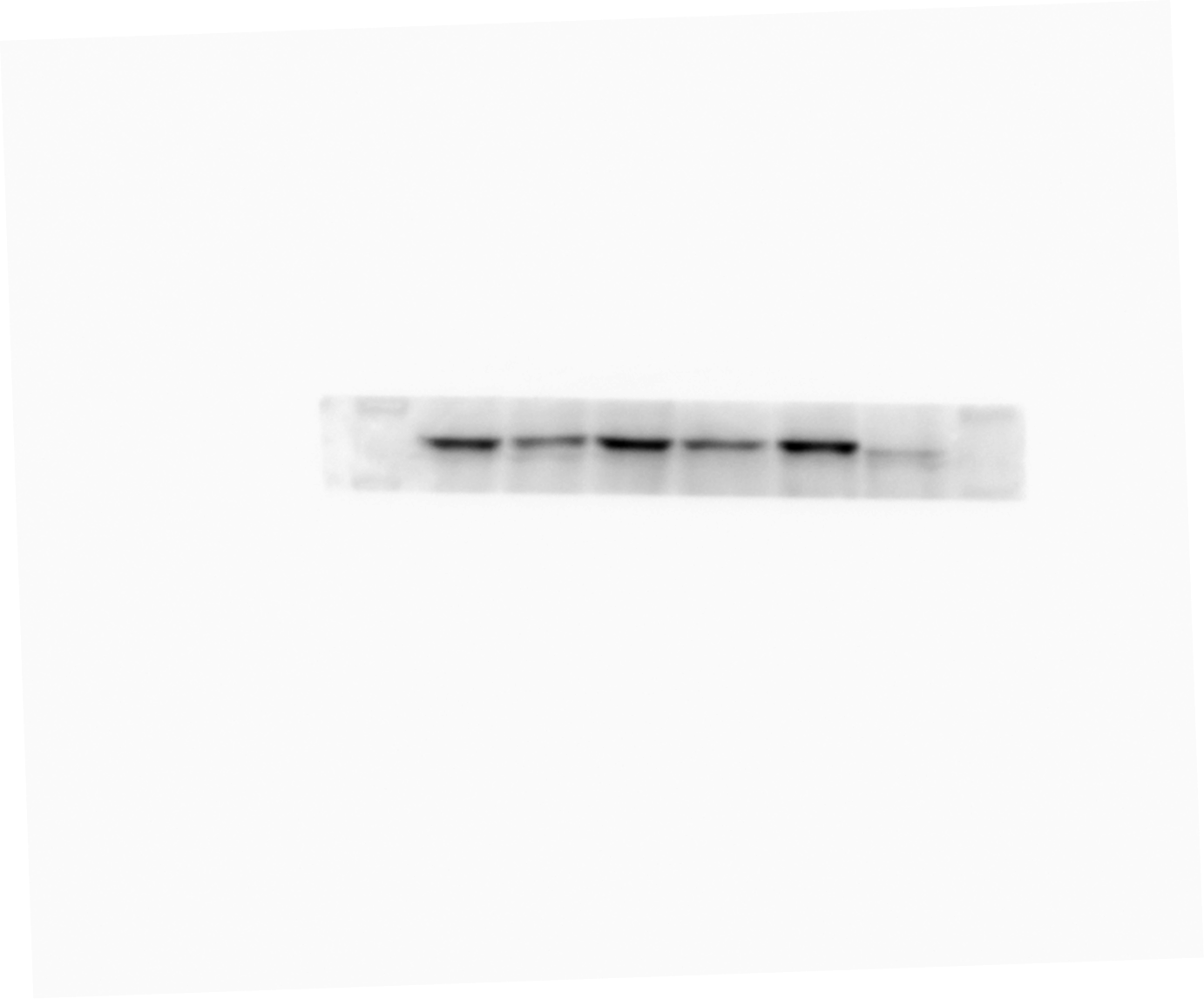

Supplement: Figure 3—source data 5. [file elife-81258-fig3-data5.zip › Figure 3-source data 5/Original files for Figure 3G NIH3T3 cells/Original files for Figure 3G NIH3T3 cells in Main text/1-P-AKT.tif]

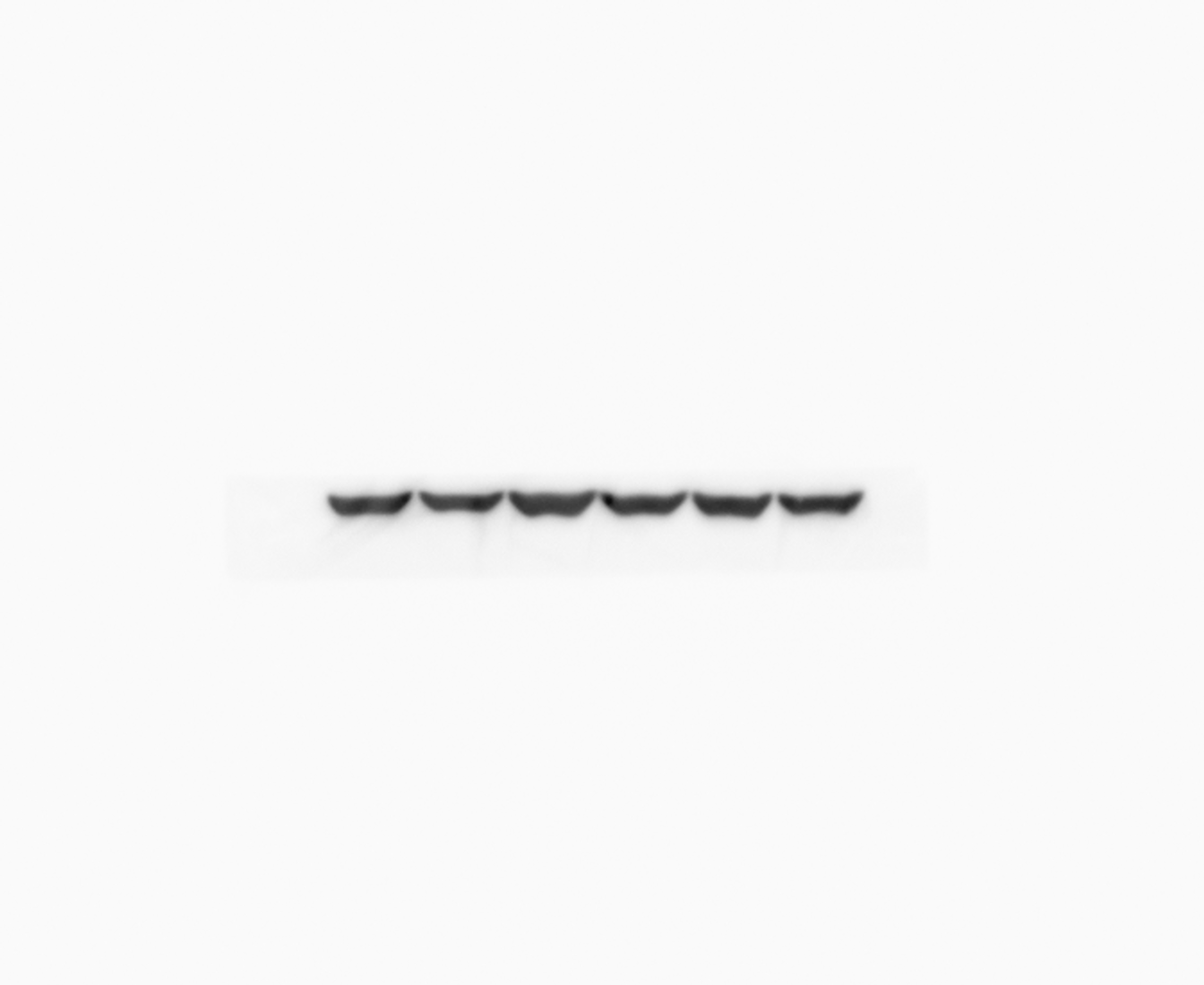

Supplement: Figure 3—source data 5. [file elife-81258-fig3-data5.zip › Figure 3-source data 5/Original files for Figure 3G NIH3T3 cells/Original files for Figure 3G NIH3T3 cells in Main text/4-ACTIN.tif]

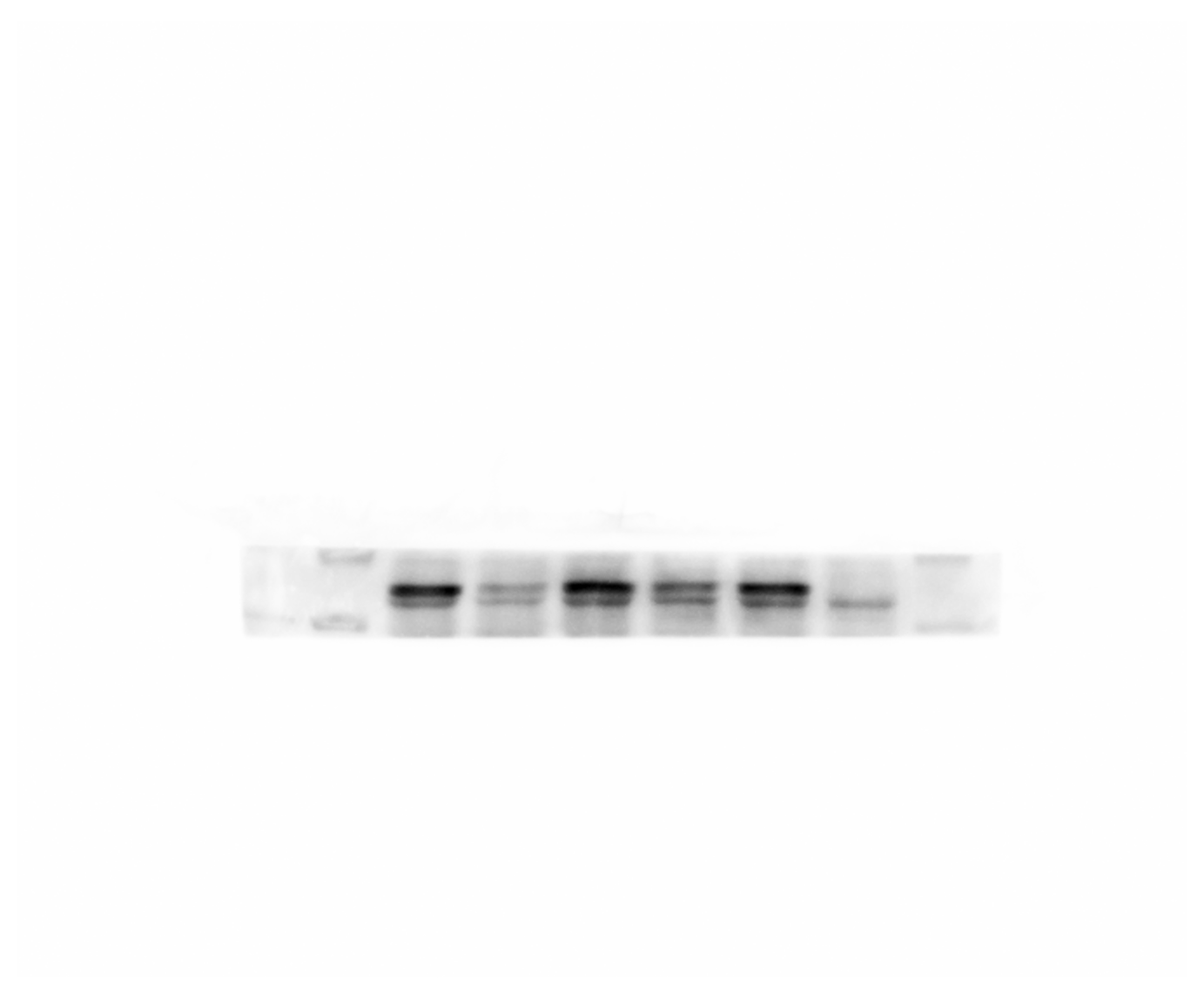

Supplement: Figure 3—source data 5. [file elife-81258-fig3-data5.zip › Figure 3-source data 5/Original files for Figure 3G NIH3T3 cells/Original files for Figure 3G NIH3T3 cells Repeat/P-AKT.tif]

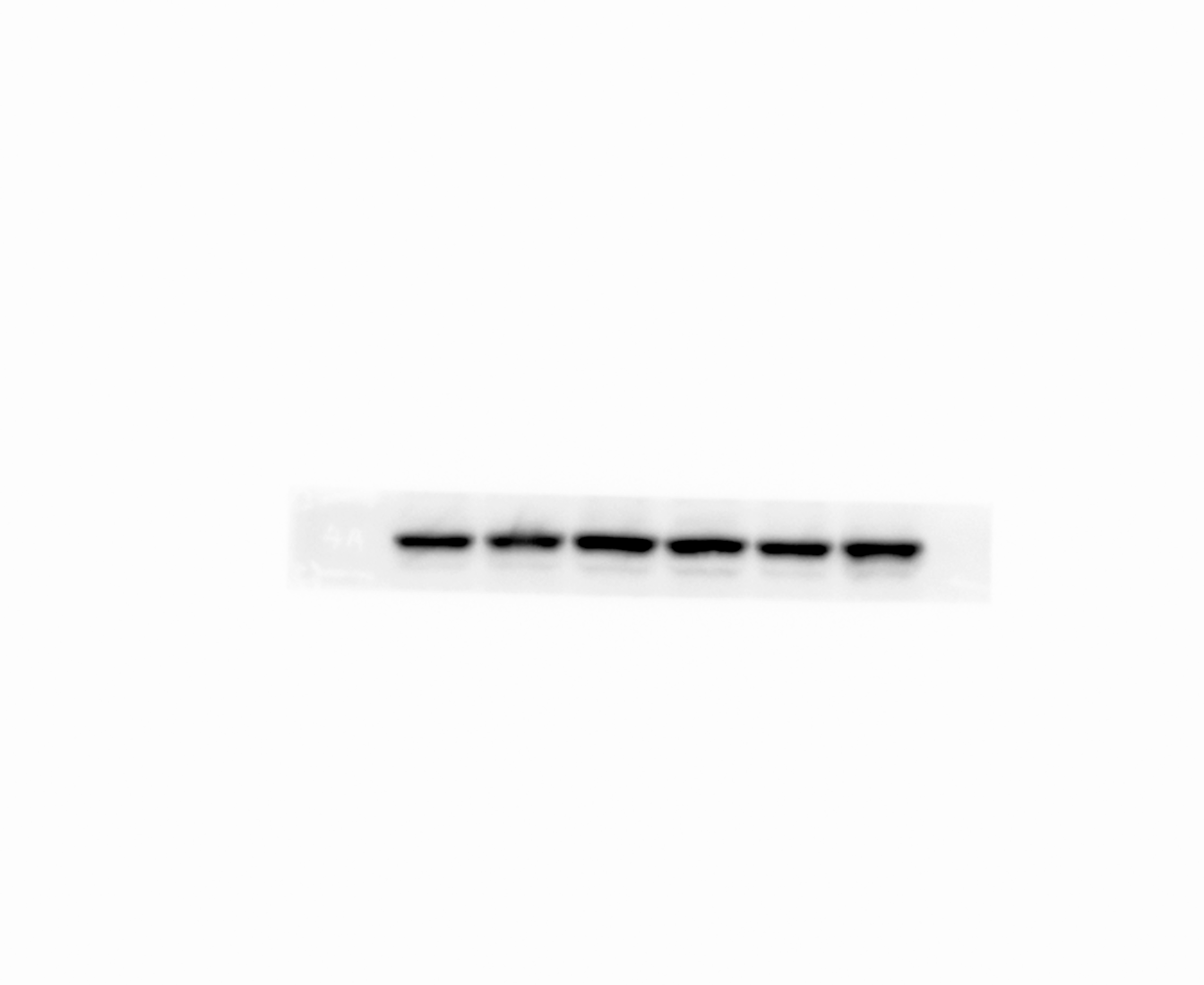

Supplement: Figure 3—source data 5. [file elife-81258-fig3-data5.zip › Figure 3-source data 5/Original files for Figure 3G NIH3T3 cells/Original files for Figure 3G NIH3T3 cells Repeat/T-AKT.tif]

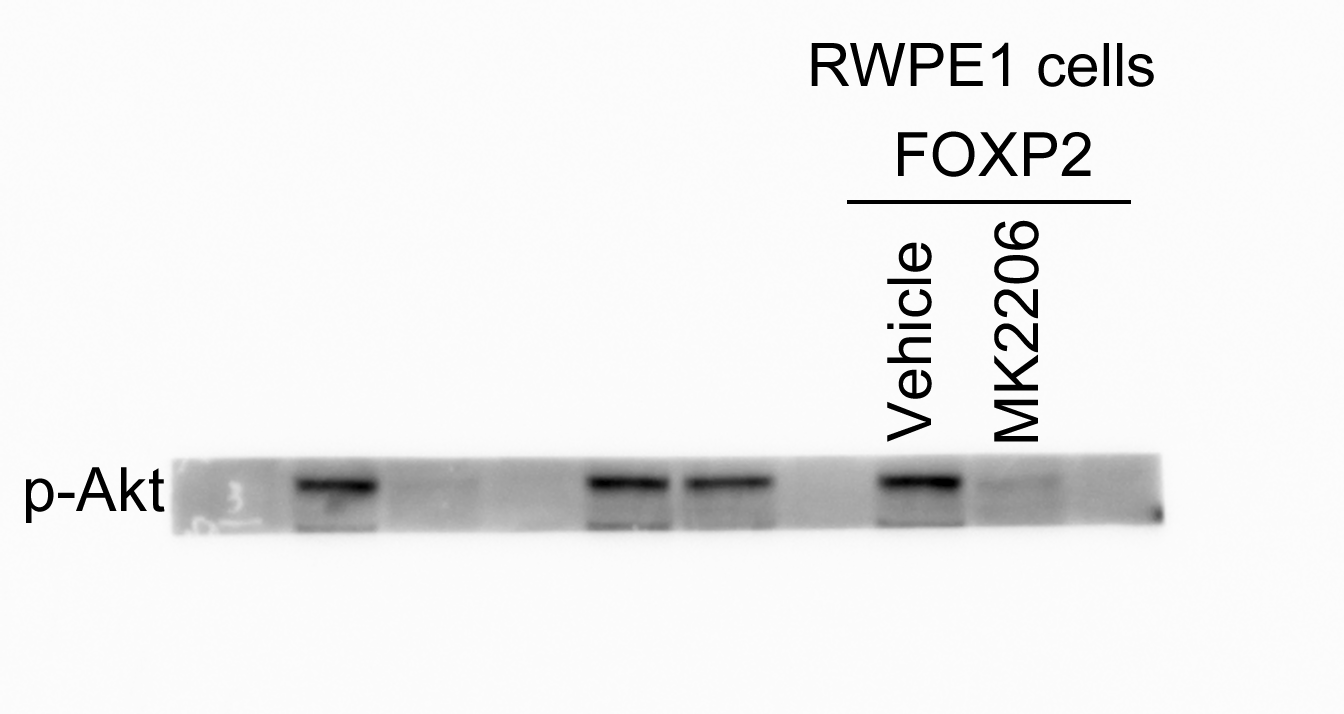

Supplement: Figure 3—source data 5. [file elife-81258-fig3-data5.zip › Figure 3-source data 5/Uncropped blots for Figure 3G in Main text/Figure 3G-source data 1.tif]

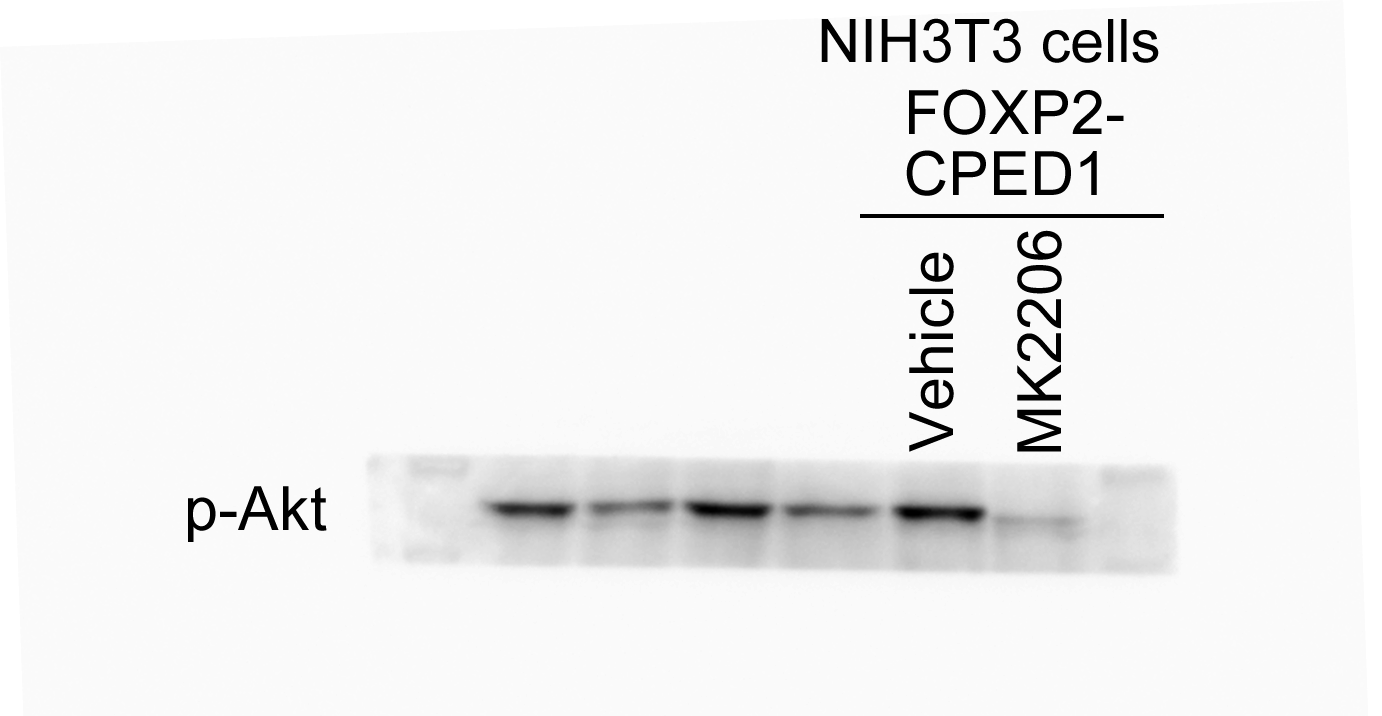

Supplement: Figure 3—source data 5. [file elife-81258-fig3-data5.zip › Figure 3-source data 5/Uncropped blots for Figure 3G in Main text/Figure 3G-source data 10.tif]

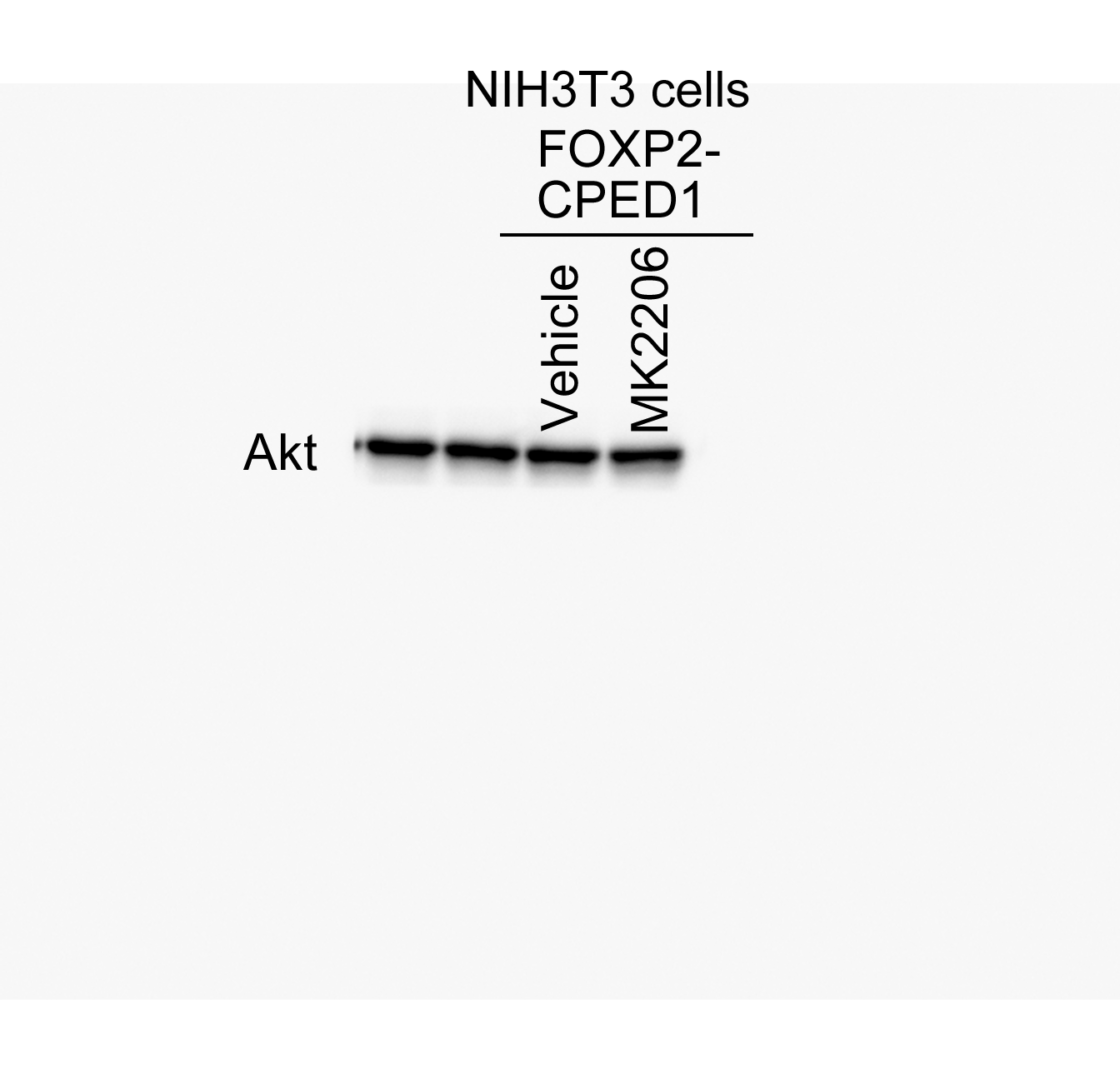

Supplement: Figure 3—source data 5. [file elife-81258-fig3-data5.zip › Figure 3-source data 5/Uncropped blots for Figure 3G in Main text/Figure 3G-source data 11.tif]

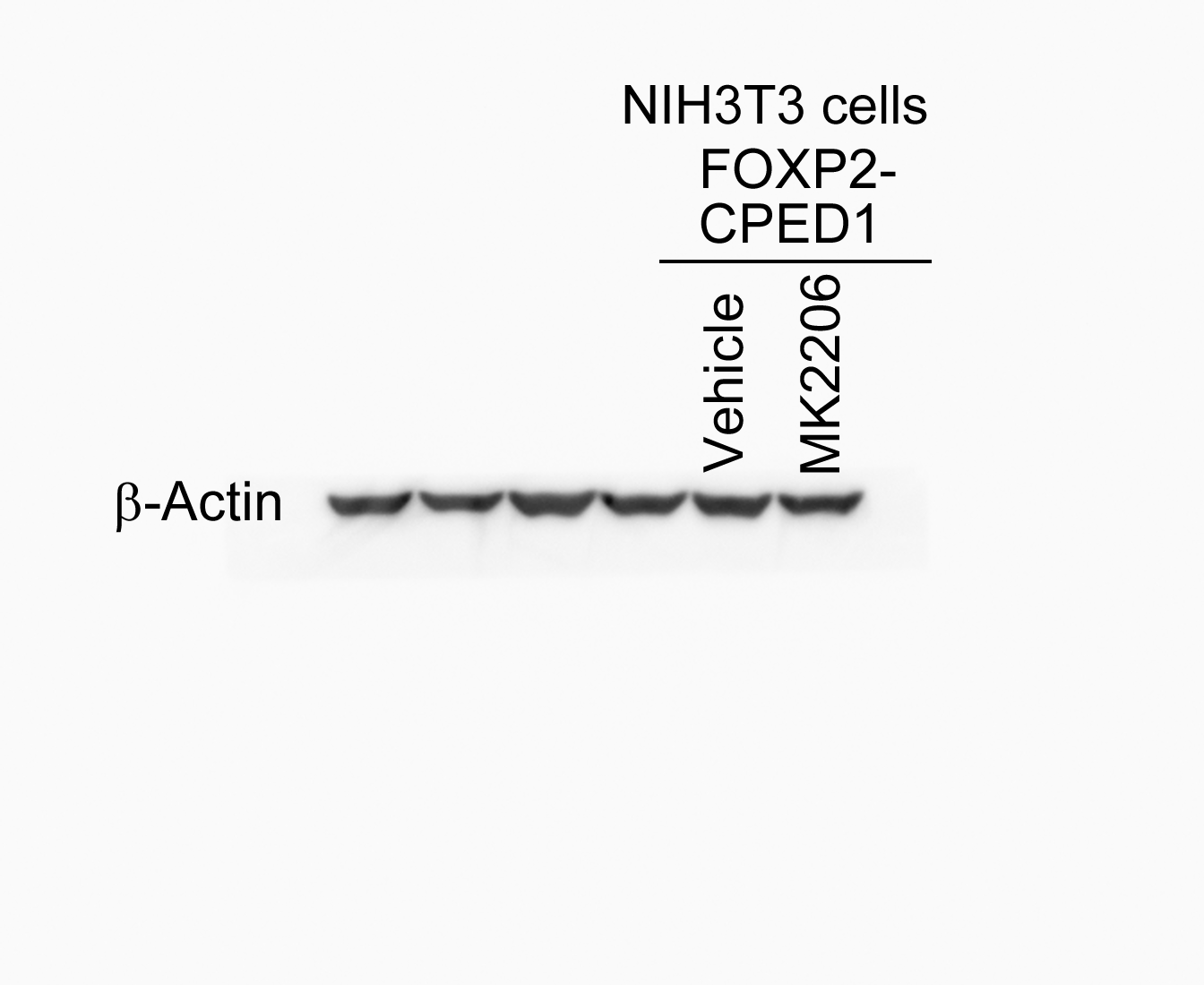

Supplement: Figure 3—source data 5. [file elife-81258-fig3-data5.zip › Figure 3-source data 5/Uncropped blots for Figure 3G in Main text/Figure 3G-source data 12.tif]

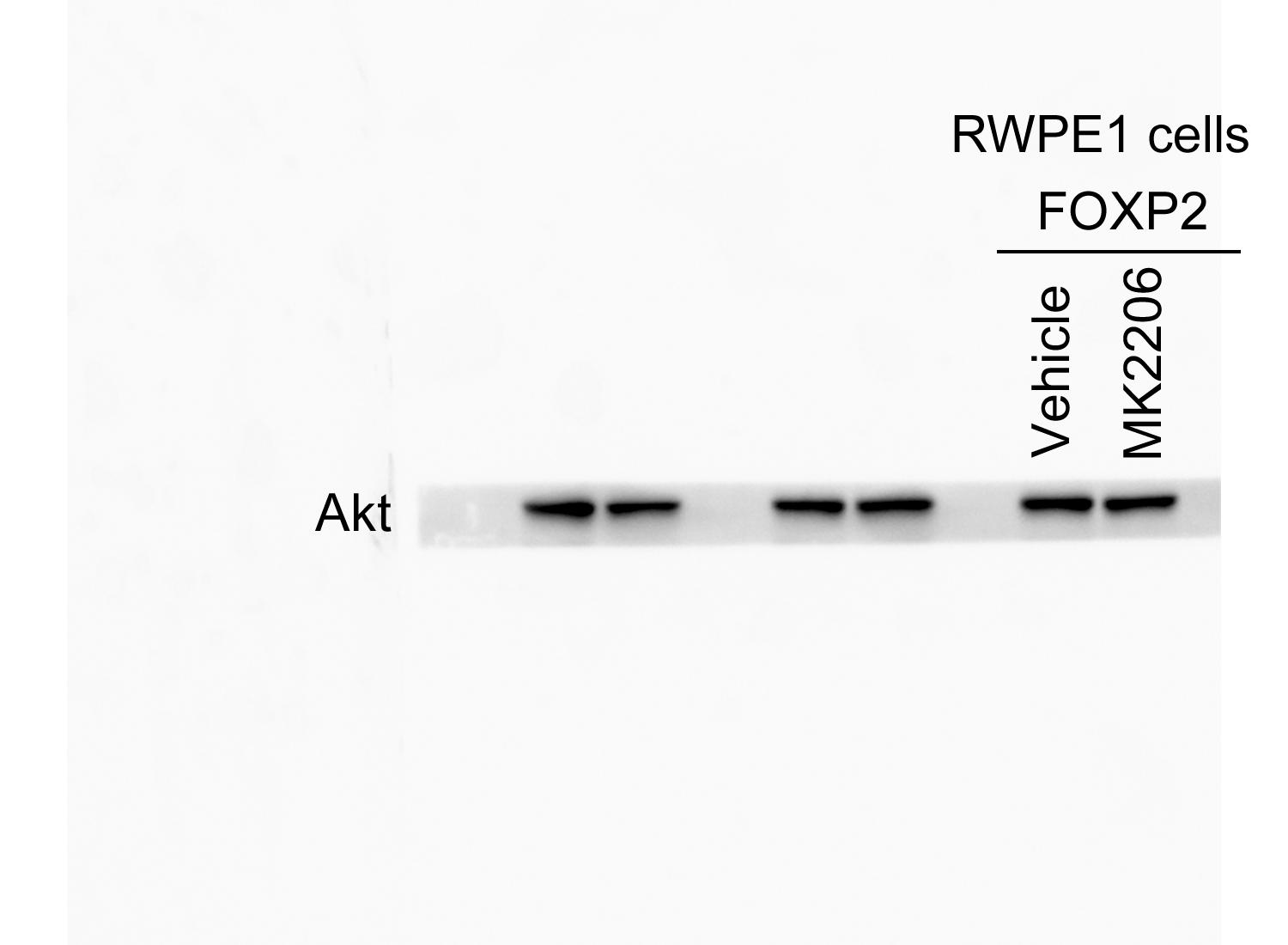

Supplement: Figure 3—source data 5. [file elife-81258-fig3-data5.zip › Figure 3-source data 5/Uncropped blots for Figure 3G in Main text/Figure 3G-source data 2.tif]

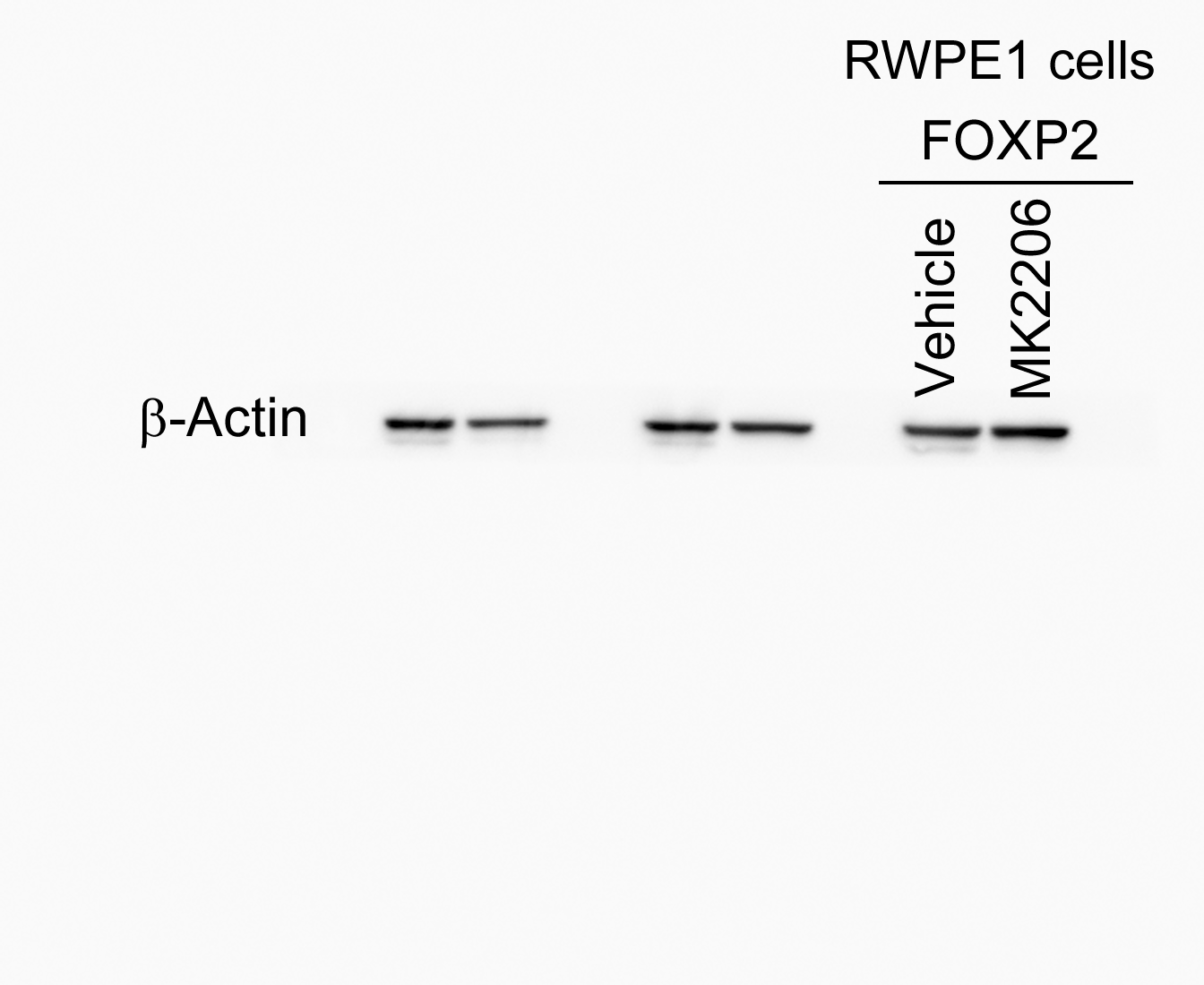

Supplement: Figure 3—source data 5. [file elife-81258-fig3-data5.zip › Figure 3-source data 5/Uncropped blots for Figure 3G in Main text/Figure 3G-source data 3.tif]

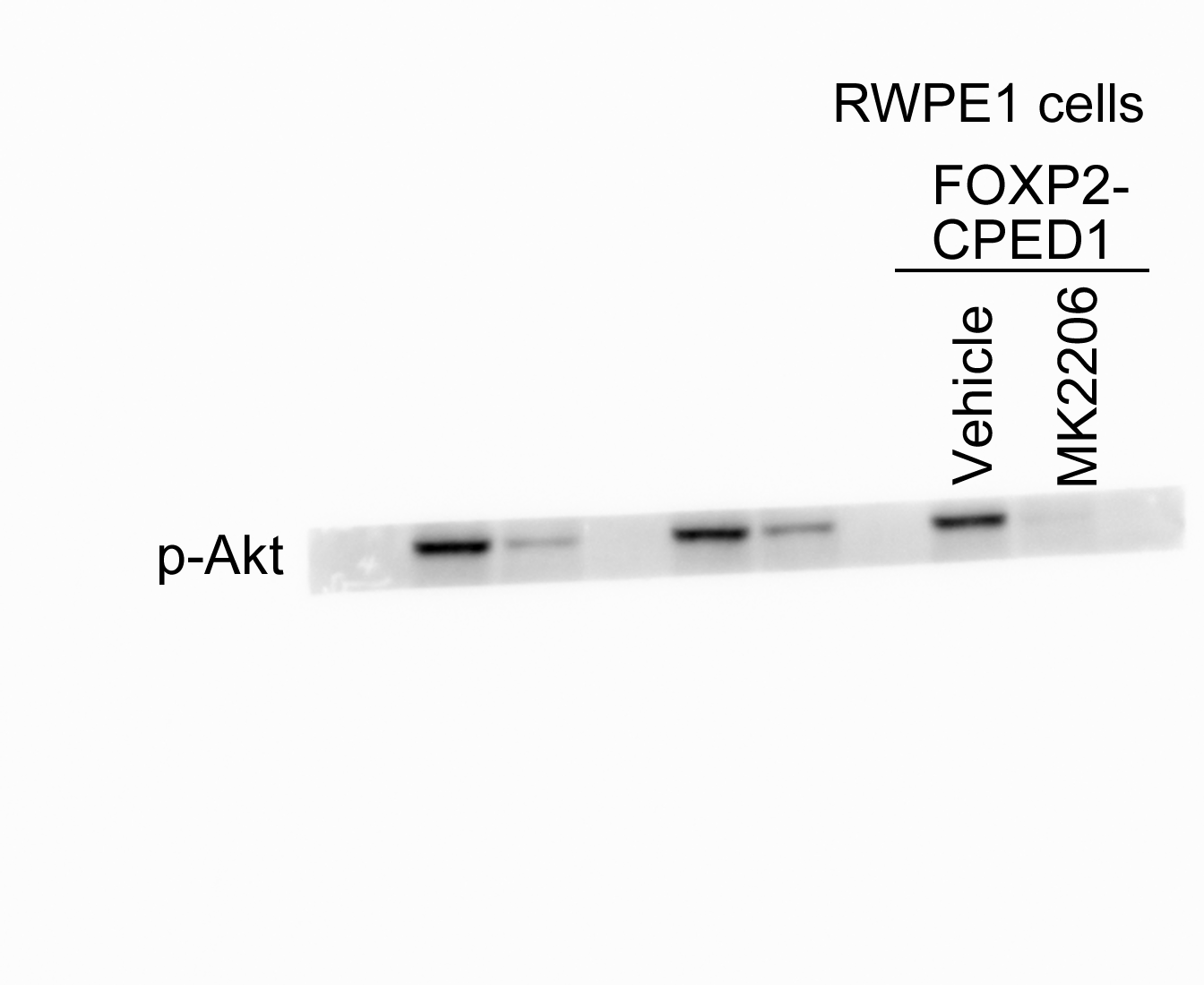

Supplement: Figure 3—source data 5. [file elife-81258-fig3-data5.zip › Figure 3-source data 5/Uncropped blots for Figure 3G in Main text/Figure 3G-source data 4.tif]

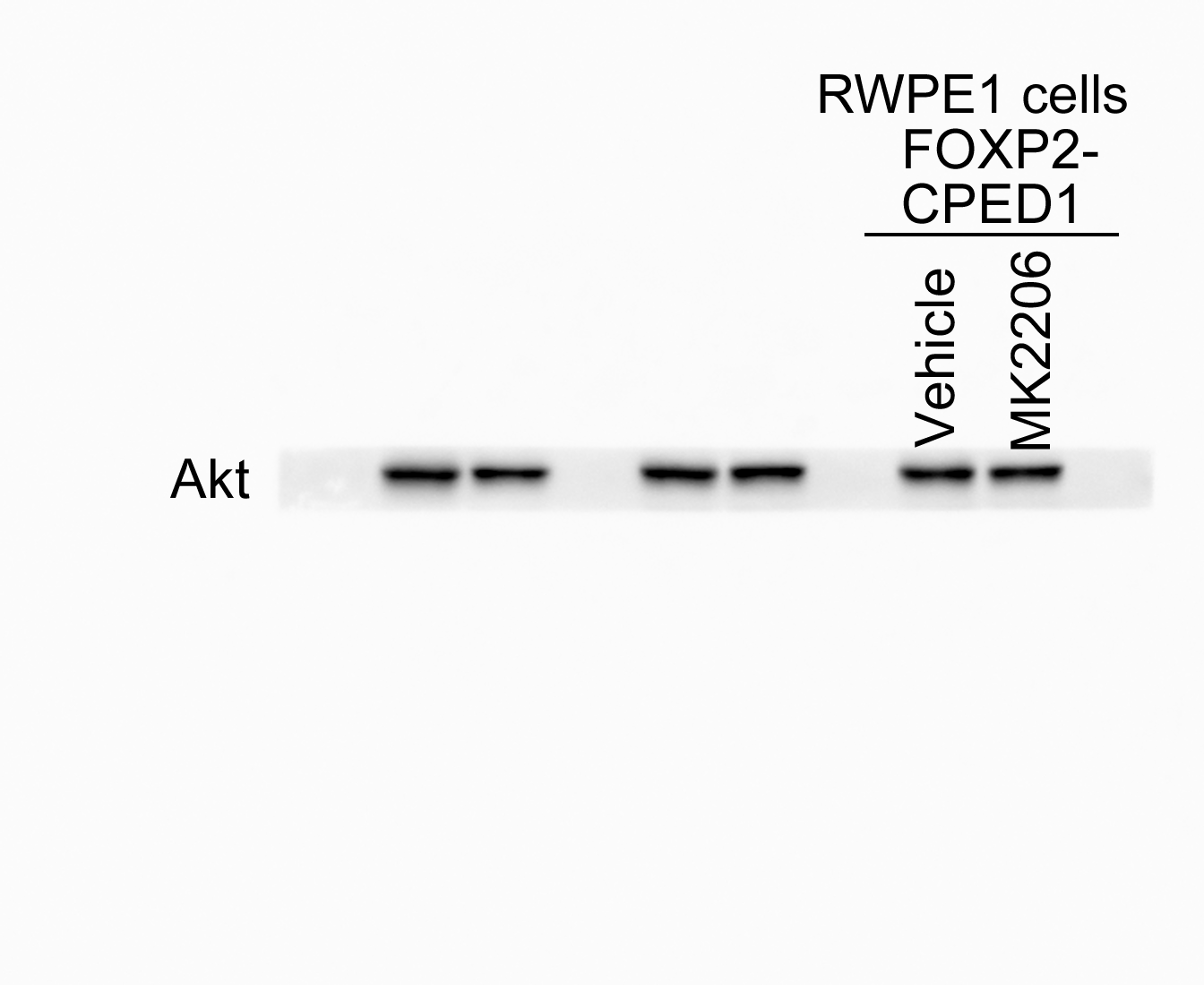

Supplement: Figure 3—source data 5. [file elife-81258-fig3-data5.zip › Figure 3-source data 5/Uncropped blots for Figure 3G in Main text/Figure 3G-source data 5.tif]

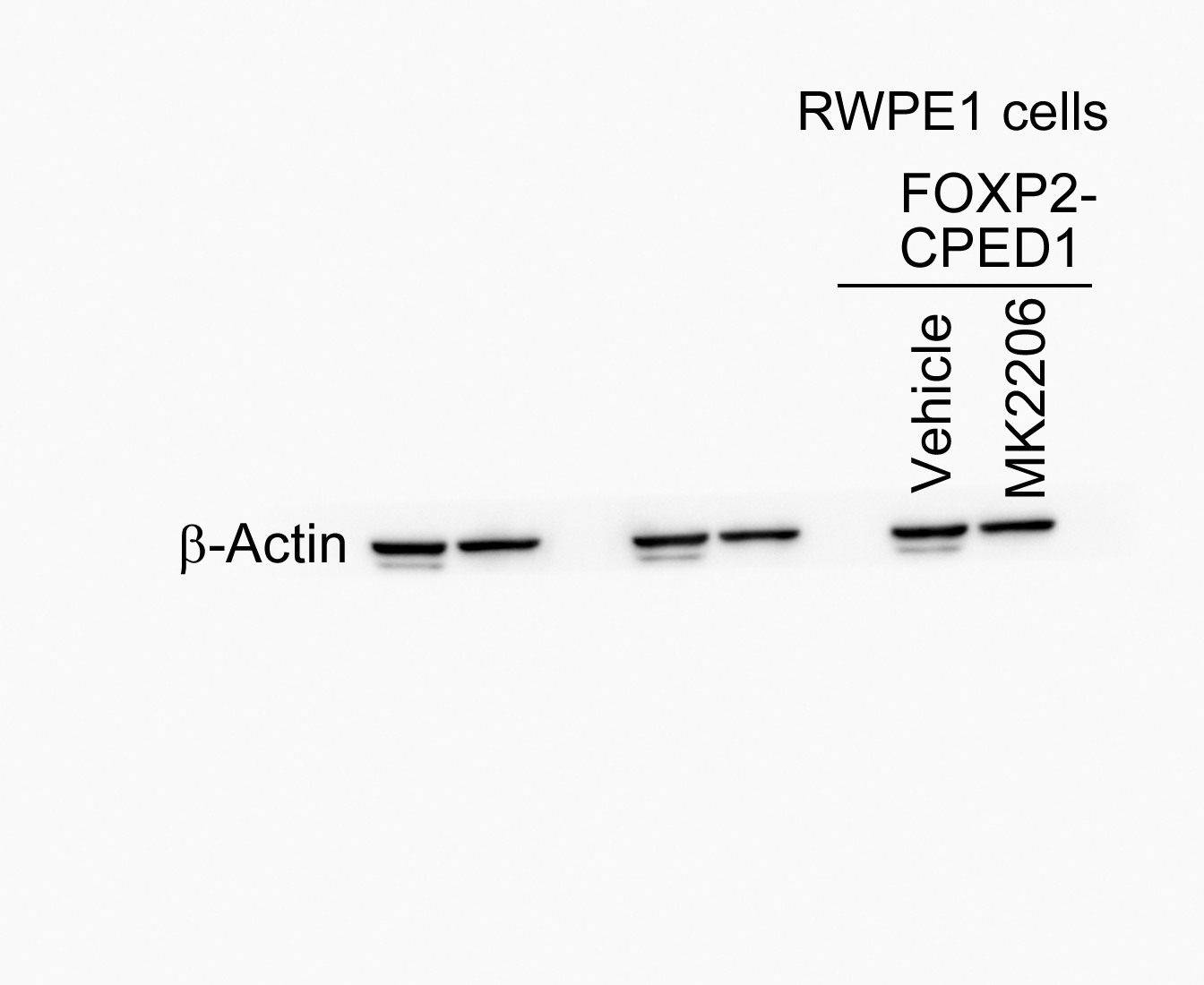

Supplement: Figure 3—source data 5. [file elife-81258-fig3-data5.zip › Figure 3-source data 5/Uncropped blots for Figure 3G in Main text/Figure 3G-source data 6.tif]

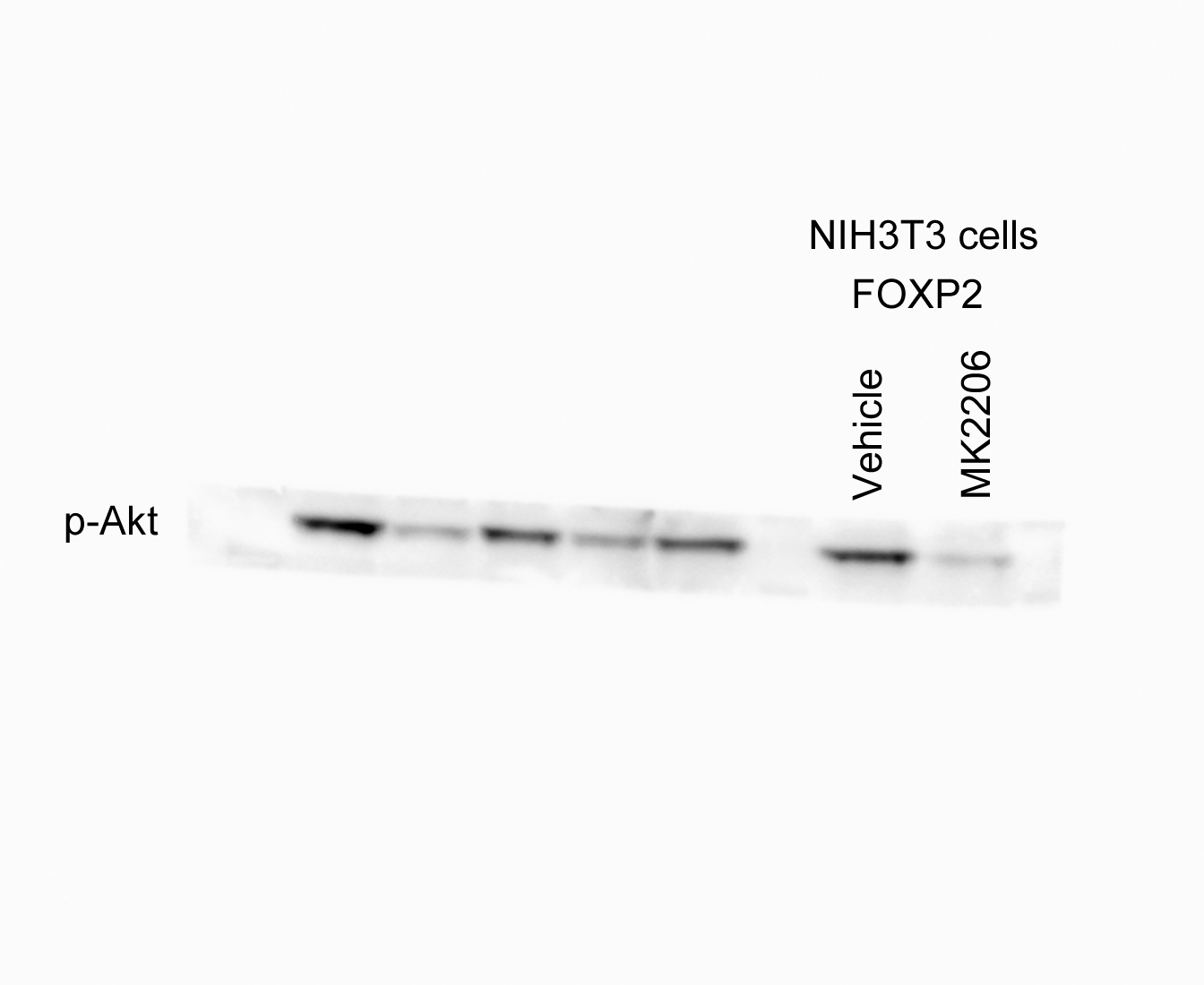

Supplement: Figure 3—source data 5. [file elife-81258-fig3-data5.zip › Figure 3-source data 5/Uncropped blots for Figure 3G in Main text/Figure 3G-source data 7.tif]

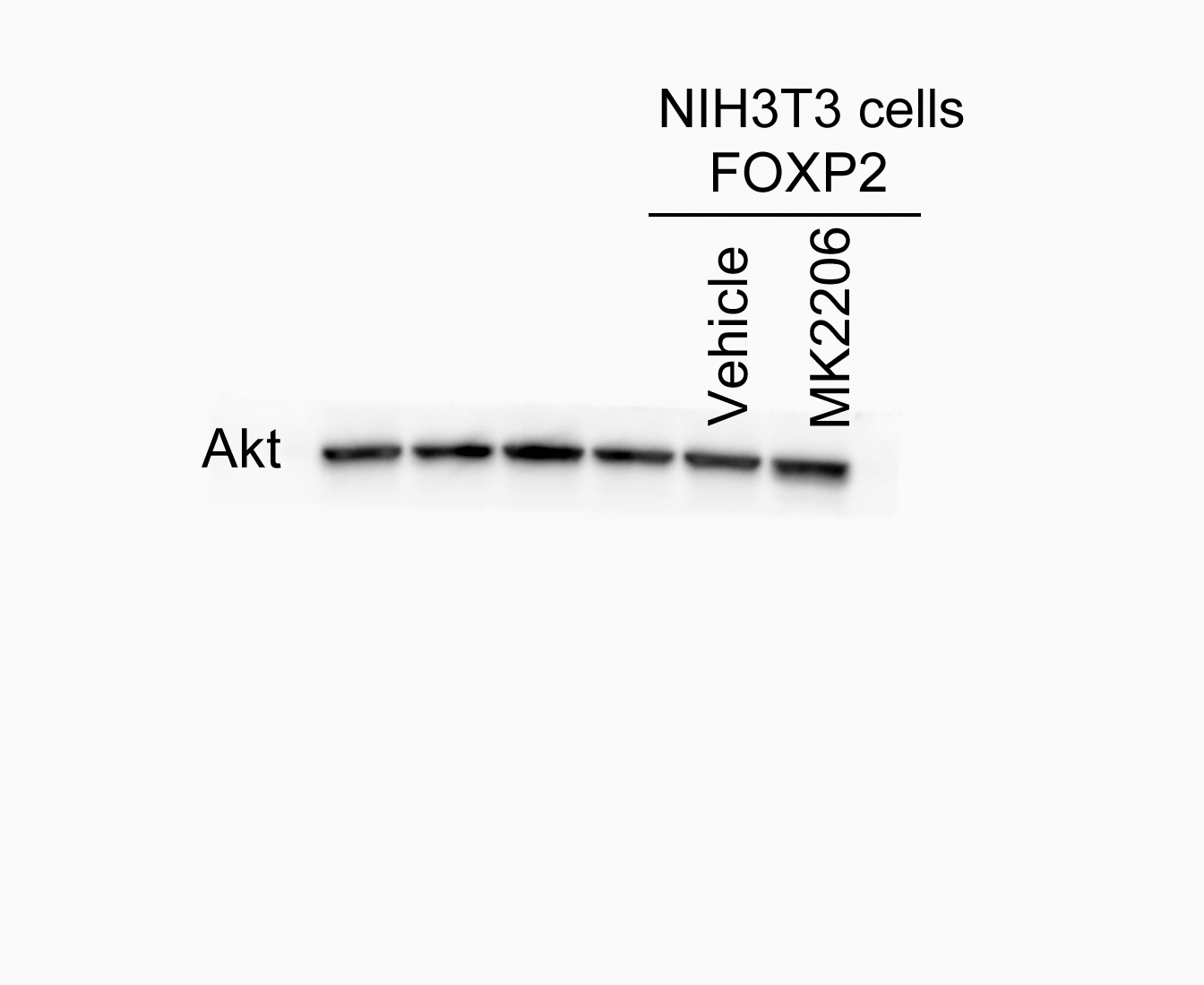

Supplement: Figure 3—source data 5. [file elife-81258-fig3-data5.zip › Figure 3-source data 5/Uncropped blots for Figure 3G in Main text/Figure 3G-source data 8.tif]

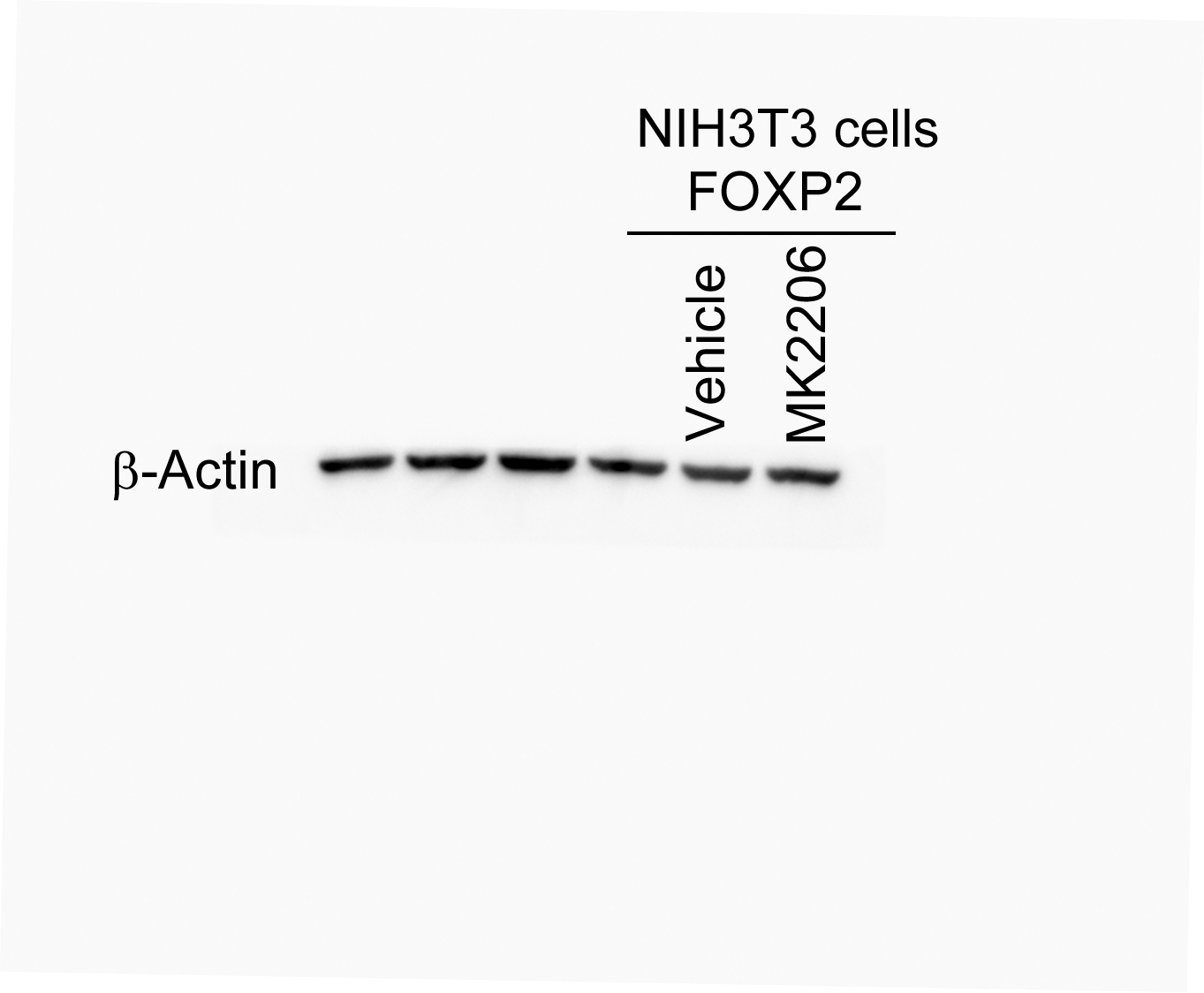

Supplement: Figure 3—source data 5. [file elife-81258-fig3-data5.zip › Figure 3-source data 5/Uncropped blots for Figure 3G in Main text/Figure 3G-source data 9.tif]

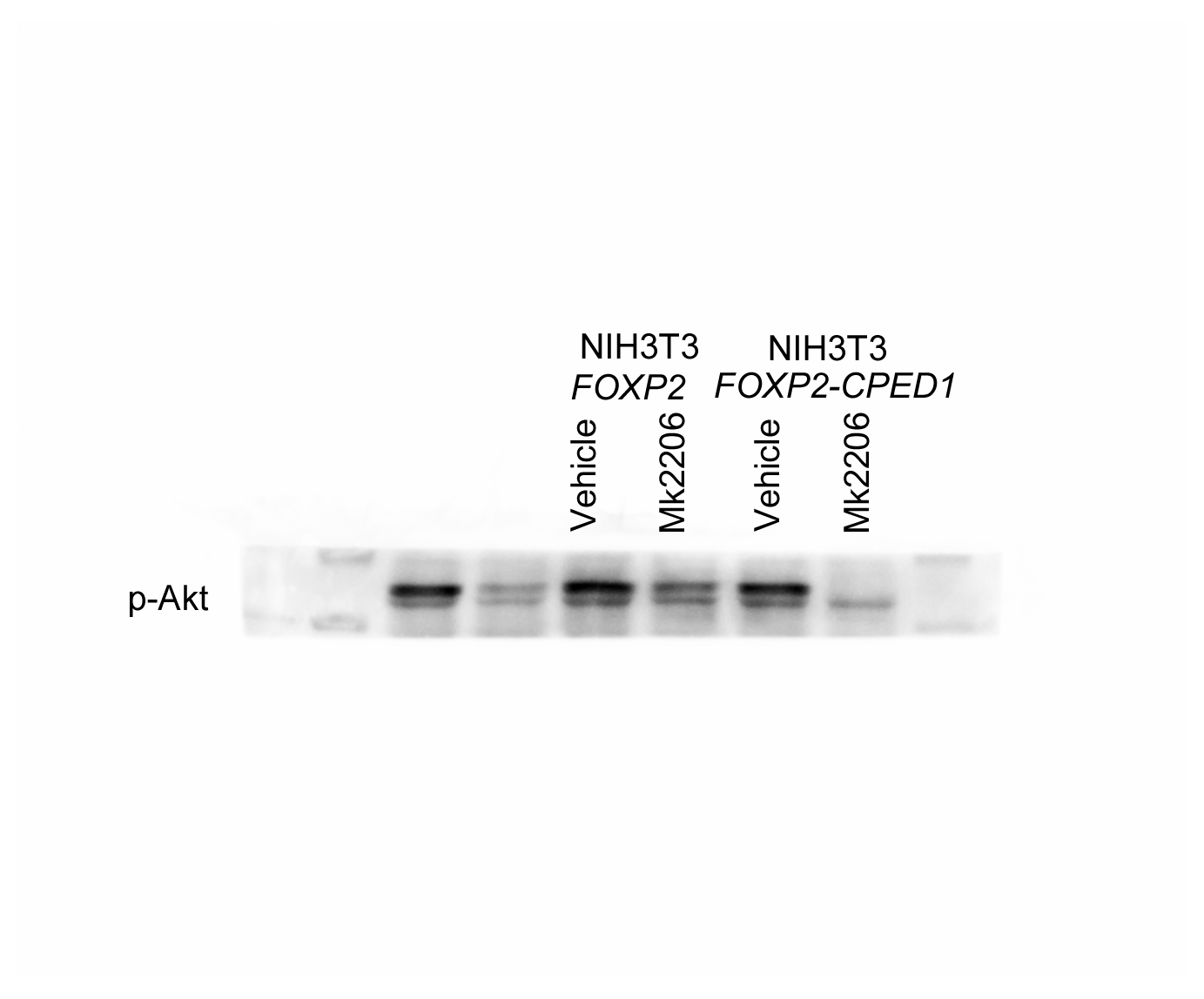

Supplement: Figure 3—source data 5. [file elife-81258-fig3-data5.zip › Figure 3-source data 5/Uncropped blots for Figure 3G NIH3T3 cells repeat/repeat/P-AKT FOXP2 and FOXP2-CPED1.tif]

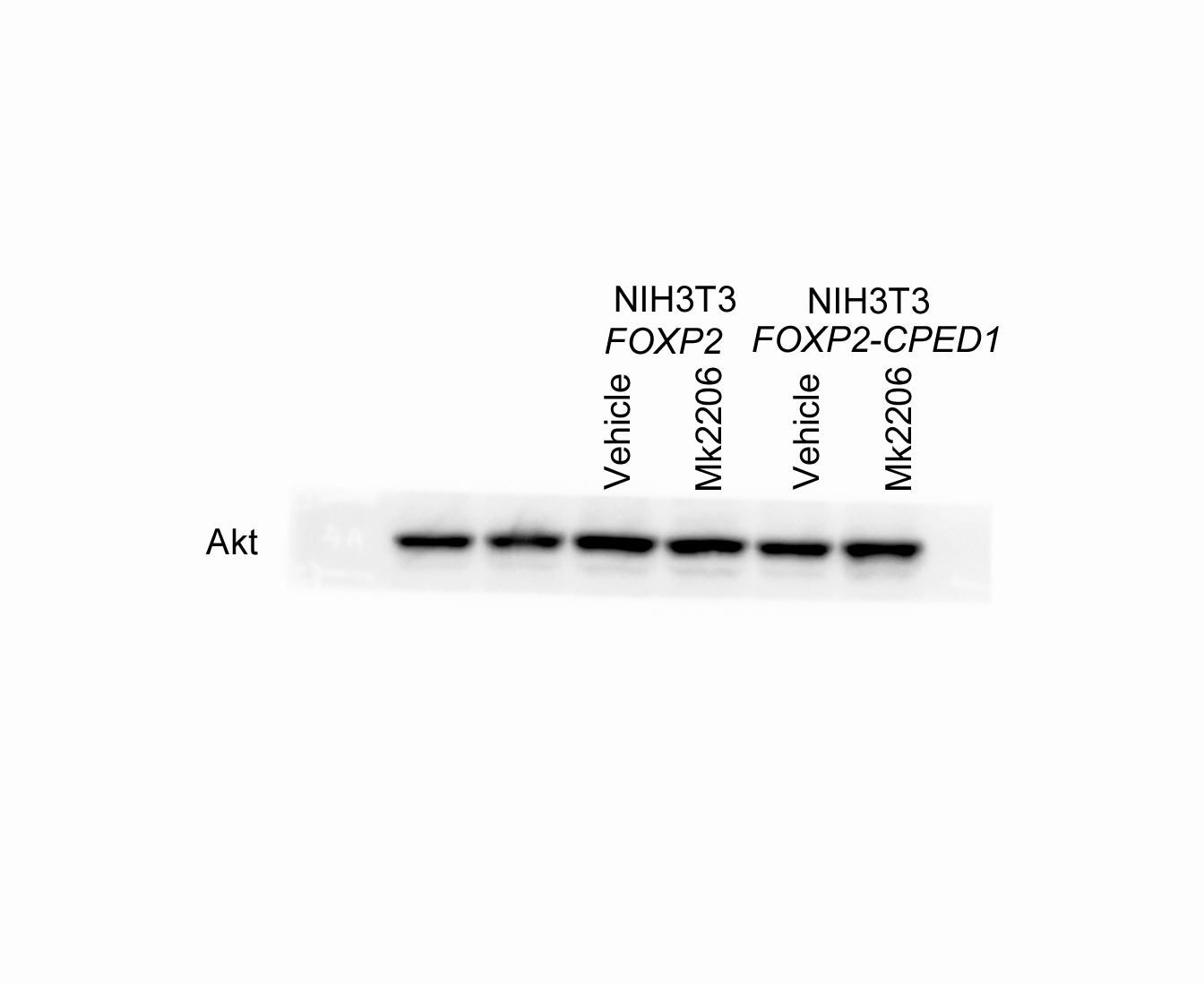

Supplement: Figure 3—source data 5. [file elife-81258-fig3-data5.zip › Figure 3-source data 5/Uncropped blots for Figure 3G NIH3T3 cells repeat/repeat/Total-AKT FOXP2 and FOXP2-CPED1.tif]

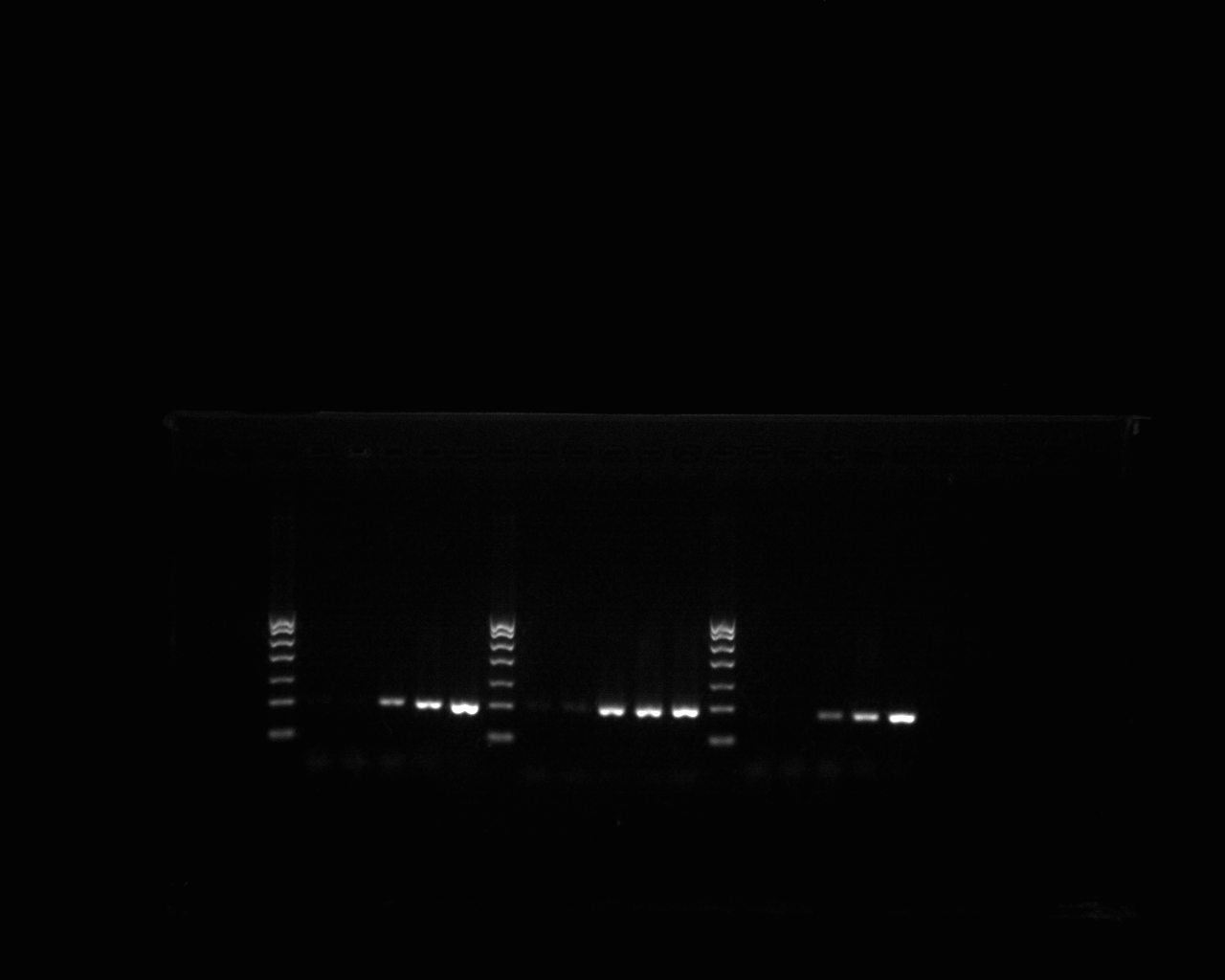

Supplement: Figure 3—figure supplement 1—source data 3. [file elife-81258-fig3-figsupp1-data3.zip › Figure 3-figure supplement 1-source data 3/Figure 3-figure supplement 1-source data 3/raw gel for Figure3-figure supplement 1D.Tif]

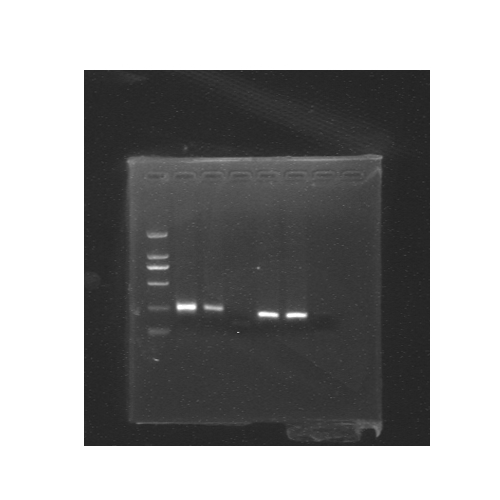

Supplement: Figure 4—figure supplement 1—source data 1. [file elife-81258-fig4-figsupp1-data1.zip › Figure 4-figure supplement 1-source data 1/raw gel for Figure4-figure supplement 1C.jpg]

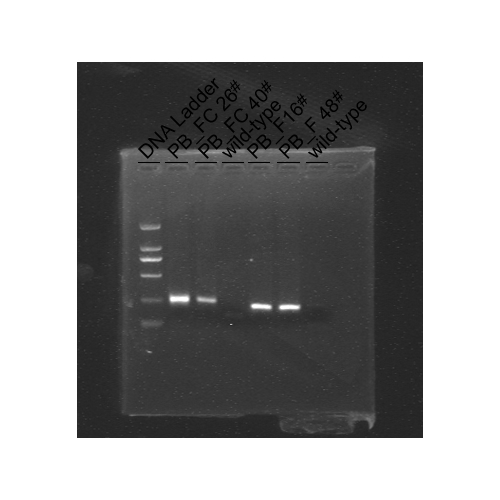

Supplement: Figure 4—figure supplement 1—source data 1. [file elife-81258-fig4-figsupp1-data1.zip › Figure 4-figure supplement 1-source data 1/uncropped gel for Figure4-figure 1C.jpg]

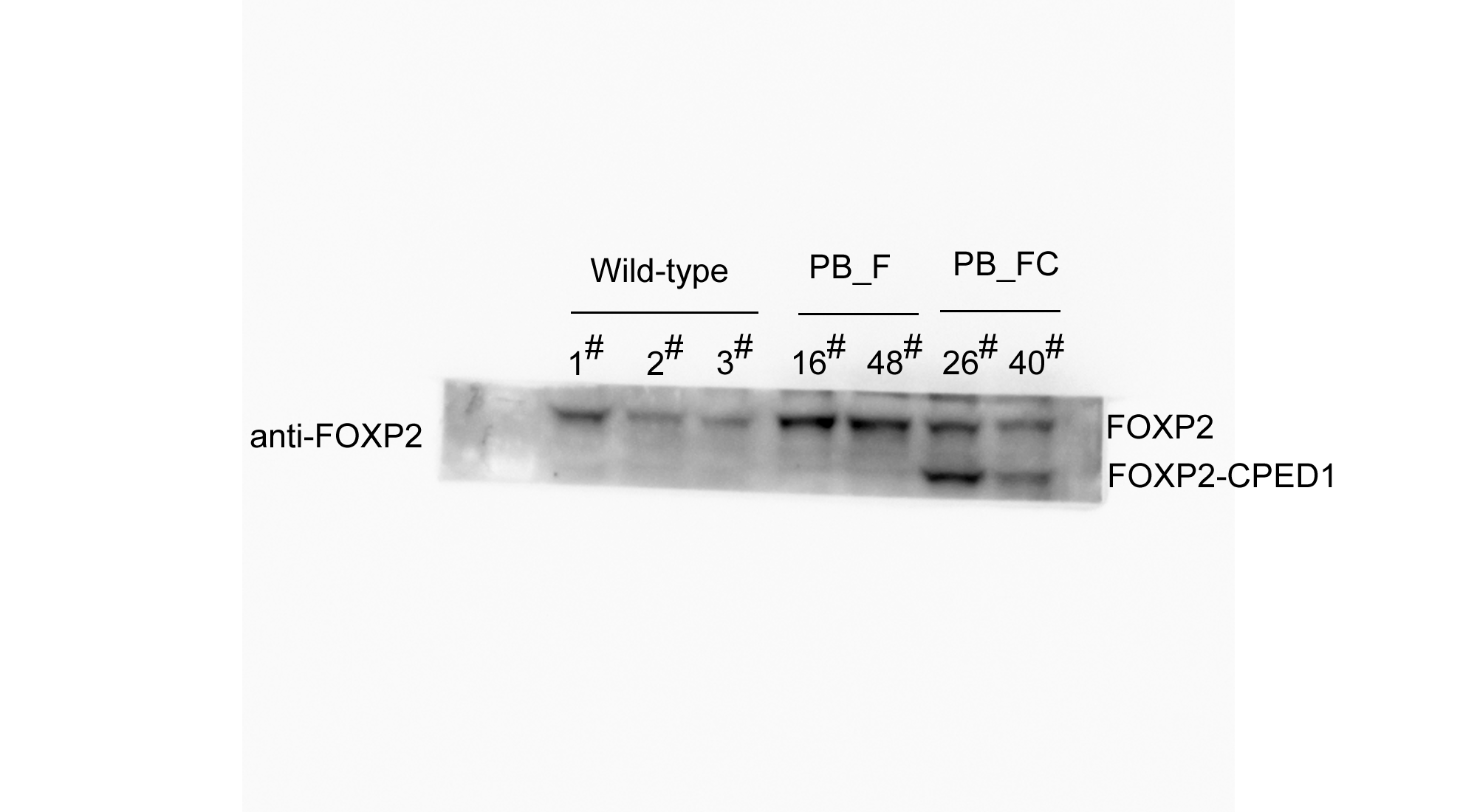

Supplement: Figure 4—figure supplement 1—source data 2. [file elife-81258-fig4-figsupp1-data2.zip › Figure 4-figure supplement 1-source data 2/Uncropped blots for Figure4-figure supplement 1D/anti-Foxp2.tif]

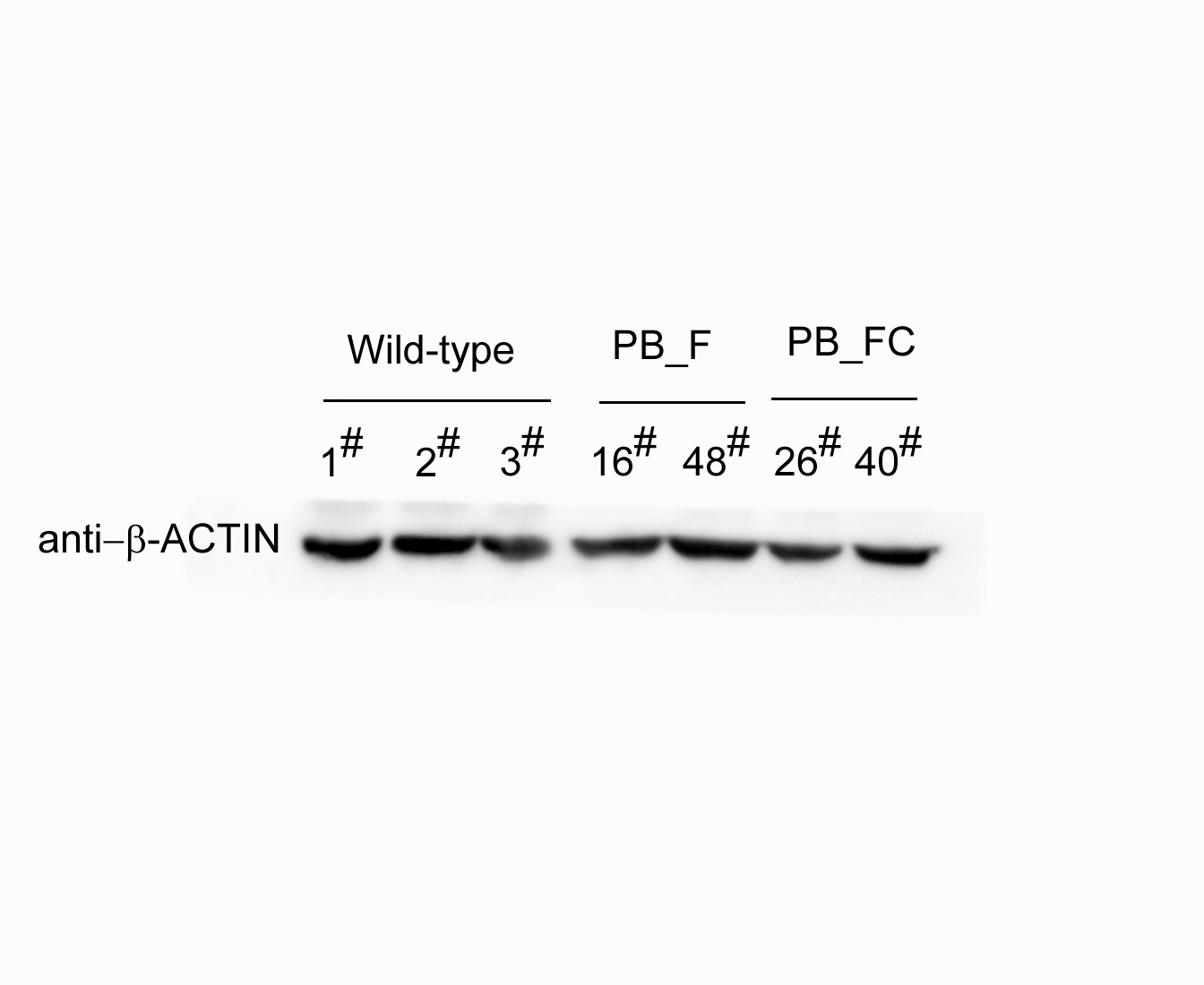

Supplement: Figure 4—figure supplement 1—source data 2. [file elife-81258-fig4-figsupp1-data2.zip › Figure 4-figure supplement 1-source data 2/Uncropped blots for Figure4-figure supplement 1D/anti-β-Actin.tif]
