# Supplementary material for: FOXP2 confers oncogenic effects in prostate cancer
Source: eLife. 2023 Sep 5;12:e81258. doi: 10.7554/eLife.81258 (PMC10513481; doi:10.7554/eLife.81258)
Supplement: Supplementary file 1. [file elife-81258-supp1.docx]

**Supplement file 1a. Clinical information of 10 localized prostate adenocarcinoma samples by RNA-Seq**

| **Sample ID** | **Age at Diagnosis (years)** | **Type** | **Histological Diagnosis** | **tPSA (ng/ml)** | **Gleason**  **Score** | **T**  **Stage** |
| --- | --- | --- | --- | --- | --- | --- |
| PC_1 | 71 | PRIMARY | Prostate Adenocarcinoma Acinar Type | 42.02 | 7 | T2c |
| PC_2 | 58 | PRIMARY | Prostate Adenocarcinoma Acinar Type | N/A | 8 | T3b |
| PC_3 | 70 | PRIMARY | Prostate Adenocarcinoma Acinar Type | 9.548 | 7 | T2c |
| PC_4 | 74 | PRIMARY | Prostate Adenocarcinoma Acinar Type | N/A | 8 | T3b |
| PC_5 | 63 | PRIMARY | Prostate Adenocarcinoma Acinar Type | 9.535 | 6 | T2c |
| PC_6 | 72 | PRIMARY | Prostate Adenocarcinoma Acinar Type | 10.784 | 7 | T2b |
| PC_7 | 73 | PRIMARY | Prostate Adenocarcinoma Acinar Type | 35.146 | 9 | T2c |
| PC_8 | 69 | PRIMARY | Prostate Adenocarcinoma Acinar Type | 6.933 | 7 | T3a |
| PC_9 | 66 | PRIMARY | Prostate Adenocarcinoma Acinar Type | N/A | 7 | T3a |
| PC_10 | 70 | PRIMARY | Prostate Adenocarcinoma Acinar Type | 6.17 | 6 | T2c |

**Supplement file 1b. New fusion genes identified and verified by RNA-Seq and RT-PCR-Sanger sequencing**

| **Sample** | **5' partner gene** | **5' partner gene Chr** | **3' partner gene** | **3' partner gene Chr** | **Fusion direction** | **Distance (Mb)** | **Span reads** | **Junc**  **reads** |
| --- | --- | --- | --- | --- | --- | --- | --- | --- |
| PC_7 | *IGF2R* | chr6 | *SLC22A2* | chr6 | fwd, rev | 0.13 | 46 | 31 |
| **PC_1** | ***FOXP2*** | **chr7** | ***CPED1*** | **chr7** | **fwd, fwd** | **6.38** | **5** | **17** |
| PC_7 | *RARS2* | chr6 | *SNAP91* | chr6 | rev, rev | 3.95 | 6 | 5 |
| PC_3 | *HCG27* | chr6 | *DDR1* | chr6 | fwd, fwd | 0.31 | 12 | 1 |
| PC_3 | *DGKD* | chr2 | *INPP5D* | chr2 | fwd, fwd | 0.24 | 9 | 3 |
| PC_3 | *GTF2I* | chr7 | *LIMK1* | chr7 | fwd, fwd | 5.52 | 1 | 5 |
| PC_5 | *ETV3* | chr1 | *CRB1* | chr1 | rev, fwd | 40.21 | 1 | 2 |
| PC_3 | *REPS2* | chrX | *TXLNG* | chrX | fwd, fwd | 1.88 | 1 | 1 |

Note: rev, reverse; fwd, forward

**Supplement file 1c. Gene set enrichment analysis of 3206 *FOXP2* expression-correlated genes**

| **GENE SETS link to MSigDB** | **SIZE** | **NES** | **FDR**  **q-val** | **FWER**  ***P*-val** |
| --- | --- | --- | --- | --- |
| WALLACE_PROSTATE_CANCER_RACE_UP | 267 | 6.93 | 0 | 0 |
| ACEVEDO_FGFR1_TARGETS_IN_PROSTATE_CANCER_MODEL_DN | 297 | 5.26 | 0 | 0 |
| KONDO_PROSTATE_CANCER_WITH_H3K27ME3 | 153 | 3.66 | 0 | 0 |
| ACEVEDO_FGFR1_TARGETS_IN_PROSTATE_CANCER_MODEL_UP | 273 | 3.34 | 0 | 0 |
| KRAS.PROSTATE_UP.V1_DN | 131 | 3.05 | 0 | 0 |
| GO_PROSTATE_GLAND_DEVELOPMENT | 40 | 2.97 | 0 | 0 |
| LIU_VAV3_PROSTATE_CARCINOGENESIS_UP | 86 | 2.86 | 0 | 0 |
| LI_PROSTATE_CANCER_EPIGENETIC | 30 | 2.72 | 0 | 0 |
| KEGG_PROSTATE_CANCER | 88 | 2.29 | 0.002 | 0.012 |
| YAMASHITA_METHYLATED_IN_PROSTATE_CANCER | 54 | 2.23 | 0.002 | 0.015 |
| OUYANG_PROSTATE_CANCER_PROGRESSION_UP | 18 | 2.13 | 0.004 | 0.038 |

**Supplement file 1d. Pathway analysis of 3206 *FOXP2* expression-correlated genes in 255 primary prostate adenocarcinomas from TCGA using gene sets from the MSigDB and the KEGG**

| **Biological pathway ID** | ***FOXP2* expression correlated genes** | **Adjusted_*P* (Bonferroni)** | **FDR** |
| --- | --- | --- | --- |
| DAIRKEE_TERT_TARGETS_UP | positive correlation | 3.16E-93 | 3.16E-93 |
| KEGG PI3K-AKT SIGNALNG | positive correlation | 1.38E-26 | 4.62E-27 |
| DEBIASI_APOPTOSIS_BY_REOVIRUS_INFECTION_DN | positive correlation | 7.76E-14 | 1.55E-14 |
| HALLMARK_MITOTIC_SPINDLE | positive correlation | 1.62E-10 | 2.33E-11 |
| PID_KIT_PATHWAY | positive correlation | 1.63E-10 | 2.33E-11 |
| BIOCARTA_MET_PATHWAY | positive correlation | 2.43E-08 | 2.86E-09 |
| HALLMARK_TGF_BETA_SIGNALLING | positive correlation | 2.57E-08 | 2.86E-09 |
| HALLMARK_INFLAMMATORY_RESPONSE | positive correlation | 3.72E-06 | 9.34E-06 |
| HALLMARK_E2F_TARGETS | negative correlation | 1.96E-07 | 1.96E-08 |
| HALLMARK_DNA_REPAIR | negative correlation | 3.50E-15 | 8.75E-16 |
| HALLMARK_OXIDATIVE_PHOSPHORYLATION | negative correlation | 3.87E-54 | 1.94E-54 |

**Supplement file 1e**. **Summary of prostate features in ARR2PB-*FOXP2* and ARR2PB-*FOXP2-CPED1* transgenic mice.**

| **ARR2PB-*FOXP2* mice** | **Age** | **Normal** | **Hyperplasia** | **PIN** |
| --- | --- | --- | --- | --- |
| Pb-F59 | 46 wk | VP, LP | AP, DP | AP |
| Pb-F67 | 52 wk | AP | LP | LP |
| Pb-F69 | 52 wk |  | AP, VP | AP, VP |
| Pb-F70 | 52 wk | AP | VP | VP |
| Pb-F72 | 52 wk |  | VP | VP |
| Pb-F82 | 60 wk |  | VP, LP | VP, LP |
| Pb-F87 | 60 wk |  | VP | VP |
| Pb-F94 | 52 wk | LP | AP, DP, DP |  |
| Pb-F101 | 57 wk |  | AP | AP |
| Pb-F102 | 57 wk |  | AP, VP | AP, VP |
| Pb-F108 | 61 wk |  | AP, VP, DP | AP, VP |
| Pb-F110 | 61 wk |  | AP | AP |
| Pb-F115 | 63 wk |  | AP | AP |
| Pb-F117 | 63 wk |  | AP, VP | AP, VP |
| Pb-F125 | 63 wk |  | AP | AP |
| Pb-F140 | 62 wk |  | VP, DP | DP |
| Pb-F142 | 62 wk |  | AP, VP | AP, VP |
| Pb-F145 | 64 wk |  | AP, VP, LP | VP, LP |
| Pb-F146 | 63 wk |  | AP, VP, DP | AP, VP, DP |
| Pb-F148 | 63 wk |  | AP | AP |
| Pb-F149 | 63 wk |  | AP, VP | AP, VP |
| Pb-F150 | 63 wk |  | AP, VP | AP, VP |
| Pb-F153 | 63 wk | VP, LP | AP | AP |
| Pb-F154 | 63 wk |  | AP, VP | VP |
| Pb-F155 | 63 wk |  | AP, VP | AP, VP |
| Pb-F157 | 59 wk |  | VP | VP |
| Pb-F160 | 59 wk |  | AP, VP | AP, VP |
| Pb-F162 | 59 wk |  | AP, VP | AP, VP |
| Pb-F165 | 59 wk |  | AP, VP | VP |
| Pb-F166 | 59 wk |  | AP, VP | VP |
| Pb-F170 | 60 wk |  | AP, VP | VP |
| Pb-F171 | 60 wk |  | AP, VP | VP |
| Pb-F176 | 61 wk |  | AP, VP, DP | AP, VP, DP |
| Pb-F178 | 61 wk | LP | AP | AP |
| Pb-F181 | 61 wk |  | AP, VP | VP |
| **Total (35 mice)** |  |  | **100% (35/35)** | **97% (34/35)** |
|  | | | | |
| **ARR2PB-*FOXP2-CPED1* mice** | **Age** | **Normal** | **Hyperplasia** | **PIN** |
| Pb-FC53 | 52 wk |  | AP, LP | AP, LP |
| Pb-FC58 | 52 wk |  | AP |  |
| Pb-FC59 | 52 wk |  | VP | VP |
| Pb-FC61 | 52 wk |  | AP, VP | VP |
| Pb-FC62 | 52 wk |  | AP | AP |
| Pb-FC65 | 51 wk |  | LP, DP | DP |
| Pb-FC68 | 51 wk |  | AP | AP |
| Pb-FC70 | 52 wk |  | AP | AP |
| Pb-FC75 | 84 wk |  | AP, VP, DP | AP, VP |
| Pb-FC85 | 65 wk |  | AP, VP | VP |
| Pb-FC88 | 65 wk |  | AP, VP | AP, VP |
| Pb-FC95 | 62 wk |  | AP, VP |  |
| Pb-FC96 | 62 wk |  | AP, VP | AP, VP |
| Pb-FC100 | 60 wk |  | AP, VP, LP, DP | AP, VP, LP, DP |
| Pb-FC110 | 64 wk |  | VP | VP |
| Pb-FC111 | 64 wk |  | LP, VP | VP |
| Pb-FC112 | 64 wk |  | AP, VP | AP, VP |
| Pb-FC116 | 64 wk |  | AP, LP, DP | AP, LP, DP |
| Pb-FC118 | 64 wk |  | AP, VP | AP, VP |
| Pb-FC121 | 64 wk |  | AP, VP |  |
| Pb-FC122 | 64 wk |  | AP | AP |
| Pb-FC124 | 64 wk |  | AP, VP, DP | VP |
| Pb-FC125 | 62 wk |  | AP, VP | AP, VP |
| Pb-FC127 | 62 wk | VP, LP | AP | AP |
| Pb-FC129 | 59 wk |  | VP | AP, VP |
| Pb-FC131 | 58 wk |  | VP | VP |
| Pb-FC135 | 58 wk |  | VP | AP, VP |
| Pb-FC136 | 58 wk |  | VP | VP |
| Pb-FC138 | 58 wk |  | AP, VP, DP | AP, VP |
| Pb-FC140 | 58 wk | DP | AP, VP |  |
| Pb-FC144 | 57 wk | AP | VP | VP |
| Pb-FC147 | 57 wk |  | AP | AP |
| **Total (32 mice)** |  |  | **100 % (32/32)** | **88 % (28/32)** |

**Supplement file 1f**. Summary of primers used in this study

| **Primers for breakpoint of *FOXP2-CPED1* at the mRNA level** | |
| --- | --- |
| Forward | 5' CTTAGCCTGCACAAGTGTTTTGTTCG 3' |
| Reverse | 5' TGGCTGCTGTATTTCTGGTAATCTG 3' |
| **Primers for breakpoint of *FOXP2-CPED1* at the genomic DNA level** | |
| geno Forward | 5' CAGGAAAAGTAAATACTTTAAGTTGG 3' |
| geno Reverse1 | 5' GTAAAAGGAAGTGGAGTAGGGTTC 3' |
| geno Reverse2 | 5' GAATACCAAGACTTTCAGAGGATAAG 3' |
| geno Reverse3 | 5' GAGACAGTGTAAGAGGGAAGCA 3' |
| geno Reverse4 | 5' CTCTATACTTCCATAGAACAACAGG 3' |
| geno Reverse5 | 5' TCTATGTGTCTTGCCAGCCT 3' |
| geno Reverse6 | 5' CCCATGCTCATGGATGAGTAG 3' |
| geno Reverse7 | 5' GGGAGATTCCAGTGCTTTACA 3' |
| geno Reverse8 | 5' TGATGTAAACCTAACTGGCACATGAC3' |
| **Primers of *FOXP2* for 3'RACE-PCR** | |
| *Foxp2* 6exon F | 5' GTCAGGGACTCATCTCCATTCCA 3' |
| *Foxp2* 11exon F | 5' GACATTCAGACAAATACAACATTCC 3' |
| *Foxp2* 16exon F | 5' GACAGCAATGGAAACAGTAGTCC 3' |
| **Primers for qPCR** | |
| ***FOXP2*** | |
| Forward | 5' TGGAGTCATCTGACAGGCAGTTAACAC 3' |
| Reverse | 5' CCTGTTATCTTTTGTGACCTTCGCTTCTGG 3' |
| ***EIF4H*** | |
| Forward | 5' CAGCATAAGGAGTGTACGGCTAGTCAG 3' |
| Reverse | 5' GCAATGTCCACACGAAGTGACCGA 3' |
| ***HGF*** | |
| Forward | 5' CTGCAGACCAATGTGCTAATAGATGTACTAGGA 3' |
| Reverse | 5' ACTTGACATGCTATTGAAGGGGAACCAGA 3' |
| ***MET*** | |
| Forward | 5' CAGTGTGGCTGGTGCCACGACA 3' |
| Reverse | 5' TCCAAAGTCCCAGCCACATATGGTC 3' |
| ***PIK3R1*** | |
| Forward | 5' GAAAGACGAGAGACCAATACTTGATGTGGT 3' |
| Reverse | 5' GCTTTGTTTCGGTTGCTGCTTCCA 3' |
| ***PIK3CA*** | |
| Forward | 5' CACCTCATAGTAGAGCAATGTATGTCTATCCT 3' |
| Reverse | 5' CATTATTTGGAGAAACTATTACCCAGATCACC 3' |
| **Primers for constructions** | |
| **FLAG-cmv4_truncted *FOXP2 FOXP2-CPED1*)** | |
| Flag_Trunc_*FOXP2* *Not*I | 5' GCAGCGGCCGCGATGCAGGAATCTGCGACAGAGAC 3' |
| Flag_Trunc_*FOXP2* *Kpn*I | 5' CGGGGTACCTCACCCTGTTATCTTTTGTGACCTTCGC 3' |
| **FLAG-cmv4_*FOXP2-CPED1*** | |
| Flag_Trunc_*FOXP2* *Not*I | 5' GCAGCGGCCGCGATGCAGGAATCTGCGACAGAGAC 3' |
| Flag_*FOXP2-CPED1 Kpn*I | 5' CGTGGTACCCTACATAGTCCTTTTATTTGCACACA 3' |
| **pCDH_ *FOXP2*** | |
| pCDH_*FOXP2* *Xba*I | 5' CCGTCTAGAATGCAGGAATCTGCGACAGAGAC 3' |
| *FOXP2* NotI | 5' GCAGCGGCCGCTCATTCCAGATCTTCAGATAAAGGCTC 3' |
| **pCDH_ *FOXP2-CPED1*** | |
| pCDH_*FOXP2* *Xba*I | 5' CCGTCTAGAATGCAGGAATCTGCGACAGAGAC 3' |
| pCDH_*CPED1 Not*I | 5' GCAGCGGCCGCTACATAGTCCTTTTATTTGCACACA 3' |
| **pmirGlo_*FOXP2* 3'UTR** | |
| pmirGlo_3'UTR *Sac*I | 5' CTCGAGCTCGAACTGACTTGTGAAACCTCAGC 3' |
| pmirGlo_3'UTR *Xho*I | 5' GCACTCGAGGATTTTGTCCTCACCTCTGTC 3' |
| **pmirGlo_*FOXP2* mut 3'UTR** | |
| pmirGlo_3'UTR *Sac*I | 5' CTCGAGCTCGAACTGACTTGTGAAACCTCAGC 3' |
| pmirGlo_3'UTR *Xho*I | 5' GCACTCGAGGATTTTGTCCTCACCTCTGTC 3' |
| 3'UTR mut_R | 5' AATAGAAAAATTTGTCAAATATTCATGG 3' |
| 3'UTR mut_F | 5' TTGACAAATTTTTCTATTTATTAAGCATGGAT 3' |
| **Primers for detection of transgenic mice with prostate-specific expression of *FOXP2*** | |
| Forward (F1) | 5' TGGAGTCATCTGACAGGCAGTTAACAC 3' |
| Reverse (R1) | 5' CCTGTTATCTTTTGTGACCTTCGCTTCTGG 3' |
| **Primers for detection of transgenic mice with prostate-specific expression of *FOXP2-CPED1*** | |
| Forward (F2) | 5' CTTAGCCTGCACAAGTGTTTTGTTCG 3' |
| hGHtestR (R2) | 5’ GCTGGTGGGCACTGGAGTGGCA 3’ |
